# Exploring Alashan Ground Squirrel (*Spermophilus alashanicus*) Diversity: Metagenomic and Transcriptomic Datasets from the Helan Mountains

**DOI:** 10.1038/s41597-024-03183-6

**Published:** 2024-05-21

**Authors:** Yao Zhao, Siwei Deng, Zhirong Zhang, Junda Chen, Liwei Teng, Zhensheng Liu

**Affiliations:** 1 College of Wildlife and Protected Area, Northeast Forestry University, Harbin, 150040 China; 2 OxTium Technology Co., Ltd, Shenzhen, 518000 China; 3 Key Laboratory of Conservation Biology, National Forestry and Grassland Administration, Harbin, 150090 China

**Keywords:** RNA sequencing, Bacterial genes

## Abstract

This study investigates the adaptive strategies of the Alashan Ground Squirrel (*Spermophilus alashanicus*) in response to habitat changes, as rodents are sensitive indicators of ecosystem changes. Despite its ecological importance, the genome and microbiome of this species have not been thoroughly studied. This research fills this gap by presenting the first comprehensive metagenomic and transcriptomic datasets of the species. Transcriptomic data was collected from five tissue types, including heart, liver, cecum, muscle, and blood, resulting in the assembly of 72,156 unigenes. Metagenomic sequencing identified predominant bacterial groups such as Firmicutes, Bacteroidetes, Verrucomicrobia, Urovircota, and Proteobacteria. Our workflow involved RNA and DNA extraction, library preparation, assembly, and annotation, yielding valuable insights into gene discovery, microbial composition, and further genome and microbial function studies. In conclusion, our findings have significant implications for understanding the adaptive mechanisms of this species in response to environmental changes.

## Background & Summary

**Fig. 1:**
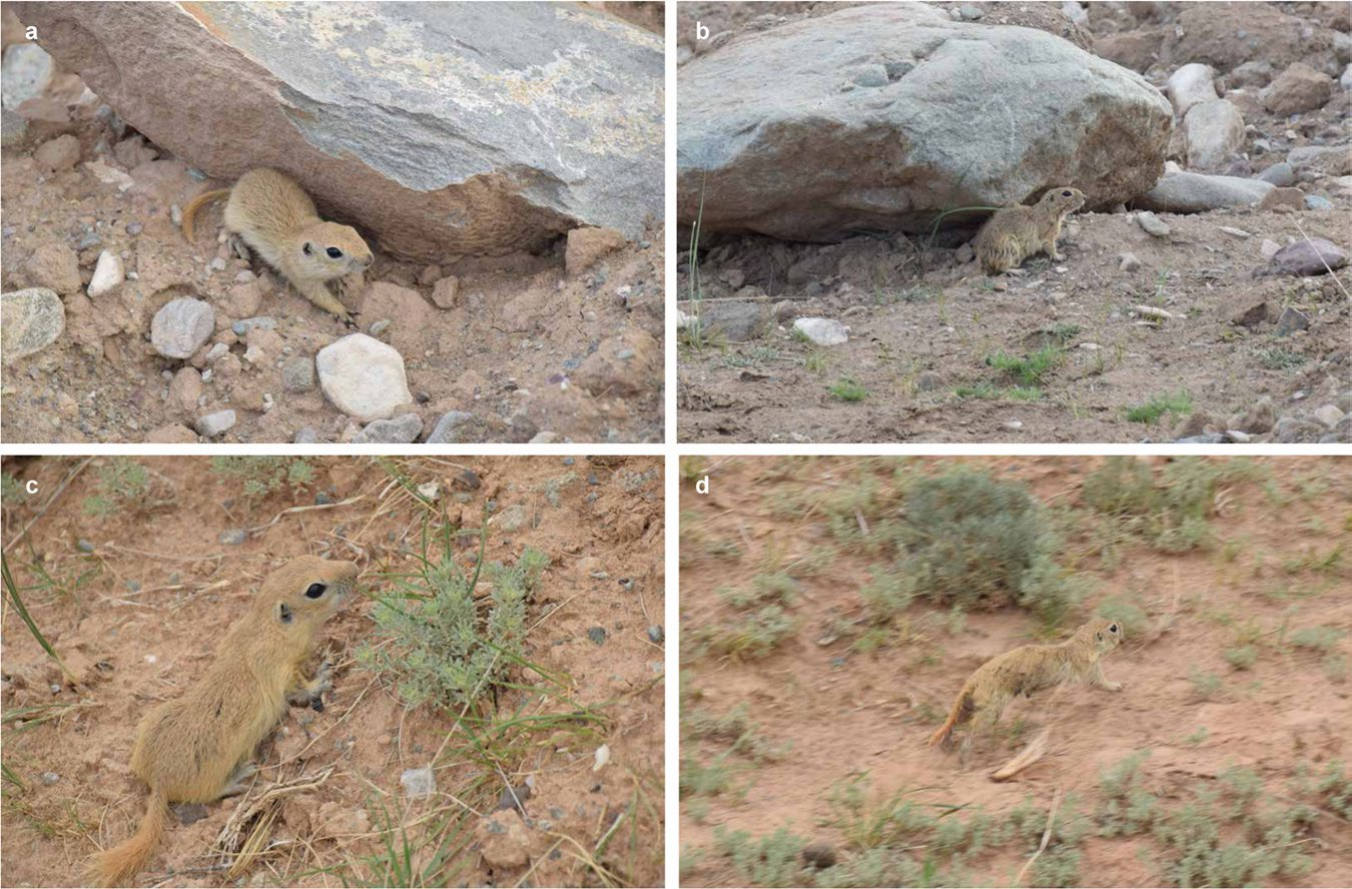
Alashan ground squirrels in the Helan Mountains. (**a**) and (**b**) was taken on the eastern slope. (**c**) and (**d**) taken for the western slope.

The Alashan Ground Squirrel (*Spermophilus alashanicus*), part of the Rodentia order and Sciuridae family, is a prevalent rodent species native to the Helan Mountains in China^1^. It thrives in forest grasslands and desert plains, predominantly consuming plants and insects. Characterised by its large, protruding eyes, degenerated outer ears, and hibernating behaviour (Fig. 1), it shares a close phylogenetic relationship with *Spermophilus dauricus*^2^. Although assessed for the IUCN Red List of Threatened Species in 2016^3^ (10.2305/IUCN.UK.2016-3.RLTS.T20478A22265832.en. Accessed on 09 December 2022), research on this species is limited due to its unique distribution, leaving its environmental adaptation mechanisms largely unexplored. Current studies are confined to individual identification^4^ and habitat suitability analysis^5^.

In the context of global climate change and its ecological repercussions, understanding the molecular mechanisms underlying adaptation to changing environments is crucial. However, until this study, limited molecular information has been available for the Alashan Ground Squirrel, particularly in metagenomic and transcriptomic domains, hindering the understanding of their biological mechanisms. This study introduces extensive metagenomic and transcriptomic datasets derived from high-throughput sequencing of squirrel specimens from different slopes of Helan Mountains. Specifically, we collected transcriptomic data from five different tissue types, including heart, liver, cecum, muscle, and blood, and metagenomic data from faecal contents tissues.

The Helan Mountains range, extending in a rare north-south direction, is a pivotal geographical feature dividing Northwest China^6^. The west slope, part of the Inner Mongolia Helan Mountains National Nature Reserve, is characterised by a gentle terrain, humid climate, and lush vegetation. Conversely, the east slope, falling under the Ningxia Helan Mountains National Nature Reserve, is noted for its steep incline, dry climate, high temperatures, and sparse vegetation. This dichotomy makes the area an ideal model for understanding how the squirrel responds to environmental changes. Especially, the transcriptional data can reflect the overall molecular response of different tissues, while the metagenomic data can reveal the metabolic and bacterial interactions when living in different environments.

Gut microbes play important roles in host health, such as immunity^7^, nutrient absorption^8^, and behaviour^9–11^. Different environmental pressures necessitate varying dietary and energy needs for animals within the same species, leading to corresponding changes in their gut microbiota^12,13^. At present, the research on rodents mainly focuses on experimental animals^14^, while the research on wild rodents is relatively limited. To better understand the functional interplay between gut microbes and their environment, we investigated both the metagenomics and transcriptomes of these squirrels. Our study provides a valuable resource for comprehending the role of gut microbiota in wild rodents.

This study provides the first comprehensive metagenomic and transcriptomic datasets of the Alashan Ground Squirrel. By bridging the knowledge gap in understanding the molecular information of this species, our aim is to provide insights into its adaptation to the environment and contribute to a better understanding of the impact of global climate change on the ecological environment.

## Methods

All procedures were carried out in accordance with the legal requirements and regulations of the Animal Experiment Ethics Committee of Northeast Forestry University (NO.20230271). All experimental procedures were approved by the Animal Care and Use Committee of Northeast Forestry University and were performed within the scope of legal requirements and regulations.

### Sample collection

**Fig. 2:**
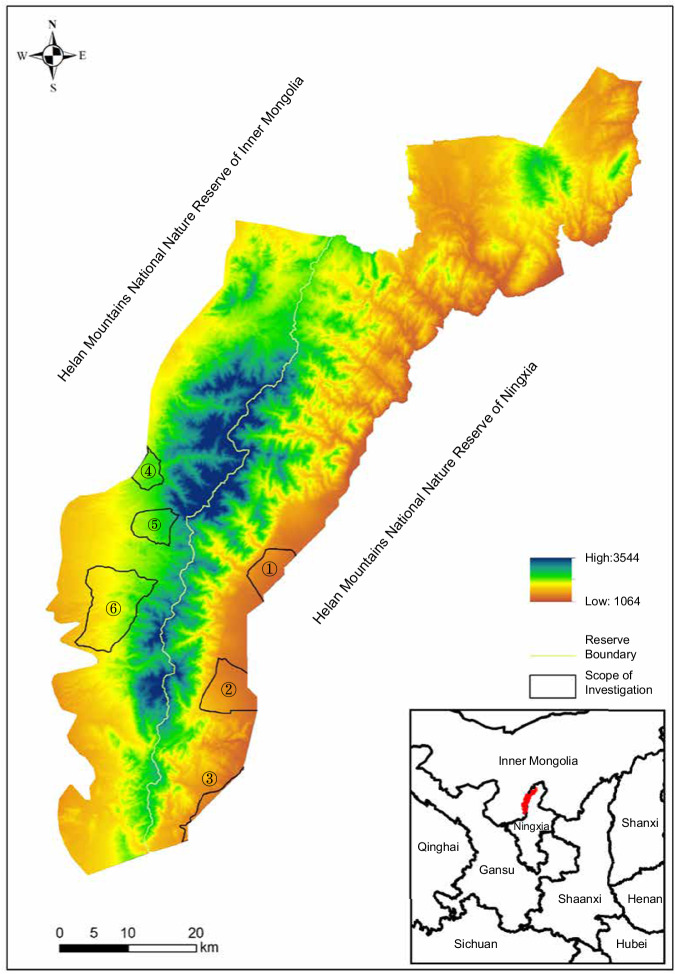
Helan Mountain capture areas for Alashan Ground Squirrels. The six regions are ① Helankou, ② Maliankou, ③ Yushugou, ④ Harau, ⑤ Fang Jiatian, ⑥ South Temple. The red-covered part in the lower right corner is Helan Mountains.

The sample collection work was led by the government to promote the prevention and control of grassland pests in 2022 (https://www.forestry.gov.cn/main/102/20220126/141650500484904.html). To explore the diversity of the squirrels, we deployed traps near the burrows in the six alluvial diluvial fan areas on both the eastern (105.34 E, 38.34 N) and western slope (105.83 E, 38.78 N) of Helan Mountains. The traps were carefully placed at 7:00 am, approximately four hours prior to capturing the squirrels. The procedure of live trapping refers to the operation of bank voles^15,16^. We analysed captures in western slope (n = 10) and eastern slope (n = 10). Fig. 2 shows the area where Alashan Ground Squirrels were captured.

For the collection of samples, we administered 5 mg/kg ethyl acetate (Xilong Scientific, CN) to anaesthetise the animals. Each specimen was assigned a unique identification number, and relevant data including weight, length, and location were recorded and shown in Table 1. Within five minutes of sacrifice, TRIzol reagent (Thermo Fisher Scientific, USA) was added to the tissues after blood collection at a ratio of 2:7. We harvested fresh heart, liver, cecum, and muscle tissues and immediately stored them in RNA extraction solution (Solarbio, CN). The contents of the cecum were collected and placed in an Eppendorf tube. Upon returning to the laboratory, all collected samples were stored at −80 °C before DNA and RNA extraction.

**Table 1:** Information on the individuals of Alashan Ground Squirrels.

Sample ID	Group	Weight (g)	Length (cm)	Location
S3	Eastern	162.31	22	Ningxia
S5	Eastern	142.13	24	Ningxia
S7	Eastern	139.93	21	Ningxia
S8	Eastern	218.57	25	Ningxia
S9	Eastern	179.77	26	Ningxia
S10	Eastern	158.05	25	Ningxia
S11	Eastern	66.63	20	Ningxia
S12	Eastern	86.89	22	Ningxia
S13	Eastern	148.31	26	Ningxia
S14	Eastern	202.16	27	Ningxia
XS1	Western	198.45	21	Inner Mongolia
XS2	Western	144.74	25	Inner Mongolia
XS3	Western	162.16	25	Inner Mongolia
XS5	Western	215.5	29	Inner Mongolia
XS6	Western	159.71	25	Inner Mongolia
XS7	Western	204.47	27	Inner Mongolia
XS8	Western	224.62	28	Inner Mongolia
XS9	Western	220.42	27.5	Inner Mongolia
XS10	Western	201.9	27	Inner Mongolia
XS11	Western	193.45	26	Inner Mongolia

### Sample preparation and RNA extraction

Approximately 50–100 mg of each tissue was taken and ground to powder in liquid nitrogen. The resulting powder was transferred to a centrifuge tube containing 1 mL of MJzol Reagent (Majorbio, CN) at a ratio greater than 10:1. The sample was thoroughly vortexed and centrifuged at 12,000 rpm for 5 minutes at 4 °C. The supernatant was then transferred to a new tube.

To isolate RNA, chloroform (Thermo Fisher Scientific, USA) was added to the supernatant at a ratio of 200 μL of chloroform per 1 mL of MJzol Reagent. The sample was vortexed for 15 seconds and allowed to stand at room temperature for 3 minutes. It was then centrifuged at 12,000 rpm for 15 minutes at 4 °C, resulting in three distinct layers: a rose-red organic layer at the bottom, a white intermediate layer, and a colourless aqueous layer at the top. The RNA was primarily present in the aqueous phase, which was transferred to a new tube.

Next, 10 μL of magnetic beads (Morck, CN) were added to the aqueous phase. The sample was vortexed for 15 seconds to disperse the beads and then allowed to stand at room temperature for 5 minutes. The tube was then placed on a magnetic stand for 3 minutes, after which the supernatant was discarded. The beads were washed by adding 500 μL of Wash Buffer (Majorbio, CN), vortexing for 15 seconds, and placing the tube on the magnetic stand for 3 minutes. Finally, 45 µL of the RNA solution was transferred to an RNase-Free tube for further analysis.

Total RNA was extracted using TRIzol® Reagent (Solarbio, CN) according to the manufacturer’s instructions (Thermo Fisher Scientific, CN). The purity and integrity of the extracted RNA were assessed by the 2100 Bioanalyser (Agilent, USA), and the concentration was measured using the NanoDrop ND-2000 (Thermo Fisher Scientific, USA). RNA samples of high quality were selected for library construction based on the following criteria: OD260/280 ratio of 1.8–2.2, OD260/230 ratio of ≥ 2.0, RNA integrity number (RIN) of ≥8.0, 28 S:18 S ratio of ≥ 1.0, and total RNA quantity of >1 μg.

### RNA library construction and sequencing

RNA purification, reverse transcription, library construction, and sequencing were performed at Majorbio Bio-pharm Biotechnology Co., Ltd. (Shanghai, CN) according to the manufacturer’s instructions (Illumina, USA). The Illumina TruSeqTM RNA preparation Kit (Illumina, USA) was used with 1 μg of total RNA to prepare the library. Briefly, poly(A) mRNA was selected using oligo(d)T beads (Invitrogen, USA) and fragmented using fragmentation buffer. The Illumina platform is designed to sequence short sequence fragments. The enriched mRNA, being a complete RNA sequence with an average length of several kb, needs to be randomly fragmented by adding 2% fragmentation buffer and selecting appropriate conditions to randomly fragment the mRNA into small fragments of about 300 bp. Using mRNA as a template, one-strand cDNA was reversely synthesised, followed by second-strand synthesis, using the SuperScript double-stranded cDNA synthesis kit (Invitrogen, UK) and random hexamer primers (Illumina, USA) to form a stable double-stranded duplex strand cDNA. Then, according to Illumina’s library construction protocol, the double-stranded cDNA structure has a sticky end. The End Repair Mix was added to make it blunt-ended, and then an A base is added to the 3′ end to connect the Y-shaped adapter. The adapter-ligated products were purified and fragment sorted, and the library was size-selected on a 2% Low Range Ultra Agarose gel to obtain a 300 bp cDNA target fragment, followed by 15 cycles of PCR amplification and purification with 2 U/μL Phusion DNA polymerase (NEB) to obtain the final library. The Qubit 4.0 (Thermo Fisher Scientific, USA) was used as a quantitative, proportional mixing machine. The cBot progressed through PCR expansion (T100 Thermal Cycler, USA) and generated clusters. Finally, the RNA-seq sequencing library was sequenced using the Illumina Novaseq 6000 platform with 2 × 150 bp read length.

### Sequence data processing and transcriptome *de novo* assembly

The data were analysed using the free online platform of Majorbio Cloud Platform (www.majorbio.com). We listed five types of original data for each sample, along with their original order number and progress order, in Table S1. To ensure the accuracy of downstream analysis, the raw sequencing data were first filtered to obtain high-quality sequencing data (clean data). The specific steps as follows: 1) Removal of adapter sequences in reads and deletion of reads lacking inserted fragments due to self-ligation of adapters and other reasons. 2) Trimming of low-quality (quality score <20) bases at the 3′ end of the sequence. If any remaining sequence still has a quality score <10, the entire sequence is deleted; otherwise, it is retained. 3) Removal of reads with an N-containing ratio exceeding 10%. 4) Exclusion of reads with adaptors and short reads (read length <20 bp). These reads were trimmed and quality-controlled on raw paired-end reads using fastp v0.19.5^17^ with default parameters. After obtaining high-quality RNA-seq data, we utilised Trinity v2.8.5^18^ for *de novo* assembly of sequencing reads, generating contigs and singletons.The first step, inchworm, involves decomposing reads, constructing a k-mer graph (K = 25) dictionary, selecting k-mer progressionsm and extending to form contigs. The second step, chrysalis, involves combining a series of contigs into a pruned isoform or a surface set with the same origin, each with its corresponding de Bruijn graph. The third step, butterfly, allows exporting each component of the de Bruijn graph, modifying the full length of the book, and obtaining the final result by tracing the original source of the sequence. The assembly results were assessed and optimised using TransRate v1.0.3^19^. Redundant and similar sequences were removed using CD-HIT v4.5.7. Transcriptome assembly integrity was assessed using BUSCO v3.0.2^20,21^.

**Fig. 3:**
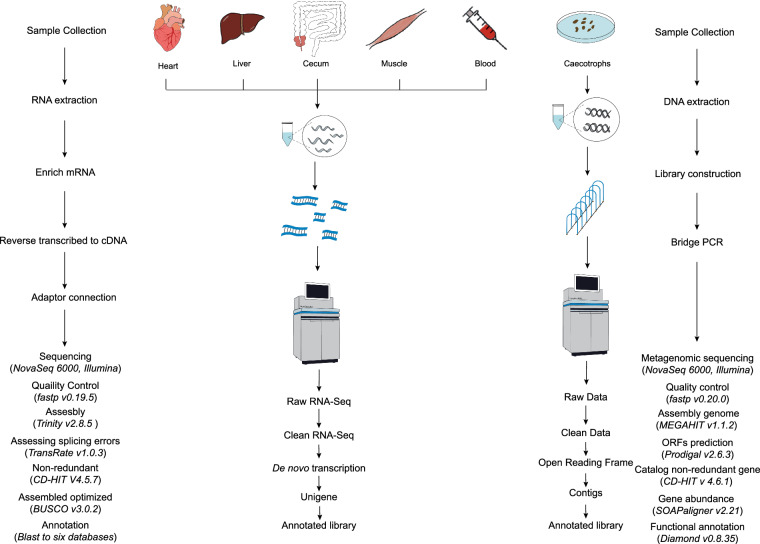
Complete workflow for transcriptomes and metagenomics.

The assembled transcripts were searched against several databases, including the NCBI protein non-redundant (NR) database, a manually annotated and reviewed protein sequence database (Swiss-Prot)^22^, Gene Ontology (GO)^23^, Pfam^24^, and Kyoto Encyclopedia of Genes and Genomes (KEGG)^25^. For analysis in NR, Clusters of Orthologous Genes (COG) and Swiss-Prot, DIAMOND v0.8.37.99 was utilised, applying a cut-off e-value of 1e-5. The Blast2GO v2.9.0^26^ facilitated the acquisition of GO annotations for unique assembled transcripts to describe biological processes, cellular components, and molecular functions. KOBAS v3.0^27^, with a cut-off e-value of 1e-5, was employed in the KEGG pathway analysis. Additionally, HMMER v3.2.1^28^ was used for Pfam with a cutoff e-value of 1e-5. Owing to the absence of a reference genome of Alashan Ground Squirrel, we executed a *de novo* transcriptome assembly pipeline. A schematic representation of all the working processes is provided in Fig. 3.

### Differential expression analysis and functional enrichment analysis

**Fig. 4:**
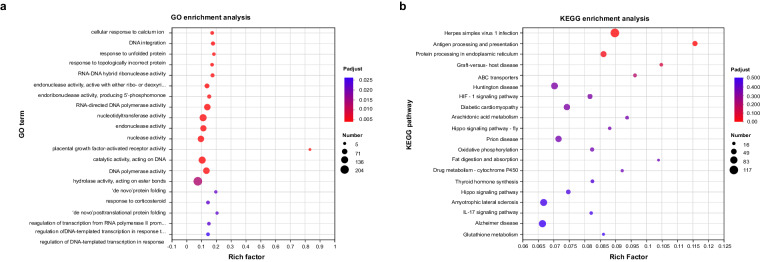
Functional enrichment analysis of the eastern and western slopes, (**a**) GO enrichment and (**b**) KEGG enrichment.

To identify differentially expressed genes (DEGs) between groups, we quantified the gene expression level of each gene using the transcripts per million reads (TPM) method. We used RSEM v1.3.1^29^ to estimate gene abundances and analysed the differential expression of genes between groups. Differential expression analysis was performed using DESeq2 v1.24.0^30^. Genes with |log2 (foldchange)| ≥ 1 and a p-adjust value ≤ 0.05 were considered DEGs. We then conducted functional enrichment analysis to identify the functions of DEGs against GO^23^ and KEGG^31^ databases using Goatools v0.6.5^32^ and a custem script developed by Majorbio (Shanghai, CN), respectively. A p-adjust < 0.05 was considered statistically significant. Enrichment analysis of GO and KEGG databases is showed in Fig. 4.

### Metagenomic DNA extraction and library preparation

Metagenomic DNA extraction was performed using the E.Z.N.A.® Soil DNA Kit (Omega Bio-tek, US) following the manufacturer’s instructions. The procedure involved adding 500 mg of magnetic beads and 0.5 g of SLX-Mlus Buffer to 2 mL of finely ground tissue in a tube, followed by vibration at 45 HZ for 250 seconds. Then, 100 μL of DS Buffer was added and mixed. The sample was incubated at 70 °C for 10 minutes and then at 95 °C for 2 minutes. After centrifugation at 13000 rpm at room temperature for 5 minutes, 800 μL of the supernatant was transferred to a fresh 2 mL tube, to which 270 μL of P2 Buffer and 100 μL of HTR Reagent were added. This was followed by incubation at −20 °C for 5 minutes and then centrifugation at 13000 rpm for another 5 minutes. The supernatant was then transferred to a fresh tube, and an equal volume of XP5 Buffer and 40 μL of magnetic beads were added. After mixing, the magnetic beads were used to adsorb and then remove the residual liquid. The tube was washed sequentially with 500 μL and then 600 μL of XP5 Buffer, followed by 600 μL of PHB. Finally, the tube was washed twice with 600 μL of SPW Wash Buffer. After the final centrifugation at 13000 rpm for 10 seconds, 100 μL of Elution Buffer was added, mixed, and left at room temperature for 5 minutes. The DNA was then transferred from the magnetic beads to a 1.5 mL tube using magnetic force.

The concentration and purity of the extracted DNA were measured using TBS-380 and NanoDrop2000, respectively. The DNA quality was assessed by running it on a 1% agarose gel at a voltage of 5 V/cm for 20 minutes. For library construction, the DNA was fragmented to an average size of approximately 400 bp using the Covaris M220 (Gene Company Limited, CN). The NEXTFLEX® Rapid DNA-seq kit (Bioo Scientific, USA) was used for the library construction. Adapters containing sequencing primer hybridisation sites were ligated to the blunt ends of the fragments. This process included adapter ligation, magnetic bead screening to remove self-ligated adapter fragments, enrichment of library templates through PCR amplification, and magnetic bead recovery of PCR products to obtain the final library.

### Bridge PCR and sequencing

Metagenomic sequencing was conducted using the Illumina NovaSeq 6000 sequencing platform at Majorbio Bio-pharm Biotechnology Co., Ltd. (Shanghai, CN) according to the manufacturer’s instructions (Illumina, USA). The process involves one end of the library molecule complementing the primer base, which, after a round of amplification, fixed the template information on the chip. The molecule’s other end, attached to the chip, randomly complements another nearby primer, forming a “bridge”. This PCR amplification resulted in DNA clusters. The DNA amplicons were then linearised into single strands. The addition of modified DNA polymerase and dNTPs with four fluorescent labels allows only one base to be synthesised in each cycle. A laser scans the reaction plate’s surface to read the nucleotide species polymerized in the first reaction round for each template sequence. The “fluorophore” and “termination group” are chemically cut to restore the 3′ end stickiness, enabling the second nucleotide’s polymerization. The sequencing of the template DNA fragment is determined by analysing the fluorescence signal statistics collected in each round.

### Sequence quality control and metagenome assembly

Adaptor sequences were removed, and low-quality reads (length <50 bp, quality value <20, or containing N bases) were filtered out using fastp v0.23.0^16^. Metagenomic sequencing data was assembled with MEGAHIT v1.1.2^33^, which utilises succinct de Bruijn graphs to resolve branching issues arising from strain differences. Contigs with a minimum length of 300 bp were kept as the final assembly, which was then used for gene prediction and annotation.

### Gene prediction, taxonomy

Open reading frames (ORFs) were predicted from each assembled contig using Prodigal v2.6.3^34^. The predicted ORFs, with a minimum length of 100 bp, were translated into amino acid sequences as potential indicators of protein-coding genes. A non-redundant gene catalogue was constructed using CD-HIT v4.6.1^20^, with a threshold of 90% sequence identity and 90% coverage. Clustering was performed based on the predicted coding fragments in the metagenomic sequencing assembly data. The longest gene in each cluster was selected as the representative sequence, reducing redundancy, and yielding the predicted gene set. High-quality reads were aligned to the non-redundant gene catalogues to calculate gene abundance, with a 95% identity threshold using SOAPaligner v2.21^35^.

### Functional annotation and quality control of annotation

**Fig. 5:**
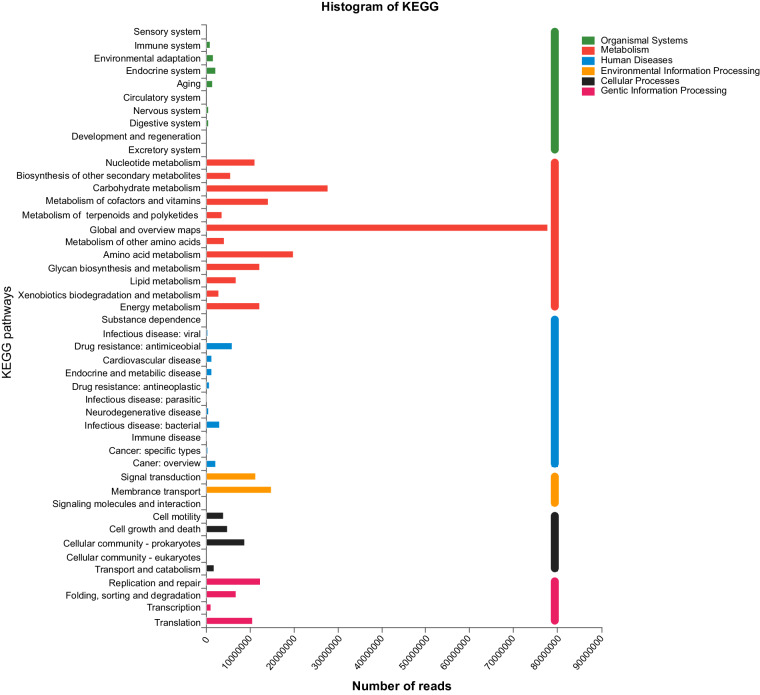
KEGG functional annotation of metagenomics.

Representative sequences from the non-redundant gene catalogue were aligned to the KEGG^25^ and COG^36,37^ databases using DIAMOND v0.8.35^38^ with an e-value cutoff of 1e-5 for taxonomic annotations. In KEGG functional annotation, the abundance of each functional category was calculated by summing the gene abundances corresponding to KO, Pathway, EC, and Module. The Carbohydrate-Active enZYmes (CAZy)^39^ database was used for comparison with the amino acid sequences of the non-redundant gene set, employing hmmscan with an expected e-value of 1e-5, to obtain carbohydrate-active enzyme annotation information. The abundance of carbohydrate-active enzymes was then calculated using the sum of the abundances of genes corresponding to these enzymes. The dominant bacterial groups identified were Firmicutes, Bacteroidetes, Verrucomicrobia, Urovircota, and Proteobacteria. An overview of KEGG annotations is shown in Fig. 5.

## Data Records

In this study, 20 Alashan Ground Squirrel individuals were used to produce 120 files, comprising different tissue RNA-seq samples and metagenomic samples. Specific details for each sample are provided in Tables 1, 2. Raw RNA-seq data were deposited in the NCBI BioProject^40^ https://identifiers.org/ncbi/bioproject:PRJNA935915. Raw metagenome data and corresponding assemblies were deposited in the NCBI BioProject^41^ https://identifiers.org/ncbi/bioproject:PRJNA932588.

**Table 2:** Summary of sample data information deposited in the SRA database.

Sample ID	Date collected	Tissue	Method	Sex	SRA accession
S3	2022-June-12	Heart, Liver, Cecum, Muscle, Blood	RNA-Seq	Male	SRR23557473, SRR23557472, SRR23557461, SRR23557415, SRR23557438
		Caecotrophs	MGS		SRR23368219
S5	2022-June-15	Heart, Liver, Cecum, Muscle, Blood	RNA-Seq	Male	SRR23557439, SRR23557428, SRR23557417, SRR23557406, SRR23557437
		Caecotrophs	MGS		SRR23368218
S7	2022-June-15	Heart, Liver, Cecum, Muscle, Blood	RNA-Seq	Male	SRR23557395, SRR23557384, SRR23557471, SRR23557470, SRR23557436
		Caecotrophs	MGS		SRR23368207
S8	2022-June-16	Heart, Liver, Cecum, Muscle, Blood	RNA-Seq	Male	SRR23557469, SRR23557468, SRR23557467, SRR23557466, SRR23557435
		Caecotrophs	MGS		SRR23368206
S9	2022-June-19	Heart, Liver, Cecum, Muscle, Blood	RNA-Seq	Male	SRR23557465, SRR23557464, SRR23557463, SRR23557462, SRR23557434
		Caecotrophs	MGS		SRR23368205
S10	2022-June-19	Heart, Liver, Cecum, Muscle, Blood	RNA-Seq	Male	SRR23557460, SRR23557459, SRR23557458, SRR23557457, SRR23557433
		Caecotrophs	MGS		SRR23368204
S11	2022-June-19	Heart, Liver, Cecum, Muscle, Blood	RNA-Seq	Unkown	SRR23557456, SRR23557455, SRR23557454, SRR23557453, SRR23557432
		Caecotrophs	MGS		SRR23368203
S12	2022-June-19	Heart, Liver, Cecum, Muscle, Blood	RNA-Seq	Unkown	SRR23557452, SRR23557451, SRR23557449, SRR23557448, SRR23557431
		Caecotrophs	MGS		SRR23368202
S13	2022-June-19	Heart, Liver, Cecum, Muscle, Blood	RNA-Seq	Male	SRR23557447, SRR23557446, SRR23557445, SRR23557444, SRR23557430
		Caecotrophs	MGS		SRR23368201
S14	2022-June-19	Heart, Liver, Cecum, Muscle, Blood	RNA-Seq	Male	SRR23557443, SRR23557442, SRR23557441, SRR23557440, SRR23557429
		Caecotrophs	MGS		SRR23368200
XS1	2022-June-26	Heart, Liver, Cecum, Muscle, Blood	RNA-Seq	Female	SRR23557427, SRR23557426, SRR23557425, SRR23557424, SRR23557383
		Caecotrophs	MGS		SRR23368217
XS2	2022-June-26	Heart, Liver, Cecum, Muscle, Blood	RNA-Seq	Male	SRR23557423, SRR23557422, SRR23557421, SRR23557420, SRR23557382
		Caecotrophs	MGS		SRR23368216
XS3	2022-June-27	Heart, Liver, Cecum, Muscle, Blood	RNA-Seq	Female	SRR23557419, SRR23557418, SRR23557416, SRR23557415, SRR23557381
		Caecotrophs	MGS		SRR23368215
XS5	2022-June-30	Heart, Liver, Cecum, Muscle, Blood	RNA-Seq	Male	SRR23557414, SRR23557413, SRR23557412, SRR23557411, SRR23557380
		Caecotrophs	MGS		SRR23368214
XS6	2022-June-27	Heart, Liver, Cecum, Muscle, Blood	RNA-Seq	Female	SRR23557410, SRR23557409, SRR23557408, SRR23557407, SRR23557379
		Caecotrophs	MGS		SRR23368213
XS7	2022-June-27	Heart, Liver, Cecum, Muscle, Blood	RNA-Seq	Male	SRR23557405, SRR23557404, SRR23557403, SRR23557402, SRR23557378
		Caecotrophs	MGS		SRR23368212
XS8	2022-June-27	Heart, Liver, Cecum, Muscle, Blood	RNA-Seq	Male	SRR23557401, SRR23557400, SRR23557399, SRR23557398, SRR23557377
		Caecotrophs	MGS		SRR23368211
XS9	2022-June-27	Heart, Liver, Cecum, Muscle, Blood	RNA-Seq	Male	SRR23557397, SRR23557396, SRR23557394, SRR23557393, SRR23557376
		Caecotrophs	MGS		SRR23368210
XS10	2022-June-27	Heart, Liver, Cecum, Muscle, Blood	RNA-Seq	Female	SRR23557392, SRR23557391, SRR23557390, SRR23557389, SRR23557375
		Caecotrophs	MGS		SRR23368209
XS11	2022-June-27	Heart, Liver, Cecum, Muscle, Blood	RNA-Seq	Male	SRR23557388, SRR23557387, SRR23557386, SRR23557385, SRR23557374
		Caecotrophs	MGS		SRR23368208

## Technical Validation

### Quality of the raw reads and assembly validation

**Fig. 6:**
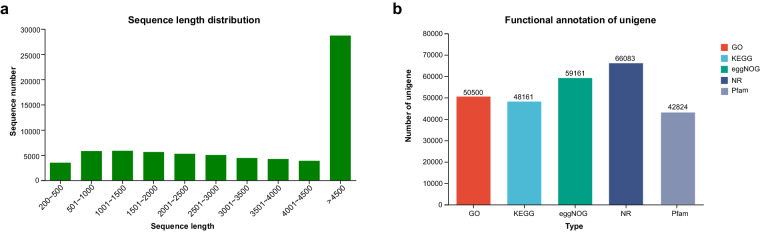
Sequence length distribution of unigenes and evaluation of functional annotation evaluation in different databases. (**a**) Sequence length distribution, (**b**) Compare all genes and transcripts obtained from transcriptome assembly with five major databases.

Over 700 million raw paired-end reads were obtained from 20 biological samples of Alashan Ground Squirrel. Subsequent trimming and filtering retained approximately 580 million high-quality paired-end reads for *de novo* assembly. The initial Trinity assembly produced 365,309 unigenes with an N50 of 4,992 bp. Transcriptome sequencing data for the five tissues is detailed in Table S1. Following assembly, we identified a total of 72,156 unigenes with an N50 of 6,703 bp and a GC content of 47.51%. The final assembled transcriptome BUSCO completeness score indicates that the assembly completeness is 98.4%. The optimised sequences were filtered for the initial assembly, which is summarised in Table 3. The length distribution of all assembled sequences and functional annotation statistics are depicted in Fig. 6, resulting in the assembly of 72,156 unigenes. The clean reads from each sample were mapped to the reference genome generated by the Trinity assembly, and the mapping statistics are reported in Table S2. This mapping forms the foundation for subsequent gene and transcript quantification for each sample.

**Table 3:** Evaluation of transcriptome assembly in Alashan Ground Squirrels.

Type	Unigene
Total number	72156
Total base	328300803
Largest length (bp)	67842
Smallest length (bp)	227
Average length (bp)	4549.88
N50 length (bp)	6703
E90 N50 length (bp)	6052
Fragment mapped percent (%)	80.969
GC percent (%)	47.51
TransRate score	0.26807
BUSCO score	C:98.4% [S:59.1%; D:39.3%]

For metagenomics analysis, fastp v0.23.0^17^ was used for data quality control, removing low-quality and N-containing reads from the original sequencing data. This process yielded high-quality sequences for further analysis, as shown in Table 4. The sequence demonstrating the best splicing effect was selected for ORF prediction. Genes with a nucleic acid length of 100 bp or greater were selected and translated into amino acid sequences, which are presented in Tables 5, 6.

**Table 4:** Clean reads statistics obtained from western and eastern slopes.

Samples ID	Clean reads	Clean base(bp)	Percent in raw reads (%)	Percent in raw bases (%)
S3	59712752	9005316270	98.52%	98.40%
S5	59479410	8968324336	97.64%	97.50%
S7	49237104	7420833301	98.21%	98.03%
S8	49198618	7419429897	98.11%	97.98%
S9	54076386	8133302780	98.13%	97.74%
S10	61077860	9213302925	98.49%	98.39%
S11	54258240	8183127430	98.32%	98.20%
S12	58610314	8841412820	98.30%	98.20%
S13	56138628	8468791902	98.28%	98.18%
S14	63057298	9511725801	98.65%	98.55%
XS1	53407122	8056172174	98.30%	98.19%
XS2	45775782	6903008003	98.22%	98.09%
XS3	56363424	8497800300	97.91%	97.76%
XS5	60185472	9073477953	98.01%	97.86%
XS6	56457206	8513672742	98.38%	98.25%
XS7	53601412	8081335678	98.28%	98.13%
XS8	51537056	7764378768	98.50%	98.27%
XS9	53349236	8019000372	98.23%	97.78%
XS10	55230542	8329274512	98.52%	98.39%
XS11	67865850	1.02E + 10	98.60%	98.40%

**Table 5:** Metagenome assembly statistics for each individual.

Sample ID	Contigs	Contigs bases (bp)	N50 (bp)	N90 (bp)	Max (bp)	Min (bp)
S3	564195	498802574	1089	379	270181	300
S5	187542	184212893	1349	388	325537	300
S7	523252	462025175	1092	380	237544	300
S8	542757	483754808	1111	380	311375	300
S9	482493	411124069	1051	365	211471	300
S10	538044	465220616	1037	372	305985	300
S11	586599	531467650	1153	382	304636	300
S12	276381	306006482	1851	409	313106	300
S13	591394	509157940	1025	375	213974	300
S14	559425	554387137	1394	398	673603	300
XS1	427167	404650506	1261	385	264424	300
XS2	385028	355760924	1218	381	258027	300
XS3	595998	535134923	1123	384	251464	300
XS5	439814	382877295	1046	377	343692	300
XS6	427739	398379714	1221	381	333016	300
XS7	586123	482065737	965	366	291077	300
XS8	387008	352732244	1231	371	248957	300
XS9	417589	349804725	1007	362	293622	300
XS10	376011	357519673	1280	385	248683	300
XS11	524589	444317122	1021	373	268105	300

**Table 6:** Gene prediction statistics for each individual. ORF = Open Reading Frame.

Sample	ORFs	Total Length (bp)	Average Length (bp)	Max (bp)	Min (bp)
S3	849425	437241855	514.75	22743	102
S5	298558	161111616	539.63	102585	102
S7	777466	402250353	517.39	26148	102
S8	819721	423971517	517.21	18435	102
S9	664382	342525429	515.55	47865	102
S10	800213	408507981	510.5	29397	102
S11	892962	464806770	520.52	16389	102
S12	470848	268707807	570.69	20535	102
S13	878409	444395196	505.91	29586	102
S14	892504	485796777	544.31	25116	102
xs1	665044	355032177	533.85	26376	102
xs2	590350	312018888	528.53	20769	102
xs3	902801	469418598	519.96	20769	102
xs5	650775	335522388	515.57	36609	102
xs6	659162	349580490	530.34	25785	102
xs7	846632	421747407	498.15	27951	102
xs8	584258	303786597	519.95	25803	102
xs9	578034	291068667	503.55	20259	102
xs10	586144	313055547	534.09	28122	102
xs11	761721	385038018	505.48	49983	102

### Quality control of annotation

The transcriptome was functionally annotated using DIAMOND^38^, KOBAS^27^, and Blast2GO^26^. We Compared all unigenes and expressed unigenes obtained from transcriptome assembly with major databases (NR, Swiss-prot, Pfam, GO and KEGG databases) to comprehensively gather functional information about unigenes. The annotations from each database are presented in Table 7.

**Table 7:** Transcriptome annotation.

	Expressedunigene number (percent)	All unigene number (percent)
GO	50500 (70.02%)	50526 (70.02%)
KEGG	48161 (66.77%)	48190 (66.79%)
eggNOG	59161 (82.02%)	59191 (82.03%)
NR	66083 (91.62%)	66113 (91.63%)
Swiss-Prot	59084 (81.92%)	59114 (81.93%)
Pfam	42824 (59.37%)	42846 (59.38%)
Total_anno	66736 (92.53%)	66766 (92.53%)
Total	72126 (100%)	72156 (100%)

For metagenomics, functional annotation was performed using DIAMOND v2.0.13^38^. We obtained species and abundance information for each taxonomic level in each sample. Comparison with the CAZy database provided functional annotation information of carbohydrate-active enzyme genes, where were then statistically analysed. Functional annotations of COG and CAZy are included in Table S3, S4 .

**Table 8:** Software for transcriptome analysis.

Software	Version	Source
fastx_toolkit	V0.0.14	http://hannonlab.cshl.edu/fastx_toolkit/
fastp	V0.19.5	https://github.com/OpenGene/fastp
TGICL	V 2.1	https://sourceforge.net/projects/tgicl/files/latest/download
Trinity	V2.8.5	https://github.com/trinityrnaseq/trinityrnaseq
SPAdes	V3.13.1	https://github.com/ablab/spades
BUSCO	V3.0.2	https://busco.ezlab.org/
cd-hit	V4.5.7	https://github.com/weizhongli/cdhit
hisat2	V2.1.0	http://ccb.jhu.edu/software/hisat2/index.shtml
samtools	V1.9	https://github.com/samtools/samtools.git
transrate	V1.0.3	http://hibberdlab.com/transrate/index.html
RSEM	V1.3.1	http://deweylab.biostat.wisc.edu/rsem/
kallisto	V0.46.0	https://pachterlab.github.io/kallisto/download
Salmon	V0.14.1	https://github.com/COMBINE-lab/salmon
bowtie2	V2.3.5.1	https://sourceforge.net/projects/bowtie-bio/files/bowtie2/2.3.5.1/
DESeq. 2	V1.24.0	http://bioconductor.org/packages/stats/bioc/DESeq. 2/
edgeR	V3.24.3	http://bioconductor.org/packages/stats/bioc/edgeR/
DEGSeq	V1.38.0	http://bioconductor.org/packages/stats/bioc/DEGSeq/
misa	v2.3.6	http://pgrc.ipk-gatersleben.de/misa/misa.html
TransDecoder	V5.5.0	http://transdecoder.github.io/
HMMER	V3.2.1	http://www.hmmer.org/download.html
bwa	V0.7.9a	https://sourceforge.net/projects/bio-bwa/files/
bcftools	V1.9	https://github.com/samtools/samtools.git
GATK	V3.8	https://software.broadinstitute.org/gatk/download/
BLAST+	V2.9.0	ftp://ftp.ncbi.nlm.nih.gov/blast/executables/blast+/2.9.0/
Diamond	V0.9.24	https://github.com/bbuchfink/diamond
WGCNA	V1.63	https://horvath.genetics.ucla.edu/html/CoexpressionNetwork/Rpackages/WGCNA/
STEM	V1.3.11	http://www.cs.cmu.edu/~jernst/stem/
maSigPro	V1.56.0	http://www.bioconductor.org/packages/release/bioc/html/maSigPro.html
GSEA	V3.0	http://software.broadinstitute.org/gsea/index.jsp
goatools	V 0.6.5	https://github.com/tanghaibao/goatools
MSigDB	V6.2	http://software.broadinstitute.org/gsea/downloads.jsp
STRING	V11.5	https://string-db.org/
AnimalTFDB	V3.0	http://bioinfo.life.hust.edu.cn/AnimalTFDB/
PlantTFDB	V 4.0	http://planttfdb.cbi.pku.edu.cn/
Pfam	V34.0	http://pfam.xfam.org/
KEGG	V2021.09	http://www.genome.jp/kegg/
eggNOG	V2020.06	http://eggnogdb.embl.de/#/app/home
Rfam	V14.6	http://rfam.janelia.org/
Swiss-Prot	V2021.06	https://www.expasy.org/resources/uniprotkb-swiss-prot
GO	V2021.0918	http://www.geneontology.org/
NR	V2021.10	https://www.ncbi.nlm.nih.gov/public/
PIR idmapping	V2021.06	ftp://ftp.pir.georgetown.edu/databases/idmapping/idmapping.tb.gz

**Table 9:** Software for metagenomic analysis.

Software	Version	Source
MEGAHIT	v1.1.2	https://github.com/voutcn/megahit
Prodigal	v2.6.3	https://github.com/hyattpd/Prodigal
CD-HIT	v4.6.1	http://weizhongli-lab.org/cd-hit/
SOAPaligner	soap2.21release	https://github.com/ShujiaHuang/SOAPaligner
CAZy	v8	http://bcb.unl.edu/dbCAN2/download/Databases/
ARDB	v1.1	http://ardb.cbcb.umd.edu/
CARD	v3.0.9	https://card.mcmaster.ca
VFDB	v20200703	http://www.mgc.ac.cn/VFs/main.htm
Diamond	v0.8.35	https://github.com/bbuchfink/diamond
HMMER	v3.1b2	http://hmmer.org/
Fastp	v0.23.0	https://github.com/OpenGene/fastp
Diamond	v2.0.13	https://github.com/bbuchfink/diamond
NR	nr_202109	https://ftp.ncbi.nlm.nih.gov/blast/db/FASTA/
COG	2020	http://eggnog5.embl.de/#/app/downloads
KEGG	202109	https://www.genome.jp/kegg

### Supplementary information

Supplementary Files

## Data Availability

Tables 8, 9 detail all the software and versions used in this study for transcriptomics and metagenomics, respectively. Unless specific parameter details are provided, the programs were utilised with their default parameters.

## References

[1] Wilson, D. E. & Reeder, D. M. Mammal Species of The World. A Taxonomic and Geographic Reference. (Smithsonian Institution Press, 1993).

[2] Kapustina SY, Brandler OV, Adiya Y (2015). Phylogeny of genus Spermophilus and position of Alashan ground squirrel (*Spermophilus alashanicus*, Buchner, 1888) on phylogenetic tree of Paleartic short-tailed ground squirrels. Mol Biol.

[3] Shar, S., Lkhagvasuren, D. & Smith, A. T. *Spermophilus alashanicus* (errata version published in 2017). *The IUCN Red List of Threatened Species* 2016: e.T20478A115158734.

[4] Chen B, Sun Y, An C, Huo L, Fan S (2014). Identification of *Spermophilus alaschanicus* and *Spermophilus dauricus* by DNA barcoding. Chinese Journal of Vector Biology and Control.

[5] Chen J, Yao Z, Shi R, Gao H, Liu Z (2022). Habitat suitability assessment of rodents on the west slope of the Helan Mountain based on MAXENT model. Acta Ecologica Sinica.

[6] Liu J (2010). Pattern and timing of late Cenozoic rapid exhumation and uplift of the Helan Mountain, China. Science China-Earth Sciences.

[7] Belkaid Y, Hand TW (2014). Role of the Microbiota in Immunity and Inflammation. Cell.

[8] Backhed F, Ley RE, Sonnenburg JL, Peterson DA, Gordon JI (2005). Host-bacterial mutualism in the human intestine. Science.

[9] Sharon G (2010). Commensal bacteria play a role in mating preference of Drosophila melanogaster. PNAS.

[10] Cryan JF, Dinan TG (2012). Mind-altering microorganisms: the impact of the gut microbiota on brain and behaviour. Nat Rev Neurosci.

[11] Ezenwa VO, Gerardo NM, Inouye DW, Medina M, Xavier JB (2012). Animal Behavior and the Microbiome. Science.

[12] Ley RE (2008). Evolution of mammals and their gut microbes. Science.

[13] Zhu L, Wu Q, Dai J, Zhang S, Wei F (2011). Evidence of cellulose metabolism by the giant panda gut microbiome. PNAS.

[14] Xiao L (2015). A catalog of the mouse gut metagenome. Nat. Biotechnol.

[15] Lavrinienko A, Tukalenko E, Mappes T, Watts PC (2018). Skin and gut microbiomes of a wild mammal respond to different environmental cues. Microbiome.

[16] Lavrinienko A (2018). Environmental radiation alters the gut microbiome of the bank vole Myodes glareolus. ISME J.

[17] Chen S, Zhou Y, Chen Y, Gu J (2018). Fastp: an ultra-fast all-in-one FASTQ preprocessor. Bioinformatics.

[18] Grabherr MG (2011). Full-length transcriptome assembly from RNA-Seq data without a reference genome. Nat Biotechnol.

[19] Smith-Unna R, Boursnell C, Patro R, Hibberd JM, Kelly S (2016). TransRate: reference-free quality assessment of de novo transcriptome assemblies. Genome Res.

[20] Fu L, Niu B, Zhu Z, Wu S, Li W (2012). CD-HIT: accelerated for clustering the next-generation sequencing data. Bioinformatics.

[21] Manni M, Berkeley MR, Seppey M, Simao FA, Zdobnov EM (2021). BUSCO Update: Novel and Streamlined Workflows along with Broader and Deeper Phylogenetic Coverage for Scoring of Eukaryotic, Prokaryotic, and Viral Genomes. Mol Biol Evol.

[22] Boeckmann B (2003). The SWISS-PROT protein knowledgebase and its supplement TrEMBL in 2003. Nucleic Acids Res..

[23] Blake JA (2015). Gene Ontology Consortium: going forward. Nucleic Acids Res.

[24] Bateman A (2004). The Pfam protein families database. Nucleic Acids Res.

[25] Kanehisa M, Furumichi M, Sato Y, Kawashima M, Ishiguro-Watanabe M (2022). KEGG for taxonomy-based analysis of pathways and genomes. Nucleic Acids Res.

[26] Conesa A (2005). Blast2GO: a universal tool for annotation, visualization and analysis in functional genomics research. Bioinformatics.

[27] Xie C (2011). KOBAS 2.0: a web server for annotation and identification of enriched pathways and diseases. Nucleic Acids Res.

[28] Marchin M, Kelly PT, Fang J (2005). Tracker: continuous HMMER and BLAST searching. Bioinformatics..

[29] Li B, Dewey CN (2011). RSEM: accurate transcript quantification from RNA-Seq data with or without a reference genome. Bmc Bioinformatics.

[30] Love MI, Huber W, Anders S (2014). Moderated estimation of fold change and dispersion for RNA-seq data with DESeq. 2. Genome Biol.

[31] Kanehisa M, Sato Y, Morishima K (2016). BlastKOALA and GhostKOALA: KEGG Tools for Functional Characterization of Genome and Metagenome Sequences. J Mol Biol.

[32] Klopfenstein DV (2018). GOATOOLS: A Python library for Gene Ontology analyses. Sci Rep.

[33] Li DLC, Luo R, Sadakane K, Lam TW (2015). MEGAHIT: an ultra-fast single-node solution for large and complex metagenomics assembly via succinct de Bruijn graph. Bioinformatics.

[34] Hyatt D (2010). Prodigal: prokaryotic gene recognition and translation initiation site identification. Bmc Bioinformatics.

[35] Li R, Li Y, Kristiansen K, Wang J (2008). SOAP: short oligonucleotide alignment program. Bioinformatics.

[36] Galperin MY (2021). COG database update: focus on microbial diversity, model organisms, and widespread pathogens. Nucleic Acids Res.

[37] Galperin MY, Makarova KS, Wolf YI, Koonin EV (2015). Expanded microbial genome coverage and improved protein family annotation in the COG database. Nucleic Acids Res.

[38] Buchfink B, Xie C, Huson DH (2015). Fast and sensitive protein alignment using DIAMOND. Nat Methods.

[39] Levasseur A, Drula E, Lombard V, Coutinho PM, Henrissat B (2013). Expansion of the enzymatic repertoire of the CAZy database to integrate auxiliary redox enzymes. Biotechnol Biofuels.

[40] (2023). BioProject.

[41] (2024). NCBI BioProject.

