# Supplementary material for: Exploring Alashan Ground Squirrel (Spermophilus alashanicus) Diversity: Metagenomic and Transcriptomic Datasets from the Helan Mountains
Source: Sci Data. 2024 May 21;11:517. doi: 10.1038/s41597-024-03183-6 (PMC11109200; doi:10.1038/s41597-024-03183-6)
Supplement: Supplementary file 1 — Supplementary Files [file 41597_2024_3183_MOESM1_ESM.pdf]

Exploring Alashan Ground Squirrel (*Spermophilus alashanicus*) Diversity: Metagenomic and Transcriptomic Datasets from the Helan Mountains

Yao Zhao[1], Siwei Deng[2], Zhirong Zhang[1], Junda Chen[1], Liwei Teng[1,3]& Zhensheng Liu[1,3]

1 College of Wildlife and Protected Area, Northeast Forestry University, Harbin 150040, China

2 OxTium Technology Co., Ltd, Shenzhen 518000, China

3 Key Laboratory of Conservation Biology, National Forestry and Grassland Administration, Harbin 150090, China

***Corresponding Authors**

Zhensheng Liu

Email: zhenshengliu@163.com

Liwei Teng

E-mail: tenglw1975@163.com

Supplementary information

Supplementary Table 1. Transcriptome sequencing data for the five tissues, including heart, liver, cecum, blood, and muscle.

Supplementary Table 2. The statistics of clean reads, mapped reads, and mapping rate for each transcriptome sequencing sample.

Supplementary Table 3. Functional annotations of metagenomics sequencing data against the database of Clusters of Orthologous Genes (COG).

Supplementary Table 4. Functional annotations of metagenomics sequencing data against the database of Carbohydrate-Active enZYmes (CAZy).

Supplementary Table 1. Transcriptome sequencing data for the five tissues, including heart, liver, cecum, blood, and muscle.

Sample	Raw reads	Clean reads	Error rate(%)	Q20(%)	Q30(%)	GC content(%)	Dup_pair	rRNA ratio(%)
xs1_Heart	58293326	57804018	0.026	97.7	93.29	50.5	0.23041751	0.389
xs1_Liver	55070528	54544168	0.0254	97.95	93.88	49.17	0.32560717	0.492
xs1_Cecum	47600506	47058382	0.0259	97.74	93.3	47.12	0.30658219	0.853
xs1_Muscle	54889736	54350778	0.0253	97.96	93.92	50.26	0.29622634	0.745
xs2_Heart	52465436	51845722	0.0257	97.83	93.49	47.83	0.33409221	0.761
xs2_Liver	53875422	53340942	0.0249	98.13	94.28	49.59	0.3154098	0.512
xs2_Cecum	53894926	53292334	0.0256	97.82	93.59	49.28	0.26739381	0.776
xs2_Muscle	44910750	44624458	0.0256	97.84	93.55	49.61	0.29422354	0.756
xs3_Heart	59927252	59581344	0.0253	97.98	93.88	47.28	0.36307529	0.958
xs3_Liver	52476600	51949768	0.0259	97.72	93.33	48.87	0.30354157	0.381
xs3_Cecum	56062060	55573922	0.0255	97.89	93.75	48.65	0.29311147	0.584
xs3_Muscle	44263908	43706382	0.0257	97.83	93.49	44.62	0.40391694	2.142
xs5_Heart	46557684	46233438	0.0259	97.74	93.3	46.97	0.33161081	0.843
xs5_Liver	56718024	56064012	0.025	98.09	94.23	49.25	0.33712835	0.499
xs5_Cecum	50100322	49588602	0.0247	98.2	94.49	54.55	0.52203952	0.86
xs5_Muscle	56744588	56005212	0.0257	97.78	93.53	52.69	0.31240871	0.719
xs6_Heart	56708406	56306574	0.0254	97.95	93.83	47.89	0.32852473	0.51
xs6_Liver	49433108	49109916	0.0254	97.92	93.78	48.81	0.28666093	0.414
xs6_Cecum	47047640	46743574	0.0253	97.97	93.93	49.34	0.24386111	0.426
xs6_Muscle	57706824	57126216	0.0254	97.92	93.82	50.99	0.30456794	0.593
xs7_Heart	55756304	55399748	0.0257	97.83	93.57	46.89	0.33025002	0.655
xs7_Liver	52519140	52137002	0.0252	98.01	94.02	50.4	0.31045471	0.547
xs7_Cecum	66717890	65978024	0.0258	97.81	93.55	49.26	0.26392358	0.409
xs7_Muscle	56836560	55839658	0.0255	97.91	93.79	53.83	0.29108663	0.604
xs8_Heart	64139408	63650844	0.0257	97.86	93.58	47.15	0.37431604	0.675
xs8_Liver	62232786	61543500	0.0253	97.99	93.99	49.37	0.34082241	0.396
xs8_Cecum	51120070	50629732	0.0255	97.88	93.77	50.67	0.2391496	0.86
xs8_Muscle	56231574	55612432	0.0255	97.85	93.71	52.44	0.31242719	0.561
xs9_Heart	51808350	51445290	0.0254	97.95	93.8	47.09	0.35044157	0.54
xs9_Liver	60414710	59807958	0.0254	97.93	93.82	49.29	0.31351774	0.433
xs9_Cecum	56931206	56508216	0.0255	97.89	93.75	49.6	0.26055308	0.524

xs9_Muscle	51562650	51011374	0.0255	97.88	93.78	53.26	0.31007763	1.167
xs10_Heart	54693264	54252878	0.0254	97.95	93.84	47.14	0.33364962	0.895
xs10_Liver	55044702	54585550	0.0251	98.09	94.15	48.94	0.30851476	0.417
xs10_Cecum	55018174	54475636	0.0254	97.92	93.82	48.85	0.26722122	0.413
xs10_Muscle	74211812	73438470	0.0255	97.91	93.8	50.96	0.32516168	0.705
xs11_Heart	60100478	59717866	0.0254	97.96	93.82	47.43	0.34385984	0.674
xs11_Liver	47163840	46721844	0.0251	98.07	94.15	48.82	0.31755348	0.384
xs11_Cecum	47912814	47605006	0.0256	97.83	93.6	48.05	0.24023957	0.408
xs11_Muscle	72847030	72019038	0.0254	97.91	93.82	50.61	0.3384244	0.792
xs1_blood	56449126	55772358	0.0251	98.03	94.11	53.02	0.53251856	0.785
xs2_blood	56114354	55690902	0.0251	98.03	94.05	54.52	0.69668669	0.859
xs3_blood	52530952	51944454	0.0249	98.13	94.27	53.7	0.68733832	0.699
xs5_blood	54560734	53766522	0.0251	98.05	94.07	53.84	0.66989991	0.969
xs6_blood	51657972	50962450	0.0251	98.06	94.12	54.31	0.67879197	0.605
xs7_blood	58545714	57576320	0.0249	98.11	94.29	54.46	0.69538428	0.937
xs8_blood	45348360	44503018	0.0256	97.86	93.63	52.67	0.56503984	1.452
xs9_blood	54727876	53733948	0.0253	97.95	93.9	53.97	0.62104899	0.93
xs10_blood	51619930	50730120	0.025	98.09	94.21	53.14	0.59697411	0.722
xs11_blood	45041648	44251670	0.025	98.09	94.16	52.6	0.64638993	1.25
S3_Heart	64649186	63159126	0.0259	97.68	93.41	52.43	0.26362189	0.567
S3_Liver	51037156	49543734	0.0255	97.8	93.73	54.27	0.27700944	1.868
S3_Cecum	46880296	45887772	0.0263	97.55	93.03	50.55	0.19742663	1.1
S3_Muscle	54480712	53369350	0.0261	97.59	93.19	54.47	0.29747921	1.471
S5_Heart	50953154	50088500	0.0258	97.75	93.41	49.32	0.27381463	1.236
S5_Liver	54509982	53367162	0.0254	97.89	93.84	50.72	0.29379471	0.934
S5_Cecum	59913600	58799092	0.026	97.63	93.23	51.17	0.22114154	1.03
S5_Muscle	57063086	55732948	0.0257	97.73	93.54	54.31	0.28036841	1.092
S7_Heart	66935284	65848406	0.0259	97.72	93.38	48.46	0.32354369	1.08
S7_Liver	55863854	54882482	0.0252	97.96	94	49.76	0.29829588	0.899
S7_Cecum	56965342	55425302	0.0262	97.54	93.1	53.21	0.22032216	1.155
S7_Muscle	66362362	64643132	0.0258	97.72	93.52	55.16	0.3105581	1.06
S8_Heart	62404926	61052778	0.026	97.61	93.26	53.59	0.28665238	1.595
S8_Liver	59458260	58040176	0.0256	97.77	93.65	51.77	0.28490286	0.949

S8_Cecum	56633532	55090418	0.0261	97.6	93.21	50.71	0.22708573	0.997
S8_Muscle	61629388	60216468	0.0256	97.77	93.64	54.81	0.31876068	1.099
S9_Heart	56513248	55166680	0.0255	97.86	93.8	52.73	0.29853894	2.006
S9_Liver	62726270	61340970	0.0255	97.84	93.77	53.53	0.30912941	1.303
S9_Cecum	51387252	50252338	0.0256	97.78	93.64	53.77	0.2242304	1.586
S9_Muscle	52822538	51680148	0.0254	97.89	93.91	53.8	0.29309595	0.981
S10_Heart	52046738	51097020	0.0258	97.75	93.47	50.13	0.30096193	1.095
S10_Liver	48862302	47713896	0.0257	97.76	93.59	51.16	0.27044775	1.262
S10_Cecum	55070704	53574098	0.0263	97.52	93.03	52.84	0.20913435	1.871
S10_Muscle	53422928	52129310	0.026	97.62	93.33	55.46	0.28142843	1.457
S11_Heart	56898694	55683370	0.0261	97.58	93.18	53.41	0.2140021	1.464
S11_Liver	54038268	52900202	0.0259	97.67	93.37	52.02	0.2595327	1.182
S11_Cecum	54490720	53333046	0.0258	97.75	93.53	50.8	0.23256872	0.993
S11_Muscle	54351922	52870632	0.0259	97.63	93.35	55.52	0.2995831	4.026
S12_Heart	44862968	44321094	0.0254	97.88	93.85	53.77	0.26247854	0.877
S12_Liver	61093506	60559172	0.0253	97.94	93.92	50.12	0.28853898	0.331
S12_Cecum	56091696	55432976	0.0257	97.75	93.53	52.97	0.21862261	0.756
S12_Muscle	54168122	53573804	0.0255	97.86	93.72	53.99	0.31367913	0.873
S13_Heart	48127328	47114712	0.0256	97.82	93.63	49.08	0.31604578	0.92
S13_Liver	52237568	51776198	0.0259	97.75	93.34	50.89	0.28871514	0.51
S13_Cecum	58578922	57922162	0.0253	97.96	94.01	50.28	0.26451502	0.579
S13_Muscle	58973178	58500252	0.0253	97.95	93.92	50.51	0.30326023	0.642
S14_Heart	47145194	46601658	0.0256	97.8	93.65	53.36	0.26839753	0.852
S14_Liver	49029284	48479304	0.0258	97.74	93.4	52.6	0.2924004	0.747
S14_Cecum	48701710	48135792	0.0262	97.59	93.1	52.7	0.23553696	0.734
S14_Muscle	47419594	46972948	0.0256	97.85	93.69	51.49	0.29838246	0.824
S3_blood	44043054	43496310	0.0252	97.99	93.99	53	0.56979049	0.725
S5_blood	53316626	52655558	0.0252	98.01	94.01	53.9	0.64447487	1.146
S7_blood	48678200	48092966	0.025	98.12	94.22	53.98	0.6738993	1.528
S8_blood	52405942	51758104	0.025	98.07	94.15	53.72	0.60879548	1.159
S9_blood	42827862	42331850	0.025	98.09	94.18	53.95	0.6533482	1.047
S10_blood	59744692	59056438	0.0251	98.05	94.11	54.02	0.67735629	0.995
S11_blood	59282740	58573030	0.0246	98.24	94.55	54.74	0.70025215	1.503

S12_blood	49477102	48901262	0.0248	98.16	94.34	54.03	0.69610343	1.235
S13_blood	45540142	44882332	0.0256	97.81	93.6	53.84	0.58706914	1.86
S14_blood	48281024	47817730	0.025	98.1	94.21	53.83	0.65255009	1.58

Supplementary Table 2. The statistics of clean reads, mapped reads, and mapping rate for each transcriptome sequencing sample.

Sample	Clean reads	Mapped reads	Mapped rate
xs1_Heart	28902009	24799890	85.81%
xs1_Liver	27272084	24843557	91.10%
xs1_Cecum	23529191	20958810	89.08%
xs1_Muscle	27175389	24104610	88.70%
xs2_Heart	25922861	23242923	89.66%
xs2_Liver	26670471	24144593	90.53%
xs2_Cecum	26646167	23413572	87.87%
xs2_Muscle	22312229	19266150	86.35%
xs3_Heart	29790672	26110008	87.64%
xs3_Liver	25974884	23471541	90.36%
xs3_Cecum	27786961	24517312	88.23%
xs3_Muscle	21853191	19880802	90.97%
xs5_Heart	23116719	20207106	87.41%
xs5_Liver	28032006	25629298	91.43%
xs5_Cecum	24794301	23048274	92.96%
xs5_Muscle	28002606	25155500	89.83%
xs6_Heart	28153287	24873783	88.35%
xs6_Liver	24554958	21797117	88.77%
xs6_Cecum	23371787	20444260	87.47%
xs6_Muscle	28563108	25417851	88.99%
xs7_Heart	27699874	24467109	88.33%
xs7_Liver	26068501	23295877	89.36%
xs7_Cecum	32989012	29314835	88.86%
xs7_Muscle	27919829	25150861	90.08%
xs8_Heart	31825422	28175889	88.53%
xs8_Liver	30771750	28092399	91.29%
xs8_Cecum	25314866	22221321	87.78%
xs8_Muscle	27806216	24894243	89.53%
xs9_Heart	25722645	22705600	88.27%
xs9_Liver	29903979	26931749	90.06%

xs9_Cecum	28254108	24937507	88.26%
xs9_Muscle	25505687	22765641	89.26%
xs10_Heart	27126439	23982621	88.41%
xs10_Liver	27292775	24495145	89.75%
xs10_Cecum	27237818	24024636	88.20%
xs10_Muscle	36719235	32655958	88.93%
xs11_Heart	29858933	26245465	87.90%
xs11_Liver	23360922	21321028	91.27%
xs11_Cecum	23802503	20071829	84.33%
xs11_Muscle	36009519	32114547	89.18%
xs1_Blood	27886179	19129863	68.60%
xs2_Blood	27845451	16893950	60.67%
xs3_Blood	25972227	14641811	56.37%
xs5_Blood	26883261	14699685	54.68%
xs6_Blood	25481225	15378191	60.35%
xs7_Blood	28788160	19098525	66.34%
xs8_Blood	22251509	14107484	63.40%
xs9_Blood	26866974	17806252	66.28%
xs10_Blood	25365060	15141804	59.70%
xs11_Blood	22125835	12346795	55.80%
S3_Heart	31579563	27870909	88.26%
S3_Liver	24771867	22295082	90.00%
S3_Cecum	22943886	19851280	86.52%
S3_Muscle	26684675	23864318	89.43%
S5_Heart	25044250	22047729	88.04%
S5_Liver	26683581	24030061	90.06%
S5_Cecum	29399546	25769162	87.65%
S5_Muscle	27866474	24864868	89.23%
S7_Heart	32924203	28683671	87.12%
S7_Liver	27441241	24648963	89.82%
S7_Cecum	27712651	24062391	86.83%
S7_Muscle	32321566	28614185	88.53%

S8_Heart	30526389	26798189	87.79%
S8_Liver	29020088	26092412	89.91%
S8_Cecum	27545209	23921494	86.84%
S8_Muscle	30108234	26821542	89.08%
S9_Heart	27583340	24673269	89.45%
S9_Liver	30670485	27606177	90.01%
S9_Cecum	25126169	22070428	87.84%
S9_Muscle	25840074	23060267	89.24%
S10_Heart	25548510	22459777	87.91%
S10_Liver	23856948	21468061	89.99%
S10_Cecum	26787049	23334814	87.11%
S10_Muscle	26064655	23382699	89.71%
S11_Heart	27841685	24219393	86.99%
S11_Liver	26450101	23516045	88.91%
S11_Cecum	26666523	23311231	87.42%
S11_Muscle	26435316	23267651	88.02%
S12_Heart	22160547	19647670	88.66%
S12_Liver	30279586	26836863	88.63%
S12_Cecum	27716488	24263206	87.54%
S12_Muscle	26786902	23821951	88.93%
S13_Heart	23557356	21258170	90.24%
S13_Liver	25888099	23254049	89.83%
S13_Cecum	28961081	25792006	89.06%
S13_Muscle	29250126	25769863	88.10%
S14_Heart	23300829	20550120	88.19%
S14_Liver	24239652	21907356	90.38%
S14_Cecum	24067896	21263109	88.35%
S14_Muscle	23486474	20982209	89.34%
S3_Blood	21748155	13447032	61.83%
S5_Blood	26327779	15750681	59.83%
S7_Blood	24046483	15632845	65.01%
S8_Blood	25879052	16175073	62.50%

S9_Blood	21165925	12329408	58.25%
S10_Blood	29528219	16945314	57.39%
S11_Blood	29286515	20388431	69.62%
S12_Blood	24450631	14027759	57.37%
S13_Blood	22441166	17627460	78.55%
S14_Blood	23908865	15651737	65.46%

Supplementary Table 3. Functional annotations of metagenomics sequencing data against the database of Clusters of Orthologous Genes (COG).

COG	COG_Description	Function	Western Slopes	Estern Slopes	Total
COG3717	5-keto 4-deoxyuronate isomerase	G	3457	3322	6779
COG1814	Predicted Fe2+/Mn2+ transporter, VIT1/CCC1 family	P	190	346	536
COG1920	2-phospho-L-lactate guanylyltransferase, coenzyme F420 biosynthesis enzyme, CobY/MobA/RfbA family	H	3	76	79
COG2842	Bacteriophage DNA transposition protein, AAA+ family ATPase	X	1607	3394	5000
COG2364	Uncharacterized membrane protein YczE	S	2638	3830	6468
COG2857	Cytochrome c1	C	29	5	34
COG5645	Uncharacterized conserved protein YceK	S	0	1	1
COG0380	Trehalose-6-phosphate synthase, GT20 family	G	33	287	320
COG2315	Predicted DNA-binding protein with double-wing structural motif, MmcQ/YjbR family	K	7615	8528	16143
COG0101	tRNA U38,U39,U40 pseudouridine synthase TruA	J	10678	12163	22841
COG1633	Rubrerythrin, includes spore coat protein YhjR	P	656	946	1602
COG4468	Galactose-1-phosphate uridylyltransferase	G	4992	5820	10812
COG3924	Uncharacterized membrane protein YhdT	S	3	3	6
COG1453	Predicted oxidoreductase of the aldo/keto reductase family	R	17309	19286	36595
COG3316	Transposase (or an inactivated derivative), DDE domain	X	1647	1694	3341
COG2852	Very-short-patch-repair endonuclease	L	1211	1258	2469
COG0826	23S rRNA C2501 and tRNA U34 5'-hydroxylation protein RlhA/YrrN/YrrO, U32 peptidase family	J	28910	32302	61212

COG5321	Uncharacterized conserved protein	S	28	59	87
COG1863	Multisubunit Na+/H+ antiporter, MnhE subunit	P	1107	1123	2230
COG1269	Archaeal/vacuolar-type H+-ATPase subunit I/STV1	C	6793	7741	14534
COG4922	Predicted SnoaL-like aldol condensation-catalyzing enzyme	R	325	173	498
COG4936	Ligand-binding sensor domain	T	687	691	1378
COG1144	Pyruvate:ferredoxin oxidoreductase or related 2-oxoacid:ferredoxin oxidoreductase, delta subunit	C	976	1889	2866
COG0643	Chemotaxis protein histidine kinase CheA	T	6742	5999	12741
COG5482	Uncharacterized conserved protein, DUF2161 domain	S	52	56	108
COG4289	Uncharacterized conserved protein, DUF2264 domain	S	2161	3521	5682
COG1007	NADH:ubiquinone oxidoreductase subunit 2 (chain N)	C	1807	2133	3940
COG0035	Uracil phosphoribosyltransferase	F	5881	6047	11928
COG3542	Predicted sugar epimerase, cupin	R	114	139	253
COG0107	Imidazole glycerol phosphate synthase subunit HisF	E	5477	7294	12771
COG2895	Sulfate adenylyltransferase subunit 1, EFTu-like GTPase family	P	245	440	685
COG1801	Sugar isomerase-related protein YecE, UPF0759/DUF72 family	R	61	96	158
COG1510	DNA-binding transcriptional regulator GbsR, MarR family	K	4565	4840	9406
COG0015	Adenylosuccinate lyase	F	9402	11383	20785

COG2117	Predicted subunit of tRNA(5-methylaminomethyl-2-thiouridylate) methyltransferase, contains the PP-loop ATPase domain	J	4	2	6
COG3793	Tellurite resistance protein TerB	P	145	5	150
COG1755	Uncharacterized conserved protein YpbQ, isoprenylcysteine carboxyl methyltransferase (ICMT) family	S	1	1	2
COG0102	Ribosomal protein L13	J	4758	5237	9995
COG2332	Cytochrome c biogenesis protein CcmE	C;O	67	184	251
COG0143	Methionyl-tRNA synthetase	J	3743	3810	7553
COG4277	Predicted DNA modification or repair protein, contains radical SAM and helix-hairpin-helix domains	R	3831	4923	8754
COG5010	Flp pilus assembly protein TadD, contains TPR repeats	U;W	9751	9773	19524
COG0810	Periplasmic protein TonB, links inner and outer membranes	M	7825	7905	15730
COG3132	Uncharacterized conserved protein YceH, UPF0502 family	S	0	1	1
COG2841	Uncharacterized conserved protein YdcH, DUF465 family	S	1	0	1
COG4750	CTP:phosphocholine cytidylyltransferase LicC	M;I	2301	2868	5170
COG2810	Predicted type IV restriction endonuclease	V	218	284	502
COG3913	Regulator of T6SS expression TagF, TagF/ImpM/SciT family (unrelated to teichoic acid polymerase TagF of B.subtilis, COG1887)	T;U	1	1	2
COG4220	Phage DNA packaging protein, Nu1 subunit of terminase	X	1170	711	1881
COG3510	Cephalosporin hydroxylase	V	404	439	842

COG3469	Chitinase	G	297	229	527
COG4643	Uncharacterized domain associated with phage/plasmid primase	X	864	954	1818
COG5449	Uncharacterized conserved protein, DUF2163 domain	S	2	6	7
COG4239	ABC-type microcin C transport system, permease component YejE	Q	333	780	1113
COG1429	Cobalamin biosynthesis protein CobN, Mg-chelatase	H	1381	2403	3784
COG1314	Protein translocase subunit SecG	U	2896	3189	6085
COG1155	Archaeal/vacuolar-type H+-ATPase catalytic subunit A/Vma1	C	5884	6977	12861
COG5823	Stage III sporulation protein SpoIIIAB, component of the engulfment complex	D	1430	1948	3378
COG4314	Nitrous oxide reductase accessory protein NosL	P	1	262	263
COG5866	Sporulation-specific protease YabG	D;O	794	1121	1915
COG0172	Seryl-tRNA synthetase	J	10360	12278	22638
COG1115	Na+/alanine symporter	E	12193	15369	27563
COG4585	Signal transduction histidine kinase ComP	T	6440	6898	13338
COG4773	Outer membrane receptor for ferric coprogen and ferric-rhodotorulic acid	P	54	104	158
COG4145	Na+/panthothenate symporter	H	66	137	203
COG5531	DNA-binding SWIB/MDM2 domain	B	0	2	2
COG3802	Uncharacterized conserved protein GguC, FAA hydrolase family	S	1	1	2
COG3710	DNA-binding winged helix-turn-helix (wHTH) domain	K	14	102	116
COG3194	Ureidoglycolate hydrolase (allantoin degradation)	F	122	237	360
COG2366	Acyl-homoserine lactone (AHL) acylase PvdQ	Q	20	18	39

COG1479	DNAse/DNA nickase specific for phosphorothioated or glycosylated phage DNA, GmrSD/DndB/SspE family, contains DUF262 and HNH nuclease domains	V	11873	13390	25263
COG3444	Phosphotransferase system, mannose/fructose/N-acetylgalactosamine-specific component IIB	G	398	1145	1543
COG4747	ACT domain-containing protein	R	1103	1561	2664
COG1509	L-lysine 2,3-aminomutase (EF-P beta-lysylation pathway)	E	1048	1625	2673
COG3591	V8-like Glu-specific endopeptidase	O	743	1085	1828
COG4961	Flp pilus assembly protein TadG, includes N-terminal TadE domain	U;W	771	829	1600
COG4955	Uncharacterized conserved protein YpbB, contains C-terminal HTH domain	S	29	77	106
COG0433	Archaeal DNA helicase HerA or a related bacterial ATPase, contains HAS-barrel and ATPase domains	L	11892	11348	23240
COG0807	GTP cyclohydrolase II	H	19	15	34
COG5663	Uncharacterized conserved protein YqfW, HAD superfamily	R	549	732	1281
COG1808	Uncharacterized membrane protein AF0785, contains DUF389 domain	S	3118	2410	5528
COG2370	Hydrogenase/urease accessory protein	O	0	1	1
COG3861	Stress response protein YsnF (function unknown)	S	0	62	62
COG5598	Trimethylamine:corrinoid methyltransferase	H	183	748	930
COG4977	Transcriptional regulator GlxA, contains an amidase domain and an AraC-type DNA-binding HTH domain	K	1427	1440	2868
COG1055	Na+/H+ antiporter NhaD or related arsenite permease	P	7618	8557	16174

COG2509	FAD-dependent dehydrogenase	R	15350	17118	32467
COG1700	Predicted anti-virus defense system component AQ645, contains DUF2357 and PD-(D/E)xK nuclease domains	V	831	627	1458
COG3293	Transposase	X	18605	15189	33794
COG1253	Hemolysin-related protein, contains CBS domains, UPF0053 family	R	15908	18316	34224
COG2176	DNA polymerase III, alpha subunit (gram-positive type)	L	21332	23580	44912
COG3713	Outer membrane scaffolding protein for murein synthesis, MipA/OmpV family	M	2	10	12
COG0224	FoF1-type ATP synthase, gamma subunit	C	6836	7786	14621
COG0266	Formamidopyrimidine-DNA glycosylase	L	805	1286	2090
COG1904	Glucuronate isomerase	G	6954	5892	12846
COG1540	5-oxoprolinase subunit A	E	216	789	1004
COG3920	Two-component sensor histidine kinase, HisKA and HATPase domains	T	18	202	220
COG5499	Antitoxin component HigA of the HigAB toxin-antitoxin module, contains an N-terminal HTH domain	V	363	255	617
COG1275	Tellurite resistance protein TehA and related permeases	V	44	311	355
COG3456	Predicted component of the type VI protein secretion system, contains a FHA domain	T;U	5	14	19
COG2849	Antitoxin component YwqK of the YwqJK toxin-antitoxin module	V	264	294	558
COG5708	Photosystem I reaction center subunit IX, PsaJ	C	1	0	1
COG0116	23S rRNA G2445 N2-methylase RlmL	J	8197	9103	17300
COG0370	Fe2+ transporter FeoB	P	28067	32973	61040

COG0775	Nucleoside phosphorylase/nucleosidase, includes 5'-methylthioadenosine/S-adenosylhomocysteine nucleosidase MtnN and futalosine hydrolase MqnB	F;H	5745	6623	12369
COG0688	Phosphatidylserine decarboxylase	I	3404	3968	7372
COG4066	Uncharacterized conserved protein MTH_811, UPF0305 family	S	8	41	48
COG0432	Thiamin phosphate synthase YjbQ, UPF0047 family	H	775	812	1587
COG4043	ASC-1 homology (ASCH) domain, predicted RNA-binding domain	R	872	1012	1884
COG0029	Aspartate oxidase	H	6759	8133	14892
COG1956	GAF domain-containing protein, putative methionine-R-sulfoxide reductase	V;T	664	772	1437
COG1151	Hydroxylamine reductase (hybrid-cluster protein)	C	2808	4261	7069
COG2128	Alkylhydroperoxidase family enzyme, contains CxxC motif	P	41	59	100
COG0138	AICAR transformylase/IMP cyclohydrolase PurH	F	8085	10571	18656
COG2269	Elongation factor P--beta-lysine ligase (EF-P beta-lysylation pathway)	J	0	4	4
COG2894	Septum site-determining ATPase MinD	D	4099	5359	9458
COG2265	tRNA/tmRNA/rRNA uracil-C5-methylase, TrmA/RlmC/RlmD family	J	19980	22605	42585
COG4509	Uncharacterized conserved protein	S	16632	16832	33464
COG2922	Uncharacterized conserved protein Smg, DUF494 family	S	0	2	2
COG1984	5-oxoprolinase subunit C/Allophanate hydrolase subunit 2	E	223	775	999
COG0495	Leucyl-tRNA synthetase	J	14950	18290	33240

COG3756	Uncharacterized conserved protein YdaU, DUF1376 family	S	121	157	278
COG3404	Formiminotetrahydrofolate cyclodeaminase	E	2784	3619	6403
COG2195	Di- or tripeptidase	E	14315	16855	31171
COG3816	Uncharacterized conserved protein, DUF1285 family	S	1	1	2
COG5042	Purine nucleoside permease	F	24	6	30
COG0404	Glycine cleavage system protein T (aminomethyltransferase)	E	3312	4218	7530
COG2152	Predicted glycosyl hydrolase, GH43/DUF377 family	G	5687	5680	11367
COG3823	Glutamine cyclotransferase	O	1538	1136	2674
COG0853	Aspartate 1-decarboxylase	H	1551	1517	3068
COG4624	Iron only hydrogenase large subunit, C-terminal domain	C	17566	20414	37980
COG1746	tRNA nucleotidyltransferase (CCA-adding enzyme)	J	87	107	194
COG3152	Uncharacterized membrane protein YhaH, DUF805 family	S	2327	3389	5717
COG1438	Arginine repressor	K	3548	3850	7398
COG3951	Rod binding protein domain	N	672	569	1241
COG0492	Thioredoxin reductase	O	13501	17684	31185
COG4304	Uncharacterized conserved protein, DUF2247 domain	S	26	12	39
COG4113	RNA interferase (RNase) VapC, contains PIN domain	V	1338	931	2269
COG3335	Transposase	X	10750	7520	18270
COG4965	Flp pilus assembly protein TadB	U;W	5774	5971	11745
COG3318	Uncharacterized conserved protein YecA, UPF0149 family, contains C-terminal Zn-binding SEC-C motif	S	4011	4876	8887
COG0763	Lipid A disaccharide synthetase	M	3064	4148	7212

COG1169	Isochorismate synthase EntC	H;Q	903	1662	2565
COG1120	ABC-type cobalamin/Fe3+-siderophores transport system, ATPase component	P;H	8973	9383	18356
COG0626	Cystathionine beta-lyase/cystathionine gamma-synthase	E	1086	1647	2732
COG4251	Bacteriophytochrome (light-regulated signal transduction histidine kinase)	T	31	52	83
COG0256	Ribosomal protein L18	J	3755	4638	8393
COG2864	Cytochrome b subunit of formate dehydrogenase	C	30	471	502
COG5078	Ubiquitin-protein ligase	O	21	6	27
COG0202	DNA-directed RNA polymerase, alpha subunit/40 kD subunit	K	8820	9701	18521
COG1502	Phosphatidylserine/phosphatidylglycerophosphate/cardiolipin synthase	I	14725	17690	32415
COG0422	4-amino-2-methyl-5-hydroxymethylpyrimidine (HMP) synthase ThiC	H	2540	3597	6136
COG0811	Biopolymer transport protein ExbB/TolQ	U	5670	7045	12715
COG4264	Siderophore synthetase component IucA/IucC/SbnC	P	0	28	28
COG1199	Rad3-related DNA helicase DinG	L	6345	7699	14043
COG3333	TctA family transporter	R	1297	3744	5041
COG4443	Uncharacterized conserved protein	S	330	389	719
COG0374	Ni,Fe-hydrogenase I large subunit	C	199	1057	1256
COG3771	Lipopolysaccharide assembly protein YciS/LapA, DUF1049 family	S	0	2	2
COG0491	Glyoxylase or a related metal-dependent hydrolase, beta-lactamase superfamily II	R	21841	24367	46208
COG2963	Transposase InsE and inactivated derivatives	X	7011	7996	15007
COG2188	DNA-binding transcriptional regulator, GntR family	K	4778	7855	12633

COG1942	Phenylpyruvate tautomerase PptA, 4-oxalocrotonate tautomerase family	Q	993	644	1637
COG3643	Glutamate formiminotransferase	E	308	939	1247
COG3261	Ni,Fe-hydrogenase III large subunit	C	1298	1389	2687
COG1056	Nicotinamide mononucleotide adenylyltransferase	H	288	445	733
COG1348	Nitrogenase ATPase subunit NifH/coenzyme F430 biosynthesis subunit	H;P	234	555	789
COG3064	Membrane protein TolA involved in colicin uptake	M	14808	18803	33612
COG0496	Broad specificity polyphosphatase and 5'/3'-nucleotidase SurE	L	3115	3663	6778
COG0467	RecA-superfamily ATPase, KaiC/GvpD/RAD55 family	T	1313	1162	2475
COG3876	Uncharacterized conserved protein YbbC, DUF1343 family	S	2464	2519	4983
COG4657	Na+-translocating ferredoxin:NAD+ oxidoreductase RNF, RnfA subunit	C	5032	4966	9997
COG4032	Sulfopyruvate decarboxylase, TPP-binding subunit (coenzyme M biosynthesis)	H	3	1	4
COG5874	Uncharacterized sporulation protein YtxC	D	294	637	931
COG3214	DNA glycosylase YcaQ, repair of DNA interstrand crosslinks	L	571	1075	1645
COG0081	Ribosomal protein L1	J	6310	7283	13593
COG2213	Phosphotransferase system, mannitol-specific IIBC component	G	155	308	462
COG1321	Mn-dependent transcriptional regulator MntR, DtxR family	K	4442	4681	9123
COG1513	Cyanate lyase	P	121	10	131
COG3557	Uncharacterized conserved protein associated with RNAses G and E, UPF0374/DUF402 family	S	422	437	858

COG3126	Uncharacterized lipoprotein YbaY	S	2	17	19
COG1785	Alkaline phosphatase	P;R	4614	5823	10436
COG1306	Predicted glycosyl hydrolase, alpha amylase family	R	3010	3024	6034
COG3227	Zn-dependent metalloprotease (Neutral protease B)	O	449	1147	1596
COG2943	Membrane glycosyltransferase	M;G	9	51	61
COG1135	ABC-type methionine transport system, ATPase component	E	2880	3577	6457
COG3397	Predicted carbohydrate-binding protein, contains CBM5 and CBM33 domains	R	28	76	104
COG4952	L-rhamnose isomerase	M	35	7	42
COG4175	ABC-type proline/glycine betaine transport system, ATPase component	E	87	168	254
COG1469	GTP cyclohydrolase FolE2	H	263	902	1165
COG2824	Uncharacterized Zn-ribbon-containing protein	R	1	5	6
COG0050	Translation elongation factor EF-Tu, a GTPase	J	6725	8542	15268
COG2238	Ribosomal protein S19E (S16A)	J	7	4	11
COG2818	3-methyladenine DNA glycosylase Tag	L	1410	1724	3134
COG0484	DnaJ-class molecular chaperone with C-terminal Zn finger domain	O	17540	21972	39512
COG2231	3-Methyladenine DNA glycosylase, HhH-GPD/Endo3 superfamily	L	65	51	116
COG0645	Predicted kinase, contains AAA domain	R	1893	1583	3476
COG3809	Predicted nucleic acid-binding protein, contains Zn-finger domain	R	4	44	49
COG3981	Predicted acetyltransferase	R	4064	4136	8200
COG1559	Endolytic transglycosylase MltG, terminates peptidoglycan polymerization	M	9981	9769	19750
COG5808	Sporulation sensor histidine kinase D	D;T	15	45	61

COG4760	Uncharacterized membrane protein, YccA/Bax inhibitor family	S	154	219	373
COG2246	Putative flippase GtrA (transmembrane translocase of bactoprenol-linked glucose)	I	7240	7391	14631
COG4698	Uncharacterized conserved protein YpmS, DUF2140 family	S	49	51	100
COG3392	Adenine-specific DNA methylase	L	3068	3294	6362
COG2154	Pterin-4a-carbinolamine dehydratase	H	85	732	817
COG1511	Uncharacterized membrane protein YhgE, phage infection protein (PIP) family	S	2376	3082	5458
COG0024	Methionine aminopeptidase	J	11979	12637	24616
COG5274	Cytochrome b involved in lipid metabolism	C;I	6	21	27
COG0151	Phosphoribosylamine-glycine ligase	F	9550	11215	20766
COG4705	Uncharacterized membrane-anchored	S	2	4	6
COG4872	Uncharacterized membrane protein	S	25	113	138
COG5405	ATP-dependent protease HslVU (ClpYQ), peptidase subunit	O	377	746	1123
COG3290	Sensor histidine kinase DipB regulating citrate/malate metabolism	T	41379	30543	71922
COG4619	ABC-type iron transporter FetAB, ATPase component	P	6	5	11
COG3243	Poly-beta-hydroxybutyrate synthase	I	49	33	82
COG1660	RNase adaptor protein RapZ for GlmZ sRNA degradation, contains a P-loop ATPase domain	T	6283	6851	13134
COG5803	Stage 0 sporulation initiation response regulator Spo0F	D;T	14	93	107
COG2153	Predicted N-acyltransferase, GNAT family	R	2571	1944	4514
COG0292	Ribosomal protein L20	J	2706	3244	5950
COG4692	Predicted neuraminidase (sialidase)	G;M	1782	2673	4455
COG3697	Phosphoribosyl-dephospho-CoA transferase (holo-ACP synthetase)	H;I	264	159	423

COG2856	Zn-dependent peptidase ImmA, M78 family	O	6987	7084	14071
COG0041	Phosphoribosylcarboxyaminoimidazole (NCAIR) mutase	F	4594	5501	10094
COG2730	Aryl-phospho-beta-D-glucosidase BglC, GH1 family	G	17180	13093	30273
COG3075	Anaerobic glycerol-3-phosphate dehydrogenase	E	181	306	487
COG5418	Predicted secreted protein	S	76	186	262
COG5568	Uncharacterized conserved protein, DUF1150 family	S	0	2	2
COG4763	Uncharacterized membrane protein YcfT	S	5	2	7
COG2520	tRNA G37 N-methylase Trm5	J	1607	1380	2987
COG3023	N-acetyl-anhydromuramyl-L-alanine amidase AmpD	M	4042	4406	8448
COG1695	DNA-binding transcriptional regulator, PadR family	K	10536	12383	22920
COG1240	vWFA (von Willebrand factor type A) domain of Mg and Co chelatases	H	12835	14192	27027
COG1268	Biotin transporter BioY	H	4641	5121	9762
COG2202	PAS domain	T	14785	14755	29540
COG3161	4-hydroxybenzoate synthetase (chorismate-pyruvate lyase)	H	2	2	4
COG3262	Ni,Fe-hydrogenase III component G	C	0	28	28
COG1591	Holliday junction resolvase Hjc, archaeal type	L	46	20	66
COG1241	DNA replicative helicase MCM subunit Mcm2, Cdc46/Mcm family	L	29	23	52
COG5512	Predicted nucleic acid-binding protein, contains Zn-ribbon domain (includes truncated derivatives)	R	40	75	114
COG5586	Uncharacterized protein, contains DUF2293 domain	S	285	349	635

COG1696	D-alanyl-lipoteichoic acid acyltransferase DltB, MBOAT superfamily	M	31016	31742	62757
COG1476	DNA-binding transcriptional regulator, XRE-family HTH domain	K	18559	18105	36663
COG1230	Co/Zn/Cd efflux system component	P	813	1146	1959
COG3454	Alpha-D-ribose 1-methylphosphonate 5-triphosphate diphosphatase PhnM	P	200	129	329
COG1974	SOS-response transcriptional repressor LexA (RecA-mediated autopeptidase)	K;T	7712	10287	17998
COG1413	HEAT repeat	R	3811	4985	8796
COG1766	Flagellar biosynthesis/type III secretory pathway M-ring protein FliF/YscJ	N;U	4545	3908	8453
COG5606	Predicted DNA-binding protein, XRE-type HTH domain	R	42	108	151
COG2206	HD-GYP domain, c-di-GMP phosphodiesterase class II (or its inactivated variant)	T	17003	15383	32386
COG0691	tmRNA-binding protein	O	4569	5534	10103
COG1891	4-(hydroxymethyl)-2-furancarboxaldehyde phosphate synthase MfnB	H	2	9	11
COG1175	ABC-type sugar transport system, permease component	G	31439	40294	71732
COG1787	Endonuclease, HJR/Mrr/RecB family	V	860	1068	1928
COG2360	Leu/Phe-tRNA-protein transferase	O	86	600	686
COG0129	Dihydroxyacid dehydratase/phosphogluconate dehydratase	E;G	5386	7971	13357
COG2175	Taurine dioxygenase, alpha-ketoglutarate-dependent	Q	6	14	20
COG3494	Uncharacterized conserved protein, DUF1009 family	S	337	1119	1456
COG0526	Thiol-disulfide isomerase or thioredoxin	O	7842	11857	19699

COG1349	DNA-binding transcriptional regulator of sugar metabolism, DeoR/GlpR family	K;G	4976	8053	13029
COG2992	Uncharacterized FlgJ-related protein	R	15	106	121
COG5425	Usg protein (tryptophan operon, function unknown)	S	0	2	2
COG2078	Predicted RNA modification protein, AMMECR1 domain	R	1096	1684	2781
COG4770	Acetyl/propionyl-CoA carboxylase, alpha subunit	I	5805	6263	12068
COG5410	Uncharacterized conserved protein	S	3433	6495	9928
COG1572	Serine protease, subtilase family	O	373	382	755
COG4481	Uncharacterized conserved protein, DUF951 family	S	654	728	1382
COG5891	Spore coat protein YheC/YheD, ATP-grasp superfamily	D	1	30	31
COG3507	Beta-xylosidase	G	32624	32972	65596
COG1574	Predicted amidohydrolase YtcJ	R	1800	3895	5695
COG2952	Uncharacterized conserved protein, DUF507 domain	S	12	0	12
COG2135	ssDNA abasic site-binding protein YedK/HMCES, SRAP family	L	713	1055	1769
COG1291	Flagellar motor component MotA	N	3430	2643	6073
COG1166	Arginine decarboxylase (spermidine biosynthesis)	E	3243	3491	6733
COG4401	Chorismate mutase AroH	E	42	150	192
COG1394	Archaeal/vacuolar-type H+-ATPase subunit D/Vma8	C	3659	3803	7462
COG4101	Uncharacterized conserved protein, RmlC-like cupin domain	R	0	3	3
COG3631	Ketosteroid isomerase-related protein	R	143	170	313
COG1647	Esterase/lipase	Q	625	1087	1712
COG0170	Dolichol kinase	O;M	442	415	857

COG3483	Tryptophan 2,3-dioxygenase (vermilion)	E	1	1	2
COG4373	Mu-like prophage FluMu protein gp28	X	284	1688	1973
COG0773	UDP-N-acetylmuramate-alanine ligase MurC and related ligases, MurC/Mpl family	M	12656	15393	28049
COG1468	CRISPR/Cas system-associated exonuclease Cas4, RecB family	V	2048	1901	3949
COG0648	Endonuclease IV	L	7901	8349	16250
COG4269	Uncharacterized membrane protein YjgN, DUF898 family	S	82	80	162
COG1286	Colicin V production accessory protein CvpA, regulator of purF expression and biofilm formation	F;M	1651	1748	3399
COG2215	ABC-type nickel/cobalt efflux system, permease component RcnA	P	82	15	97
COG2356	Endonuclease I	L	4730	4805	9536
COG1949	Oligoribonuclease (3'-5' exoribonuclease)	A	0	117	117
COG1254	Acylphosphatase	C	258	415	672
COG4413	Urea transporter	E	1298	1121	2419
COG5836	Stage V sporulation protein SpoVAC, subunit of dipicolinate uptake complex	D;E	3593	3971	7564
COG4715	Uncharacterized protein, contains SWIM-type Zn finger domain	S	2032	2554	4586
COG4107	ABC-type phosphonate transport system, ATPase component PhnK	P	120	31	152
COG0582	Integrase/recombinase, includes phage integrase	L;X	3122	4452	7574
COG4667	Predicted phospholipase, patatin/cPLA2 family	I	7808	7740	15547
COG4559	ABC-type hemin transport system, ATPase component	P	1	5	6
COG5717	Photosystem II reaction center chlorophyll a-binding protein CP47, PsbB	C	21	8	30

COG2355	Zn-dependent dipeptidase, microsomal dipeptidase homolog	O;E	4237	6408	10645
COG2937	Glycerol-3-phosphate O-acyltransferase	I	961	933	1894
COG2063	Flagellar basal body L-ring protein FlgH	N	32	104	136
COG1916	Pheromone shutdown protein TraB, contains GTxH motif (function unknown)	S	12	22	34
COG3151	Uncharacterized conserved protein YqiB, DUF1249 family	S	4	6	10
COG1645	Uncharacterized Zn-finger containing protein, UPF0148 family	R	1781	2124	3905
COG1951	Tartrate dehydratase alpha subunit/Fumarate hydratase class I, N-terminal domain	C	4621	5476	10097
COG0639	Diadenosine tetraphosphatase ApaH/serine/threonine protein phosphatase, PP2A family	T	666	1068	1735
COG4991	Uncharacterized conserved protein YraI	S	1920	3532	5452
COG1428	Deoxyadenosine/deoxycytidine kinase	F	1548	1206	2754
COG2385	Peptidoglycan hydrolase (amidase) enhancer domain SpoIID	M	12657	13914	26571
COG1614	CO dehydrogenase/acetyl-CoA synthase beta subunit	C	127	159	285
COG2348	Lipid II:glycine glycyltransferase (Peptidoglycan interpeptide bridge formation enzyme)	M	4037	4076	8113
COG2173	D-alanyl-D-alanine dipeptidase	M	1507	1982	3489
COG4706	Predicted 3-hydroxylacyl-ACP dehydratase, HotDog domain	I	20	81	101
COG3722	DNA-binding transcriptional regulator, MltR family	K	2	6	8
COG1191	DNA-directed RNA polymerase specialized sigma subunit	K	12288	14152	26440
COG4388	Mu-like prophage I protein	X	153	797	949

COG3251	MbtH family protein, regulates adenylation domains of NRPSs	Q	2	9	11
COG5712	Photosystem I assembly protein Ycf4	C	15	25	40
COG3886	HKD family nuclease	L	259	104	363
COG4521	ABC-type taurine transport system, periplasmic component	P	27	12	39
COG5562	Prophage-encoded protein YbcV, DUF1398 family	X	2	2	3
COG4988	ABC-type transport system involved in cytochrome bd biosynthesis, ATPase and permease components	C;O	1095	949	2045
COG1295	Uncharacterized membrane protein, BrkB/YihY/UPF0761 family (not an RNase)	S	7261	7908	15168
COG0683	ABC-type branched-chain amino acid transport system, periplasmic component	E	7306	9672	16978
COG0850	Septum site-determining protein MinC	D	1683	2143	3826
COG3711	Transcriptional antiterminator	K	1593	3906	5499
COG3884	Acyl-ACP thioesterase	I	7169	8338	15508
COG3740	Phage head maturation protease	X	2074	1986	4060
COG5510	Predicted small secreted protein	S	0	0	0
COG1315	Flagellar assembly protein FapA, interacts with EIIAGlc	N	9598	6853	16451
COG3814	SspB-like protein, predicted to bind SsrA peptide	O	21	83	104
COG4703	Uncharacterized conserved protein YkuJ, DUF1797 family	S	17	18	35
COG1743	Adenine-specific DNA methylase, contains a Zn-ribbon domain	L	704	1347	2051
COG2912	Regulator of sirC expression, contains transglutaminase-like and TPR domains	T	101	15	116
COG0196	FAD synthase	H	9598	10351	19948
COG5635	Predicted NTPase, NACHT family domain	T	2854	2214	5068

COG0777	Acetyl-CoA carboxylase beta subunit	I	2965	4023	6987
COG4769	Uncharacterized membrane protein	S	3825	3709	7534
COG1653	ABC-type glycerol-3-phosphate transport system, periplasmic component	G	70487	81028	151514
COG4980	Gas vesicle protein YhaH	R	445	470	915
COG0193	Peptidyl-tRNA hydrolase	J	7488	8397	15884
COG3966	Poly-D-alanine transfer protein DltD, involved in esterification of teichoic acids	M	240	250	490
COG2311	Uncharacterized membrane protein YeiB	S	700	1031	1732
COG2252	Xanthine/guanine/uracil/vitamin C permease GhxP/GhxQ, nucleobase:cation symporter 2 (NCS2) family	F	3064	4619	7683
COG3833	ABC-type maltose transport system, permease component MalG	G	3039	2829	5868
COG2920	Sulfur transfer complex TusBCD TusE component, DsrC family (tRNA 2-thiouridine synthesizing protein C)	J	6	14	19
COG0749	DNA polymerase I, 3'-5' exonuclease and polymerase domains	L	8215	8397	16611
COG1673	Predicted RNA-binding protein, contains PUA-like EVE domain	R	62	37	98
COG1022	Long-chain acyl-CoA synthetase (AMP-forming)	I	13377	16280	29657
COG5838	Stage V sporulation protein SpoVAEA, mutants lead to the production of immature spores	D	1	52	53
COG4336	Uncharacterized conserved protein YcsI, UPF0317/DUF1446 family	S	107	271	377
COG3679	Cell fate regulator YlbF, YheA/YmcA/DUF963 family (controls sporulation, competence, biofilm development)	T	1069	1203	2272

COG1940	Sugar kinase of the NBD/HSP70 family, may contain an N-terminal HTH domain	G;K	22693	30691	53383
COG0150	Phosphoribosylaminoimidazole (AIR) synthetase	F	8541	9845	18385
COG0259	Pyridoxine/pyridoxamine 5'-phosphate oxidase	H	244	559	804
COG0296	1,4-alpha-glucan branching enzyme	G	22164	23182	45346
COG0727	Uncharacterized protein YkgJ, contains CxxCxxCC motif	R	2047	2957	5004
COG4630	Xanthine dehydrogenase, Fe-S cluster and FAD-binding subunit XdhA	F	1	3	3
COG0176	Transaldolase/fructose-6-phosphate aldolase	G	1513	2165	3678
COG1494	Fructose-1,6-bisphosphatase/sedoheptulose 1,7-bisphosphatase or related protein	G	20	58	78
COG3692	Uncharacterized conserved protein YifN, PemK superfamily	S	334	359	693
COG2158	Uncharacterized conserved protein, contains a Zn-finger-like domain	R	264	427	691
COG4836	Uncharacterized membrane protein YwzB	S	17	18	35
COG3838	Type IV secretory pathway, VirB2 component (pilin)	U	10	48	58
COG4567	DNA-binding response regulator, ActR/RegA family, consists of REC and Fis-type HTH domains	T;K	40	36	76
COG5665	CCR4-NOT transcriptional regulation complex, NOT5 subunit	K	26	42	68
COG0210	Superfamily I DNA or RNA helicase	L	29769	35317	65086
COG5471	Predicted phage recombinase, RecA/RadA family	X	118	72	190
COG1816	Adenosine deaminase	F	3357	3804	7161

COG4295	Predicted RNA repair component, a phosphatase of H2Macro superfamily, DUF2263 family	R	1638	1304	2942
COG0184	Ribosomal protein S15P/S13E	J	1803	2054	3856
COG1934	Lipopolysaccharide export system protein LptA	M	2966	2941	5908
COG3845	ABC-type guanosine uptake system NupNOPQ, ATPase component NupO	F	5285	7562	12847
COG0707	UDP-N-acetylglucosamine:LPS N-acetylglucosamine transferase	M	15846	19663	35509
COG0341	Preprotein translocase subunit SecF	U	10803	11668	22471
COG5279	Cytokinesis protein 3, contains TGc (transglutaminase/protease-like) domain	D	21495	19378	40873
COG5018	3'-5' exonuclease KapD, inhibitor of KinA-controlled sporulation	T	2403	2284	4687
COG1373	Predicted ATPase, AAA+ superfamily	R	58113	63944	122057
COG3647	Uncharacterized membrane protein YjdF	S	2	0	2
COG1244	Archaeosine formation enzyme, radical SAM superfamily	J	1045	1016	2061
COG4813	Trehalose utilization protein	G	808	512	1321
COG2308	Circularly permuted ATP-grasp protein	R	1107	1503	2611
COG3129	23S rRNA A1618 N6-methylase RlmF	J	1	4	5
COG4146	Uncharacterized membrane permease YidK, sodium:solute symporter family	R	1699	2171	3869
COG2203	GAF domain	T	607	542	1149
COG1648	Siroheme synthase (precorrin-2 oxidase/ferrochelatase domain)	H	619	1109	1728
COG1084	GTP-binding protein, GTP1/Obg family	R	0	1	1
COG3879	Uncharacterized conserved protein YlxW, UPF0749 family	S	619	1204	1823
COG0217	Transcriptional and/or translational regulatory protein YebC/TACO1	K;J	5918	7895	13813

COG2847	Copper(I)-binding protein	P	0	3	3
COG3657	Putative component of the toxin-antitoxin plasmid stabilization module	V	21	180	201
COG0246	Mannitol-1-phosphate/altronate dehydrogenases	G	7922	8247	16169
COG3419	Type IV pilus assembly protein, tip-associated adhesin PilY1	N;W	7	0	7
COG5455	Periplasmic regulator RcnB of Ni and Co efflux	P	5	4	9
COG1550	Stress-induced protein YlxP, DUF503	S	17	61	78
COG0325	Pyridoxal 5'-phosphate homeostasis protein YggS, UPF0001 family	H	7616	7817	15433
COG2983	Uncharacterized cysteine cluster protein YcgN, CxxCxxCC family	R	12	150	162
COG4395	Predicted lipid-binding transport protein, Tim44 family	I	325	730	1055
COG0465	ATP-dependent Zn proteases	O	21743	26326	48069
COG3232	5-carboxymethyl-2-hydroxymuconate isomerase	E	0	0	0
COG3815	Uncharacterized membrane protein	S	144	133	277
COG2362	D-aminopeptidase	E	532	1012	1544
COG0459	Chaperonin GroEL (HSP60 family)	O	10143	11551	21694
COG1048	Aconitase A	C	7507	10505	18013
COG4850	Phosphatidate phosphatase APP1	I	0	3	3
COG3528	Uncharacterized conserved protein, DUF2219 family	S	290	748	1038
COG5379	S-adenosylmethionine:diacylglycerol 3-amino-3-carboxypropyl transferase	I	81	132	213
COG5006	Threonine/homoserine efflux transporter RhtA	E	645	755	1399
COG0735	Fe2+ or Zn2+ uptake regulation protein Fur/Zur	P	5431	6035	11466

COG3449	DNA gyrase inhibitor GyrI/SbmC	L	99	159	258
COG2425	Uncharacterized conserved protein, contains a von Willebrand factor type A (vWA) domain	S	4396	4432	8828
COG4652	Uncharacterized conserved protein, DUF1430 domain	S	939	543	1482
COG2911	Autotransporter translocation and assembly protein TamB	U	8931	8597	17528
COG4383	Mu-like prophage protein gp29	X	3130	3906	7036
COG4117	Thiosulfate reductase cytochrome b subunit	P	0	2	2
COG5949	CRISPR-Cas system type I-E effector complex small subunit Cse2	V	103	133	236
COG3197	Cytochrome oxidase maturation protein, CcoS/FixS family	O	3	0	3
COG3791	Uncharacterized conserved protein	S	20	17	37
COG4096	Type I site-specific restriction endonuclease, part of a restriction-modification system	V	10505	12215	22720
COG0718	DNA-binding nucleoid-associated protein YbaB/EfbC	K	2529	3342	5870
COG3267	Type II secretory pathway ATPase component GspA/ExeA/MshM	U;W	3468	6450	9917
COG2975	Fe-S-cluster formation regulator IscX/YfhJ	O	0	14	14
COG1621	Sucrose-6-phosphate hydrolase SacC, GH32 family	G	7891	11591	19482
COG1948	ERCC4-type crossover junction endonuclease	L	5	418	423
COG3688	EndoRNase involved in mRNA decay, NYN (Nedd4-BP1/Rae1/YacP nuclease) family, contains PIN domain	J	201	309	510
COG1259	Bifunctional DNase/RNase	R	916	1320	2236
COG1330	Scaffold subunit RecC of the DNA repair enzyme RecBCD (exonuclease V)	L	3	318	322

COG3199	NAD kinase	F	25	40	64
COG0655	Multimeric flavodoxin WrbA, includes NAD(P)H:quinone oxidoreductase	C	19598	22190	41788
COG1204	Replicative superfamily II helicase	L	4048	3432	7481
COG0846	NAD-dependent protein deacetylase, SIR2 family	O	10955	12712	23667
COG0748	Putative heme iron utilization protein, contains PNPOx domain	P	19	0	19
COG1631	Ribosomal protein L44E	J	3	2	5
COG0493	NADPH-dependent glutamate synthase beta chain or related oxidoreductase	E;R	16942	20706	37648
COG4870	Cysteine protease, C1A family	O	2311	2832	5143
COG1908	Coenzyme F420-reducing hydrogenase, delta subunit	C	223	360	583
COG0746	Molybdopterin-guanine dinucleotide biosynthesis protein A	H	483	716	1199
COG3040	Bacterial lipocalin Blc	M	291	532	823
COG1972	Nucleoside permease NupC	F	283	492	775
COG5465	Uncharacterized conserved protein YbjN, helps stabilize unstable proteins	R	347	522	869
COG4641	Spore maturation protein CgeB	D	5542	4121	9663
COG2301	Citrate lyase beta subunit	G	1687	2127	3815
COG2321	Predicted metalloprotease	R	550	853	1402
COG4787	Flagellar basal body rod protein FlgF	N	1	5	5
COG5401	Spore germination protein GerM	D	3227	4599	7826
COG3743	Predicted 5' DNA nuclease, flap endonuclease-1-like, helix-3-turn-helix (H3TH) domain	L	32	147	179
COG2956	Lipopolysaccharide biosynthesis regulator YciM/LapB, contains six TPR domains and a C-terminal metal-binding domain	M	1483	1925	3408
COG5618	Predicted periplasmic lipoprotein	S	19	66	85

COG4377	Predicted metal-dependent membrane protease YhfC, DUF2324 family	R	1025	744	1769
COG4223	Uncharacterized conserved protein	S	0	61	61
COG4258	Predicted exporter	R	10	41	51
COG1669	Predicted nucleotidyltransferase MJ0435	R	5411	5653	11064
COG3387	Glucoamylase (glucan-1,4-alpha-glucosidase), GH15 family	G	462	1141	1604
COG5839	Stage V sporulation protein SpoVAEB, subunit of dipicolinate uptake complex	D;E	2199	2567	4766
COG5911	Spore germination protease Gpr	D	5821	6662	12483
COG3142	Copper homeostasis protein CutC	P	1390	2368	3758
COG3407	Mevalonate pyrophosphate decarboxylase	I	75	77	151
COG3118	Chaperedoxin CnoX, contains thioredoxin-like and TPR-like domains, YbbN/TrxSC family	O	5747	6882	12630
COG3638	ABC-type phosphate/phosphonate transport system, ATPase component	P	160	172	333
COG4796	Type II secretory pathway, component	U	16	227	243
COG4925	Uncharacterized conserved protein	S	5366	5347	10714
COG0509	Glycine cleavage system protein H (lipoate-binding)	E	1374	1684	3058
COG3189	Uncharacterized conserved protein YeaO, DUF488 family	S	26	296	322
COG1239	Mg-chelatase subunit ChlI	H	301	474	774
COG3738	Uncharacterized conserved protein YijF, DUF1287 family	S	627	1010	1637
COG0205	6-phosphofructokinase	G	16686	21753	38439
COG0818	Diacylglycerol kinase	I	1579	1595	3173
COG4660	Na+-translocating ferredoxin:NAD+ oxidoreductase RNF, RnfE subunit	C	6505	6446	12951
COG0524	Sugar or nucleoside kinase, ribokinase	G	20824	27727	48551

COG1185	Polyribonucleotide nucleotidyltransferase (polynucleotide phosphorylase)	J	11585	14944	26529
COG4957	Predicted transcriptional regulator	K	18	72	90
COG3119	Arylsulfatase A or related enzyme, AlkP superfamily	P	14083	20158	34241
COG1026	Zn-dependent peptidase, M16 (insulinase) family	O	6331	6729	13060
COG1038	Pyruvate carboxylase	C	840	1791	2631
COG3963	Phosphatidylethanolamine N-methyltransferase	I	755	973	1728
COG0522	Ribosomal protein S4 or related protein	J	5815	7266	13082
COG0774	UDP-3-O-acyl-N-acetylglucosamine deacetylase	M	90	740	830
COG2766	Predicted Ser/Thr protein kinase	T	0	4	4
COG1091	dTDP-4-dehydrorhamnose reductase	M	8549	9476	18025
COG3476	Tryptophan-rich sensory protein TspO/CrtK (mitochondrial benzodiazepine receptor homolog)	T	3003	3025	6028
COG1282	NAD/NADP transhydrogenase beta subunit	C	175	284	459
COG1850	Ribulose 1,5-bisphosphate carboxylase, large subunit, or a RuBisCO-like protein	G	107	349	456
COG1354	Chromatin segregation and condensation protein Rec8/ScpA/Scc1, kleisin family	L	5344	6850	12194
COG5553	Predicted metal-dependent enzyme of the double-stranded beta helix superfamily	R	1	2	2
COG0229	Peptide methionine sulfoxide reductase	O	51	374	425
COG5476	Microcystin degradation protein MlrC, contains DUF1485 domain	R	212	256	468
COG1846	DNA-binding transcriptional regulator, MarR family	K	11779	14244	26023
COG1127	ATPase subunit MlaF of the ABC-type intermembrane phospholipid transporter Mla	M	1982	2992	4974

COG3702	Type IV secretory pathway, VirB3 component	U	0	77	77
COG2959	Proteobacterial HemX domain, involved in 2-ketogluconate production (unrelated to B. subtilis HemX, COG0755, no evidence of involvement in heme biosynthesis)	R	0	2	2
COG3138	Arginine/ornithine N-succinyltransferase beta subunit	E	2	8	9
COG1310	Proteasome lid subunit RPN8/RPN11, contains Jab1/MPN domain metalloenzyme (JAMM) motif	O	148	106	254
COG4880	Secreted protein containing C-terminal beta-propeller domain distantly related to WD-40 repeats	R	2465	2550	5016
COG5523	Uncharacterized membrane protein	S	4025	5002	9027
COG3442	Glutamine amidotransferase related to the GATase domain of CobQ	R	1676	2339	4015
COG1709	Predicted transcriptional regulator,contains XRE-type HTH domain	K	416	252	668
COG3656	Predicted periplasmic protein	S	375	328	703
COG4644	Transposase and inactivated derivatives, TnpA family	X	19	46	66
COG1427	Chorismate dehydratase (menaquinone biosynthesis, futalosine pathway)	H	331	360	692
COG2981	Sulfate transporter CysZ	E;P	60	58	118
COG4606	ABC-type enterochelin transport system, permease component	P	150	200	349
COG1902	2,4-dienoyl-CoA reductase or related NADH-dependent reductase, Old Yellow Enzyme (OYE) family	C	5536	8915	14451
COG5504	Predicted Zn-dependent protease YjaZ, DUF2268 family	R	238	161	400

COG0418	Dihydroorotase	F	283	607	889
COG0200	Ribosomal protein L15	J	5673	6504	12177
COG3393	Predicted acetyltransferase, GNAT family	R	7239	7010	14249
COG2262	50S ribosomal subunit-associated GTPase HflX	J	9976	10711	20687
COG1125	ABC-type proline/glycine betaine transport system, ATPase component	E	310	541	852
COG2087	Adenosyl cobinamide kinase/adenosyl cobinamide phosphate guanylyltransferase	H	4042	4202	8245
COG0352	Thiamine monophosphate synthase	H	5366	6719	12085
COG2240	Pyridoxal/pyridoxine/pyridoxamine kinase	H	5485	5633	11118
COG2333	DNA uptake channel protein ComEC C-terminal domain, metallo-beta-lactamase superfamily	U	11094	13657	24751
COG2831	Hemolysin activation/secretion protein	U	27	320	347
COG1520	Outer membrane protein assembly factor BamB, contains PQQ-like beta-propeller repeat	M	2316	3703	6019
COG1874	Beta-galactosidase GanA	G	11588	12990	24578
COG0215	Cysteinyl-tRNA synthetase	J	11536	13901	25437
COG3733	Cu2+-containing amine oxidase	Q	0	3	3
COG2454	Predicted nuclease, contains PIN domain	R	351	347	699
COG2021	Homoserine O-acetyltransferase	E	1539	2074	3613
COG4544	Uncharacterized conserved protein	S	2	4	6
COG4232	Thiol:disulfide interchange protein DsbD	O	5196	6653	11849
COG0128	5-enolpyruvylshikimate-3-phosphate synthase	E	10312	11718	22030
COG1188	Ribosomal 50S subunit-recycling heat shock protein, contains S4 domain	J	2578	2551	5129
COG2198	HPt (histidine-containing phosphotransfer) domain	T	2958	2551	5509

COG1845	Heme/copper-type cytochrome/quinol oxidase, subunit 3	C	264	220	484
COG0715	ABC-type nitrate/sulfonate/bicarbonate transport system, periplasmic component	P	9320	12993	22313
COG1047	Peptidyl-prolyl cis-trans isomerase, FKBP type	O	1929	2111	4040
COG3505	Type IV secretory pathway, VirD4 component, TraG/TraD family ATPase	U	37979	42011	79990
COG0695	Glutaredoxin	O	376	469	845
COG2832	Uncharacterized membrane protein YbaN, DUF454 family	S	389	341	730
COG2411	Predicted RNA-binding protein, contains PUA-like ASCH domain	R	38	37	75
COG5402	Uncharacterized protein, contains DUF1214 domain	S	0	3	4
COG0410	ABC-type branched-chain amino acid transport system, ATPase component LivF	E	2622	4518	7140
COG3048	D-serine dehydratase	E	174	597	771
COG1322	DNA anti-recombination protein (rearrangement mutator) RmuC	L	5304	6204	11508
COG0527	Aspartate kinase	E	11917	13209	25126
COG1775	Benzoyl-CoA reductase/2-hydroxyglutaryl-CoA dehydratase subunit,	E;Q	2150	3711	5861
COG5017	UDP-N-acetylglucosamine transferase subunit ALG13	G	770	780	1549
COG0276	Protoheme ferro-lyase (ferrochelatase)	H	116	368	484
COG2402	Predicted nucleic acid-binding protein, contains PIN domain	R	12	8	20
COG0114	Fumarate hydratase class II	C	832	1341	2173
COG4578	DNA-binding transcriptional regulator of glucitol operon	K	190	190	380

COG0765	ABC-type amino acid transport system, permease component	E	9497	10771	20268
COG1440	Phosphotransferase system cellobiose-specific component IIB	G	406	618	1024
COG3827	Cell pole-organizing protein PopZ	D	2022	2999	5021
COG0117	Riboflavin biosynthesis protein RibD, pyrimidine deaminase domain	H	2913	3167	6080
COG1512	Uncharacterized membrane protein YgcG, contains a TPM-fold domain	S	4876	5803	10680
COG2255	Holliday junction resolvasome RuvABC, ATP-dependent DNA helicase subunit RuvB	L	7704	10206	17910
COG0359	Ribosomal protein L9	J	5533	6465	11998
COG0452	Phosphopantothenoylcysteine synthetase/decarboxylase CoaBC	H	6768	8036	14803
COG5506	Uncharacterized conserved protein YueI, DUF2278 family	S	40	52	92
COG1516	Flagellin-specific chaperone FliS	N;U	2671	2281	4952
COG4102	Uncharacterized conserved protein, DUF1501 family	S	1	6	6
COG1684	Flagellar biosynthesis protein FliR	N	2922	2541	5463
COG4152	ABC-type uncharacterized transport system, ATPase component	R	6208	5529	11737
COG0579	L-2-hydroxyglutarate oxidase LhgO	G	3823	4526	8349
COG1712	L-aspartate dehydrogenase, NAD(P)-dependent	E	405	861	1266
COG1528	Ferritin	P	2538	3154	5692
COG3306	Glycosyltransferase involved in LPS biosynthesis, GR25 family	M	854	750	1605
COG3196	Colicin E2 tolerance protein CbrC, UPF0167 family	R	291	128	418
COG5266	Uncharacterized protein, contains GH25 family domain	R	29	158	186

COG2976	Putative negative regulator of RcsB-dependent stress response, UPF0070 family	T	1	143	143
COG2837	Periplasmic deferrochelatase/peroxidase EfeB	P	107	114	221
COG4512	Accessory gene regulator protein AgrB	K;T	5057	2677	7735
COG0480	Translation elongation factor EF-G, a GTPase	J	29633	32663	62296
COG1861	Spore coat polysaccharide biosynthesis protein SpsF, cytidylyltransferase family	M	649	472	1120
COG0627	S-formylglutathione hydrolase FrmB	V	2925	3282	6207
COG0320	Lipoate synthase	H	2480	2954	5434
COG3544	Uncharacterized conserved protein, DUF305 family	S	2891	2463	5353
COG0464	AAA+-type ATPase, SpoVK/Ycf46/Vps4 family	M;D;T	13011	12538	25548
COG3327	DNA-binding transcriptional regulator PaaX (phenylacetic acid degradation)	K	0	1	1
COG0583	DNA-binding transcriptional regulator, LysR family	K	32953	47483	80436
COG1791	Acireductone dioxygenase (methionine salvage), cupin superfamily	E	3	6	8
COG3728	Phage terminase, small subunit	X	1984	1846	3830
COG3622	Hydroxypyruvate/dehydroerythronate isomerase, Hyi/OtnI family	G	269	425	694
COG0551	DNA topoisomerase I, ssDNA-binding Zn-finger and Zn-ribbon domains	L	2255	2783	5038
COG3549	Plasmid maintenance system killer protein	V	363	371	734
COG0099	Ribosomal protein S13	J	2801	3068	5869
COG2069	CO dehydrogenase/acetyl-CoA synthase delta subunit (corrinoid Fe-S protein)	C	998	1202	2200
COG3162	Uncharacterized membrane protein, DUF485 family	S	7	0	7

COG2830	Uncharacterized conserved protein, DUF452 domain	S	429	580	1009
COG2339	Membrane proteinase PrsW, cleaves anti-sigma factor RsiW, M82 family	T	1616	998	2614
COG2761	Predicted dithiol-disulfide isomerase, DsbA/YjbH family (virulence, stress resistance)	O	238	454	691
COG1250	3-hydroxyacyl-CoA dehydrogenase	I	1194	3375	4569
COG3712	Periplasmic ferric-dicitrate binding protein FecR, regulates iron transport	P;T	5020	7671	12691
COG0262	Dihydrofolate reductase	H	5388	5869	11257
COG4104	Zn-binding Pro-Ala-Ala-Arg (PAAR) domain, involved in Type VI secretion	U	276	137	413
COG0003	Anion-transporting ATPase, ArsA/GET3 family	P	105	155	260
COG3588	Fructose-bisphosphate aldolase class 1	G	43	41	84
COG3083	Periplasmic protein PbgA/YejM, regulator of the LPS biosynthesis, AlkP superfamily	M;T	197	382	579
COG0442	Prolyl-tRNA synthetase	J	11782	14239	26020
COG2011	ABC-type methionine transport system, permease component	E	2258	2746	5005
COG4270	Uncharacterized membrane protein	S	0	1	2
COG1019	Phosphopantetheine adenylyltransferase	H	1	3	4
COG3157	Type VI protein secretion system component Hcp (secreted cytotoxin)	U	21	9	30
COG1277	ABC-type transport system involved in multi-copper enzyme maturation, permease component	O	6277	8618	14896
COG3828	Type 1 glutamine amidotransferase (GATase1)-like domain	R	406	1202	1608
COG2738	Zn-dependent membrane protease YugP	O	5623	6510	12133
COG2988	Succinylglutamate desuccinylase	E	0	3	3

COG3597	Uncharacterized conserved protein, DUF697 family	S	507	606	1113
COG3762	Uncharacterized membrane protein	S	49	159	208
COG0045	Succinyl-CoA synthetase, beta subunit	C	97	1029	1125
COG2945	Alpha/beta superfamily hydrolase	R	275	537	812
COG0578	Glycerol-3-phosphate dehydrogenase	C	558	1175	1732
COG1082	Sugar phosphate isomerase/epimerase	G	17489	21498	38987
COG1697	DNA topoisomerase VI, subunit A	L	666	658	1324
COG2177	Cell division protein FtsX	D	10609	10681	21290
COG5339	Uncharacterized conserved protein YdgA, DUF945 family	S	0	4	4
COG5338	Uncharacterized conserved protein	S	2	6	8
COG0705	Membrane-associated serine protease, rhomboid family	O	9794	10739	20533
COG4483	Uncharacterized conserved protein YqgQ, DUF910 family	S	8	11	19
COG3734	2-keto-3-deoxy-galactonokinase	G	153	249	402
COG1053	Succinate dehydrogenase/fumarate reductase, flavoprotein subunit	C	8929	16287	25216
COG0704	Phosphate uptake regulator PhoU	P	4773	6052	10825
COG1702	Phosphate starvation-inducible protein PhoH, predicted ATPase	T	7651	8812	16463
COG2965	Primosomal replication protein N	L	1	54	55
COG2897	3-mercaptopyruvate sulfurtransferase SseA, contains two rhodanese domains	P	147	307	454
COG4181	Predicted ABC-type transport system involved in lysophospholipase L1 biosynthesis, ATPase component	Q	18	66	84
COG4470	Uncharacterized conserved protein YutD, DUF1027 family	S	204	238	442
COG0612	Predicted Zn-dependent peptidase, M16 family	R	26524	30465	56989

COG3334	Flagellar motility protein MotE, a chaperone for MotC folding	N	1556	1497	3053
COG4828	Uncharacterized membrane protein	S	224	298	522
COG3870	Cyclic di-AMP receptor DarA/PstA	T	939	757	1697
COG3236	N-glycosidase YbiA/RibX (riboflavin biosynthesis, damage control), NADAR superfamily	H;V	1414	1713	3127
COG1251	NAD(P)H-nitrite reductase, large subunit	C	2636	4135	6771
COG0337	3-dehydroquinate synthetase	E	9966	11728	21694
COG3355	Predicted transcriptional regulator	K	122	325	447
COG4704	Uncharacterized conserved protein, DUF2141 family	S	3276	2764	6040
COG1108	ABC-type Mn2+/Zn2+ transport system, permease component	P	5537	6085	11622
COG3297	Type II secretory pathway, component PulL	U	0	2	2
COG3804	Uncharacterized conserved protein	S	2381	3806	6187
COG1457	Purine-cytosine permease or related protein	F	718	1427	2145
COG2143	Thioredoxin-related protein SoxW	O	359	1151	1510
COG1961	Site-specific DNA recombinase SpoIVCA/DNA invertase PinE	L	150458	155880	306337
COG0239	Fluoride ion exporter CrcB/FEX, affects chromosome condensation	D;P	1676	1771	3447
COG4457	Virulence factor SrfB-related protein	Q	542	623	1166
COG2948	Type IV secretory pathway, VirB10 component	U	122	687	809
COG4929	Uncharacterized membrane-anchored	S	39	75	114
COG2111	Multisubunit Na+/H+ antiporter, MnhB subunit	P	1770	1906	3676
COG5495	Predicted oxidoreductase, contains short-chain dehydrogenase (SDR) and DUF2520 domains	R	2802	2708	5511

COG1807	PMT family glycosyltransferase ArnT/Agl22, involved in glycosylation of proteins and lipid IVA	M	6835	9776	16611
COG1670	Protein N-acetyltransferase, RimJ/RimL family	J;O	27929	30236	58165
COG3961	TPP-dependent 2-oxoacid decarboxylase, includes indolepyruvate decarboxylase	G;H;R	39	148	187
COG5581	Cyclic di-GMP-binding flagellar brake protein FlgZ/YcgR, contains PilZNR(YcgR) and PilZ domains	N	1338	1268	2606
COG3727	G:T-mismatch repair DNA endonuclease Vsr, very short patch repair protein	L	2665	3170	5835
COG4723	Phage-related protein, tail assembly protein I	X	1	1	1
COG2225	Malate synthase	C	6	17	23
COG3561	Phage anti-repressor protein Ant	X	1310	1274	2584
COG3192	Ethanolamine transporter EutH, required for ethanolamine utilization at low pH	E	1268	1984	3252
COG4176	ABC-type proline/glycine betaine transport system, permease component	E	68	149	216
COG3047	Outer membrane protein OmpW	M	85	129	214
COG0739	Murein DD-endopeptidase MepM and murein hydrolase activator NlpD, contains LysM domain	M	44501	47989	92490
COG0130	tRNA U55 pseudouridine synthase TruB, may also work on U342 of tmRNA	J	9822	10918	20740
COG0600	ABC-type nitrate/sulfonate/bicarbonate transport system, permease component	P	7448	10596	18044
COG3689	Uncharacterized membrane protein YcgQ, UPF0703/DUF1980 family	S	1483	1182	2666
COG0365	Acyl-coenzyme A synthetase/AMP-(fatty) acid ligase	I	3385	3866	7251

COG3970	Fumarylacetoacetate (FAA) hydrolase family protein	R	2	6	7
COG2182	Maltose-binding periplasmic protein MalE	G	7671	8052	15722
COG2226	Ubiquinone/menaquinone biosynthesis C-methylase UbiE/MenG	H	30353	32949	63301
COG0597	Lipoprotein signal peptidase	M;U	7208	8180	15388
COG2103	N-acetylmuramic acid 6-phosphate (MurNAc-6-P) etherase	M	784	1358	2142
COG0323	DNA mismatch repair ATPase MutL	L	18028	19246	37274
COG3165	Ubiquinone biosynthesis protein UbiJ, contains SCP2 domain	H	0	1	1
COG0140	Phosphoribosyl-ATP pyrophosphohydrolase	E	516	472	987
COG3968	Glutamine synthetase type III	E	12685	15111	27795
COG0182	5-methylthioribose/5-deoxyribulose 1-phosphate isomerase (methionine salvage pathway), a paralog of eIF-2B alpha subunit	E	2728	2752	5480
COG5814	Sporulation sigma factor SigK (mother cell-specific, SpoIIIC/SpoIVCB)	D;K	2481	3483	5964
COG1606	ATP-utilizing enzyme, PP-loop superfamily	R	2244	3357	5601
COG4849	Predicted nucleotidyltransferase	R	337	675	1012
COG0804	Urease alpha subunit	E	2001	2014	4015
COG0649	NADH:ubiquinone oxidoreductase 49 kD subunit (chain D)	C	1598	1929	3528
COG0398	Uncharacterized membrane protein YdjX, related to fungal oxalate transporter, TVP38/TMEM64 family	S	3372	4983	8355
COG4143	ABC-type thiamine transport system, periplasmic component TbpA	H	0	5	5
COG0847	DNA polymerase III, epsilon subunit or related 3'-5' exonuclease	L	7014	7342	14356
COG0644	Dehydrogenase (flavoprotein)	C	4426	5623	10049
COG0197	Ribosomal protein L16/L10AE	J	2629	3531	6160

COG5016	Pyruvate/oxaloacetate carboxyltransferase	C	5996	7570	13567
COG4940	Competence protein ComGF	X	0	1	1
COG2982	Uncharacterized conserved protein AsmA involved in outer membrane biogenesis	M	3845	4578	8423
COG0431	NAD(P)H-dependent FMN reductase	C	299	321	619
COG3609	Transcriptional regulator, contains Arc/MetJ-type RHH (ribbon-helix-helix) DNA-binding domain	K	159	203	362
COG1691	NCAIR mutase (PurE)-related protein	F	2109	3266	5375
COG3172	Nicotinamide riboside kinase	H	3033	2957	5989
COG4545	Glutaredoxin-related protein	O	111	96	207
COG1334	Uncharacterized conserved protein, FlaG/YvyC family	R	955	902	1857
COG3154	Ubiquinone biosynthesis accessory factor UbiT, lipid carrier SCP2 domain	H	2	1	4
COG1058	ADP-ribose pyrophosphatase domain of DNA damage- and competence-inducible protein CinA	L	6027	7206	13232
COG5008	Type IV pilus assembly protein, ATPase	N;W	6	5	10
COG3367	Uncharacterized conserved protein, NAD-dependent epimerase/dehydratase family	R	24	4	28
COG0004	Ammonia channel protein AmtB	P	5461	4881	10342
COG0378	Hydrogenase/urease maturation factor HypB, Ni2+-binding GTPase	O	2093	3081	5174
COG5565	Phage terminase large (ATPase) subunit and inactivated derivatives	X	328	833	1161
COG1786	Mevalonate 5-phosphate dehydratase subunit 2, swiveling domain (modified mevalonate pathway)	I	39	101	140
COG3703	Gamma-glutamylcyclotransferase ChaC2 (glutathione degradation)	P	1	4	6

COG5897	Spore coat protein YsxE, aminoglycoside phosphotransferase family	D	57	158	215
COG5746	Light-independent protochlorophyllide reductase subunit B, BchB (= ChlB)	H	0	2	2
COG0385	Predicted Na+-dependent transporter YfeH	R	3604	4387	7991
COG1358	Ribosomal protein L7Ae or related RNA K-turn-binding protein	J	2621	2907	5528
COG4473	Predicted ABC-type exoprotein transport system, permease component	U	83	77	159
COG0670	Integral membrane protein YbhL, putative Ca2+ regulator, Bax inhibitor (BI-1)/TMBIM family	P	2478	2886	5364
COG1682	ABC-type polysaccharide/teichoic acid/polyol phosphate export permease	G	6895	7047	13942
COG3893	Inactivated superfamily I helicase	L	3	5	9
COG1748	Saccharopine dehydrogenase, NADP-dependent	E	3231	4191	7422
COG4948	L-alanine-DL-glutamate epimerase or related enzyme of enolase superfamily	M;R	2905	5116	8022
COG1917	Cupin domain protein related to quercetin dioxygenase	R	8846	10647	19493
COG2007	Ribosomal protein S8E	J	15	10	25
COG0601	ABC-type dipeptide/oligopeptide/nickel transport system, permease component	E;P	11586	19549	31135
COG1419	Flagellar biosynthesis GTPase FlhF	N	1836	1853	3688
COG1219	ATP-dependent protease Clp, ATPase subunit ClpX	O	13814	16126	29940
COG4286	Uncharacterized conserved protein, UPF0160 family	S	236	423	659
COG0815	Apolipoprotein N-acyltransferase	M	2607	3225	5832
COG1463	Periplasmic subunit MlaD of the ABC-type intermembrane phospholipid transporter Mla	M	2521	3553	6073

COG3527	Alpha-acetolactate decarboxylase	Q	52	103	155
COG4714	Uncharacterized membrane-anchored	S	2	5	7
COG0505	Carbamoylphosphate synthase small subunit	E;F	7930	9626	17556
COG0141	Histidinol dehydrogenase	E	5366	6656	12021
COG3164	Uncharacterized conserved protein YhdP, contains DUF3971 and AsmA2 domains	S	8	600	607
COG3376	High-affinity nickel/cobalt permease	P	0	5	5
COG1436	Archaeal/vacuolar-type H+-ATPase subunit F/Vma7	C	3777	4553	8330
COG3604	FhlA-type transcriptional regulator, contains GAF, AAA-type ATPase, and DNA-binding Fis domains	K;T	397	964	1361
COG0028	Acetolactate synthase large subunit or other thiamine pyrophosphate-requiring enzyme	E;H	18521	20240	38761
COG0720	6-pyruvoyl-tetrahydropterin synthase	H	2201	2242	4444
COG3732	Phosphotransferase system sorbitol-specific IIB component	G	354	485	839
COG5436	Uncharacterized membrane protein	S	1	2	4
COG3225	ABC-type uncharacterized transport system involved in gliding motility, auxiliary component	N	4312	4230	8541
COG2703	Hemerythrin	T	1789	1503	3292
COG5617	Predicted membrane glycosyltransferase TK1552, contains 6-pyruvoyl-tetrahydropterin synthase (PTPS)-related domain	R	3496	4144	7640
COG5722	Photosystem II reaction center 10 kDa phosphoprotein, PsbH	C	8	4	13
COG3605	Signal transduction protein containing GAF and PtsI domains	T	49	172	222
COG1264	Phosphotransferase system IIB components	G	61	77	138

COG4456	Virulence-associated protein VagC (function unknown)	S	124	173	297
COG1607	Acyl-CoA hydrolase	I	574	1034	1609
COG3070	Transcriptional regulator of competence genes, TfoX/Sxy family	K	1668	2152	3820
COG2115	Xylose isomerase	G	2115	2230	4345
COG0546	Phosphoglycolate phosphatase, HAD superfamily	C	19929	21511	41440
COG0833	Amino acid permease	E	237	464	701
COG1287	Asparagine N-glycosylation enzyme, membrane subunit Stt3	O	32	52	84
COG1773	Flavorubredoxin	P	2556	3569	6126
COG0800	2-keto-3-deoxy-6-phosphogluconate aldolase	G	4848	4869	9717
COG1296	Predicted branched-chain amino acid permease (azaleucine resistance)	E	4868	5731	10599
COG5812	Sporulation sigma factor SigF (forespore-specific, SpoIIAC)	D;K	1547	2313	3861
COG2259	Uncharacterized membrane protein YphA, DoxX/SURF4 family	S	3002	3686	6688
COG0545	FKBP-type peptidyl-prolyl cis-trans isomerase	O	6612	6892	13504
COG4506	Uncharacterized beta-barrel protein YwiB, DUF1934 family	S	2648	2751	5398
COG3560	Fatty acid repression mutant protein (predicted oxidoreductase)	R	620	517	1137
COG4913	Uncharacterized conserved protein, contains a C-terminal ATPase domain	S	3771	2989	6760
COG2379	Glycerate-2-kinase	G	1082	2198	3280
COG4816	Ethanolamine utilization protein EutL, microcompartment shell protein	E	752	1233	1985

COG5820	Stage II sporulation protein SpoIIQ, required for engulfement	D	535	424	958
COG0672	High-affinity Fe2+/Pb2+ permease	P	8	10	18
COG2126	Ribosomal protein L37E	J	0	0	1
COG5352	Uncharacterized conserved protein	S	22	120	142
COG0556	Excinuclease UvrABC helicase subunit	L	11494	14803	26297
COG4674	ABC-type uncharacterized transport system, ATPase component	R	94	18	112
COG0355	FoF1-type ATP synthase, epsilon subunit	C	3132	3206	6338
COG0265	Periplasmic serine protease, S1-C subfamily, contain C-terminal PDZ domain	O	25383	32683	58066
COG0134	Indole-3-glycerol phosphate synthase	E	4151	4310	8461
COG2073	Cobalamin biosynthesis protein CbiG	H	2863	2934	5797
COG3628	Phage baseplate assembly protein W	X	1426	1179	2604
COG3322	Extracellular (periplasmic) sensor domain CHASE (specificity unknown)	T	3	4	7
COG3434	c-di-GMP phosphodiesterase YuxH/PdeH, contains EAL and HDOD domains	T	3143	2116	5259
COG2179	Predicted phosphohydrolase YqeG, HAD superfamily	R	3705	4123	7829
COG0678	Peroxiredoxin	O	12	12	23
COG0479	Succinate dehydrogenase/fumarate reductase, Fe-S protein subunit	C	2117	2811	4928
COG3191	L-aminopeptidase/D-esterase	E	863	1629	2492
COG1778	3-deoxy-D-manno-octulosonate 8-phosphate phosphatase KdsC and related HAD superfamily phosphatases	M;R	2843	2548	5391
COG1140	Nitrate reductase beta subunit	C;P	1	238	239
COG0821	4-hydroxy-3-methylbut-2-en-1-yl diphosphate synthase IspG/GcpE	I	9707	11101	20808
COG4805	Uncharacterized conserved protein, DUF885 family	S	3118	3962	7080

COG3471	Predicted secreted (periplasmic) protein	S	84	165	248
COG0679	Predicted permease, AEC (auxin efflux carrier) family	R	9533	11056	20589
COG1518	CRISPR-Cas system-associated integrase Cas1	V	4709	5451	10160
COG5153	Putative lipase ATG15 (essential for vacuolar disintegration of autophagic	U	3	6	9
COG0346	Catechol 2,3-dioxygenase or related enzyme, vicinal oxygen chelate (VOC)	Q	3750	5193	8943
COG0731	Wyosine [tRNA(Phe)-imidazoG37] synthetase, radical SAM superfamily	J	2318	2491	4809
COG1577	Mevalonate kinase	I	156	171	327
COG1136	ABC-type lipoprotein export system, ATPase component	M	56314	57984	114298
COG0490	K+/H+ antiporter KhtSTU, c-di-AMP-binding regulatory subunit KhtT, contains RCK_C (TrkA_C) domain	P;T	102	105	207
COG4392	Branched-chain amino acid transport protein	E	708	640	1348
COG2946	DNA relaxase NicK	L	755	543	1298
COG1679	Mevalonate 5-phosphate dehydratase subunit 1, aconitase superfamily (modified mevalonate pathway)	I	116	250	367
COG5547	Uncharacterized membrane protein	S	36	57	92
COG5272	Ubiquitin	O	101	131	232
COG4103	Tellurite resistance protein TerB	P	46	597	643
COG0321	Lipoate-protein ligase B	H	1729	2109	3838
COG2312	Erythromycin esterase homolog	Q	204	168	373
COG0077	Prephenate dehydratase	E	7753	8679	16431
COG0851	Septum formation topological specificity factor MinE	D	535	918	1452
COG1198	Primosomal protein N' (replication factor Y) - superfamily II helicase	L	15721	18529	34251

COG0165	Argininosuccinate lyase	E	6887	9312	16199
COG0544	FKBP-type peptidyl-prolyl cis-trans isomerase (trigger factor)	O	15642	17768	33410
COG2164	Uncharacterized protein with cyclophilin fold, contains DUF369 domain	R	5	0	5
COG3596	Predicted GTPase	R	2410	3332	5743
COG5505	Uncharacterized membrane protein YjcL	S	1176	820	1997
COG0186	Ribosomal protein S17	J	1267	1482	2749
COG2433	Possible nuclease of RNase H fold, RuvC/YqgF family	R	41	38	79
COG0641	Sulfatase maturation enzyme AslB, radical SAM superfamily	O	21873	24560	46432
COG3311	DNA-binding transcriptional regulator AlpA	K;X	4875	4830	9705
COG1073	Fermentation-respiration switch esterase FrsA, DUF1100 family	T	10392	11126	21519
COG2074	2-phosphoglycerate kinase/Mevalonate-3-phosphate 5-kinase	G;I	81	86	167
COG0684	RNA degradosome component RraA (regulator of RNase E activity)	J	968	2055	3023
COG4822	Cobalamin biosynthesis protein CbiK, Co2+ chelatase	H	3252	3738	6990
COG0728	Lipid II flippase MurJ/MviN (peptidoglycan biosynthesis)	M	1383	2854	4237
COG3000	Sterol desaturase/sphingolipid hydroxylase, fatty acid hydroxylase superfamily	I	15	26	40
COG1910	Periplasmic molybdate-binding protein/domain	P	64	96	160
COG0122	3-methyladenine DNA glycosylase/8-oxoguanine DNA glycosylase	L	5495	5576	11072
COG0531	Serine transporter YbeC, amino acid:H+ symporter family	E	4906	7967	12872
COG1765	Uncharacterized OsmC-related protein	R	646	887	1534

COG2241	Precorrin-6B methylase 1	H	1799	1857	3656
COG4812	Ethanolamine utilization protein EutT, cobalamin adenosyltransferase	E	801	1142	1943
COG1527	Archaeal/vacuolar-type H+-ATPase subunit C/Vma6	C	3500	3637	7136
COG5580	Activator of HSP90 ATPase	O	26	25	51
COG2009	Succinate dehydrogenase/fumarate reductase, cytochrome b subunit	C	28	265	293
COG0476	Molybdopterin or thiamine biosynthesis adenylyltransferase	H	4908	6278	11186
COG4469	Competence protein CoiA, contains predicted nuclease domain	R	440	967	1406
COG3113	Binding protein subunit MlaB of the ABC-type intermembrane phospholipid transporter Mla, contains STAS domain	M	0	1	1
COG3875	Nickel-dependent lactate racemase	M	999	3399	4398
COG2456	Uncharacterized conserved protein, DUF2304 domain	S	295	493	788
COG5350	Predicted protein tyrosine phosphatase	R	12	28	40
COG4139	ABC-type cobalamin transport system, permease component BtuC	H	196	230	426
COG0230	Ribosomal protein L34	J	288	167	455
COG3864	Predicted metal-dependent peptidase	R	3291	3894	7185
COG0389	Nucleotidyltransferase/DNA polymerase DinP involved in DNA repair	L	15430	18867	34298
COG0068	Hydrogenase maturation factor HypF (carbamoyltransferase)	O	832	2056	2888
COG4649	TPR-like repeat domain	S	23	906	929
COG4154	L-fucose mutarotase/ribose pyranase, RbsD/FucU family	G	602	1025	1627
COG0342	Preprotein translocase subunit SecD	U	2899	3322	6221

COG4453	Uncharacterized conserved protein, DUF1778 family	S	6	5	11
COG0006	Xaa-Pro aminopeptidase	E	16624	19628	36252
COG2310	Stress response protein SCP2	T	4044	3011	7055
COG2926	Uncharacterized conserved protein YeeX, DUF496 family	S	0	1	1
COG2816	NADH pyrophosphatase NudC, Nudix superfamily	F	2749	3109	5858
COG5917	Uncharacterized sporulation protein YjbA, contains UPF0736/DUF3603 domain	D	7	11	18
COG1114	Branched-chain amino acid permease	E	350	793	1143
COG4656	Na+-translocating ferredoxin:NAD+ oxidoreductase RNF, RnfC subunit	C	10767	13330	24096
COG5297	Cellulase/cellobiase CelA1	G	6590	4951	11541
COG5542	Mannosyltransferase related to Gpi18	G	2444	2545	4989
COG4819	Ethanolamine utilization protein EutA, possible chaperonin	E	1089	1638	2727
COG0173	Aspartyl-tRNA synthetase	J	15785	18041	33826
COG5569	Periplasmic Cu and Ag efflux protein CusF	P	0	1	1
COG3807	SH3-like domain	S	9	72	82
COG3569	DNA topoisomerase IB	L	3	5	8
COG0360	Ribosomal protein S6	J	3562	3789	7351
COG4137	ABC-type uncharacterized transport system, permease component	R	7	548	555
COG3566	Uncharacterized conserved protein, DUF2213 domain	S	575	804	1379
COG1732	Periplasmic glycine betaine/choline-binding (lipo)protein of an ABC-type transport system (osmoprotectant binding protein)	M	82	141	222
COG3081	dsDNA-binding nucleoid-associated protein YejK/NdpA	L	950	769	1719

COG5000	Signal transduction histidine kinase NtrY involved in nitrogen fixation and metabolism regulation	T	2544	4214	6759
COG4635	Protoporphyrinogen IX oxidase, menaquinone-dependent (flavodoxin	H	2076	1715	3791
COG1526	Formate dehydrogenase assembly factor FdhD, a sulfurtransferase	C	177	233	409
COG0623	Enoyl-[acyl-carrier-protein] reductase FabI	I	934	1866	2800
COG2391	Uncharacterized membrane protein YedE/YeeE, contains two sulfur transport domains	R	133	306	439
COG0133	Tryptophan synthase beta chain	E	4007	4405	8412
COG1313	Radical SAM superfamily enzyme PflX	R	6208	7324	13533
COG5002	Sensor histidine kinase WalK	T	28190	33659	61849
COG2453	Protein-tyrosine phosphatase	T	223	251	474
COG3769	Mannosyl-3-phosphoglycerate phosphatase YedP/MpgP, HAD superfamily	G	0	3	3
COG0056	FoF1-type ATP synthase, alpha subunit	C	7186	8336	15522
COG4065	Uncharacterized conserved protein MJ1451	S	0	1	1
COG5923	Uncharacterized sporulation membrane protein YrzE, contains DUF3792 domain	D	814	938	1752
COG2124	Cytochrome P450	Q;V	20	24	44
COG5385	Histidine phosphotransfer protein ChpT, HPt domain	T	13	68	81
COG0001	Glutamate-1-semialdehyde aminotransferase	H	2292	4597	6889
COG4712	Predicted ssDNA annealing protein, contains HHH motif, Rad42/Rad22/RecT/erf family	L	1129	1188	2318
COG5848	SigmaK-processing regulatory protein BofA (Bypass-of-forespore protein A)	D;T	212	394	606
COG4695	Phage portal protein BeeE	X	8194	9170	17364
COG1887	CDP-glycerol glycerophosphotransferase, TagB/SpsB family	M;I	11294	11656	22951

COG4790	Type III secretory pathway, EscR/YscR component	U	1	4	5
COG1051	ADP-ribose pyrophosphatase YjhB, NUDIX family	F	22416	24225	46641
COG2048	Heterodisulfide reductase, subunit B	C	1699	1913	3612
COG4092	Predicted glycosyltransferase involved in capsule biosynthesis	M	6	1	7
COG1871	Chemotaxis receptor (MCP) glutamine deamidase CheD	T	1825	1491	3317
COG1233	Phytoene dehydrogenase-related protein	Q	1036	1408	2444
COG3059	Reactive chlorine resistance protein RclC/YkgB, DUF417 family	V	9	29	38
COG1538	Outer membrane protein TolC	M	23655	27614	51269
COG2067	Long-chain fatty acid transport protein	I	9360	9024	18385
COG3965	Predicted Co/Zn/Cd cation transporter, cation efflux family	P	113	109	223
COG3894	Uncharacterized 2Fe-2S and 4Fe-4S clusters-containing protein, contains DUF4445 domain	S	4249	4910	9159
COG5827	Stage III sporulation protein SpoIIIAF, component of the engulfment complex	D	376	535	911
COG3095	Chromosome condensin MukBEF, MukE localization factor	D	0	0	0
COG2329	Heme-degrading monooxygenase HmoA and related ABM domain proteins	H	2	6	8
COG3594	Fucose 4-O-acetylase or related acetyltransferase	G	7749	8038	15787
COG3411	2Fe-2S ferredoxin	C	484	784	1267
COG0147	Anthranilate/para-aminobenzoate synthases component I	E;H	5031	5500	10530
COG4318	Uncharacterized conserved protein	S	3	5	8

COG0450	Alkyl hydroperoxide reductase subunit AhpC (peroxiredoxin)	V	1773	2408	4181
COG0843	Heme/copper-type cytochrome/quinol oxidase, subunit 1	C	297	245	542
COG5869	Spore cortex formation membrane protein YabQ	D	1362	1525	2888
COG3514	Uncharacterized conserved protein, DUF4415 family	S	206	234	440
COG4195	Phage-related replication protein YjqB, UPF0714/DUF867 family	X	9	21	31
COG0137	Argininosuccinate synthase	E	3694	5618	9312
COG1161	Ribosome biogenesis GTPase RbgA	J	6663	8198	14861
COG5258	GTPase	R	0	0	0
COG5881	Spore coat protein CotI/CotS, protein kinase superfamily	D	2318	2789	5107
COG1216	Glycosyltransferase, GT2 family	G	23109	24714	47822
COG1661	Predicted DNA-binding protein with PD1-like DNA-binding motif, PPC/DUF296 domain	R	366	485	851
COG1733	DNA-binding transcriptional regulator, HxlR family	K	3933	3570	7504
COG0021	Transketolase	G	5903	7854	13757
COG2442	Predicted antitoxin component of a toxin-antitoxin system, DUF433 family	V	57	93	150
COG0284	Orotidine-5'-phosphate decarboxylase	F	8502	9515	18017
COG1671	Uncharacterized conserved protein YaiI, UPF0178 family	S	462	423	884
COG3062	Cytoplasmic chaperone NapD for the signal peptide of periplasmic nitrate reductase NapAB	O	22	10	32
COG5050	sn-1,2-diacylglycerol ethanolamine- and cholinephosphotranferases	I	80	152	231

COG1011	FMN and 5-amino-6-(5-phospho-D-ribitylamino)uracil phosphatase YigB, HAD superfamily (riboflavin biosynthesis)	H	16441	18471	34912
COG0841	Multidrug efflux pump subunit AcrB	V	22387	30427	52814
COG0512	Anthranilate/para-aminobenzoate synthase component II (glutamine amidotransferase)	E;H	3218	3295	6513
COG4810	Ethanolamine utilization protein EutS, microcompartment shell protein	E	135	356	491
COG1434	Lipid carrier protein ElyC involved in cell wall biogenesis, DUF218 family	M	5934	6620	12554
COG4632	Sugar-P-sugar glycosidase (uncovering enzyme, UCE), NAGPA superfamily	G	10166	11979	22146
COG2030	Acyl-CoA dehydratase PaaZ	I	648	1287	1935
COG4744	Uncharacterized conserved protein, DUF2149 domain	S	166	205	371
COG1692	2',3'- and 3',5'-cNMP phosphodiesterase YmdB, calcineurin family	T	4257	4562	8819
COG4927	Predicted choloylglycine hydrolase	R	1835	2590	4426
COG3219	Uncharacterized conserved protein, DUF2063 family	S	3	2	5
COG3589	Uncharacterized conserved protein, DUF871 domain	S	130	806	936
COG4959	Type IV secretory pathway, protease TraF	O;U	341	845	1186
COG0792	Predicted endonuclease distantly related to archaeal Holliday junction resolvase, YraN/UPF0102 family	L	4037	4791	8828
COG0500	SAM-dependent methyltransferase	Q;R	15	147	162
COG1680	CubicO group peptidase, beta-lactamase class C family	V	15016	15932	30947
COG3764	Sortase (surface protein transpeptidase)	M	10517	15107	25624
COG2077	Peroxiredoxin	O	853	798	1651
COG5633	Uncharacterized conserved protein YcfL	S	0	1	1

COG4683	Uncharacterized conserved protein	S	147	151	298
COG1592	Rubrerythrin	C	12731	14143	26874
COG0516	IMP dehydrogenase/GMP reductase	F	7570	9557	17127
COG5585	Putative phage head morphogenesis protein, F of phage Mu or gp7 of SPP1	X	724	970	1694
COG3719	Ribonuclease I	J	0	3	4
COG0179	2-keto-4-pentenoate hydratase/2-oxohepta-3-ene-1,7-dioic acid hydratase (catechol pathway)	Q	2664	3640	6303
COG4702	Uncharacterized conserved protein, UPF0303 family	S	83	207	290
COG3063	Type IV pilus assembly protein PilF/PilW	N;W	13173	16543	29716
COG1280	Threonine/homoserine/homoserine lactone efflux protein	E	3449	3652	7101
COG1966	Carbon starvation protein CstA (peptide/pyruvate transporter)	C;E	5241	6099	11339
COG1267	Phosphatidylglycerophosphatase A	I	531	705	1237
COG2088	DNA-binding protein SpoVG, cell septation regulator	D	3705	3558	7263
COG4257	Streptogramin lyase	V	271	402	673
COG5718	Photosystem II reaction center chlorophyll a-binding protein CP43, PsbC	C	19	12	31
COG2110	O-acetyl-ADP-ribose deacetylase (regulator of RNase III), contains Macro domain	J	6878	7409	14287
COG2109	ATP:corrinoid adenosyltransferase	H	2566	2785	5351
COG4710	Predicted DNA-binding protein with an HTH domain	R	313	298	612
COG0211	Ribosomal protein L27	J	1022	1407	2430
COG2949	Uncharacterized periplasmic protein SanA, affects membrane permeability for vancomycin	M	1271	1731	3002

COG5570	Uncharacterized conserved protein, DUF465 domain	S	0	1	1
COG1857	CRISPR-Cas system type I effector complex subunit Cas7, RAMP superfamily	V	186	232	418
COG4548	Nitric oxide reductase activation protein	P	2370	2525	4895
COG1521	Pantothenate kinase type III	H	8328	8826	17153
COG0682	Prolipoprotein diacylglyceryltransferase	M	11743	13002	24745
COG5185	Chromosome segregation protein NDC80, interacts with SMC proteins	D	50	30	80
COG1854	S-ribosylhomocysteine lyase LuxS, autoinducer biosynthesis	T	2336	2609	4945
COG1270	Cobalamin biosynthesis protein CobD/CbiB	H	3384	4582	7966
COG4022	Uncharacterized conserved protein, DUF2099 domain	S	62	97	159
COG5842	Dipicolinate synthase subunit A (sporulation protein SpoVFA)	D;E	4347	3872	8219
COG0630	Type IV secretory pathway ATPase VirB11/Archaellum biosynthesis ATPase ArlI/FlaI	N;U	221	961	1182
COG3298	Predicted 3'-5' exonuclease related to the exonuclease domain of PolB	L	16	0	16
COG3895	Membrane-bound inhibitor of C-type lysozyme	M	0	3	3
COG4684	Uncharacterized membrane protein	S	2671	2863	5534
COG4680	mRNA-degrading endonuclease (mRNA interferase) HigB, toxic component of the HigAB toxin-antitoxin module	J;V	302	167	470
COG5829	Stage III sporulation protein SpoIIIAH, component of the engulfment complex	D	4231	4847	9078
COG5433	Predicted transposase YbfD/YdcC associated with H repeats	X	30187	35279	65467

COG1962	Tetrahydromethanopterin S-methyltransferase, subunit H	H	0	1	1
COG3971	2-keto-4-pentenoate hydratase	Q	19	246	265
COG4924	Uncharacterized conserved protein	S	998	873	1871
COG3869	Protein-arginine kinase McsB	O	2312	3445	5757
COG2872	Ser-tRNA(Ala) deacylase AlaX (editing enzyme)	J	418	571	989
COG0402	Cytosine/adenosine deaminase or related metal-dependent hydrolase	F;R	10225	14187	24412
COG4845	Chloramphenicol O-acetyltransferase	V	3590	4369	7959
COG3475	Phosphorylcholine metabolism protein LicD	I	8174	8355	16529
COG2217	Cation-transporting P-type ATPase	P	31784	37515	69300
COG3128	Predicted 2-oxoglutarate- and Fe(II)-dependent dioxygenase YbiX	R	5	11	16
COG0506	Proline dehydrogenase	E	567	1297	1863
COG1988	Membrane-bound metal-dependent hydrolase YbcI, DUF457 family	R	404	294	698
COG3473	Maleate cis-trans isomerase	Q	35	166	201
COG3009	Intermembrane transporter PqiABC lipoprotein subunit PqiC	M	16	71	88
COG4508	Dimeric dUTPase, all-alpha-NTP-PPase (MazG) superfamily	F	51	134	186
COG5435	Uncharacterized conserved protein	S	0	2	2
COG2191	Formylmethanofuran dehydrogenase subunit E	C	107	223	329
COG0366	Glycosidase/amylase (phosphorylase)	G	41010	45477	86487
COG0796	Glutamate racemase	M	7885	8606	16490
COG1403	5-methylcytosine-specific restriction endonuclease McrA	V	6220	7528	13748
COG2099	Precorrin-6x reductase	H	2783	3576	6359
COG4573	Tagatose-1,6-bisphosphate aldolase non-catalytic subunit AgaZ/GatZ	G	570	1065	1635

COG0783	DNA-binding ferritin-like protein (oxidative damage protectant)	P;V	241	383	624
COG0566	tRNA G18 (ribose-2'-O)-methylase SpoU	J	24355	25872	50227
COG3550	Serine/threonine protein kinase HipA, toxin component of the HipAB toxin-antitoxin module	T	7191	8700	15891
COG4794	Type III secretory pathway, EscS/YscS component	U	0	1	1
COG1098	Predicted RNA-binding protein, contains ribosomal protein S1 (RPS1) domain	R	1739	1768	3507
COG0191	Fructose/tagatose bisphosphate aldolase	G	7013	10608	17621
COG1897	Homoserine O-succinyltransferase	E	4957	5465	10422
COG2823	Osmotically-inducible protein OsmY, contains BON domain	M	12	176	187
COG0258	5'-3' exonuclease Xni/ExoIX (flap endonuclease)	L	17966	22346	40312
COG1158	Transcription termination factor Rho	K	6920	9265	16185
COG0411	ABC-type branched-chain amino acid transport system, ATPase component LivG	E	3135	4890	8025
COG2227	2-polyprenyl-3-methyl-5-hydroxy-6-metoxy-1,4-benzoquinol methylase	H	20898	19272	40170
COG4591	ABC-type transport system involved in lipoprotein release, permease component LolC	M	54264	61482	115746
COG2771	DNA-binding transcriptional regulator, CsgD family	K	366	843	1209
COG3863	Uncharacterized conserved protein YycO, NlpC/P60 family	S	1495	1456	2951
COG1986	Non-canonical (house-cleaning) NTP pyrophosphatase, all-alpha NTP-PPase family	F;V	338	395	733
COG3676	Transposase and inactivated derivatives	X	340	690	1030

COG0581	ABC-type phosphate transport system, permease component	P	5723	6934	12657
COG0306	Phosphate/sulfate permease	P	6689	8726	15415
COG0446	NADPH-dependent 2,4-dienoyl-CoA reductase, sulfur reductase, or a related oxidoreductase	I	753	2327	3080
COG3531	Predicted protein-disulfide isomerase, contains CxxC motif	O	8	17	24
COG3716	Phosphotransferase system, mannose/fructose/N-acetylgalactosamine-specific IID component	G	709	1930	2639
COG3707	Two-component response regulator, AmiR/NasT family, consists of REC and RNA-binding antiterminator (ANTAR) domains	T;K	3875	3878	7753
COG0291	Ribosomal protein L35	J	1086	1146	2232
COG2804	Type II secretory pathway ATPase GspE/PulE or T4P pilus assembly pathway ATPase PilB	N;U;W	5466	8091	13557
COG3072	Adenylate cyclase, class I	T	0	6	6
COG1209	dTDP-glucose pyrophosphorylase	M	14156	15965	30120
COG2181	Nitrate reductase gamma subunit	C;P	49	259	308
COG3147	Cell division protein DedD (periplasmic protein involved in septation)	D	1052	1211	2263
COG1524	c-di-AMP phosphodiesterase AtaC or nucleotide pyrophosphatase, AlkP superfamily	T	4684	5394	10078
COG3620	Predicted transcriptional regulator, contains an XRE-type HTH domain (archaeal members contain CBS pair)	K	1917	2103	4020
COG3425	3-hydroxy-3-methylglutaryl CoA synthase	I	82	98	180

COG1556	L-lactate utilization protein LutC, contains LUD domain	C	90	150	240
COG2161	Antitoxin component YafN of the YafNO toxin-antitoxin module, PHD/YefM family	V	2828	2825	5652
COG4174	ABC-type microcin C transport system, permease component YejB	Q	19	102	122
COG0093	Ribosomal protein L14	J	1508	1949	3456
COG3408	Glycogen debranching enzyme (alpha-1,6-glucosidase)	G	25857	31879	57736
COG4339	Predicted metal-dependent phosphohydrolase, HD superfamily	R	1	10	11
COG4194	Uncharacterized membrane protein, DUF1648 family	S	101	376	476
COG3874	Uncharacterized spore protein YtfJ	S	2866	3268	6134
COG1584	Succinate-acetate transporter SatP	C	7	10	17
COG4978	GyrI-like small molecule binding domain	T	295	215	510
COG4166	ABC-type oligopeptide transport system, periplasmic component	E	10243	11488	21731
COG0481	Translation elongation factor EF-4, membrane-bound GTPase	J	9678	12768	22447
COG1672	Predicted ATPase, archaeal AAA+ ATPase superfamily	R	11661	13859	25520
COG4844	Uncharacterized conserved protein YuzB, UPF0349 family	S	4	1	5
COG1009	Membrane H+-translocase/NADH:ubiquinone oxidoreductase subunit 5 (chain L)/Multisubunit Na+/H+ antiporter, MnhA subunit	C	2889	3453	6342
COG0287	Prephenate dehydrogenase	E	6471	7922	14394
COG2896	GTP 3',8-cyclase (molybdenum cofactor biosynthesis protein MoaA)	H	3231	4596	7827

COG3180	Uncharacterized membrane protein AbrB, regulator of aidB expression	R	127	356	483
COG1655	Uncharacterized conserved protein, DUF2225 family	S	1626	1581	3208
COG3292	Periplasmic ligand-binding sensor domain	T	14608	19625	34233
COG3027	Cell division protein ZapA, inhibits GTPase activity of FtsZ	D	1454	1735	3189
COG3115	Cell division protein ZipA, interacts with	D	1800	1568	3368
COG5752	Signaling protein combining a Ser/Thr protein kinase domain and the GUN4/Ycf53 porphyrin-binding domain	T	14	5	19
COG3133	Outer membrane lipoprotein SlyB	M	18	512	530
COG0790	TPR repeat	R	14362	16286	30649
COG4651	Predicted Kef-type K+ transport protein, K+/H+ antiporter domain	P	450	1159	1610
COG1883	Na+-transporting oxaloacetate/methylmalonyl-CoA decarboxylase, beta subunit	C	8162	9956	18118
COG0697	Permease of the drug/metabolite transporter (DMT) superfamily	G;E;R	19909	25195	45104
COG5662	Transmembrane transcriptional regulator RsiW (anti-sigma-W factor)	K	1570	2114	3684
COG3258	Thiosulfate dehydrogenase TsdA, contains C-terminal cytochrome c domain	P	1440	1245	2685
COG3451	Type IV secretory pathway, VirB4 component	U	34588	40958	75546
COG0518	GMP synthase, glutamine amidotransferase domain	F	9744	12522	22265
COG3319	Thioesterase domain of type I polyketide synthase or non-ribosomal peptide	Q	3	1	5
COG2951	Membrane-bound lytic murein transglycosylase B	M	46	349	395

COG3820	Uncharacterized conserved protein, DUF1013 family	S	22	121	143
COG2068	CTP:molybdopterin cytidylyltransferase MocA	H	597	1703	2300
COG0238	Ribosomal protein S18	J	1435	1686	3122
COG4966	Type IV pilus assembly protein PilW	N;W	64	6	70
COG5310	Homospermidine synthase	Q	28	73	100
COG3169	Uncharacterized membrane protein, DMT/DUF486 family	S	1009	1181	2190
COG0268	Ribosomal protein S20	J	2566	2789	5356
COG4681	Uncharacterized conserved protein YaeQ, suppresses RfaH defect	S	0	1	1
COG1792	Cell shape-determining protein MreC	D;Z	8836	9161	17997
COG3969	Predicted phosphoadenosine phosphosulfate sulfurtransferase, contains C-terminal DUF3440 domain	R	1673	2133	3807
COG0110	Acetyltransferase, isoleucine patch superfamily	R	21297	24567	45864
COG3523	Type VI protein secretion system component VasK	U	25	16	42
COG0646	Methionine synthase I (cobalamin-dependent), methyltransferase domain	E	11217	12063	23279
COG0241	Histidinol phosphatase/D-glycero-mannoheptose bisphosphatephosphatase, HAD superfamily	E	2302	2869	5172
COG0614	ABC-type Fe3+-hydroxamate transport system, periplasmic component	P	12094	12979	25073
COG3427	Carbon monoxide dehydrogenase subunit CoxG	C	683	475	1158
COG0613	5'-3' exoribonuclease TrpH/YciV (RNase AM), contains PHP domain	F	10734	12681	23415

COG5377	Phage-related protein, predicted endonuclease	X	4985	5455	10440
COG4475	Uncharacterized conserved protein YwlG, UPF0340 family	S	1174	1446	2620
COG1813	Archaeal ribosome-binding protein aMBF1, putative translation factor, contains Zn-ribbon and HTH domains	J	733	873	1606
COG3907	Membrane-associated enzyme, PAP2 (acid phosphatase) superfamily	R	33	7	39
COG0616	Periplasmic serine protease, ClpP class	O	5532	4762	10293
COG2040	Homocysteine/selenocysteine methylase (S-methylmethionine-dependent)	E	172	351	523
COG1386	Chromosome segregation and condensation protein ScpB	K	4478	5418	9896
COG2875	Precorrin-4 methylase	H	3206	3732	6937
COG3066	DNA mismatch repair protein MutH	L	608	447	1055
COG3345	Alpha-galactosidase	G	19280	24210	43489
COG4888	Transcription elongation factor Elf1, contains Zn-ribbon domain	K	993	992	1985
COG2967	Uncharacterized conserved protein ApaG affecting Mg2+/Co2+ transport	P	53	182	235
COG3664	Beta-xylosidase	G	1416	2370	3785
COG5875	Uncharacterized sporulation protein YunB	D	2842	3383	6225
COG5419	Uncharacterized conserved protein, DUF2285 domain	S	4	6	9
COG0622	Predicted phosphodiesterase, calcineurin family	R	11159	12023	23181
COG4886	Leucine-rich repeat (LRR) protein	K	18890	16882	35772
COG2080	Aldehyde, CO, or xanthine dehydrogenase, Fe-S subunit, CoxS/CutS family	C	665	2908	3573
COG0233	Ribosome recycling factor	J	6132	7104	13236
COG3145	Alkylated DNA repair dioxygenase AlkB	L	1	183	185

COG4594	ABC-type Fe3+-citrate transport system, periplasmic component	P	2	0	2
COG3467	Nitroimidazole reductase NimA or a related FMN-containing flavoprotein, pyridoxamine 5'-phosphate oxidase superfamily	V	5171	5532	10703
COG5520	O-Glycosyl hydrolase	M	2906	4265	7171
COG3379	Predicted phosphohydrolase or phosphomutase, AlkP superfamily	R	293	1034	1327
COG3584	3D (Asp-Asp-Asp) domain, usually in lytic transglycosylases	S	1463	2583	4046
COG3587	Restriction endonuclease, type III RM system	V	8019	8979	16998
COG1835	Peptidoglycan/LPS O-acetylase OafA/YrhL, contains acyltransferase and SGNH-hydrolase domains	M	10537	10452	20988
COG1674	DNA segregation ATPase FtsK/SpoIIIE or related protein	D	28351	30986	59337
COG3178	Predicted phosphotransferase, aminoglycoside/choline kinase (APH/ChoK) family	R	320	842	1162
COG1571	tRNA(Ile2) C34 agmatinyltransferase TiaS	J	22	71	93
COG5720	Photosystem II cytochrome b559 alpha subunit, PsbE	C	5	4	9
COG4185	Predicted ABC-type ATPase or kinase	R	2294	2234	4528
COG5880	Spore coat protein CotE	D	364	389	754
COG3135	Predicted benzoate:H+ symporter BenE	Q	12	19	31
COG1578	House-cleaning carbohydrate phosphatase, DUF89 family	V	1113	967	2080
COG4215	ABC-type arginine transport system, permease component	E	0	2	2
COG0457	Tetratricopeptide (TPR) repeat	R	18369	22516	40885

COG3787	Uncharacterized conserved protein YhbP, UPF0306 family	S	161	62	223
COG3320	Thioester reductase domain of alpha aminoadipate reductase Lys2 and NRPSs	Q	4	69	73
COG0192	S-adenosylmethionine synthetase	H	11289	14533	25822
COG0510	Thiamine kinase or a related kinase	H	2938	2665	5603
COG1610	Uncharacterized conserved protein YqeY, may have tRNA amino acid amidase activity	R	3074	4477	7551
COG4177	ABC-type branched-chain amino acid transport system, permease component	E	4066	6182	10249
COG5921	Uncharacterized sporulation protein YqfX, contains DUF308 domain	D	2	0	2
COG1881	Uncharacterized conserved protein, phosphatidylethanolamine-binding protein (PEBP) family	R	615	1026	1641
COG3630	Na+-transporting oxaloacetate/methylmalonyl-CoA decarboxylase, gamma subunit	C	2249	2049	4298
COG1643	HrpA-like RNA helicase	J	1092	2982	4074
COG1725	DNA-binding transcriptional regulator YhcF, GntR family	K	9343	9655	18998
COG4394	Elongation factor P Arg32-rhamnosyltransferase EarP	J	1	189	189
COG4502	5'(3')-deoxyribonucleotidase	F	617	1534	2151
COG1181	D-alanine-D-alanine ligase or related ATP-grasp enzyme	M;R	15129	17074	32203
COG0373	Glutamyl-tRNA reductase	H	983	2083	3066
COG0316	Fe-S cluster assembly iron-binding protein IscA	O	11	156	167
COG4387	Mu-like prophage protein gp36	X	210	551	761
COG2910	Putative NADH-flavin reductase	R	312	336	648

COG0580	Glycerol uptake facilitator or related aquaporin (Major Intrinsic protein Family)	G	2812	2449	5261
COG3111	Predicted periplasmic protein YdeI with OB-fold, BOF family	S	21	248	269
COG2268	Uncharacterized membrane protein YqiK, contains Band7/PHB/SPFH domain	S	3424	4708	8132
COG3662	Uncharacterized conserved protein, DUF2236 family	S	1	4	6
COG1496	Copper oxidase (laccase) domain	P	4995	6346	11341
COG3436	Transposase	X	36980	57460	94440
COG1776	Phosphoaspartate phosphatase CheC, specific for CheY-P	T	6274	5388	11662
COG2604	Uncharacterized conserved protein	S	2683	2422	5106
COG3343	DNA-directed RNA polymerase, delta subunit	K	286	199	484
COG0721	Asp-tRNAAsn/Glu-tRNAGln amidotransferase C subunit	J	1306	1600	2907
COG4861	Uncharacterized conserved protein	S	161	638	800
COG1297	Predicted oligopeptide transporter, OPT family	R	4433	4357	8791
COG3503	Uncharacterized membrane protein, DUF1624 family	S	3310	2752	6062
COG3269	Predicted RNA-binding protein, contains TRAM domain	R	1	1	2
COG3435	Gentisate 1,2-dioxygenase	Q	4	13	17
COG2440	Ferredoxin-like protein FixX	C	20	63	83
COG0044	Dihydroorotase or related cyclic amidohydrolase	F	10952	13720	24673
COG1328	Anaerobic ribonucleoside-triphosphate reductase	F	18940	23476	42415
COG4463	Transcriptional regulator CtsR	K	940	1113	2053
COG1067	Predicted ATP-dependent protease	O	906	2047	2953

COG1088	dTDP-D-glucose 4,6-dehydratase	M	8539	8070	16609
COG3210	Large exoprotein involved in heme utilization or adhesion	U	6453	9404	15857
COG3629	DNA-binding transcriptional regulator DnrI/AfsR/EmbR, SARP family, contains BTAD domain	K	1143	3093	4236
COG0470	DNA polymerase III, delta prime subunit	L	687	1475	2162
COG1806	Regulator of PEP synthase PpsR, kinase-PPPase family (combines ADP:protein kinase and phosphorylase activities)	T	145	316	461
COG0830	Urease accessory protein UreF	O	1445	1265	2710
COG1895	HEPN domain protein, predicted toxin of MNT-HEPN system	V	1842	1607	3450
COG4598	ABC-type histidine transport system, ATPase component	E	19	51	71
COG0330	Regulator of protease activity HflC, stomatin/prohibitin superfamily	O	18225	22211	40436
COG5828	Stage III sporulation protein SpoIIIAG, component of the engulfment complex	D	3487	3822	7309
COG4150	ABC-type sulfate transport system, periplasmic component	P	0	3	3
COG3972	Superfamily I DNA and RNA helicases	L	829	1626	2456
COG4121	tRNA U34 5-methylaminomethyl-2-thiouridine-forming methyltransferase	J	153	312	466
COG0584	Glycerophosphoryl diester phosphodiesterase	I	9776	11458	21234
COG0439	Biotin carboxylase	I	4777	5752	10529
COG2715	Spore maturation protein SpmA (function unknown)	R	4684	5309	9993
COG5888	Spore coat protein CotY/CotZ (outer spore coat)	D	44	61	105
COG0557	Exoribonuclease R	K	12623	14439	27062

COG4944	Uncharacterized conserved protein	S	1	3	4
COG4795	Type II secretory pathway, PulJ/GspJ component	U	99	1	100
COG3024	Endogenous inhibitor of DNA gyrase, YacG/DUF329 family	L	1	44	45
COG1823	L-cystine transporter TcyP, sodium:dicarboxylate symporter family	E	14	53	67
COG1357	Uncharacterized conserved protein YjbI, contains pentapeptide repeats	S	571	1145	1716
COG3420	Nitrous oxide reductase accessory protein NosD, contains tandem CASH domains	P	1559	1984	3543
COG1437	Adenylate cyclase class IV, CYTH domain (includes archaeal enzymes of unknown function)	T;R	476	892	1368
COG0434	Membrane biogenesis protein, BtpA/SgcQ family	M	381	675	1056
COG5001	Cyclic di-GMP metabolism protein, combines GGDEF and EAL domains with a 6TM membrane domain	T	5412	3911	9323
COG0437	Fe-S-cluster-containing dehydrogenase component (DMSO reductase)	C	1721	4183	5904
COG1657	Terpene cyclase SqhC	I	3	612	615
COG4748	Uncharacterized conserved protein, contains restriction enzyme R protein N terminal (HSDR_N) domain	S	1478	1372	2849
COG3013	Uncharacterized conserved protein YfbU, UPF0304 family	S	0	11	11
COG1741	Redox-sensitive bicupin YhaK, pirin superfamily	R	404	327	731
COG3948	Phage-related baseplate assembly protein	X	2237	2058	4295
COG0647	Ribonucleotide monophosphatase NagD, HAD superfamily	F	2251	3309	5561

COG4876	Uncharacterized conserved protein YdaT	S	3	2	5
COG1106	ATPase/GTPase, AAA15 family	R	8917	9625	18542
COG2608	Copper chaperone CopZ	P	2970	2915	5885
COG5317	Uncharacterized conserved protein, DUF1465 domain	S	0	2	2
COG5319	Uncharacterized conserved protein, DUF1178 domain	S	2	91	93
COG0368	Cobalamin synthase CobS (adenosylcobinamide-GDP ribazoletransferase)	H	3247	3779	7026
COG2925	Exonuclease I (degrades ssDNA)	L	2	238	240
COG0088	Ribosomal protein L4	J	7573	8618	16191
COG0052	Ribosomal protein S2	J	6914	8120	15034
COG2893	Phosphotransferase system, mannose/fructose-specific component IIA	G	589	1464	2053
COG0082	Chorismate synthase	E	9722	10552	20274
COG3338	Carbonic anhydrase	P	0	1	1
COG1999	Cytochrome oxidase Cu insertion factor, SCO1/SenC/PrrC family	O	113	178	291
COG4198	Uncharacterized conserved protein, DUF1015 family	S	8750	9705	18455
COG3506	Regulation of enolase protein 1 (function unknown), concanavalin A-like superfamily	S	3392	3810	7203
COG0732	Restriction endonuclease S subunit	V	29223	30585	59808
COG2877	3-deoxy-D-manno-octulosonic acid (KDO) 8-phosphate synthase	M	1993	2527	4520
COG4826	Serine protease inhibitor	O	9154	7431	16585
COG3453	Predicted phosphohydrolase, protein tyrosine phosphatase (PTP) superfamily, DUF442 family	R	3	9	12
COG1227	Inorganic pyrophosphatase/exopolyphosphatase	C	3481	5396	8877

COG3093	Plasmid maintenance system antidote protein VapI, contains XRE-type HTH domain	V	3036	3243	6278
COG1117	ABC-type phosphate transport system, ATPase component	P	4778	6268	11045
COG3186	Phenylalanine-4-hydroxylase	E	2	2	4
COG4555	ABC-type Na+ transport system, ATPase component NatA	C;P	346	384	730
COG0023	Translation initiation factor 1 (eIF-1/SUI1)	J	1024	960	1984
COG0167	Dihydroorotate dehydrogenase	F	10641	11514	22155
COG4942	Septal ring factor EnvC, activator of murein hydrolases AmiA and AmiB	D	14789	15086	29876
COG1689	Class II terpene cyclase family protein AF1543	R	130	166	297
COG3182	PepSY-associated TM region	S	163	366	529
COG4824	Phage-related holin (Lysis protein)	X	3009	3449	6458
COG1235	Phosphoribosyl 1,2-cyclic phosphate phosphodiesterase	P	12112	15178	27290
COG5403	Uncharacterized conserved protein, DUF937 domain	S	37	32	69
COG2845	Uncharacterized conserved protein, DUF459 domain	S	12	0	12
COG2155	Uncharacterized membrane protein YuzA, DUF378 family	S	1113	1094	2207
COG1020	EntF, seryl-AMP synthase component of non-ribosomal peptide synthetase	Q	9382	10595	19978
COG5750	Chlorophyll biosynthesis regulator GUN4/Ycf53 domain	T	0	5	5
COG5658	Bacterial immunity/signal transduction membrane protein SdpI, SdpI/YbgB/DUF1648 family	V	2646	2143	4788
COG2116	Formate/nitrite transporter FocA, FNT	P	2998	3793	6791

COG5819	Stage II sporulation protein SpoIIP, required for dissolution of the septal cell	D	7248	7376	14625
COG3255	Putative sterol carrier protein, contains SCP2 domain	I	1011	620	1631
COG2261	Uncharacterized membrane protein YeaQ/YmgE, transglycosylase-associated protein family	R	610	546	1156
COG0540	Aspartate carbamoyltransferase, catalytic subunit	F	7382	8716	16099
COG0842	ABC-type multidrug transport system, permease component	V	15635	16593	32228
COG3037	Ascorbate-specific PTS system IIC component UlaA	G	353	986	1339
COG0358	DNA primase (bacterial type)	L	42493	43183	85676
COG1926	Predicted phosphoribosyltransferase	R	19	1	20
COG3860	Uncharacterized conserved protein, DUF2087 family	S	36	61	97
COG2212	Multisubunit Na+/H+ antiporter, MnhF subunit	P	706	705	1411
COG0447	1,4-Dihydroxy-2-naphthoyl-CoA synthase	H	758	1566	2324
COG2220	L-ascorbate lactonase UlaG, metallo-beta-lactamase superfamily	G	5608	7897	13505
COG1826	Twin-arginine protein secretion pathway components TatA and TatB	U	904	1267	2171
COG1066	DNA repair protein RadA/Sms, contains AAA+ ATPase domain	L	14151	13664	27816
COG1192	ParA-like ATPase involved in chromosome/plasmid partitioning or cellulose biosynthesis protein BcsQ	D;N	26926	28280	55206
COG3043	Nitrate reductase cytochrome c-type subunit NapB	C;P	12	61	73
COG4099	Predicted peptidase	R	1612	3201	4812

COG3618	Predicted metal-dependent hydrolase, TIM-barrel fold	R	318	426	745
COG2005	DNA-binding transcriptional regulator ModE (molybdenum-dependent)	K	224	894	1118
COG5362	Phage terminase large subunit	X	2781	2010	4791
COG5579	Uncharacterized conserved protein, DUF1810 family	S	707	804	1511
COG5263	Glucan-binding domain (YG repeat)	G	15102	18832	33933
COG4034	Uncharacterized conserved protein, DUF1152 domain	S	1	1	2
COG1343	CRISPR/Cas system-associated endoribonuclease Cas2	V	1424	1588	3012
COG3167	Type IV pilus assembly protein PilO	N;W	226	234	459
COG1157	Flagellar biosynthesis/type III secretory pathway ATPase FliI	N;U	2966	2918	5885
COG1716	Forkhead associated (FHA) domain, binds pSer, pThr, pTyr	T	12944	15742	28686
COG0281	Malic enzyme	C	6918	8765	15682
COG0169	Shikimate 5-dehydrogenase	E	8754	10590	19345
COG1028	NAD(P)-dependent dehydrogenase, short-chain alcohol dehydrogenase family	I	37174	44064	81237
COG0413	Ketopantoate hydroxymethyltransferase	H	3360	3775	7135
COG2029	Uncharacterized conserved protein, DUF366 domain	S	8	82	90
COG2084	3-hydroxyisobutyrate dehydrogenase or related beta-hydroxyacid dehydrogenase	I	847	2717	3565
COG3684	Tagatose-1,6-bisphosphate/sulfofructosephosphate	G;P	163	110	273
COG2508	DNA-binding transcriptional regulator, PucR/PutR family	K	1380	3597	4977
COG3515	Type VI protein secretion system component TssA1/VasJ/EvfE, contains	U	13	11	24

COG4479	Uncharacterized conserved protein YozE, UPF0346 family	S	12	17	29
COG2194	Phosphoethanolamine transferase for periplasmic glucans OpgE, AlkP superfamily	M	3817	5092	8910
COG1566	Multidrug resistance efflux pump EmrA	V	9678	11845	21523
COG2272	Carboxylesterase type B	I	2727	3745	6473
COG4422	Bacteriophage protein gp37	X	3892	3752	7643
COG3546	Mn-containing catalase (includes spore coat protein CotJC)	P	5475	5727	11202
COG2013	AIM24 protein, required for mitochondrial respiration	C	3240	2756	5996
COG2200	EAL domain, c-di-GMP-specific phosphodiesterase class I (or its enzymatically inactive variant)	T	18663	15240	33902
COG5551	CRISPR/Cas system endoribonuclease Cas6, RAMP superfamily	V	422	740	1162
COG4120	ABC-type uncharacterized transport system, permease component	R	5867	5788	11655
COG1090	NAD dependent epimerase/dehydratase family enzyme	R	64	548	612
COG1201	Lhr-like helicase	L	2889	2116	5005
COG1481	DNA-binding transcriptional regulator WhiA, involved in cell division	K	7652	8308	15960
COG3572	Gamma-glutamylcysteine synthetase	H	219	441	660
COG3418	Flagellar biosynthesis/type III secretory pathway chaperone FlgN	N;U	253	168	421
COG1959	DNA-binding transcriptional regulator, IscR family	K	5295	6269	11564
COG4464	Tyrosine-protein phosphatase YwqE	T	5863	7544	13407
COG3103	Uncharacterized conserved protein YgiM, contains N-terminal SH3 domain, DUF1202 family	R	7288	8384	15672

COG0663	Carbonic anhydrase or acetyltransferase, isoleucine patch superfamily	R	2487	2932	5419
COG5577	Spore coat protein CotF	M	331	676	1007
COG3464	Transposase	X	26436	25101	51537
COG3331	Penicillin-binding protein-related factor A, putative recombinase	R	1940	2228	4169
COG0559	Branched-chain amino acid ABC-type transport system, permease component	E	3660	5700	9360
COG1202	Superfamily II helicase, archaea-specific	L	2	25	27
COG5557	Ni/Fe-hydrogenase 2 integral membrane subunit HybB	C	354	1026	1379
COG2707	Uncharacterized membrane protein, DUF441 family	S	35	91	126
COG1143	Formate hydrogenlyase subunit 6/NADH:ubiquinone oxidoreductase 23 kD subunit (chain I)	C	8598	10345	18943
COG5653	Acetyltransferase involved in cellulose biosynthesis, CelD/BcsL family	N	107	118	225
COG3601	Riboflavin transporter FmnP	H	5045	5278	10323
COG0592	DNA polymerase III sliding clamp (beta) subunit, PCNA homolog	L	13284	14210	27494
COG1197	Transcription-repair coupling factor (superfamily II helicase)	L;K	21533	26067	47600
COG1618	Nucleoside-triphosphatase THEP1	F	1060	1468	2527
COG3231	Aminoglycoside phosphotransferase	J	761	1071	1832
COG1218	3'-Phosphoadenosine 5'-phosphosulfate (PAPS) 3'-phosphatase	P	1958	1719	3678
COG4214	ABC-type xylose transport system, permease component	G	863	1216	2079
COG2038	NaMN:DMB phosphoribosyltransferase	H	3227	4499	7725
COG3386	Sugar lactone lactonase YvrE	G	4508	4007	8514

COG0799	Ribosomal silencing factor RsfS, regulates association of 30S and 50S subunits	J	3568	3880	7447
COG0204	1-acyl-sn-glycerol-3-phosphate acyltransferase	I	18440	22519	40958
COG0681	Signal peptidase I	U	14330	17474	31804
COG3669	Alpha-L-fucosidase	G	9074	13700	22773
COG5587	Uncharacterized protein, contains DUF2461 domain	S	3494	3667	7161
COG3206	Exopolysaccharide export protein/domain GumC/Wzc1	M	825	1960	2784
COG0164	Ribonuclease HII	L	7613	8596	16209
COG1168	Bifunctional PLP-dependent enzyme with beta-cystathionase and maltose regulon repressor activities	E;R	6392	10067	16459
COG2335	Uncaracterized surface protein containing fasciclin (FAS1) repeats	R	446	757	1203
COG0673	Predicted dehydrogenase	R	24106	31701	55807
COG4460	Uncharacterized conserved protein, DUF4440 domain	S	0	0	0
COG3617	Prophage antirepressor	X	10419	14317	24736
COG2151	Metal-sulfur cluster biosynthetic enzyme	O	1035	1306	2341
COG0537	Purine nucleoside phosphoramidase/Ap4A hydrolase, histidine triade (HIT) family	F;R	7007	7406	14414
COG1040	DNA utilization protein ComFC/GntX, contains phosphoribosyltransferase domain	R	8843	9720	18563
COG1459	Type II secretory pathway, component PulF	N;U;W	7058	8511	15569
COG1220	ATP-dependent protease HslVU (ClpYQ), ATPase subunit HslU	O	780	1585	2364
COG1298	Flagellar biosynthesis protein FlhA	N	4617	4180	8798
COG3540	Phosphodiesterase/alkaline phosphatase D	P	34	287	321
COG3153	Predicted N-acetyltransferase YhbS	R	8543	8124	16667
COG3687	Predicted metal-dependent hydrolase	R	2	4	6

COG3935	DNA replication protein DnaD	L	13907	15192	29099
COG2980	Outer membrane lipoprotein LptE/RlpB (LPS assembly)	M	0	95	95
COG3559	Putative exporter of oligosaccharides and polyketide antibiotics DrrB/NodJ, ABC-2 family	U	219	358	576
COG3401	Fibronectin type 3 domain	R	6795	5880	12674
COG5595	Predicted nucleic acid-binding protein, contains Zn-ribbon domain	R	1	82	83
COG0590	tRNA(Arg) A34 adenosine deaminase TadA	J	7012	8380	15392
COG4330	Uncharacterized membrane protein, DUF1361 domain	S	111	81	192
COG1338	Flagellar biosynthesis protein FliP	N	3345	2638	5983
COG3746	Phosphate-selective porin	P	2975	2536	5511
COG3461	Uncharacterized conserved protein	S	2	14	16
COG3736	Type IV secretory pathway, component VirB8	U	25	245	270
COG0711	FoF1-type ATP synthase, membrane subunit b or b'	C	4856	5185	10041
COG4118	Antitoxin component of toxin-antitoxin stability system, DNA-binding transcriptional repressor	V	666	506	1172
COG2602	Beta-lactamase class D	V	303	421	723
COG1146	NAD-dependent dihydropyrimidine dehydrogenase, PreA subunit	F	70	381	451
COG1406	Chemotaxis protein CheX, a CheY~P-specific phosphatase	N	1027	649	1676
COG3575	Uncharacterized conserved protein	S	136	162	299
COG3516	Predicted component TssA of the type VI protein secretion system	U	10	3	12
COG1585	Membrane protein implicated in regulation of membrane protease activity	O	3070	3816	6886

COG1738	Queuosine precursor transporter YhhQ, DUF165 family	J	2130	2036	4166
COG3312	FoF1-type ATP synthase accessory protein AtpI	C	0	2	2
COG0123	Acetoin utilization deacetylase AcuC or a related deacetylase	Q	48	330	378
COG2839	Uncharacterized conserved protein YqgC, DUF456 family	S	709	1121	1830
COG3417	Outer membrane lipoprotein LpoB, binds and activates PBP1b	M	86	71	157
COG2120	N-acetylglucosaminyl deacetylase, LmbE family	G	4520	4397	8916
COG4533	DNA-binding transcriptional regulator SgrR of sgrS sRNA, contains a MarR-type HTH domain and a periplasmic-type solute-binding domain	K	0	5	5
COG5649	Uncharacterized conserved protein, DUF1801 domain	S	2	4	6
COG5889	Spore coat protein GerQ/YwdL (inner spore coat)	D	128	171	298
COG4774	Outer membrane receptor for monomeric catechols	P	74	837	912
COG4290	Guanyl-specific ribonuclease Sa	F	1948	1954	3901
COG4975	Glucose uptake protein GlcU	G	458	556	1014
COG3498	Phage tail tube protein FII	X	742	698	1440
COG5622	Protein required for attachment to host cells	M	2	6	8
COG1447	Phosphotransferase system cellobiose-specific component IIA	G	303	640	943
COG1811	Uncharacterized membrane protein YqgA, affects biofilm formation	S	1903	2133	4036
COG4452	Inner membrane protein CreD involved in colicin E2 resistance	V	280	366	646

COG2812	DNA polymerase III, gamma/tau subunits	L	25617	27631	53248
COG4122	tRNA 5-hydroxyU34 O-methylase TrmR/YrrM	J	5207	6059	11267
COG2319	WD40 repeat	R	1826	1734	3560
COG0148	Enolase	G	8024	9222	17246
COG2234	Zn-dependent amino- or carboxypeptidase, M28 family	O;E	10321	14577	24898
COG4529	Uncharacterized NAD(P)/FAD-binding protein YdhS	R	4	17	20
COG2271	Sugar phosphate permease	G	6802	9482	16284
COG0460	Homoserine dehydrogenase	E	8732	10239	18971
COG4467	Regulator of replication initiation timing	L	58	71	130
COG1768	Predicted phosphohydrolase, DR1119 family, metallophosphatase superfamily	R	3529	4028	7557
COG3694	ABC-type uncharacterized transport system, permease component	R	4311	3143	7454
COG4067	Uncharacterized conserved protein	S	3	64	68
COG3881	Uncharacterized conserved protein YrrD, contains PRC-barrel domain	S	25	158	183
COG2035	Uncharacterized membrane protein, DUF368 family	S	5109	4444	9553
COG4801	Predicted acyltransferase, contains DUF342 domain	R	144	99	243
COG4908	Uncharacterized conserved protein, contains a NRPS condensation (elongation) domain	R	3515	4549	8064
COG1363	Putative aminopeptidase FrvX	E;G	10629	13117	23746
COG2267	Lysophospholipase, alpha-beta hydrolase superfamily	I	12249	13712	25961
COG4930	Predicted ATP-dependent Lon-type protease	O	3108	2569	5676

COG3715	Phosphotransferase system, mannose/fructose/N-acetylgalactosamine-specific IIC component	G	680	2000	2679
COG2747	Negative regulator of flagellin synthesis (anti-sigma28 factor)	K;N	570	495	1064
COG5314	Conjugal transfer/entry exclusion protein	X	26	112	138
COG2032	Cu/Zn superoxide dismutase	P	2513	2457	4970
COG0541	Signal recognition particle GTPase	U	10103	11499	21602
COG0183	Acetyl-CoA acetyltransferase	I	1997	5024	7021
COG3477	Uncharacterized membrane protein YagU, involved in acid resistance, DUF1440 family	S	0	1	1
COG4894	Putative phospholipid scramblase YxjI, Tubby2 superfamily	I	1044	1189	2233
COG2887	RecB family exonuclease	L	5709	7257	12966
COG1803	Methylglyoxal synthase	G	2205	2572	4777
COG2908	UDP-2,3-diacylglucosamine pyrophosphatase LpxH	M	3532	3896	7429
COG5507	Uncharacterized conserved protein YbaA, DUF1428 family	S	3	3	6
COG0642	Signal transduction histidine kinase	T	155513	161261	316774
COG1968	Undecaprenyl pyrophosphate phosphatase	I	9883	11306	21189
COG1210	UTP-glucose-1-phosphate	M	2956	3438	6395
COG1311	Archaeal DNA polymerase II, small subunit/DNA polymerase delta, subunit B	L	83	67	150
COG1583	CRISPR/Cas system endoribonuclease Cas6, RAMP superfamily	V	433	383	816
COG2820	Uridine phosphorylase	F	6956	8475	15431
COG2108	Uncharacterized radical SAM domain-containing protein	S	363	411	774
COG1002	Type II restriction/modification system, endonuclease and methylase domains	V	8734	12024	20758

COG1774	Cell fate regulator YaaT, PSP1 superfamily (controls sporulation, competence, biofilm development)	T	11842	12063	23905
COG4623	Membrane-bound lytic murein transglycosylase MltF	M;T	5338	4661	9999
COG0392	Predicted membrane flippase AglD2/YbhN, UPF0104 family	M	5407	5982	11390
COG2958	Uncharacterized conserved protein	S	515	1026	1541
COG1398	Fatty-acid desaturase	I	4	8	12
COG1865	Adenosylcobinamide amidohydrolase	H	11	8	19
COG2716	Glycine cleavage system regulator GcvR	E	11	138	150
COG3273	Uncharacterized protein, contains PhoU and TrkA_C domains	R	15	7	22
COG1195	Recombinational DNA repair ATPase RecF	L	13115	14410	27526
COG5345	Uncharacterized conserved protein, DUF2333 domain	S	16	108	125
COG4097	Predicted ferric reductase	P	6	5	10
COG1553	Sulfur relay (sulfurtransferase) complex TusBCD TusD component, DsrE family	P	2	4	7
COG3883	Uncharacterized N-terminal coiled-coil domain of peptidoglycan hydrolase CwlO	S	1301	1903	3205
COG0005	Purine nucleoside phosphorylase	F	6087	6732	12818
COG4820	Ethanolamine utilization protein EutJ, possible chaperonin	E	47	239	286
COG1852	Predicted redox protein with CxxCxxC motif, DUF116 family	R	20	98	118
COG1480	Cyclic di-AMP-specific phosphodiesterase PgpH, HD superfamily	T	4078	5231	9310
COG4301	Uncharacterized protein, contains predicted SAM-dependent methyltransferase domain	R	2	6	8

COG2002	Bifunctional DNA-binding transcriptional regulator of stationary/sporulation/toxin gene expression and antitoxin component of the YhaV-PrlF toxin-antitoxin module	K;V	2003	2286	4289
COG3592	Uncharacterized Fe-S cluster protein YjdI	S	1	2	3
COG3239	Fatty acid desaturase	I	1	5	6
COG4129	Uncharacterized membrane protein YgaE, UPF0421/DUF939 family	S	1619	2352	3971
COG1137	ABC-type lipopolysaccharide export system, ATPase component	M	1844	2666	4510
COG0071	Small heat shock protein IbpA, HSP20 family	O	3612	4159	7771
COG1171	Threonine deaminase	E	5275	7189	12465
COG1868	Flagellar motor switch protein FliM	N	3014	2738	5752
COG0030	16S rRNA A1518 and A1519 N6-dimethyltransferase RsmA/KsgA/DIM1 (may also have DNA glycosylase/AP lyase activity)	J	13994	15491	29486
COG5255	Uncharacterized conserved protein	S	8	1	9
COG1754	Uncharacterized C-terminal domain of topoisomerase IA	S	297	118	415
COG3562	Capsule polysaccharide modification protein KpsS	M	30	119	149
COG1561	Uncharacterized stationary-phase protein YicC, UPF0701 family	S	8708	10000	18708
COG3448	CBS-domain-containing membrane protein	T	681	581	1262
COG0396	Fe-S cluster assembly ATPase SufC	O	5974	7277	13251
COG1015	Phosphopentomutase	G	4615	5933	10548
COG3457	Predicted amino acid racemase	E	217	612	829
COG5383	Uncharacterized metalloenzyme YdcJ, glyoxalase superfamily	R	0	3	3
COG1573	Uracil-DNA glycosylase	L	3033	5640	8672

COG1604	CRISPR/Cas system CMR subunit Cmr6, Cas7 group, RAMP superfamily	V	91	25	115
COG3577	Predicted aspartyl protease	R	438	710	1148
COG2927	DNA polymerase III, chi subunit	L	7	132	139
COG3731	Phosphotransferase system sorbitol-specific IIA component	G	196	221	417
COG4006	CRISPR-Cas system-associated protein Csm6, contains CARF domain	V	18	22	40
COG0199	Ribosomal protein S14	J	814	948	1763
COG1381	Recombinational DNA repair protein RecO (RecF pathway)	L	8462	9100	17562
COG3859	Thiamine transporter ThiT	H	3992	4075	8067
COG3284	Transcriptional regulator DhaR of acetoin/glycerol metabolism	K	83	217	299
COG2165	Type II secretory pathway, pseudopilin	N;U;W	6220	8540	14759
COG0530	Ca2+/Na+ antiporter	P	9870	10538	20408
COG0628	Predicted PurR-regulated permease PerM	R	23390	26528	49918
COG1416	Intracellular sulfur oxidation protein, DsrE/DsrF family	P	10	13	23
COG4254	Uncharacterized peptidoglycan binding protein, contains LysM and FecR domains	R	94	209	303
COG3916	N-acyl-homoserine lactone synthase LasI (autoinducer biosynthesis)	T	4	6	10
COG3121	P pilus assembly protein, chaperone PapD	W	2	24	26
COG0253	Diaminopimelate epimerase	E	9663	12452	22114
COG3086	RseC, positive regulator of sigma E activity	T	1233	1163	2396
COG2350	YciI superfamily enzyme, includes 5-CHQ dehydrochlorinase, contains active-site pHis	Q;R	36	13	49
COG1830	Fructose-bisphosphate aldolase class Ia, DhnA family	G	410	661	1071
COG0499	S-adenosylhomocysteine hydrolase	H	4009	5392	9401

COG0156	7-keto-8-aminopelargonate synthetase or related enzyme	H	6478	8224	14702
COG3034	Murein L,D-transpeptidase YafK	M	322	699	1021
COG2990	Outer membrane protein VirK/YbjX involved in antimicrobial peptide resistance, DUF535 family	S	1	92	93
COG4136	ABC-type uncharacterized transport system YnjBCD, ATPase component	R	0	1	1
COG3015	Uncharacterized lipoprotein NlpE involved in copper resistance	M;V	274	159	432
COG3045	Periplasmic catabolite regulation protein CreA (function unknown)	T	0	102	102
COG1508	DNA-directed RNA polymerase specialized sigma subunit, sigma54 homolog	K	3377	5158	8535
COG1514	RNA 2',3'-cyclic phosphodiesterase (2'-5' RNA ligase)	J	724	928	1652
COG1324	Divalent cation tolerance protein CutA	P	60	63	122
COG3010	Putative N-acetylmannosamine-6-phosphate epimerase	G	762	1331	2093
COG1246	N-acetylglutamate synthase or related acetyltransferase, GNAT family	E	4553	4498	9052
COG1535	Isochorismate hydrolase	Q	1	11	12
COG1478	F420-0:Gamma-glutamyl ligase (F420 biosynthesis)	H	1993	1840	3833
COG2515	1-aminocyclopropane-1-carboxylate deaminase/D-cysteine desulfhydrase, PLP-dependent ACC family	E	311	730	1041
COG4696	NTP pyrophosphatase, MazG superfamily	F	280	202	482
COG5343	Anti-sigma-K factor RskA	T	0	0	1
COG4447	Uncharacterized conserved protein related to plant photosystem II stability/assembly	R	5719	4947	10666
COG5207	Uncharacterized Zn-finger protein, UBP-	R	1	1	2

COG1076	DnaJ domain-containing protein	O	16	131	148
COG2139	Ribosomal protein L21E	J	2	3	5
COG4865	Glutamate mutase epsilon subunit	E	239	502	740
COG1842	Phage shock protein A	K;T	1235	1195	2429
COG3748	Uncharacterized membrane protein	S	0	0	0
COG1380	Putative effector of murein hydrolase LrgA, UPF0299 family	R	697	1172	1869
COG1159	GTPase Era, involved in 16S rRNA processing	J	13400	14562	27961
COG3076	Regulator of RNase E activity RraB	J	47	63	110
COG4814	Uncharacterized conserved protein with an alpha/beta hydrolase fold	S	279	428	707
COG4768	Uncharacterized conserved protein YoxC, contains an MCP-like domain	S	67	87	155
COG0039	Malate/lactate dehydrogenase	C	7296	9398	16694
COG1333	Cytochrome c biogenesis protein ResB	C;O	285	842	1128
COG0388	Omega-amidase YafV/Nit2, hydrolyzes alpha-ketoglutaramate	C	9579	10085	19664
COG0097	Ribosomal protein L6P/L9E	J	6259	6989	13249
COG3877	Predicted DNA-binding transcriptional regulator with XRE-family HTH domain, DUF2089 family	K	399	531	930
COG2914	Putative antitoxin component PasI (RatB) of the RatAB toxin-antitoxin module, ubiquitin-RnfH superfamily	V	0	42	42
COG1215	Glycosyltransferase, catalytic subunit of cellulose synthase and poly-beta-1,6-N-acetylglucosamine synthase	N	16777	19685	36463
COG0553	Superfamily II DNA or RNA helicase, SNF2 family	K;L	20688	26412	47100
COG4590	ABC-type uncharacterized transport system, permease component	R	2	669	671

COG1639	HD-like signal output (HDOD) domain, no enzymatic activity	T	19	48	67
COG4158	Predicted ABC-type sugar transport system, permease component	R	2	6	8
COG4026	Uncharacterized conserved protein, contains TOPRIM domain, potential nuclease	R	0	2	2
COG4148	ABC-type molybdate transport system, ATPase component ModC	P	27	216	243
COG3933	Transcriptional regulatory protein LevR, contains PRD, AAA+ and EIIA domains	K	0	1	1
COG0653	Preprotein translocase subunit SecA (ATPase, RNA helicase)	U	18466	21743	40209
COG4581	Superfamily II RNA helicase	L	53	350	403
COG3071	Uncharacterized protein HemY, contains HemY_N domain and TPR repeats (unrelated to protoporphyrinogen oxidase HemY)	S	1	1	1
COG5346	Uncharacterized membrane protein	S	39	45	84
COG0445	tRNA U34 5-carboxymethylaminomethyl modifying enzyme MnmG/GidA	J	13967	16926	30893
COG2815	PASTA domain, binds beta-lactams	M	12667	11477	24143
COG1309	DNA-binding protein, AcrR family, includes nucleoid occlusion protein SlmA	K	46794	48722	95516
COG3004	Na+/H+ antiporter NhaA	C;P	2999	2463	5462
COG1767	Triphosphoribosyl-dephospho-CoA synthetase	H	712	880	1592
COG3450	Predicted enzyme of the cupin superfamily	R	4	10	15
COG3547	Transposase	X	57038	79767	136805
COG5903	Spore germination receptor GerABC, GerC subunit	D	1821	2659	4479
COG0061	NAD kinase	H	8807	9820	18627

COG1288	Predicted membrane transporter YfcC, affects glyoxylate shunt	R	1182	2383	3565
COG3763	Uncharacterized conserved protein YneF, UPF0154 family	S	81	78	159
COG3675	Predicted lipase	I	8	102	110
COG2878	Na+-translocating ferredoxin:NAD+ oxidoreductase RNF, RnfB subunit	C	3903	3889	7792
COG1339	Archaeal CTP-dependent riboflavin kinase	H	25	25	50
COG0835	Chemotaxis signal transduction protein CheW	T	3281	2742	6023
COG0154	Asp-tRNAAsn/Glu-tRNAGln amidotransferase A subunit or related amidase	J	7162	10410	17572
COG0767	Permease subunit MlaE of the ABC-type intermembrane phospholipid transporter Mla	M	2046	3123	5169
COG5716	Photosystem II reaction center D1, PsbA	C	24	16	41
COG4785	Lipoprotein NlpI, contains TPR repeats	M	834	669	1503
COG3069	C4-dicarboxylate transporter DcuC	C	81	2200	2281
COG5628	Predicted acetyltransferase	R	650	561	1211
COG1346	Putative effector of murein hydrolase	M	1353	2265	3618
COG0046	Phosphoribosylformylglycinamidine (FGAM) synthase, synthetase domain	F	16383	19950	36333
COG1012	Acyl-CoA reductase or other NAD-dependent aldehyde dehydrogenase	I	9580	14107	23687
COG3779	Uncharacterized conserved protein YegJ, DUF2314 family	S	493	598	1092
COG4365	Putative cysteine ligase BshC/YllA (bacillithiol biosynthesis)	Q	0	1	1
COG0780	NADPH-dependent 7-cyano-7-deazaguanine reductase QueF, C-terminal domain, T-fold superfamily	J	1394	1473	2867
COG0163	Flavin prenyltransferase UbiX	H	287	425	712

COG0240	Glycerol-3-phosphate dehydrogenase	C	10275	12404	22678
COG1825	Ribosomal protein L25 (general stress protein Ctc)	J	2467	2656	5124
COG0665	Glycine/D-amino acid oxidase (deaminating)	E	7471	9449	16920
COG3209	Uncharacterized conserved protein RhaS, contains 28 RHS repeats	R	60288	70535	130823
COG5941	CRISPR-Cas system type I-C effector complex large subunit Cas8c	V	3629	3787	7416
COG0608	ssDNA-specific exonuclease RecJ, DHH superfamily, may be involved in archaeal DNA replication intiation	L	14601	16548	31149
COG1654	Biotin operon repressor	K	38	19	57
COG0384	Predicted epimerase YddE/YHI9, PhzF superfamily	R	378	754	1133
COG2848	Uncharacterized conserved protein, UPF0210 family	D	5055	5994	11049
COG4608	ABC-type oligopeptide transport system, ATPase component	E	2177	1932	4108
COG1299	Phosphotransferase system, fructose-specific IIC component	G	1993	1486	3479
COG1739	Putative translation regulator, IMPACT (imprinted ancient) protein family	R	7168	7509	14677
COG0125	Thymidylate kinase	F	7097	8182	15279
COG5373	Uncharacterized membrane protein	S	16	57	73
COG4932	Clumping factor A-related surface protein, MSCRAMM (microbial surface components recognizing adhesive matrix molecules) family, DEv-IgG fold	M	48208	60570	108778
COG1737	DNA-binding transcriptional regulator, MurR/RpiR family, contains HTH and SIS domains	K	5912	8961	14873
COG3275	Sensor histidine kinase, LytS/YehU family	T	6138	6508	12646

COG0801	7,8-dihydro-6-hydroxymethylpterin pyrophosphokinase (folate biosynthesis)	H	4437	4290	8727
COG5397	Uncharacterized conserved protein	S	2	14	16
COG2079	2-methylcitrate dehydratase PrpD	G	1276	2353	3629
COG1780	Flavodoxin NrdI, NrdF-interacting activator of class Ib ribonucleotide reductase	F	36	41	77
COG0013	Alanyl-tRNA synthetase	J	16858	20177	37035
COG2094	3-methyladenine DNA glycosylase Mpg	L	1648	1581	3229
COG4791	Type III secretory pathway, EscT/YscT component	U	4	9	13
COG4851	Uncharacterized conserved protein involved in sex pheromone biosynthesis	R	128	165	292
COG0242	Peptide deformylase	J	7422	8467	15889
COG2107	1,4-dihydroxy-6-naphtoate synthase (menaquinone biosynthesis, futalosine pathway)	H	265	129	395
COG2025	Electron transfer flavoprotein, alpha subunit FixB	C	3848	6005	9853
COG0225	Peptide methionine sulfoxide reductase	O	1202	2420	3622
COG4972	Type IV pilus assembly protein, ATPase PilM	N;W	3187	3778	6964
COG5503	DNA-dependent RNA polymerase auxiliary subunit epsilon	K;V	26	15	41
COG1922	UDP-N-acetyl-D-mannosaminuronic acid transferase, WecB/TagA/CpsF family	M	3570	5079	8649
COG3957	Phosphoketolase	G	948	1534	2482
COG3019	Uncharacterized metal-binding protein, DUF411 family	S	1	3	3
COG5817	Stage II sporulation protein SpoIIE/SpoIIH (serine phosphatase - sigma-F activation)	D	10158	11021	21180
COG3384	Aromatic ring-opening dioxygenase, catalytic subunit, LigB family	Q	4	9	13

COG3158	K+ uptake protein Kup	P	361	489	850
COG3831	WGR domain, predicted DNA-binding domain in MolR	K	801	946	1747
COG5386	Heme-binding NEAT domain	P	396	426	821
COG4476	Uncharacterized conserved protein YktA, UPF0223 family	S	20	27	48
COG4209	ABC-type polysaccharide transport system, permease component	G	14207	14559	28766
COG0709	Selenophosphate synthase	E	519	1244	1764
COG0142	Geranylgeranyl pyrophosphate synthase	H	12364	13992	26356
COG1043	Acyl-[acyl carrier protein]--UDP-N-acetylglucosamine O-acyltransferase	M	4600	5667	10267
COG0784	CheY-like REC (receiver) domain, includes chemotaxis protein CheY and sporulation regulator Spo0F	T	8647	11323	19970
COG1226	Voltage-gated potassium channel Kch	P	220	290	510
COG5424	Pyrroloquinoline quinone (PQQ) biosynthesis protein C	H	0	1	1
COG0363	6-phosphogluconolactonase/Glucosamine-6-phosphate isomerase/deaminase	G	5603	8051	13654
COG3914	Predicted O-linked N-acetylglucosamine transferase, SPINDLY family	O	211	457	668
COG0069	Glutamate synthase domain 2	E	1934	2209	4142
COG0747	ABC-type transport system, periplasmic component	E	15449	26902	42351
COG4733	Phage-related protein, tail protein J	X	1272	1313	2585
COG2838	Monomeric isocitrate dehydrogenase	C	34	0	34
COG4926	Phage-related protein	X	5040	7738	12778
COG1466	DNA polymerase III, delta subunit	L	10381	11742	22123
COG0573	ABC-type phosphate transport system, permease component	P	6734	6791	13524
COG0724	RNA recognition motif (RRM) domain	J	318	700	1018

COG0820	Adenine C2-methylase RlmN of 23S rRNA A2503 and tRNA A37	J	11858	12893	24751
COG1953	Cytosine/uracil/thiamine/allantoin permease	F;H	479	645	1124
COG1214	tRNA A37 threonylcarbamoyladenosine modification protein TsaB	J	7822	9273	17095
COG0049	Ribosomal protein S7	J	3681	4233	7914
COG0017	Aspartyl/asparaginyl-tRNA synthetase	J	10561	13335	23896
COG3079	Uncharacterized conserved protein YgfB, UPF0149 family	S	0	1	1
COG4403	Lantibiotic modifying enzyme	V	835	619	1454
COG0234	Co-chaperonin GroES (HSP10)	O	2252	2336	4588
COG4393	Uncharacterized membrane protein	S	13	1	14
COG3212	PepSY domain containing protein, regulator of zincin peptidase activity	O	2683	4091	6774
COG1389	DNA topoisomerase VI, subunit B	L	59	6	66
COG2862	Uncharacterized membrane protein YqhA	S	11	0	11
COG3366	Uncharacterized membrane protein	S	23	45	68
COG0189	Glutathione synthase, LysX or RimK-type ligase, ATP-grasp superfamily	E;H;J;Q	4538	5832	10370
COG2223	Nitrate/nitrite transporter NarK	P	5835	5892	11727
COG5475	Uncharacterized conserved protein YodC, DUF2158 family	S	12	4	16
COG4808	Uncharacterized lipoprotein YehR, DUF1307 family	S	0	1	1
COG3187	Heat shock protein HslJ	O	3125	2708	5833
COG3439	Uncharacterized conserved protein, DUF302 family	S	0	2	2
COG0113	Delta-aminolevulinic acid dehydratase, porphobilinogen synthase	H	944	2034	2978
COG1182	FMN-dependent NADH-azoreductase	C	215	437	652
COG2861	Uncharacterized conserved protein YibQ, putative polysaccharide deacetylase 2 family	G	69	226	295

COG0364	Glucose-6-phosphate 1-dehydrogenase	G	303	439	742
COG1118	ABC-type sulfate/molybdate transport systems, ATPase component	P	944	1398	2342
COG0223	Methionyl-tRNA formyltransferase	J	11191	14245	25436
COG1918	Fe2+ transport protein FeoA	P	2677	2956	5632
COG3321	Acyl transferase domain in polyketide synthase (PKS) enzymes	Q	163	1224	1387
COG0822	Fe-S cluster assembly scaffold protein IscU, NifU family	O	5238	6380	11618
COG3391	DNA-binding beta-propeller fold protein YncE	R	11517	12671	24187
COG4380	Uncharacterized conserved protein, DUF799 domain	S	7	14	21
COG5283	Phage-related tail protein	X	3734	4042	7776
COG3899	Predicted ATPase	R	11	46	57
COG2969	Stringent starvation protein B, binds SsrA peptide	O	0	84	84
COG4721	ABC-type thiamine/hydroxymethylpyrimidine transport	H	89	121	210
COG3176	Putative hemolysin	R	7221	6621	13842
COG4271	Predicted nucleotide-binding protein containing TIR -like domain	R	1004	1157	2161
COG5014	Uncharacterized conserved protein TM0948, MoaA-related, radical SAM	R	11	6	17
COG2721	Altronate dehydratase	G	6121	6038	12159
COG3774	Mannosyltransferase OCH1 or related enzyme	M	5958	6167	12125
COG1969	Ni,Fe-hydrogenase I cytochrome b subunit	C	71	735	806
COG0548	N-acetylglutamate kinase	E	4804	6785	11589
COG1683	Uncharacterized conserved protein YbbK, DUF523 family	S	653	960	1613

COG2890	Methylase of polypeptide chain release factors	J	11671	14212	25883
COG3241	Azurin	C	0	1	1
COG1515	Deoxyinosine 3'-endonuclease (endonuclease V)	L	286	75	361
COG0451	Nucleoside-diphosphate-sugar epimerase	M	42520	46417	88937
COG4963	Flp pilus assembly ATPase CpaE/TadZ, contains N-terminal REC/TadZ_N domain	U;W	265	547	812
COG4960	Flp pilus assembly protein, peptidase CpaA	O;T	661	741	1402
COG3395	D-threonate/D-erythronate kinase OtnK and related C4-acid sugar kinases, YgbK/DUF1537 family	G	915	1435	2350
COG0567	2-oxoglutarate dehydrogenase complex, dehydrogenase (E1) component, and related enzymes	C	14	946	960
COG0267	Ribosomal protein L33	J	387	359	746
COG1390	Archaeal/vacuolar-type H+-ATPase subunit E/Vma4	C	2289	2674	4963
COG5890	Uncharacterized sporulation proteins YjcM and YhbB, contain C-terminal amidase domain	D	617	702	1319
COG0149	Triosephosphate isomerase	G	7545	8623	16168
COG1950	Uncharacterized membrane protein YvlD, DUF360 family	S	235	467	702
COG5900	Spore coat protein YybI (inner spore coat)	D	28	60	88
COG4566	DNA-binding response regulator, FixJ family, consists of REC and HTH domains	T;K	21	1696	1717
COG4834	Uncharacterized conserved protein, DUF2184 domain	S	465	474	940
COG2236	Hypoxanthine phosphoribosyltransferase	H	206	442	648
COG0042	tRNA-dihydrouridine synthase	J	12434	15257	27692

COG1763	Molybdopterin-guanine dinucleotide biosynthesis protein	H	162	631	792
COG3897	Protein N-terminal and lysine N-methylase, NNT1/EFM7 family	O	37	29	66
COG4887	Uncharacterized metal-binding protein MJ0455, DUF1847 family	S	167	505	673
COG5526	Lysozyme family protein	R	4	22	26
COG1247	L-amino acid N-acyltransferase MnaT	E	5925	6315	12240
COG4105	Outer membrane protein assembly factor BamD, BamD/ComL family	M	4418	4441	8860
COG1477	FAD:protein FMN transferase ApbE	H;O	9300	9774	19074
COG1637	Endonuclease NucS, RecB family	L	241	133	374
COG0295	Cytidine deaminase	F	5642	5793	11435
COG2855	Uncharacterized membrane protein YadS, UPF0324 family	S	3177	4040	7217
COG3250	Beta-galactosidase/beta-glucuronidase	G	65172	70149	135321
COG2935	Arginyl-tRNA--protein-N-Asp/Glu arginylyltransferase	O	16	5	21
COG3842	ABC-type Fe3+/spermidine/putrescine transport systems, ATPase component	E	6596	9277	15873
COG3208	Surfactin synthase thioesterase subunit	Q	143	189	332
COG5826	Stage III sporulation protein SpoIIIAE, component of the engulfment complex	D	6115	7027	13143
COG0058	Glucan phosphorylase	G	16329	19273	35603
COG3179	Chitinase, GH19 family	G	80	34	114
COG1758	DNA-directed RNA polymerase, subunit K/omega	K	1994	2133	4127
COG1764	Organic hydroperoxide reductase OsmC/OhrA	V	0	7	7
COG0829	Urease accessory protein UreH	O	1366	1498	2864
COG4646	Adenine-specific DNA methylase, N12 class	L	11510	11217	22727

COG5464	Recombination-promoting DNA endonuclease RpnC/YadD	L	2643	2591	5234
COG0425	Sulfur carrier protein TusA (tRNA thiolation, molybdenum cofactor	J;H;O	293	655	947
COG0300	Short-chain dehydrogenase	R	10034	12002	22036
COG4771	Outer membrane receptor for ferrienterochelin and colicins	P	33932	36661	70593
COG1879	ABC-type sugar transport system, periplasmic component, contains N-terminal xre family HTH domain	G	8530	15496	24026
COG1703	GTPase of the G3E family (not a periplasmic protein kinase)	O	2744	2886	5630
COG3254	L-rhamnose mutarotase	M	665	877	1542
COG0838	NADH:ubiquinone oxidoreductase subunit 3 (chain A)	C	712	786	1498
COG2121	Uncharacterized conserved protein, lysophospholipid acyltransferase (LPLAT) superfamily	S	278	782	1060
COG1234	Ribonuclease BN, tRNA processing enzyme	J	6796	7965	14761
COG1396	Transcriptional regulator, contains XRE-family HTH domain	K	117995	122109	240103
COG3039	Transposase and inactivated derivatives, IS5 family	X	8204	16471	24675
COG4575	Membrane-anchored ribosome-binding protein ElaB, inhibits growth in stationary phase, YqjD/DUF883 family	J	58	109	167
COG4496	Predicted DNA-binding transcriptional regulator YerC, contains ArsR-like HTH domain	R	1632	1717	3348
COG3573	Predicted oxidoreductase	R	100	201	301
COG5813	Sporulation sigma factor SigG (forespore-specific, SpoIIIG)	D;K	4644	5650	10294

COG1665	Predicted nucleotidyltransferase AF1967	R	21	35	56
COG0298	Hydrogenase maturation factor HybG, HypC/HupF family	O	78	276	354
COG4111	Uncharacterized conserved protein	S	259	338	597
COG0098	Ribosomal protein S5	J	4458	5433	9891
COG0794	D-arabinose 5-phosphate isomerase GutQ	G;M	1706	2165	3871
COG3084	Uncharacterized conserved protein YihD, DUF1040 family	S	0	2	2
COG5368	Endo-beta-1,2-glucanase, glucoamylase superfamily	G	278	592	870
COG2971	BadF-type ATPase, related to human N-acetylglucosamine kinase	G	2449	3060	5509
COG0212	5-formyltetrahydrofolate cyclo-ligase	H	5943	6189	12132
COG3426	Butyrate kinase	C	3723	4957	8680
COG4569	Acetaldehyde dehydrogenase (acetylating)	Q	64	37	101
COG3848	Phosphohistidine swiveling domain of PEP-utilizing enzymes	T	36	24	60
COG4716	Myosin-crossreactive antigen (function unknown)	S	230	455	684
COG1376	Lipoprotein-anchoring transpeptidase ErfK/SrfK	M	7201	11646	18847
COG0488	ATPase components of ABC transporters with duplicated ATPase domains	R	28758	35523	64282
COG0324	tRNA A37 N6-isopentenylltransferase MiaA	J	10797	12563	23360
COG2828	2-Methylaconitate cis-trans-isomerase PrpF (2-methyl citrate pathway)	C	172	642	814
COG2368	Aromatic ring hydroxylase	Q	226	887	1113
COG0394	Protein-tyrosine-phosphatase	T	5193	6645	11838
COG3328	Transposase (or an inactivated derivative)	X	27023	24720	51743
COG5872	Stage IV sporulation protein YqfD	D	6913	7813	14726
COG1788	Acyl CoA:acetate/3-ketoacid CoA transferase, alpha subunit	I	268	1009	1276

COG0809	S-adenosylmethionine:tRNA-ribosyltransferase-isomerase (queuine synthetase)	J	12291	14024	26315
COG1337	CRISPR-Cas system type III CSM-effector complex subunit Csm3, RAMP superfamily Cas7 group	V	912	1258	2170
COG3264	Small-conductance mechanosensitive channel MscK	M	3396	4656	8051
COG1484	DNA replication protein DnaC	L	22002	26690	48692
COG0119	Isopropylmalate/homocitrate/citramalate synthases	E	15229	16890	32119
COG3369	Uncharacterized conserved protein, contains Zn-finger domain of CDGSH type	S	864	621	1485
COG2814	Predicted arabinose efflux permease AraJ, MFS family	G	13757	20860	34617
COG3851	Signal transduction histidine kinase UhpB, glucose-6-phosphate specific	T	0	5	5
COG0109	Polyprenyltransferase (heme O synthase)	H;I	2	7	8
COG0327	Putative GTP cyclohydrolase 1 type 2, NIF3 family	H	7572	8213	15785
COG2503	Predicted secreted acid phosphatase	R	60	68	128
COG0629	Single-stranded DNA-binding protein	L	14315	15678	29993
COG1532	Predicted RNA-binding protein	R	1	5	6
COG0661	Predicted protein kinase regulating ubiquinone biosynthesis, AarF/ABC1/UbiB family	H;T	531	864	1395
COG0713	NADH:ubiquinone oxidoreductase subunit 11 or 4L (chain K)	C	458	527	985
COG3202	ATP/ADP translocase	C	109	27	135
COG4420	Uncharacterized membrane protein	S	108	173	282
COG1802	DNA-binding transcriptional regulator, GntR family	K	4822	7720	12542

COG2461	Uncharacterized conserved protein AF0170, contains SAB, hemerythrin HHE, and PAS domains	R	175	108	283
COG0680	Ni,Fe-hydrogenase maturation factor	C	64	542	606
COG4719	Uncharacterized conserved protein, UCP014979 family	S	0	8	8
COG0668	Small-conductance mechanosensitive	M	10134	10558	20692
COG5801	Stage 0 sporulation initiation regulator Spo0A (response regulator, REC-HTH	D;T	8450	10032	18482
COG5349	Uncharacterized conserved protein, DUF983 family	S	2	5	7
COG1608	Isopentenyl phosphate kinase	I	28	0	28
COG0033	Phosphoglucomutase/phosphomannomutase	G	28	181	209
COG0019	Diaminopimelate decarboxylase	E	14678	16368	31046
COG4732	ECF-type thiazole transporter, membrane (S) component ThiW	H	748	703	1452
COG1208	NDP-sugar pyrophosphorylase, includes eIF-2Bgamma, eIF-2Bepsilon, and LPS biosynthesis protein s	J;M	10704	10501	21205
COG3309	Virulence-associated protein VapD, endoribonuclease	V	30	45	75
COG0121	Predicted glutamine amidotransferase YafJ	R	83	436	519
COG3868	Predicted glycosyl hydrolase, GH114 family	G	5	538	544
COG0523	Zinc metallochaperone YeiR/ZagA and related GTPases, G3E family	R	8172	9853	18025
COG2811	Archaeal/vacuolar-type H+-ATPase subunit H	C	1713	1408	3121
COG2919	Cell division protein FtsB	D	627	961	1588
COG3058	Formate dehydrogenase maturation protein FdhE	C;O	0	185	186
COG0855	Polyphosphate kinase	P	7698	8533	16232

COG1875	Predicted ribonuclease YlaK, contains NYN-type RNase and PhoH-family ATPase domains	R	389	1109	1498
COG1770	Protease II	E	6	7	14
COG1936	Broad-specificity NMP kinase	F	288	277	565
COG1307	Fatty acid-binding protein DegV (function unknown)	I	23892	28076	51969
COG2501	Ribosome-associated protein YbcJ, S4-like RNA-binding protein	J	1003	1019	2022
COG0745	DNA-binding response regulator, OmpR family, contains REC and winged-helix (wHTH) domain	T;K	89803	100687	190491
COG0376	Catalase (peroxidase I)	P	1	10	11
COG2131	Deoxycytidylate deaminase	F	5747	6322	12069
COG4640	Uncharacterized protein YvbJ, contains N-terminal Zn ribbon domain	S	1816	1678	3494
COG1116	ABC-type nitrate/sulfonate/bicarbonate transport system, ATPase component	P	5905	8762	14666
COG0126	3-phosphoglycerate kinase	G	8335	10554	18890
COG2924	Fe-S cluster biosynthesis and repair protein YggX	P;O	0	44	44
COG4878	Uncharacterized conserved protein, DUF2194 domain	S	683	958	1642
COG5830	Stage III sporulation protein SpoIIID, transcriptional regulator of sigma-E- and sigma-K-dependent genes	D;K	1535	1501	3036
COG4803	Uncharacterized membrane protein	S	34	42	76
COG3347	Rhamnose utilisation protein RhaD, predicted bifunctional aldolase and	G	501	532	1032
COG4012	Uncharacterized protein, DUF1786 family, actin-like ATPase superfamily	R	8	15	23

COG5304	Predicted DNA binding protein, CopG/RHH family	K	0	0	0
COG0593	Chromosomal replication initiation ATPase DnaA	L	14138	16052	30190
COG3931	Predicted N-formylglutamate	E	0	10	10
COG3786	L,D-peptidoglycan transpeptidase YkuD, ErfK/YbiS/YcfS/YnhG family	M	510	1091	1602
COG4089	Uncharacterized membrane protein	S	133	335	468
COG5815	Stage II sporulation protein SpoIIAA (antagonist of anti-sigma F factor)	D;T	1847	1849	3696
COG1832	Predicted CoA-binding protein	R	8	19	27
COG4809	Archaeal ADP-dependent glucokinase/phosphofructokinase	G	77	227	304
COG1492	Cobyric acid synthase	H	3979	4839	8818
COG5511	Phage capsid protein	X	2878	2649	5527
COG2201	Chemotaxis response regulator CheB, contains REC and protein-glutamate methylesterase domains	T	4834	3981	8815
COG4943	Redox-sensing c-di-GMP phosphodiesterase, contains CSS-motif and EAL domains	T	0	12	12
COG3017	Outer membrane lipoprotein LolB, involved in outer membrane biogenesis	M	5	135	140
COG2263	Predicted RNA methylase	R	346	484	829
COG3745	Flp pilus assembly protein CpaB	U;W	1558	1305	2863
COG4582	Cell division protein ZapD, interacts with FtsZ	D	1	152	152
COG5934	CRISPR-Cas system endoribonuclease Cas6e, RAMP superfamily	V	201	217	419
COG0243	Anaerobic selenocysteine-containing dehydrogenase	C	3741	7748	11490
COG4798	Predicted methyltransferase	R	62	208	270

COG0356	FoF1-type ATP synthase, membrane subunit a	C	6596	6905	13501
COG4868	Uncharacterized conserved protein, UPF0371 family	S	4959	6235	11194
COG1485	Cell division protein ZapE (Z ring-associated ATPase), AFG1 superfamily	D	5	9	14
COG1609	DNA-binding transcriptional regulator, LacI/PurR family	K	29154	37829	66983
COG5452	Uncharacterized conserved protein	S	1	3	4
COG3910	Predicted ATPase	R	2338	2852	5190
COG0350	DNA repair enzyme Ada (O6-methylguanine-DNA--protein-cysteine methyltransferase)	L	4998	5845	10843
COG1558	Flagellar basal body rod protein FlgC	N	1796	1423	3219
COG3325	Chitinase, GH18 family	G	3860	5630	9490
COG3314	Spore dipicolinate transporter SpoVV/YjiH/YlbJ, contains nucleoside recognition GATE domain	D;E	5424	8533	13958
COG2764	Zn-dependent glyoxalase, PhnB family	C	424	396	820
COG0051	Ribosomal protein S10	J	1527	1651	3178
COG1834	N-Dimethylarginine dimethylaminohydrolase	E	216	458	674
COG4869	Propanediol utilization protein	Q	2434	2952	5386
COG5389	Uncharacterized conserved protein, DUF721 domain	S	13	76	90
COG3193	Heme-binding protein HbpS, GlcG/HbpS family	T	135	259	394
COG0065	Homoaconitase/3-isopropylmalate dehydratase large subunit	E	4674	7874	12547
COG3340	Peptidase E	E	3419	4106	7524
COG2605	Predicted kinase related to galactokinase and mevalonate kinase	R	2959	3433	6393
COG3265	Gluconate kinase	G	2	2	4

COG3247	Acid resistance membrane protein HdeD, DUF308 family	R	6793	7316	14109
COG2169	Methylphosphotriester-DNA--protein-cysteine methyltransferase (N-terminal fragment of Ada), contains Zn-binding and two AraC-type DNA-binding domains	L	2498	3165	5663
COG3586	Uncharacterized protein domain, C-terminal to COG1479 DNAse/DNA nickase	R	500	726	1226
COG0089	Ribosomal protein L23	J	2758	3156	5913
COG1292	Choline-glycine betaine transporter	M	1054	2316	3370
COG2942	Mannose or cellobiose epimerase, N-acyl-D-glucosamine 2-epimerase family	G	6022	6201	12222
COG5336	FoF1-type ATP synthase AtpZ/Atp1/AtpQ subunit, putative Ca2+/Mg2+ transporter	C	369	457	826
COG1793	ATP-dependent DNA ligase	L	1194	1997	3191
COG3757	Lyzozyme M1 (1,4-beta-N-acetylmuramidase), GH25 family	M	21561	20531	42091
COG2256	Replication-associated recombination protein RarA (DNA-dependent ATPase)	L	13920	16354	30274
COG1450	Type II secretory pathway component GspD/PulD (secretin)	U	235	692	927
COG3511	Phospholipase C	M	5	0	5
COG3919	Predicted ATP-dependent carboligase, ATP-grasp superfamily	R	1672	2901	4573
COG3465	Uncharacterized conserved protein YwgA	S	208	84	292
COG2129	Predicted phosphoesterase, related to the Icc protein	R	5845	8563	14408
COG1308	Transcription factor homologous to NACalpha-BTF3	K	227	261	488
COG1329	RNA polymerase-interacting regulator, CarD/CdnL/TRCF family	K	3623	4118	7740

COG3941	Phage tail tape-measure protein, controls tail length	X	3333	2594	5927
COG0469	Pyruvate kinase	G	10146	12007	22153
COG4962	Pilus assembly protein, ATPase of CpaF family	U;W	7839	8359	16199
COG2939	Carboxypeptidase C (cathepsin A)	E	136	206	342
COG3770	Murein endopeptidase MepA (D-alanyl-D-alanine-endopeptidase)	M	0	1	1
COG1285	Magnesium uptake protein YhiD/SapB, involved in acid resistance	P	6757	7731	14488
COG0369	Flavoprotein (flavin reductase) subunit CysJ of sulfite and N-hydroxylaminopurine reductases	F;P	281	752	1033
COG0633	Ferredoxin	C	131	350	481
COG1963	Acid phosphatase family membrane protein YuiD	R	1270	1574	2844
COG1864	DNA/RNA endonuclease G, NUC1	F	4095	4299	8394
COG3705	ATP phosphoribosyltransferase regulatory subunit HisZ	E	4407	5190	9597
COG1650	D-tyrosyl-tRNA(Tyr) deacylase	J	46	54	101
COG1612	Heme A synthase	H	2	0	2
COG0466	ATP-dependent Lon protease, bacterial type	O	12779	15501	28281
COG0083	Homoserine kinase	E	802	1221	2023
COG5444	Predicted ribonuclease, toxin component of the YeeF-YezG toxin-antitoxin module	V	1968	1226	3194
COG5276	Uncharacterized secreted protein, contains LVIVD repeats, choice-of-anchor domain	S	265	463	728
COG0249	DNA mismatch repair ATPase MutS	L	24274	29505	53779
COG2906	Bacterioferritin-associated ferredoxin	P	35	79	114
COG4471	Uncharacterized conserved protein YlbG, UPF0298 family	S	0	30	30

COG5825	Stage III sporulation protein SpoIIIAD, component of the engulfment complex	D	2319	2889	5209
COG4625	Uncharacterized conserved protein, contains a C-terminal beta-barrel porin domain	S	4678	13868	18546
COG4818	Uncharacterized membrane protein MJ1443, UPF0132 family	S	522	555	1078
COG3324	Lactoylglutathione lyase-related enzyme, vicinal oxygen chelate (VOC) family	R	235	456	691
COG4547	Cobalamin biosynthesis cobaltochelatase CobT subunit	H	1303	2029	3332
COG1914	Mn2+ or Fe2+ transporter, NRAMP family	P	339	856	1195
COG1430	Uncharacterized conserved membrane protein, UPF0127 family	S	79	188	267
COG0532	Translation initiation factor IF-2, a GTPase	J	14436	18001	32437
COG5039	Exopolysaccharide biosynthesis protein EpsI, predicted pyruvyl transferase	G;M	507	576	1083
COG3148	tRNA U47 aminocarboxypropyltransferaseTapT/TuaA/YfiP, DTW domain	J	0	1	1
COG2986	Histidine ammonia-lyase	E	1615	2845	4459
COG3947	Two-component response regulator, SAPR family, consists of REC, wHTH and BTAD domains	T;K	1063	1159	2222
COG5489	Uncharacterized conserved protein, DUF736 family	S	4	2	6
COG4693	Oxidoreductase (NAD-binding), involved in siderophore biosynthesis	P	9	0	9
COG1921	Seryl-tRNA(Sec) selenium transferase	J	206	575	781
COG0328	Ribonuclease HI	L	3780	5393	9173
COG0571	dsRNA-specific ribonuclease	K	7267	8527	15793

COG2071	Gamma-glutamyl-gamma-aminobutyrate hydrolase PuuD (putrescine degradation), contains GATase1-like domain	E	2366	4046	6413
COG2014	Uncharacterized conserved protein, contains DUF4213 and DUF364 domains	S	53	103	156
COG1638	TRAP-type C4-dicarboxylate transport system, periplasmic component	G	3171	9735	12906
COG4268	5-methylcytosine-specific restriction endonuclease McrBC, regulatory subunit McrC	V	1594	1786	3381
COG2205	K+-sensing histidine kinase KdpD	T	25445	31523	56968
COG4976	Predicted methyltransferase, contains TPR repeat	R	1328	1197	2525
COG3666	Transposase, IS1182 family	X	27951	36696	64647
COG1183	Phosphatidylserine synthase	I	3246	3834	7080
COG1364	Glutamate N-acetyltransferase (ornithine transacetylase)	E	4967	6010	10976
COG2386	ABC-type transport system involved in cytochrome c biogenesis, permease component	O	64	233	297
COG0361	Translation initiation factor IF-1	J	807	1045	1852
COG4284	UDP-N-acetylglucosamine pyrophosphorylase	G	2376	3412	5788
COG2759	Formyltetrahydrofolate synthetase	F	7486	10740	18226
COG0159	Tryptophan synthase alpha chain	E	3613	3654	7267
COG4842	Secreted virulence factor YukE/EsxA, WXG100 family	V	415	543	958
COG4984	Uncharacterized membrane protein	S	60	64	123
COG3535	Uncharacterized conserved protein, DUF917 family	S	130	328	458
COG4895	Uncharacterized conserved protein YwbE, DUF2196 family	S	19	44	63

COG5679	Cytochrome b6f complex subunit IV, PetD, 17 kd cytochrome b	C	13	6	20
COG2868	Uncharacterized conserved protein YsxB, DUF464 family	S	1849	2274	4123
COG3775	Phosphotransferase system, galactitol-specific IIC component	G	466	915	1380
COG0171	NH3-dependent NAD+ synthetase	H	13486	15935	29421
COG3706	Two-component response regulator, PleD family, consists of two REC domains and a diguanylate cyclase (GGDEF) domain	T;K	4107	4161	8268
COG0012	Ribosome-binding ATPase YchF, GTP1/OBG family	J	9059	13117	22176
COG3759	Uncharacterized membrane protein	S	1	41	43
COG1632	Ribosomal protein L15E	J	4	2	7
COG4856	Cyclic di-AMP synthase regulator CdaR, YbbR domain	T	6196	7457	13653
COG0314	Molybdopterin synthase catalytic subunit MoaE	H	87	255	341
COG5470	Uncharacterized conserved protein, DUF1330 family	S	0	3	4
COG3754	Lipopolysaccharide biosynthesis protein	M	3738	4023	7760
COG2170	Gamma-glutamyl:cysteine ligase YbdK, ATP-grasp superfamily	O	0	1	1
COG0675	Transposase	X	5206	7165	12372
COG2921	Putative lipoate-binding regulatory protein, UPF0250 family	T	5	1	6
COG4399	Uncharacterized membrane protein YheB, UPF0754 family	S	245	318	564
COG4188	Predicted dienelactone hydrolase	R	1924	2502	4426
COG3283	Transcriptional regulator TyrR of aromatic amino acids metabolism	K;E	0	10	10

COG0208	Ribonucleotide reductase beta subunit, ferritin-like domain	F	127	338	465
COG4753	Two-component response regulator, YesN/AraC family, consists of REC and AraC-type DNA-binding domains	T;K	11849	20604	32453
COG0798	Arsenite efflux pump ArsB, ACR3 family	P	3	9	12
COG2163	Ribosomal protein L14E/L6E/L27E	J	1549	1580	3129
COG2043	Uncharacterized conserved protein, DUF169 family	S	105	262	367
COG3012	Uncharacterized conserved protein YchJ, contains N- and C-terminal SEC-C domains	S	33	155	189
COG4739	Uncharacterized conserved protein, contains ferredoxin domain	S	349	493	841
COG1387	Histidinol phosphatase or related hydrolase of the PHP family	E;R	12131	15562	27693
COG3619	Uncharacterized membrane protein YoaK, UPF0700 family	S	922	951	1873
COG3822	D-lyxose ketol-isomerase	G	487	1509	1996
COG5564	Predicted TIM-barrel enzyme	S	56	247	303
COG3146	Predicted N-acyltransferase	R	119	243	362
COG1260	Myo-inositol-1-phosphate synthase	I	1031	1149	2180
COG1493	Serine kinase of the HPr protein, regulates carbohydrate metabolism	T	7616	8814	16430
COG3410	Uncharacterized conserved protein, DUF2075 family	S	896	1669	2565
COG0353	Recombinational DNA repair protein RecR	L	6441	7935	14377
COG1799	Cell division protein SepF/YlmF, interacts with FtsZ	D	3311	4264	7575
COG0332	3-oxoacyl-[acyl-carrier-protein] synthase III	I	8662	11712	20374
COG2445	Uncharacterized HEPN domain protein YutE, UPF0331/DUF86 family	R	27	90	117

COG5430	Fimbrial subunit ScuA/B, ScuA/B/SCPU domain	W	0	3	3
COG4564	Signal transduction histidine kinase	T	3	1	4
COG0085	DNA-directed RNA polymerase, beta subunit/140 kD subunit	K	18819	23602	42421
COG4579	Isocitrate dehydrogenase kinase/phosphatase	T	0	0	0
COG3678	Periplasmic chaperone Spy, Spy/CpxP	O	9	44	53
COG3201	Nicotinamide riboside transporter PnuC	H	2521	2911	5432
COG0383	Alpha-mannosidase	G	4337	8890	13226
COG1501	Alpha-glucosidase/xylosidase, GH31 family	G	15804	20858	36662
COG4312	Predicted dithiol-disulfide oxidoreductase, DUF899 family	R	0	3	3
COG1896	5'-deoxynucleotidase YfbR and related HD superfamily hydrolases	F;R	4265	5271	9536
COG3955	Uncharacterized conserved protein, DUF1919 family	M	923	1198	2121
COG0515	Serine/threonine protein kinase	T	49741	55602	105343
COG1649	Uncharacterized lipoprotein YddW, UPF0748 family	S	9203	10402	19605
COG3812	Uncharacterized conserved protein, DUF1993 domain	S	3	6	9
COG4386	Mu-like prophage tail sheath protein gpL	X	319	600	919
COG0776	Bacterial nucleoid DNA-binding protein IHF-alpha	L	5229	7867	13096
COG3078	Ribosome assembly protein YihI, activator of Der GTPase	J	0	2	2
COG2844	UTP:GlnB (protein PII) uridylyltransferase	O;T	7	264	271
COG1225	Peroxiredoxin	O	9939	13760	23699
COG5571	Uncharacterized conserved protein YhjY, contains autotransporter beta-barrel domain	R	69	83	152
COG3271	Predicted double-glycine leader peptidase, C39-like (CLD) domain	U	90	165	255

COG1271	Cytochrome bd-type quinol oxidase, subunit 1	C	2090	3256	5346
COG3315	O-Methyltransferase involved in polyketide biosynthesis	Q	1219	834	2053
COG5467	Uncharacterized conserved protein, DUF1476 domain	S	2	0	2
COG3768	Uncharacterized membrane protein YcjF, UPF0283 family	S	98	36	133
COG4767	Glycopeptide antibiotics resistance protein	V	5017	7027	12044
COG4843	Uncharacterized conserved protein YebE, UPF0316/DUF2179 family	S	136	286	421
COG1472	Periplasmic beta-glucosidase and related glycosidases	G	48704	65484	114188
COG0351	Hydroxymethylpyrimidine/phosphomethylpyrimidine kinase	H	4085	5223	9308
COG1533	DNA repair photolyase	L	8189	9539	17728
COG2336	Antitoxin component MazE of the MazEF toxin-antitoxin module	V	425	440	866
COG3639	ABC-type phosphate/phosphonate transport system, permease component	P	257	258	515
COG1744	Lipoprotein Med, regulator of KinD/Spo0A, PBP1-ABC superfamily, includes NupN	T	10420	14141	24562
COG0174	Glutamine synthetase	E	7297	9055	16352
COG3958	Transketolase, C-terminal subunit	G	7738	10969	18707
COG1167	DNA-binding transcriptional regulator, MocR family, contains an aminotransferase domain	K;E	14558	17102	31660
COG2720	Vancomycin resistance protein YoaR (function unknown), contains peptidoglycan-binding and VanW domains	V	7953	12711	20664
COG4668	Mannitol/fructose-specific phosphotransferase system, IIA domain	G	154	688	842

COG3363	Archaeal IMP cyclohydrolase	F	3700	4139	7839
COG3459	Cellobiose phosphorylase	G	12279	17730	30009
COG4226	Predicted nuclease of the RNAse H fold, HicB family	R	663	1090	1752
COG2377	1,6-Anhydro-N-acetylmuramate kinase	M	195	1056	1251
COG4306	Uncharacterized conserved protein, DUF2321 domain	S	76	85	161
COG0587	DNA polymerase III, alpha subunit	L	21094	28509	49603
COG2216	K+ transport ATPase, ATPase subunit KdpB	P	178	278	456
COG3667	Uncharacterized conserved protein involved in copper resistance	P	9	13	22
COG2317	Zn-dependent carboxypeptidase, M32 family	O	816	1764	2579
COG0760	Peptidyl-prolyl isomerase, parvulin family	O	14833	19655	34488
COG0666	Ankyrin repeat	T	1160	2930	4091
COG1408	Predicted phosphohydrolase, MPP superfamily	R	10090	14186	24276
COG5388	Uncharacterized conserved protein	S	2	3	6
COG3625	Alpha-D-ribose 1-methylphosphonate 5-triphosphate synthase subunit PhnH	P	101	33	134
COG3915	Uncharacterized conserved protein	S	0	1	1
COG3116	Cell division protein FtsL, interacts with FtsB and FtsQ	D	9	0	10
COG4421	Capsular polysaccharide biosynthesis protein	M	171	229	400
COG4465	GTP-sensing pleiotropic transcriptional regulator CodY	K	1047	1474	2521
COG2929	Uncharacterized conserved protein, DUF497 family	S	622	573	1195
COG2344	NADH/NAD ratio-sensing transcriptional regulator Rex	K	5896	5920	11816
COG1620	L-lactate permease	C	296	454	750
COG0779	Ribosome maturation factor RimP	J	5013	5931	10944

COG1596	Periplasmic protein Wza involved in polysaccharide export, contains SLBB domain of the beta-grasp fold	M	5825	7530	13355
COG0861	Tellurite resistance membrane protein TerC	P	378	726	1104
COG5892	Spore coat protein YlbD, FlgN family	D	68	77	145
COG2902	NAD-specific glutamate dehydrogenase	E	12	10	21
COG5496	Predicted thioesterase	R	992	1312	2303
COG1335	Nicotinamidase-related amidase	H;R	3693	4631	8323
COG2918	Gamma-glutamylcysteine synthetase	H	49	249	298
COG3784	Uncharacterized conserved protein YdbL, DUF1318 family	S	1	0	1
COG3750	Uncharacterized conserved protein, UPF0335 family	S	3	43	46
COG4990	Predicted cysteine peptidase, C39 family	R	6405	7253	13658
COG5461	Type IV pilus biogenesis protein	W	1	2	3
COG3749	Uncharacterized conserved protein, DUF934 family	S	1	0	1
COG0560	Phosphoserine phosphatase	E	8562	10388	18951
COG0362	6-phosphogluconate dehydrogenase	G	288	916	1204
COG0585	tRNA(Glu) U13 pseudouridine synthase TruD	J	19	4	23
COG3766	Uncharacterized membrane protein YjfL, UPF0719 family	S	34	37	71
COG0738	Fucose permease	G	11553	15069	26621
COG1777	Predicted transcriptional regulator, ArsR family	K	33	6	39
COG3693	Endo-1,4-beta-xylanase, GH35 family	G	4394	3730	8124
COG0708	Exonuclease III	L	4649	7113	11762
COG1720	tRNA (Thr-GGU) A37 N6-methylase	J	2979	3695	6674
COG0257	Ribosomal protein L36	J	96	47	144
COG1442	Lipopolysaccharide biosynthesis protein, LPS:glycosyltransferase	M	12095	13652	25747

COG1074	ATP-dependent exoDNAse (exonuclease V) beta subunit (contains helicase and exonuclease domains)	L	21727	28470	50197
COG0475	Kef-type K+ transport system, membrane component KefB	P	9805	12367	22172
COG5416	Uncharacterized integral membrane protein YrvD	S	0	24	24
COG5809	Sporulation sensor histidine kinase E	D;T	13	103	117
COG0764	3-hydroxymyristoyl/3-hydroxydecanoyl-(acyl carrier protein) dehydratase	I	4440	5630	10070
COG4068	Predicted nucleic acid-binding protein, contains Zn-ribbon domain	R	0	8	8
COG5840	Stage V sporulation protein SpoVAF, subunit of dipicolinate uptake complex, GerA superfamily	D;E	2670	2788	5458
COG0686	Alanine dehydrogenase (includes sporulation protein SpoVN)	E	2207	3101	5309
COG1877	Trehalose-6-phosphate phosphatase	G	0	3	3
COG3670	Carotenoid cleavage dioxygenase or a related enzyme	Q	9	11	20
COG5360	Uncharacterized conserved protein, heparinase superfamily	R	1480	2334	3814
COG2052	Regulator of extracellular matrix RemA, YlzA/DUF370 family	M	893	1120	2014
COG0458	Carbamoylphosphate synthase large subunit	E;F	18369	26814	45183
COG5721	Photosystem II cytochrome b559 beta chain, PsbF	C	1	0	1
COG3835	Sugar diacid utilization regulator CdaR	K;T	6657	8730	15386
COG4728	Uncharacterized conserved protein, DUF1653 family	S	1571	1900	3471
COG4359	2-hydroxy-3-keto-5-methylthiopentenyl-1-phosphate phosphatase (methionine salvage)	E	11	93	104

COG3088	Cytochrome c-type biogenesis protein CcmH/NrfF	C;O	1	195	196
COG3304	Uncharacterized membrane protein YccF, DUF307 family	S	918	867	1786
COG3668	Plasmid stabilization system protein ParE	X	4276	4640	8916
COG0836	Mannose-1-phosphate guanylyltransferase	M	4634	4883	9517
COG4086	Uncharacterized conserved protein YpuA, DUF1002 family	S	309	835	1144
COG1145	Ferredoxin	C	2465	2864	5330
COG0354	Folate-binding protein YgfZ, synthesis and repair of Fe-S clusters	O	4	158	162
COG2076	Multidrug transporter EmrE and related cation transporters	V	545	593	1138
COG4954	Uncharacterized conserved protein, DUF2000 domain	S	885	771	1657
COG3053	Citrate lyase synthetase CitC	C	2003	2547	4550
COG5614	Phage head-tail adaptor	X	502	769	1271
COG1032	Radical SAM superfamily enzyme YgiQ, UPF0313 family	R	27116	31354	58470
COG4126	Asp/Glu/hydantoin racemase	E	404	755	1159
COG1104	Cysteine desulfurase/Cysteine sulfinate desulfinase IscS or related enzyme, NifS family	E	18107	22657	40764
COG1565	SAM-dependent methyltransferase, MidA family	R	315	610	925
COG1094	rRNA processing protein Krr1/Pno1, contains KH domain	J	7953	10655	18608
COG0018	Arginyl-tRNA synthetase	J	13165	16771	29936
COG3496	Uncharacterized conserved protein, DUF1365 family	S	0	3	3
COG3959	Transketolase, N-terminal subunit	G	7518	10217	17735

COG0816	YqgF/RuvX protein, pre-16S rRNA maturation RNase/Holliday junction resolvase/anti-termination factor	J;L;K	4599	5875	10474
COG2909	ATP-, maltotriose- and DNA-dependent transcriptional regulator MalT	K	2675	2969	5644
COG2768	Uncharacterized Fe-S cluster protein	S	7395	10526	17921
COG2303	Choline dehydrogenase or related flavoprotein	I;R	53	103	156
COG3409	Peptidoglycan-binding (PGRP) domain of peptidoglycan hydrolases	M	11989	14950	26940
COG3131	Periplasmic glucan biosynthesis protein OpgG	M	7	41	48
COG1704	Magnetosome formation protein MamQ, lipoprotein antigen LemA family	M	3411	4801	8212
COG3421	Type III restriction endonuclease	V	608	574	1182
COG4821	Uncharacterized conserved protein, contains SIS (Sugar ISomerase) phosphosugar binding domain	R	173	762	935
COG4248	Uncharacterized conserved protein YegI with protein kinase and helix-hairpin-helix DNA-binding domains	R	1433	1223	2656
COG4396	Mu-like prophage host-nuclease inhibitor protein Gam	X	529	514	1043
COG2357	ppGpp synthetase catalytic domain (RelA/SpoT-type nucleotidyltranferase)	F;T	5731	7226	12958
COG0549	Carbamate kinase	E	2294	4188	6482
COG4648	Uncharacterized membrane protein	S	1	9	9
COG2020	Protein-S-isoprenylcysteine O-methyltransferase Ste14	O	2063	2188	4251
COG3204	Uncharacterized Ca-binding beta-propeller protein YjiK	R	59	126	186

COG4745	Predicted membrane-bound mannosyltransferase, involved in protein glycosylation	R	18	41	59
COG1625	Fe-S oxidoreductase, related to NifB/MoaA family	C	3764	4645	8409
COG4283	Uncharacterized conserved protein DfsB/IRC4, DUF1706 (PF08020) domain	R	371	617	988
COG0053	Divalent metal cation (Fe/Co/Zn/Cd) efflux pump	P	10181	12562	22743
COG1139	L-lactate utilization protein LutB, contains a ferredoxin-type domain	C	3098	3335	6433
COG1152	CO dehydrogenase/acetyl-CoA synthase alpha subunit	C	2820	3700	6520
COG3478	Predicted nucleic-acid-binding protein, contains Zn-ribbon domain	R	37	54	91
COG0483	Archaeal fructose-1,6-bisphosphatase or related enzyme, inositol monophosphatase family	G	2405	4230	6635
COG0502	Biotin synthase or related enzyme	H	4058	5667	9725
COG1257	Hydroxymethylglutaryl-CoA reductase	I	12	120	132
COG2017	Galactose mutarotase or related enzyme	G	14046	17496	31542
COG4352	Ribosomal protein L13E	J	2	5	6
COG1705	Flagellum-specific peptidoglycan hydrolase FlgJ	M;N	4094	4685	8779
COG0406	Broad specificity phosphatase PhoE	G	10069	12362	22431
COG3281	Predicted trehalose synthase	G	0	7	7
COG4592	ABC-type Fe2+-enterobactin transport system, periplasmic component	P	0	5	5
COG3174	Membrane component of predicted Mg2+ transport system, contains DUF4010 domain	P	78	173	250
COG2807	Cyanate permease	P	426	1296	1721
COG0854	Pyridoxine 5'-phosphate synthase PdxJ	H	2424	2712	5136

COG3652	Predicted outer membrane protein, contains DUF4142 domain	S	3	5	8
COG0086	DNA-directed RNA polymerase, beta' subunit/160 kD subunit	K	17457	23818	41275
COG0280	Phosphotransacetylase (includes Pta, EutD and phosphobutyryltransferase)	C	8298	12077	20376
COG1194	Adenine-specific DNA glycosylase, acts on AG and A-oxoG pairs	L	5067	7725	12793
COG3852	Signal transduction histidine kinase NtrB, nitrogen specific	T	119	707	826
COG0338	DNA-adenine methylase	L	14947	15555	30502
COG3555	Aspartyl/asparaginyl beta-hydroxylase, cupin superfamily	O	4	4	8
COG1954	Glycerol-3-phosphate responsive antiterminator (mRNA-binding)	K	254	727	981
COG1900	Sulfur incorporation enzyme MA1821 (anaerobic homocysteine biosynthesis), HcyBio/DUF39 family	E	82	86	168
COG1383	Ribosomal protein S17E	J	8	6	14
COG5678	Cytochrome b6f complex cytochrome f subunit, PetA	C	12	22	33
COG4838	Uncharacterized conserved protein YlaN, UPF0358 family	S	0	21	21
COG2907	Predicted flavin-containing amine oxidase	R	0	4	4
COG4133	ABC-type transport system involved in cytochrome c biogenesis, ATPase component	O	507	788	1296
COG0409	Hydrogenase maturation factor HypD	O	500	1459	1959
COG3222	Uncharacterized conserved protein, glycosyltransferase A (GT-A) superfamily, DUF2064 family	S	130	165	295

COG1595	DNA-directed RNA polymerase specialized sigma subunit, sigma24 family	K	84896	92822	177719
COG2859	Outer membrane channel-forming protein BP26/OMP28, SIMPL family	M	1878	2256	4134
COG1003	Glycine cleavage system protein P (pyridoxal-binding), C-terminal domain	E	1319	2124	3443
COG4720	ECF-type riboflavin transporter, membrane (S) component	H	6245	6558	12804
COG2968	Uncharacterized conserved protein YggE, contains kinase-interacting SIMPL domain	S	2824	2794	5618
COG0096	Ribosomal protein S8	J	3377	4280	7657
COG0274	Deoxyribose-phosphate aldolase	F	6592	8247	14840
COG4995	Uncharacterized conserved protein, contains CHAT domain	S	1585	1700	3285
COG4864	Uncharacterized conserved protein YqfA, UPF0365 family	S	1545	2417	3961
COG1041	tRNA G10 N-methylase Trm11	J	2478	2607	5084
COG0823	Periplasmic component TolB of the Tol biopolymer transport system	U	3043	4242	7285
COG0667	Pyridoxal reductase PdxI or related oxidoreductase, aldo/keto reductase family	H;R	8493	10005	18498
COG1872	Uncharacterized conserved protein YggU, UPF0235/DUF167 family	S	88	348	437
COG3800	Predicted transcriptional regulator	R	0	2	2
COG0474	Magnesium-transporting ATPase (P-type)	P	24493	33412	57905
COG3051	Citrate lyase, alpha subunit	C	932	1357	2289
COG0725	ABC-type molybdate transport system, periplasmic Mo-binding protein ModA	P	717	1487	2204
COG1525	Endonuclease YncB, thermonuclease family	L	1252	2561	3813
COG1024	Enoyl-CoA hydratase/carnithine racemase	I	1293	4604	5897
COG0304	3-oxoacyl-(acyl-carrier-protein) synthase	I;Q	9513	13211	22724

COG2717	Heme-binding membrane subunit of periplasmic DMSO/TMAO and protein-methionine-sulfoxide reductases	C	23	62	85
COG1663	Tetraacyldisaccharide-1-P 4'-kinase (Lipid A 4'-kinase)	M	2835	3653	6488
COG5421	Transposase	X	35512	68046	103558
COG2947	Predicted RNA-binding protein, contains EVE domain	R	126	393	519
COG2414	Aldehyde:ferredoxin oxidoreductase	C	507	1132	1639
COG5036	SPX domain-containing protein involved in vacuolar polyphosphate accumulation	P;U	4059	4010	8069
COG3979	Chitodextrinase	G	2682	3209	5891
COG4116	Predicted triphosphatase or cyclase YjbK, contains CYTH domain	R	721	321	1042
COG2873	O-acetylhomoserine/O-acetylserine sulfhydrylase, pyridoxal phosphate-dependent	E	10286	11829	22114
COG0817	Holliday junction resolvasome RuvABC endonuclease subunit RuvC	L	4329	5262	9591
COG0084	3'->5' ssDNA/RNA exonuclease TatD	N	11359	14616	25975
COG1278	Cold shock protein, CspA family	K	3549	3390	6939
COG2822	Iron uptake system EfeUOB, periplasmic (or lipoprotein) component EfeO/EfeM	P	0	2	2
COG3905	Predicted transcriptional regulator, contains ribbon-helix-helix (RHH_1) domain	K	11	18	29
COG4241	Uncharacterized conserved protein YybS, DUF2232 family	S	24	150	174
COG0206	Cell division GTPase FtsZ	D	9873	12461	22334
COG0536	GTPase involved in cell partioning and DNA repair	D;L	8714	12313	21026
COG4775	Outer membrane protein assembly factor BamA	M	17298	18392	35690

COG1071	TPP-dependent pyruvate or acetoin dehydrogenase subunit alpha	C	716	1553	2269
COG1391	Glutamine synthetase adenylyltransferase	O	3	347	350
COG1996	DNA-directed RNA polymerase, subunit RPC12/RpoP, contains C4-type Zn-finger	K	6349	6436	12784
COG5527	Protein involved in initiation of plasmid replication	X	2121	2342	4464
COG0308	Aminopeptidase N, contains DUF3458 domain	E	2599	4388	6987
COG1662	Transposase and inactivated derivatives, IS1 family	X	4059	2144	6203
COG4633	Plastocyanin domain containing protein	R	0	47	47
COG4939	Membrane-anchored lipoprotein Tpp15, major membrane immunogen	S	58	20	78
COG0399	dTDP-4-amino-4,6-dideoxygalactose transaminase	M	25599	31727	57326
COG2056	Predicted histidine transporter YuiF, NhaC family	E	23	354	377
COG1190	Lysyl-tRNA synthetase (class II)	J	8251	10534	18785
COG4087	Soluble P-type ATPase	R	9	23	32
COG0444	ABC-type dipeptide/oligopeptide/nickel transport system, ATPase component	E;P	13036	26174	39210
COG4907	Uncharacterized membrane protein	S	983	2051	3034
COG2274	ABC-type bacteriocin/lantibiotic exporters, contain an N-terminal double-glycine peptidase domain	V	11647	11311	22958
COG5513	Chagasin-like inhibitor of cysteine peptidase, I42 family	O	34	98	132
COG0135	Phosphoribosylanthranilate isomerase	E	3029	3139	6168
COG5938	CRISPR-Cas system type I-B effector complex large subunit Cas8b1	V	263	114	377
COG1111	ERCC4-related helicase	L	9	2	11

COG5529	Phage-encoded DNA-binding protein ECs1768, contains HTH and DnaT DNA-binding domains	X	654	638	1292
COG4531	ABC-type Zn2+ transport system, periplasmic component/surface adhesin	P	1	2	3
COG5651	PPE-repeat protein	S	486	641	1127
COG2993	Cbb3-type cytochrome oxidase, cytochrome c subunit FixO	C	17	3	20
COG2209	Na+-transporting NADH:ubiquinone oxidoreductase, subunit NqrE	C	1248	1262	2510
COG3414	Phosphotransferase system, galactitol-specific IIB component	G	62	306	368
COG3326	Uncharacterized membrane protein YsdA, DUF1294 family	S	1367	1604	2971
COG0080	Ribosomal protein L11	J	2892	3850	6742
COG0161	Adenosylmethionine-8-amino-7-oxononanoate aminotransferase	H	1791	2755	4545
COG1236	RNA processing exonuclease, beta-lactamase fold, Cft2 family	J	4417	6702	11119
COG5302	Post-segregation antitoxin (ccd killing mechanism protein) encoded by the F plasmid	X	1	0	1
COG4666	TRAP-type uncharacterized transport system, fused permease components	R	1045	2904	3949
COG1395	Predicted transcriptional regulator	K	700	314	1014
COG5931	CRISPR-Cas system type-V protein Cas12a	V	238	109	348
COG3396	1,2-phenylacetyl-CoA epoxidase, catalytic subunit	Q	0	3	3
COG3739	Uncharacterized membrane protein YoaT, DUF817 family	S	2	2	4
COG3752	Steroid 5-alpha reductase family enzyme	R	2227	2051	4278

COG2055	Malate/lactate/ureidoglycolate dehydrogenase, LDH2 family	C	1028	2025	3053
COG1193	dsDNA-specific endonuclease/ATPase MutS2	L	13992	17488	31481
COG0414	Panthothenate synthetase	H	3240	3920	7160
COG3260	Ni,Fe-hydrogenase III small subunit	C	180	242	422
COG0521	Molybdopterin biosynthesis enzyme MoaB/MogA	H	720	1607	2328
COG3114	Heme exporter protein D	U	0	15	15
COG0400	Predicted esterase	R	15	135	150
COG1294	Cytochrome bd-type quinol oxidase, subunit 2	C	1826	2708	4534
COG0302	GTP cyclohydrolase I	H	3609	3518	7128
COG1978	Predicted RNase H-related nuclease YkuK, DUF458 family	R	15	11	26
COG1890	Ribosomal protein S3AE	J	12	9	21
COG1112	Superfamily I DNA and/or RNA helicase	L	9207	11627	20834
COG2230	Cyclopropane fatty-acyl-phospholipid synthase and related methyltransferases	I	1272	1807	3079
COG0289	4-hydroxy-tetrahydrodipicolinate reductase	E	6954	8414	15368
COG1981	Protoporphyrinogen oxidase HemJ (unrelated to HemG or HemY)	H	9	2	11
COG1344	Flagellin and related hook-associated protein FlgL	N	23951	19146	43098
COG2100	Uncharacterized Fe-S cluster-containing enzyme, radical SAM superfamily	R	1	23	24
COG0062	NAD(P)H-hydrate repair enzyme Nnr, NAD(P)H-hydrate epimerase domain	F	7856	9026	16883
COG1760	L-serine deaminase	E	4766	7873	12639
COG4694	Wobble nucleotide-excising tRNase	J	2345	2786	5131
COG1519	3-deoxy-D-manno-octulosonic-acid transferase	M	2213	3492	5705

COG1686	D-alanyl-D-alanine carboxypeptidase	M	26235	33344	59578
COG2004	Ribosomal protein S24E	J	1	17	18
COG3882	Predicted enzyme involved in methoxymalonyl-ACP biosynthesis	I	3118	3798	6916
COG3772	Phage-related lysozyme (muramidase), GH24 family	M	2217	3840	6057
COG3381	Cytoplasmic chaperone TorD involved in molybdoenzyme TorA maturation	O	147	270	418
COG4654	Cytochrome c551/c552	C	0	166	166
COG3695	Alkylated DNA nucleotide flippase Atl1, participates in nucleotide excision repair, Ada-like DNA-binding domain	K	1639	1644	3282
COG2307	Uncharacterized conserved protein, Alpha-E superfamily	S	560	690	1251
COG4799	Acetyl-CoA carboxylase, carboxyltransferase component	I	7414	8532	15947
COG3480	Predicted secreted protein YlbL, contains PDZ domain	T	276	390	666
COG2979	Uncharacterized membrane protein YebE, DUF533 family	S	18	13	31
COG0620	Methionine synthase II (cobalamin-independent)	E	247	729	976
COG4858	Uncharacterized membrane-anchored	S	1	184	185
COG0301	Adenylyl- and sulfurtransferase ThiI (thiamine and tRNA 4-thiouridine	H;J	6616	8083	14699
COG4690	Dipeptidase	E	3756	5163	8919
COG1751	Ligand-binding C-terminal domain of pyruvate kinase, DUF1867	G;T	3	34	38
COG2846	Iron-sulfur cluster repair protein YtfE, RIC family, contains ScdAN and hemerythrin domains	O	1832	1846	3678

COG3530	Uncharacterized conserved protein, DUF3820 family	S	57	220	277
COG4536	Mg2+ and Co2+ transporter CorB, contains DUF21, CBS pair, and CorC-HlyC domains	P	3284	4279	7563
COG3964	Predicted amidohydrolase	R	209	879	1088
COG0401	Membrane potential modulator PMP3/YqaE, plasma membrane proteolipid 3 (PMP3)/Esi3/RCI2/UPF0057 family	C	0	73	73
COG3074	Cell division protein ZapB, interacts with FtsZ	D	15	1	16
COG0636	FoF1-type ATP synthase, membrane subunit c/Archaeal/vacuolar-type H+-ATPase, subunit K	C	3262	3703	6964
COG0659	Sulfate permease or related transporter, MFS superfamily	P	2853	4687	7540
COG1113	L-asparagine transporter or related	E	21	401	422
COG4333	Uncharacterized conserved protein, DUF1643 domain	S	924	842	1766
COG2081	Predicted flavoprotein YhiN	R	12465	14841	27306
COG3026	RseB, negative regulator of sigma E activity	T	0	3	3
COG2010	Cytochrome c, mono- and diheme variants	C	497	1012	1509
COG2242	Precorrin-6B methylase 2	H	1834	1682	3516
COG3173	Predicted kinase, aminoglycoside phosphotransferase (APT) family	R	2059	3241	5300
COG5807	Sporulation sensor histidine kinase C	D;T	78	294	372
COG1495	Disulfide bond formation protein DsbB	O	2	4	5
COG0074	Succinyl-CoA synthetase, alpha subunit	C	415	1585	2000
COG0858	Ribosome-binding factor RbfA	J	4060	4894	8954
COG4260	Membrane protease subunit, stomatin/prohibitin family, contains C-terminal Zn-ribbon domain	O	8332	9040	17372

COG2375	NADPH-dependent ferric siderophore reductase, contains FAD-binding and SIP domains	P	0	1	1
COG2323	Uncharacterized membrane protein YcaP, DUF421 family	S	4820	5956	10777
COG0430	RNA 3'-terminal phosphate cyclase	A	1	2	3
COG5126	Ca2+-binding protein, EF-hand superfamily	T	2	4	6
COG0131	Imidazoleglycerol phosphate dehydratase HisB	E	3053	4005	7058
COG1937	DNA-binding transcriptional regulator, FrmR family	K	2169	2810	4979
COG1979	Alcohol dehydrogenase YqhD, Fe-dependent ADH family	C	5871	8122	13993
COG5566	Transcriptional regulator, middle operon regulator (Mor) family	K;X	802	915	1717
COG1377	Flagellar biosynthesis protein FlhB	N	3001	3036	6037
COG2452	Predicted site-specific integrase-resolvase	X	241	497	738
COG1471	Ribosomal protein S4E	J	8	8	15
COG5924	Uncharacterized sporulation protein YtfI, contains DUF2953 domain	D	2	2	4
COG3585	Molybdopterin-binding protein MopI	H	21	48	69
COG4605	ABC-type enterochelin transport system, permease component	P	77	150	227
COG0677	UDP-N-acetyl-D-mannosaminuronate dehydrogenase	M	4937	6909	11846
COG2345	Predicted transcriptional regulator, ArsR family	K	19	158	177
COG4817	DNA-binding ferritin-like protein (Dps family)	L	72	33	105
COG3824	Predicted Zn-dependent protease, minimal metalloprotease (MMP)-like domain	O	6	9	15

COG5490	Predicted polyhydroxyalkanoate (PHA)-binding protein, phasin family	S	4	3	7
COG1426	Cytoskeletal protein RodZ, contains Xre-like HTH and DUF4115 domains	D	1635	1783	3418
COG0105	Nucleoside diphosphate kinase	F	904	1499	2404
COG4898	Uncharacterized conserved protein	S	156	310	467
COG0723	Rieske Fe-S protein	C	25	17	43
COG3301	Nitrite/polysulfide reductase, membrane component NrfD/PsrC	P	99	585	684
COG1706	Flagellar basal body P-ring protein FlgI	N	45	195	240
COG3493	Na+/citrate or Na+/malate symporter	C	284	364	647
COG0852	NADH:ubiquinone oxidoreductase 27 kD subunit (chain C)	C	396	730	1126
COG0840	Methyl-accepting chemotaxis protein (MCP)	T	52245	42448	94693
COG3898	Uncharacterized protein HemY, contains HemY_N domain and TPR repeats (unrelated to HemY-type protoporphyrinogen oxidase)	S	4	49	53
COG2869	Na+-transporting NADH:ubiquinone oxidoreductase, subunit NqrC	C	1257	1305	2561
COG2998	ABC-type tungstate transport system, permease component TupA	P	222	465	687
COG4267	Uncharacterized membrane protein	S	822	1257	2079
COG3570	Streptomycin 6-kinase	V	394	362	756
COG4973	Site-specific recombinase XerC	L	7237	7358	14595
COG1804	Crotonobetainyl-CoA:carnitine CoA-transferase CaiB and related acyl-CoA transferases	I	1525	3898	5423
COG2954	CYTH domain, found in class IV adenylate cyclase and various triphosphatases	R	1918	2641	4559
COG2166	Sulfur transfer protein SufE, Fe-S cluster assembly	O	1166	1563	2730

COG1858	Cytochrome c peroxidase	O	309	856	1164
COG1582	Swarming motility protein SwrD	N	433	294	726
COG2819	Predicted hydrolase of the alpha/beta superfamily	R	3155	4021	7177
COG0318	O-succinylbenzoic acid-CoA ligase MenE or related acyl-CoA synthetase (AMP-forming)	I	16162	21680	37842
COG5901	Spore germination receptor GerABC, GerA subunit (L-alanine receptor)	D	5607	7483	13090
COG0348	Polyferredoxin NapH	C	5129	6715	11844
COG1876	LD-carboxypeptidase LdcB, LAS superfamily	M	9261	10060	19322
COG1886	Flagellar motor switch/type III secretory pathway protein FliN	N;U	129	250	378
COG4884	Uncharacterized conserved protein YfeS, contains WGR domain	S	0	5	5
COG1092	23S rRNA G2069 N7-methylase RlmK or C1962 C5-methylase RlmI	J	13498	16700	30198
COG4262	Predicted spermidine synthase with an N-terminal membrane domain	R	250	407	657
COG3278	Cbb3-type cytochrome oxidase, subunit 1	C	23	4	27
COG0288	Carbonic anhydrase	P	645	1270	1915
COG0219	tRNA(Leu) C34 or U34 (ribose-2'-O)-methylase TrmL, contains SPOUT domain	J	3967	5186	9153
COG4565	DNA-binding response regulator DpiB of citrate/malate metabolism	K;T	221	354	575
COG3773	Cell wall hydrolase CwlJ, involved in spore germination	D;M	7990	10803	18793
COG4115	Toxin component of the Txe-Axe toxin-antitoxin module, Txe/YoeB family	V	1278	1528	2806
COG1122	Energy-coupling factor transporter ATP-binding protein EcfA2	P;R	11199	15356	26555

COG5646	Iron-binding protein Fra/YdhG, frataxin family (Fe-S cluster biosynthesis)	O;P	446	712	1158
COG1593	TRAP-type C4-dicarboxylate transport system, large permease component	G	2905	9471	12377
COG3797	Uncharacterized conserved protein, DUF1697 family	S	294	423	717
COG1351	Thymidylate synthase ThyX, FAD-dependent family	F	3093	9858	12951
COG3634	Alkyl hydroperoxide reductase subunit	V	36	122	158
COG1049	Aconitase B	C	19	8	28
COG0828	Ribosomal protein S21	J	430	653	1083
COG1160	Double Era-like domain GTPase Der	J	12309	16026	28335
COG5849	SigmaK checkpoint regulator BofC (Bypass-of-forespore protein C)	D;T	1	57	58
COG1107	Archaea-specific RecJ-like exonuclease, contains DnaJ-type Zn finger domain	L	0	1	1
COG0606	Predicted Mg-chelatase, contains ChlI-like and ATPase domains, YifB family	O	10779	14151	24930
COG5298	Predicted metal-dependent carbohydrate esterase YdaL, contains NodB-like catalytic (CE4) domain	R	3	2	5
COG0449	Glucosamine 6-phosphate synthetase, contains amidotransferase and phosphosugar isomerase domains	M	14310	17288	31598
COG4576	Carboxysome shell and ethanolamine utilization microcompartment protein CcmK/EutM	Q;C	283	518	801
COG3554	Uncharacterized conserved protein	S	10	31	42
COG2928	Uncharacterized membrane protein	S	6	134	140
COG2207	AraC-type DNA-binding domain and AraC-containing proteins	K	76977	92145	169122
COG1397	ADP-ribosylglycohydrolase	O	5930	7202	13132

COG4021	tRNA(His) 5'-end guanylyltransferase	J	310	682	992
COG1123	ABC-type glutathione transport system ATPase component, contains duplicated ATPase domain	O	10991	14776	25767
COG4332	Uncharacterized conserved protein, DUF1062 domain	S	352	465	817
COG3302	DMSO reductase anchor subunit DmsC	C	67	173	240
COG0538	Isocitrate dehydrogenase	C	3645	4990	8636
COG2733	Uncharacterized membrane-anchored protein YjiN, DUF445 family	S	7	181	188
COG3091	Predicted Zn-dependent metalloprotease, SprT family	R	158	701	860
COG0100	Ribosomal protein S11	J	2038	2786	4824
COG0390	ABC-type iron transport system FetAB, permease component	P	95	321	416
COG1717	Ribosomal protein L32E	J	6	2	8
COG4305	Peptidoglycan-binding domain, expansin YoaJ	M	155	167	323
COG3415	CRISPR-associated protein Csa3, CARF domain	V	41189	49227	90416
COG2027	D-alanyl-D-alanine carboxypeptidase	M	1311	1841	3152
COG3360	Flavin-binding protein dodecin	R	24	51	75
COG3819	Uncharacterized membrane protein	S	28	19	47
COG2850	Ribosomal protein L16 Arg81 hydroxylase, contains JmjC domain	J	5	12	17
COG3382	B3/B4 domain (DNA/RNA-binding domain of Phe-tRNA-synthetase)	R	547	971	1518
COG1033	Predicted exporter protein, RND	R	4560	6481	11041
COG2314	Uncharacterized membrane protein YozV, TM2 domain, contains pTyr	R	3085	2874	5959
COG1619	Muramoyltetrapeptide carboxypeptidase LdcA (peptidoglycan recycling)	M	5597	7083	12680

COG3682	Transcriptional regulator, CopY/TcrY	K	10612	11682	22294
COG3049	Penicillin V acylase or related amidase, Ntn superfamily	M;R	9527	10080	19607
COG3545	Predicted esterase of the alpha/beta hydrolase fold	R	231	458	690
COG1454	Alcohol dehydrogenase, class IV	C	11468	15989	27458
COG0758	Predicted Rossmann fold nucleotide-binding protein DprA/Smf involved in DNA uptake	L	12757	14101	26857
COG5854	Small acid-soluble spore protein SspF (alpha/beta-type)	D	9	30	38
COG0209	Ribonucleotide reductase alpha subunit	F	4073	8246	12319
COG1431	PIWI domain, catalyzes dsRNA-guided hydrolysis of ssRNA, involved in RNA silencing, RNA metabolism and antiviral defense	J;V	385	624	1009
COG5735	Photosystem II reaction center protein Z, PsbZ/Ycf9	C	2	2	4
COG1409	3',5'-cyclic AMP phosphodiesterase CpdA	T	14434	20257	34691
COG5009	Membrane carboxypeptidase/penicillin-binding protein	M	2945	4582	7527
COG2851	Mg2+/citrate symporter	C	313	714	1027
COG1027	Aspartate ammonia-lyase	E	1436	1941	3377
COG1207	Bifunctional protein GlmU, N-acetylglucosamine-1-phosphate-uridyltransferase/glucosamine-1-phosphate-acetyltransferase	M	8357	9985	18342
COG0007	Uroporphyrinogen-III methylase (siroheme synthase)	H	1551	3889	5440
COG0031	Cysteine synthase	E	8729	11712	20440
COG0136	Aspartate-semialdehyde dehydrogenase	E	7593	9794	17387
COG0706	Membrane protein insertase Oxa1/YidC/SpoIIIJ	M	13354	16027	29381

COG5361	Uncharacterized conserved protein	X	13	5	19
COG2065	Pyrimidine operon attenuation protein PyrR/uracil phosphoribosyltransferase	F	1089	1350	2439
COG0095	Lipoate-protein ligase A	H	1621	2607	4227
COG0397	Protein adenylyltransferase (AMPylase) SelO/YdiU (selenoprotein O)	O	21	11	32
COG5824	Stage III sporulation protein SpoIIIAC, component of the engulfment complex	D	529	571	1100
COG3259	Coenzyme F420-reducing hydrogenase, alpha subunit	C	102	98	199
COG1420	Transcriptional regulator of heat shock response	K	6347	8000	14348
COG2964	Predicted transcriptional regulator YheO, contains PAS and DNA-binding HTH domains	K	821	2174	2995
COG4127	Predicted restriction endonuclease, Mrr-cat superfamily	R	129	210	339
COG1589	Cell division septal protein FtsQ	D	7758	8922	16680
COG5448	Uncharacterized conserved protein, DUF2460 domain	S	20	34	53
COG1946	Acyl-CoA thioesterase	I	9	12	22
COG1342	Predicted DNA-binding protein, UPF0251 family	R	948	1242	2190
COG3755	Uncharacterized conserved protein YecT, DUF1311 family	S	960	1082	2042
COG5545	Predicted P-loop ATPase and inactivated derivatives	X	5598	7688	13287
COG2972	Sensor histidine kinase YesM	T	18821	26214	45035
COG4178	ABC-type uncharacterized transport system, permease and ATPase components	R	11	211	223

COG2337	mRNA-degrading endonuclease MazF, toxin component of the MazEF toxin-antitoxin module	V	5627	7419	13046
COG5852	Small acid-soluble spore protein SspA/SspB/SspC/SspD (alpha/beta-type)	D	560	717	1277
COG1017	Hemoglobin-like flavoprotein	C	2	11	12
COG4553	Poly-beta-hydroxyalkanoate depolymerase	I	17	4	21
COG1252	NADH dehydrogenase, FAD-containing subunit	C	512	894	1407
COG0087	Ribosomal protein L3	J	5773	7527	13300
COG2719	Stage V sporulation protein SpoVR/YcgB, involved in spore cortex formation (function unknown)	D	0	3	3
COG3646	Phage regulatory protein Rha	X	2924	2341	5265
COG3463	Uncharacterized membrane protein	S	813	749	1562
COG3624	Alpha-D-ribose 1-methylphosphonate 5-triphosphate synthase subunit PhnG	P	65	14	79
COG5620	Uncharacterized conserved protein, DUF1851 domain	S	1008	263	1271
COG3183	Predicted restriction endonuclease, HNH family	V	923	1125	2048
COG2113	ABC-type proline/glycine betaine transport system, periplasmic component	E	15	142	157
COG3583	Uncharacterized conserved protein YabE, contains G5 and tandem DUF348 domains	S	3383	3411	6794
COG1715	Restriction endonuclease Mrr	V	2697	3250	5947
COG3627	Alpha-D-ribose 1-methylphosphonate 5-phosphate C-P lyase PhnJ	P	105	37	141
COG2518	Protein-L-isoaspartate O-methyltransferase	O	229	411	640
COG4128	Zona occludens toxin, predicted ATPase	R	703	904	1607
COG4252	Extracytoplasmic sensor domain CHASE2 (specificity unknown)	T	73	159	232

COG4923	Predicted nuclease (RNAse H fold)	R	5	34	39
COG4731	Uncharacterized conserved protein, DUF2147 family	S	139	64	203
COG3826	Uncharacterized conserved protein	S	1	2	3
COG1448	Aspartate/aromatic aminotransferase	E	251	530	781
COG3615	Tellurite resistance protein TehB, SAM-dependent methylase, cupin superfamily	P	0	29	29
COG3117	Lipopolysaccharide export system protein LptC	M	733	924	1658
COG5342	Invasion protein IalB, involved in pathogenesis	R	0	57	57
COG1031	Radical SAM superfamily enzyme with C-terminal helix-hairpin-helix motif	R	11	62	73
COG4325	Uncharacterized membrane protein	S	5	11	17
COG1134	ABC-type polysaccharide/polyol phosphate transport system, ATPase component	G	7013	9073	16085
COG0770	UDP-N-acetylmuramyl pentapeptide synthase	M	14210	17886	32096
COG0153	Galactokinase	G	9977	11466	21443
COG1410	Methionine synthase I, cobalamin-binding domain	E	2712	3273	5986
COG2813	16S rRNA G1207 methylase RsmC	J	757	990	1747
COG5265	ABC-type transport system involved in Fe-S cluster assembly, permease and ATPase components	O	20	46	66
COG0228	Ribosomal protein S16	J	2914	3133	6047
COG0766	UDP-N-acetylglucosamine enolpyruvyl transferase	M	13896	18600	32496
COG1206	Folate-dependent tRNA-U54 methylase TrmFO/GidA	J	5287	6406	11693
COG5483	Uncharacterized conserved protein, DUF488 family	S	752	927	1679

COG2260	rRNA maturation protein Nop10, contains Zn-ribbon domain	J	9	22	31
COG3608	Predicted deacylase	R	4187	3537	7724
COG3783	Soluble cytochrome b562	C	0	1	1
COG0283	Cytidylate kinase	F	6505	8210	14715
COG2723	Beta-glucosidase/6-phospho-beta-glucosidase/beta-galactosidase	G	5438	8406	13844
COG4461	Lysozyme inhibitor LprI	V	98	267	366
COG5438	Uncharacterized membrane protein	S	2159	2445	4604
COG1444	tRNA(Met) C34 N-acetyltransferase TmcA	J	357	394	751
COG3942	Surface antigen	M	790	522	1312
COG3949	Uncharacterized membrane protein YkvI	S	637	2122	2759
COG4725	N6-adenosine-specific RNA methylase IME4	J	2837	4123	6960
COG1350	Predicted alternative tryptophan synthase beta-subunit (paralog of TrpB)	E	2430	3879	6309
COG1815	Flagellar basal body rod protein FlgB	N	1407	1295	2702
COG5375	Uncharacterized conserved protein	S	2	125	126
COG1237	Metal-dependent hydrolase, beta-lactamase superfamily II	R	2718	3295	6013
COG3007	Trans-2-enoyl-CoA reductase	I	1275	590	1865
COG3599	Cell division septum initiation protein DivIVA, interacts with FtsZ and MinD	D	3226	3578	6803
COG3440	Predicted restriction endonuclease	V	1309	2002	3312
COG1256	Flagellar hook-associated protein FlgK	N	6997	6499	13496
COG2049	5-oxoprolinase subunit B/Allophanate hydrolase subunit 1	E	181	616	797
COG4631	Xanthine dehydrogenase, molybdopterin-binding subunit XdhB	F	2	4	6
COG5340	Abortive phage infection protein AbiEi, antitoxin conponent of an AbiEi-AbiEii TA system	V	6826	8840	15666

COG4095	Sugar transporter, SemiSWEET family, contains PQ motif	G	254	209	463
COG1906	Predicted GntP-related membrane permease AF0261, DUF401 family	R	11	291	302
COG3590	Predicted metalloendopeptidase	O	4223	4595	8818
COG5387	Mitochondrial FoF1-type ATP synthase assembly chaperone ATP12	O	1	44	45
COG2258	N-hydroxylaminopurine reductase YiiM, contains MOSC domain	F;V	232	419	651
COG1149	MinD superfamily P-loop ATPase, contains an inserted ferredoxin domain	R	3537	4250	7787
COG3517	Predicted component TssB of the type VI protein secretion system, VipA/VipB/TssB family	U	16	8	24
COG2122	Uncharacterized conserved protein, UPF0280 family, ApbE superfamily	S	83	93	176
COG1372	Intein/homing endonuclease	L;X	161	246	407
COG1882	Pyruvate-formate lyase	C	7783	13757	21539
COG0482	tRNA U34 2-thiouridine synthase MnmA/TrmU, contains the PP-loop ATPase domain	J	10476	13230	23707
COG0441	Threonyl-tRNA synthetase	J	13732	18803	32535
COG4360	ATP adenylyltransferase (5',5'''-P-1,P-4-tetraphosphate phosphorylase II)	F	478	698	1176
COG4492	ACT domain-containing protein, UPF0735 family	R	1334	1971	3305
COG3726	Uncharacterized membrane protein Smp affecting hemolysin expression	S	0	6	6
COG5434	Polygalacturonase	G	14934	15480	30414
COG1600	Epoxyqueuosine reductase QueG (queuosine biosynthesis)	J	3805	4321	8126

COG0819	Aminopyrimidine aminohydrolase TenA (thiamine salvage pathway)	H	1162	1808	2970
COG5841	Stage V sporulation protein SpoVB/SpoIIIF, required for spore cortex	D	15057	19737	34794
COG0048	Ribosomal protein S12	J	1652	2194	3846
COG0198	Ribosomal protein L24	J	2940	3930	6870
COG0690	Preprotein translocase subunit SecE	U	1880	2198	4078
COG0322	Excinuclease UvrABC, nuclease subunit	L	12396	16441	28836
COG2999	Glutaredoxin 2	O	0	3	3
COG5480	Uncharacterized membrane protein	S	3	3	5
COG0788	Formyltetrahydrofolate hydrolase	F	207	222	429
COG1529	Aldehyde, CO or xanthine dehydrogenase, Mo-binding subunit	C	1746	5489	7235
COG2372	Copper-binding protein CopC (methionine-rich)	P	1	1	2
COG1359	Quinol monooxygenase YgiN	C	1092	1745	2837
COG3862	Uncharacterized conserved protein with two CxxC motifs	S	697	1078	1775
COG3653	N-acyl-D-aspartate/D-glutamate deacylase	Q	419	1737	2156
COG0436	Aspartate/methionine/tyrosine aminotransferase	E	26597	36328	62924
COG4676	Uncharacterized conserved protein YfaP, DUF2135 family	S	1325	1827	3152
COG0599	Uncharacterized conserved protein YurZ, alkylhydroperoxidase/carboxymuconolacton e decarboxylase family	R	1650	1955	3606
COG5727	Photosystem II reaction center protein M, PsbM	C	2	0	2
COG2840	DNA-nicking endonuclease, Smr domain	L	30	208	238
COG3501	Uncharacterized conserved protein VgrG, implicated in type VI secretion and phage assembly	U;X;R	3579	3757	7336

COG1455	Phosphotransferase system cellobiose-specific component IIC	G	670	2514	3184
COG3975	Predicted metalloprotease, contains C-terminal PDZ domain	R	47	178	225
COG2146	Ferredoxin subunit of nitrite reductase or a ring-hydroxylating dioxygenase	P;Q	132	194	326
COG1148	Heterodisulfide reductase, subunit A (polyferredoxin)	C	2400	2769	5169
COG1652	Cytoplasmic potassium-binding protein Kbp/XkdP/YgaU, contains LysM domain	P	6661	7821	14482
COG5371	Nucleoside diphosphatase, GDA1/CD39 family	F	239	113	352
COG4125	Uncharacterized membrane protein	S	0	1	1
COG0575	CDP-diglyceride synthetase	I	7998	10039	18037
COG2900	Uncharacterized coiled-coil protein SlyX (sensitive to lysis X)	S	119	67	185
COG0381	UDP-N-acetylglucosamine 2-epimerase	M	8198	11591	19789
COG4938	Predicted ATPase	R	2694	2997	5691
COG2104	Sulfur carrier protein ThiS (thiamine biosynthesis)	H	263	492	755
COG4989	Predicted oxidoreductase YdhF	R	383	740	1123
COG3744	PIN domain nuclease, a component of toxin-antitoxin system (PIN domain)	V	438	546	984
COG2102	Diphthamide synthase (EF-2-diphthine--ammonia ligase)	J	9	36	45
COG3858	Spore cortex hydrolase SleL/YaaH, N-acetylglucosaminidase of GH18 family	D	9172	11481	20654
COG1623	c-di-AMP synthetase DisA, contains DisA_N, linker and DNA-binding domains	T	256	616	873
COG3846	Type IV secretory pathway, TrbL components	U	33	137	170

COG2974	DNA recombination-dependent growth factor RdgC	L	26	47	74
COG4427	Uncharacterized conserved protein, DUF2332 domain	S	3	0	3
COG5871	Spore cortex protein YqfC, YabP family	D	383	740	1123
COG0558	Phosphatidylglycerophosphate synthase	I	8801	10557	19358
COG1575	1,4-dihydroxy-2-naphthoate polyprenyltransferase	H	2611	3800	6412
COG3073	RseA, negative regulator of sigma E activity	T	45	124	169
COG3661	Alpha-glucuronidase	G	1273	1085	2358
COG0187	DNA gyrase/topoisomerase IV, subunit B	L	21172	28870	50042
COG2137	SOS response regulatory protein OraA/RecX, interacts with RecA	O	5866	6712	12577
COG1289	Uncharacterized membrane protein YccC	S	10	51	61
COG3170	Type IV pilus assembly protein FimV	N;W	1878	2250	4128
COG1379	PHP family phosphoesterase with a Zn ribbon	R	270	525	795
COG2426	Uncharacterized membrane protein	S	1947	3052	4999
COG3660	Mitochondrial fission protein ELM1	D	289	832	1121
COG1301	Na+/H+-dicarboxylate symporter	C	3263	6563	9826
COG5713	Photosystem I assembly protein Ycf3, contains TPR repeats	C	7	5	12
COG4288	Uncharacterized conserved protein	S	154	175	329
COG1099	Predicted metal-dependent hydrolase, TIM-barrel fold	R	0	484	484
COG1304	FMN-dependent dehydrogenase, includes L-lactate dehydrogenase and type II isopentenyl diphosphate isomerase	C;I;R	975	2113	3088
COG0632	Holliday junction resolvasome RuvABC DNA-binding subunit	L	6425	7849	14273
COG5864	Small acid-soluble spore protein SAS2, clostridial type	D	43	190	232

COG1722	Exonuclease VII small subunit	L	1150	1759	2908
COG4132	ABC-type uncharacterized transport system, permease component	R	16	65	81
COG3844	Kynureninase	E	10	14	23
COG2516	Biotin synthase-related protein, radical SAM superfamily	R	419	661	1080
COG3857	ATP-dependent helicase/DNAse subunit B	L	15060	19699	34759
COG3945	Hemerythrin domain	R	102	101	203
COG2133	Glucose/arabinose dehydrogenase, beta-propeller fold	G	441	492	933
COG2239	Mg/Co/Ni transporter MgtE (contains CBS domain)	P	6787	8493	15280
COG3538	Meiotically up-regulated gene 157 (Mug157) protein (function unknown)	S	1082	1863	2945
COG2854	Periplasmic subunit MlaC of the ABC-type intermembrane phospholipid transporter Mla	M	21	248	269
COG3534	Alpha-L-arabinofuranosidase	G	8731	11148	19878
COG3623	L-ribulose-5-phosphate 3-epimerase UlaE	G	229	512	741
COG5007	Acid stress protein IbaG/YrbA, BolA-like family	T	0	1	1
COG1034	NADH dehydrogenase/NADH:ubiquinone oxidoreductase 75 kD subunit (chain G)	C	457	1021	1478
COG1421	CRISPR-Cas system type III CSM-effector complex small subunit Csm2	V	50	158	208
COG3543	Uncharacterized conserved protein, DUF1284 domain	S	531	402	932
COG1223	Predicted ATPase, AAA+ superfamily	R	268	480	748
COG3847	Flp pilus assembly protein, pilin Flp	U;W	4	9	13
COG2313	Pseudouridine-5'-phosphate glycosidase (pseudoU degradation)	F	1294	1705	2999
COG0569	Trk/Ktr K+ transport system regulatory component TrkA/KtrA/KtrC, RCK domain	P;T	17713	21177	38890

COG4877	Uncharacterized conserved protein	S	923	755	1678
COG3285	Eukaryotic-type DNA primase	L	146	261	407
COG1641	CTP-dependent cyclometallase, nickel-pincer nucleotide (NPN) cofactor biosynthesis	H	2464	4363	6827
COG0561	Hydroxymethylpyrimidine pyrophosphatase and other HAD family phosphatases	H;R	18641	25741	44382
COG5659	SRSO17 transposase	X	8906	8940	17846
COG2060	K+-transporting ATPase, KdpA subunit	P	125	267	392
COG4945	Carbohydrate-binding DOMON domain	G;T	316	279	594
COG5146	Pantothenate kinase	H	187	376	563
COG2885	Outer membrane protein OmpA and related peptidoglycan-associated (lipo)proteins	M	28862	37315	66177
COG1797	Cobyrinic acid a,c-diamide synthase	H	3810	4243	8053
COG2740	Nucleoid-associated protein YlxR, Predicted RNA-binding, DUF448 family	R	1734	2107	3840
COG4112	Predicted phosphoesterase YmaB, NUDIX family	R	168	406	574
COG5517	3-phenylpropionate/cinnamic acid dioxygenase, small subunit	Q	3	5	8
COG3405	Endo-1,4-beta-D-glucanase Y	G	1215	1379	2594
COG0106	Phosphoribosylformimino-5-aminoimidazole carboxamide ribonucleotide (ProFAR) isomerase	E	4093	5314	9407
COG0319	ssRNA-specific RNase YbeY, 16S rRNA maturation enzyme	J	5140	6389	11529
COG0011	Thiamin-binding stress-response protein YqgV, UPF0045 family	H	84	135	219
COG1445	Phosphotransferase system fructose-specific component IIB	G	41	47	88
COG1781	Aspartate carbamoyltransferase, regulatory subunit	F	1413	2170	3583

COG3937	Polyhydroxyalkanoate synthesis regulator phasin	Q;T	47	76	123
COG4892	Predicted heme/steroid binding protein	R	0	15	15
COG0092	Ribosomal protein S3	J	6769	8460	15228
COG1174	ABC-type proline/glycine betaine transport system, permease component	E	247	773	1020
COG2827	Predicted endonuclease, GIY-YIG superfamily	L	636	1052	1688
COG1487	Ribonuclease/mRNA interferase VapC, contains PIN domain	V	457	520	978
COG2072	Predicted flavoprotein CzcO associated with the cation diffusion facilitator CzcD	P	22	36	58
COG1189	Predicted rRNA methylase YqxC, contains S4 and FtsJ domains	J	4930	6207	11137
COG5833	Stage IV sporulation protein SpoIVFA, regulates SpoIVFB	D	485	789	1274
COG1843	Flagellar hook-capping protein FlgD	N	3959	3448	7407
COG3030	FxsA protein affecting phage T7 exclusion by the F plasmid, UPF0716 family	R	0	0	0
COG2882	Flagellar biosynthesis chaperone FliJ	N	1016	1085	2101
COG2112	Predicted Ser/Thr protein kinase	T	3	0	3
COG1817	Predicted glycosyltransferase	R	176	82	258
COG2755	Lysophospholipase L1 or related esterase. Includes spore coat protein LipC/YcsK	D;I	47854	48933	96787
COG1677	Flagellar hook-basal body complex protein FliE	N	959	971	1931
COG5322	Predicted amino acid dehydrogenase	R	2	158	160
COG0534	Na+-driven multidrug efflux pump, DinF/NorM/MATE family	V	89814	113398	203213
COG1154	Deoxyxylulose-5-phosphate synthase	H;I	12305	15080	27385
COG0285	Folylpolyglutamate synthase/Dihydropteroate synthase	H	10248	12226	22473

COG2138	Sirohydrochlorin ferrochelatase	H	1217	1803	3019
COG2159	5-carboxyvanillate decarboxylase LigW (lignin degradation), amidohydro domain	G	4641	6213	10854
COG2821	Membrane-bound lytic murein transglycosylase	M	58	584	642
COG2148	Sugar transferase involved in LPS biosynthesis (colanic, teichoic acid)	M	18112	25166	43278
COG3127	Predicted ABC-type transport system involved in lysophospholipase L1 biosynthesis, permease component	Q	8002	8524	16526
COG5680	Cytochrome b6f complex subunit V, PetG	C	3	1	4
COG5031	Ubiquinone biosynthesis protein Coq4	H	3	2	5
COG4382	Mu-like prophage protein gp16	X	6	23	30
COG4713	Uncharacterized membrane protein	S	1138	2305	3443
COG0190	5,10-methylene-tetrahydrofolate dehydrogenase/Methenyl tetrahydrofolate cyclohydrolase	H	8167	10906	19073
COG3452	Extracellular (periplasmic) sensor domain CHASE (specificity unknown)	T	0	2	2
COG0072	Phenylalanyl-tRNA synthetase beta subunit	J	11977	15918	27895
COG4776	Exoribonuclease II	K	0	9	9
COG4291	Uncharacterized membrane protein	S	2	3	4
COG0027	Formate-dependent phosphoribosylglycinamide formyltransferase (GAR transformylase)	F	1681	1748	3428
COG5330	Uncharacterized conserved protein, DUF2336 family	S	0	12	12
COG1302	Uncharacterized conserved protein YloU, alkaline shock protein (Asp23) family	S	4594	6309	10903
COG3143	Phosphoaspartate phosphatase CheZ, dephosphorylates CheY~P	N;T	11	22	33

COG5285	Ectoine hydroxylase-related dioxygenase, phytanoyl-CoA dioxygenase (PhyH) family	Q	7	17	25
COG1735	Predicted metal-dependent hydrolase, phosphotriesterase family	R	92	390	482
COG0043	3-polyprenyl-4-hydroxybenzoate decarboxylase	H	472	746	1218
COG2961	23S rRNA A2030 N6-methylase RlmJ	J	6	12	18
COG1319	Aldehyde, CO, or xanthine dehydrogenase, FAD-binding subunit	C	582	2258	2840
COG0207	Thymidylate synthase	F	6767	7490	14257
COG2340	Spore germination protein YkwD and related proteins with CAP (CSP/antigen 5/PR1) domain	D;R	6946	7059	14004
COG1023	6-phosphogluconate dehydrogenase (decarboxylating)	G	1	4	5
COG3598	RecA-family ATPase	L	6931	7326	14257
COG4679	Phage-related protein	X	1185	1562	2748
COG4584	Transposase	X	16628	25694	42322
COG3107	Outer membrane lipoprotein LpoA, binds and activates PBP1a	M	21	285	306
COG4778	Alpha-D-ribose 1-methylphosphonate 5-triphosphate synthase subunit PhnL	P	103	32	135
COG4586	ABC-type uncharacterized transport system, ATPase component	R	5492	3952	9444
COG0227	Ribosomal protein L28	J	990	1063	2053
COG5108	Mitochondrial DNA-directed RNA polymerase	K	0	9	9
COG1186	Protein chain release factor PrfB	J	7973	11464	19437
COG5478	Low affinity Fe/Cu permease	P	0	0	1
COG1124	ABC-type dipeptide/oligopeptide/nickel transport system, ATPase component	E;P	249	718	966

COG4708	ECF-type transporter of queuosine precursor, membrane (S) component	F;J	2686	3107	5793
COG2257	Type III secretion system substrate exporter, FlhB-like	U	656	712	1368
COG3794	Plastocyanin	C	2	1	3
COG3104	Dipeptide/tripeptide permease	E	4025	5339	9364
COG1401	5-methylcytosine-specific restriction endonuclease McrBC, GTP-binding regulatory subunit McrB	V	5276	5830	11106
COG2320	GrpB domain, predicted nucleotidyltransferase, UPF0157 family	R	4715	4408	9122
COG0795	Lipopolysaccharide export LptBFGC system, permease protein LptF	M;N	6326	8118	14445
COG0588	Phosphoglycerate mutase (BPG-dependent)	G	1173	1614	2787
COG4449	Predicted protease, Abi (CAAX) family	R	4	0	4
COG1042	Acyl-CoA synthetase (NDP forming)	C	805	1272	2077
COG0309	Carbamoyl dehydratase HypE (hydrogenase maturation factor)	O	2211	3889	6100
COG0813	Purine-nucleoside phosphorylase	F	2106	3481	5587
COG1894	NADH:ubiquinone oxidoreductase, NADH-binding 51 kD subunit (chain F)	C	4536	7519	12055
COG0158	Fructose-1,6-bisphosphatase	G	286	380	666
COG0037	tRNA(Ile)-lysidine synthase TilS/MesJ	J	18898	24232	43130
COG0501	Zn-dependent protease with chaperone function	O	3322	3789	7111
COG1602	Archaeal DNA repair protein NreA	L	0	0	0
COG1086	NDP-sugar epimerase, includes UDP-GlcNAc-inverting 4,6-dehydratase FlaA1 and capsular polysaccharide biosynthesis protein EpsC	M;O	17809	20350	38158
COG3108	Uncharacterized conserved protein YcbK, DUF882 family	S	1134	1105	2239

COG3537	Putative alpha-1,2-mannosidase	G	6649	9546	16195
COG2829	Outer membrane phospholipase A	M	940	825	1766
COG1001	Adenine deaminase	F	1220	3099	4319
COG0726	Peptidoglycan/xylan/chitin deacetylase, PgdA/NodB/CDA1 family	G;M	32596	38804	71400
COG0864	Metal-responsive transcriptional regulator, contains CopG/Arc/MetJ DNA-binding domain	K	228	401	629
COG1368	Phosphoglycerol transferase MdoB/OpgB, AlkP superfamily	M	11994	17845	29840
COG1035	Coenzyme F420-reducing hydrogenase, beta subunit	C	8897	12315	21212
COG0335	Ribosomal protein L19	J	2824	3505	6330
COG1853	FMN reductase RutF, DIM6/NTAB family	C	12488	14455	26943
COG1345	Flagellar capping protein FliD	N	7380	6876	14256
COG0489	Fe-S cluster carrier ATPase, Mrp/ApbC/NBP35 family	D	21330	26295	47625
COG4603	ABC-type guanosine uptake system NupNOPQ, permease component NupP	F	4318	6702	11019
COG5877	Stand-alone amidase domain	D	1309	1516	2824
COG2186	DNA-binding transcriptional regulator, FadR family	K	1974	3966	5940
COG0010	Arginase/agmatinase family enzyme	E	3022	3522	6543
COG4766	Ethanolamine utilization protein EutQ, cupin superfamily (function unknown)	E	112	260	372
COG3677	Transposase InsA	X	9788	19427	29215
COG5619	Uncharacterized conserved protein, DUF2290 domain	S	33	23	56
COG0542	ATP-dependent Clp protease, ATP-binding subunit ClpA	O	34889	44604	79494
COG0393	Uncharacterized pentameric protein YbjQ, UPF0145 family	S	769	1066	1835

COG1482	Mannose-6-phosphate isomerase, class I	G	6892	8678	15570
COG3536	Uncharacterized conserved protein, DUF971 family	S	0	92	92
COG3803	Uncharacterized conserved protein, DUF924 family	S	1	50	51
COG2731	Beta-galactosidase, beta subunit	G	1541	2775	4316
COG5514	Uncharacterized conserved protein, DUF1794 domain	S	1	132	133
COG3579	Aminopeptidase C	E	3894	4727	8621
COG5184	Alpha-tubulin suppressor ATS1 and related RCC1 domain-containing proteins	D;Z	19144	24757	43900
COG4941	Predicted RNA polymerase sigma factor, contains C-terminal TPR domain	K	8	7	15
COG1018	Flavodoxin/ferredoxin--NADP reductase	C	7	188	195
COG0313	16S rRNA C1402 (ribose-2'-O) methylase RsmI	J	10666	12603	23269
COG5811	Sporulation sigma factor SigE (mother cell-specific, SpoIIGB)	D;K	4247	5522	9770
COG0761	4-Hydroxy-3-methylbut-2-enyl diphosphate reductase IspH	I	5336	6655	11990
COG2607	Predicted ATPase, AAA+ superfamily	R	5202	5597	10799
COG5702	Photosystem I reaction center subunit A2, PsaB	C	30	19	49
COG2865	Predicted transcriptional regulator, contains HTH domain	K	29002	35567	64569
COG5912	Spore cortex protein CoxA (YrbB), YhcN/YlaJ family	D	11	33	44
COG3655	DNA-binding transcriptional regulator, XRE family	K	5491	6857	12349
COG4992	Acetylornithine/succinyldiaminopimelate/putrescine aminotransferase	E	7567	9097	16664
COG1899	Deoxyhypusine synthase	O;J	54	59	112

COG1546	Nicotinamide mononucleotide (NMN) deamidase PncC	H	2167	2227	4394
COG0699	Replication fork clamp-binding protein CrfC (dynamin-like GTPase family)	L	387	908	1295
COG4807	Uncharacterized conserved protein YehS, DUF1456 family	S	0	2	2
COG3758	Various environmental stresses-induced protein Ves (function unknown)	S	231	422	653
COG1162	Ribosome biogenesis GTPase RsgA	J	9842	12045	21887
COG1624	c-di-AMP synthetase, contains DisA_N domain	T	8588	10454	19043
COG4540	Phage P2 baseplate assembly protein gpV	X	385	549	933
COG5353	Uncharacterized conserved protein YpmB, contains C-terminal PepSY domain	S	1	43	44
COG0120	Ribose 5-phosphate isomerase	G	316	564	880
COG1994	Zn-dependent protease (includes sporulation protein SpoIVFB)	O	5181	7211	12392
COG3246	Uncharacterized conserved protein, DUF849 family	S	445	1646	2091
COG4917	Ethanolamine utilization protein EutP, contains a P-loop NTPase domain	E	137	409	546
COG0564	Pseudouridine synthase RluA, 23S rRNA- or tRNA-specific	J	26988	33604	60592
COG3616	D-serine deaminase, pyridoxal phosphate-dependent	E	92	738	831
COG1690	RNA-splicing ligase RtcB, repairs tRNA damage	J	3247	4089	7336
COG2273	Beta-glucanase, GH16 family	G	6213	7225	13438
COG4792	Type III secretory pathway, EscU/YscU component	U	3	3	7
COG4409	Neuraminidase (sialidase) NanH, contains C-terminal autotransporter domain	G;M	8871	12119	20990

COG4634	Predicted nuclease, contains PIN domain, potential toxin-antitoxin system component	R	0	0	0
COG0016	Phenylalanyl-tRNA synthetase alpha subunit	J	8536	11041	19577
COG3061	Cell division protein YtfB/OapA (opacity-associated protein A)	D	0	2	2
COG3185	4-hydroxyphenylpyruvate dioxygenase and related hemolysins	E;R	852	1280	2133
COG5856	Small acid-soluble spore protein SspI	D	17	24	41
COG3576	Predicted flavin-nucleotide-binding protein, pyridoxine 5'-phosphate oxidase superfamily	R	10	19	29
COG1402	Creatinine amidohydrolase/Fe(II)-dependent FAPy formamide hydrolase (riboflavin and F420 biosynthesis)	H	1277	2043	3320
COG4934	Serine protease, subtilase family	O	289	478	767
COG2096	Cob(II)alamin adenosyltransferase	H	2438	2779	5217
COG1862	Protein translocase subunit YajC	U	3187	3530	6717
COG0698	Ribose 5-phosphate isomerase RpiB	G	5400	7051	12451
COG5639	Uncharacterized conserved protein, DUF2274 domain	S	3	1	4
COG3663	G:T/U-mismatch repair DNA glycosylase	L	3908	4823	8731
COG1232	Protoporphyrinogen oxidase HemY/PPOX	H	4391	5306	9697
COG0631	Serine/threonine protein phosphatase PrpC	T	19241	21308	40549
COG2995	Intermembrane transporter PqiABC subunit PqiA	M	0	6	6
COG3567	Uncharacterized conserved protein, DUF1073 domain	S	648	1248	1896
COG2866	Murein tripeptide amidase MpaA	M	2659	3825	6484
COG0762	Cytochrome b6 maturation protein CCB3/Ycf19 and related maturases, YggT family	O	88	274	362
COG0525	Valyl-tRNA synthetase	J	13787	19375	33162

COG4191	Signal transduction histidine kinase regulating C4-dicarboxylate transport	T	868	3728	4595
COG3287	FIST domain protein MJ1623, contains FIST_N and FIST_C domains	T	798	888	1686
COG2853	Lipoprotein subunit MlaA of the ABC-type intermembrane phospholipid transporter Mla	M	47	497	544
COG4928	Predicted P-loop ATPase, KAP-like	R	2685	3702	6387
COG1464	ABC-type metal ion transport system, periplasmic component/surface antigen	P	2411	3443	5854
COG0278	Glutaredoxin-related protein	O	3	1	4
COG5462	Predicted secreted (periplasmic) protein	S	11	47	59
COG3458	Cephalosporin-C deacetylase or related acetyl esterase	Q	1722	2038	3760
COG1284	Uncharacterized membrane-anchored protein YitT, contains DUF161 and DUF2179 domains	S	16012	19925	35937
COG2876	3-deoxy-D-arabino-heptulosonate 7-phosphate (DAHP) synthase	E	2939	4560	7499
COG1385	16S rRNA U1498 N3-methylase RsmE	J	7460	8542	16002
COG2512	Predicted transcriptional regulator, contains CW (cell wall-binding) repeats and an HTH domain	R	667	906	1574
COG1360	Flagellar motor protein MotB	N	3695	3570	7265
COG4224	Uncharacterized conserved protein YnzC, UPF0291/DUF896 family	S	475	660	1135
COG3216	Uncharacterized conserved protein, DUF2062 family	S	3	69	72
COG4160	ABC-type arginine/histidine transport system, permease component	E	0	3	3
COG4637	Predicted ATPase	R	3715	5832	9548
COG4477	Septation ring formation regulator EzrA	D	287	650	937
COG5518	Phage capsid portal protein XkdE	X	417	438	855

COG0108	3,4-dihydroxy-2-butanone 4-phosphate synthase	H	3219	4437	7656
COG1317	Flagellar biosynthesis/type III secretory pathway protein FliH	N;U	3006	3874	6880
COG0786	Na+/glutamate symporter	E	498	1109	1607
COG0657	Acetyl esterase/lipase	I	19598	25885	45484
COG0771	UDP-N-acetylmuramoylalanine-D-glutamate ligase	M	12063	15388	27452
COG4969	Type IV pilus assembly protein, major pilin PilA	N;W	75	110	185
COG0371	Glycerol dehydrogenase or related enzyme, iron-containing ADH family	C	1527	2562	4089
COG0596	2-succinyl-6-hydroxy-2,4-cyclohexadiene-1-carboxylate synthase MenH and related esterases, alpha/beta hydrolase fold	H;R	15332	19280	34612
COG3640	CO dehydrogenase nickel-insertion accessory protein CooC1	O	1906	3740	5645
COG0692	Uracil-DNA glycosylase	L	5131	7433	12564
COG1432	NYN domain, predicted PIN-related RNAse, tRNA/rRNA maturation	R	4140	3957	8097
COG3701	Type IV secretory pathway, TrbF component	U	1981	3531	5513
COG1340	Uncharacterized coiled-coil protein, contains DUF342 domain	S	816	1232	2048
COG2244	Membrane protein involved in the export of O-antigen and teichoic acid	M	34601	44805	79406
COG3607	Lactoylglutathione lyase-related enzyme, vicinal oxygen chelate (VOC) family	R	3	11	14
COG3724	Succinylarginine dihydrolase	E	1	5	6
COG2156	K+-transporting ATPase, KdpC subunit	P	76	120	196
COG2233	Xanthine/uracil permease	F	4710	8029	12739
COG2059	Chromate transport protein ChrA	P	9349	12023	21372

COG1941	Coenzyme F420-reducing hydrogenase, gamma subunit	C	66	74	140
COG2374	Predicted extracellular nuclease	R	5927	5580	11507
COG0251	Enamine deaminase RidA, house cleaning of reactive enamine intermediates, YjgF/YER057c/UK114 family	V	3883	5675	9558
COG2521	Predicted archaeal methyltransferase	R	21	18	40
COG4655	Uncharacterized conserved protein, contains TadX/Tad_C domain	R	24	23	47
COG2710	Nitrogenase Mo-Fe protein NifD/coenzyme F430 biosynthesis subunit CfbD	H;P	614	1113	1727
COG4518	Mu-like prophage FluMu protein gp41	X	0	1	1
COG1133	Peptide antibiotic transporter SbmA/BacA, ABC-type permease family	V	0	3	3
COG1530	Ribonuclease G or E	J	5714	8699	14413
COG0455	MinD-like ATPase FlhG/YlxH, activator of the FlhF-type GTPase	D;N	2773	2344	5117
COG2409	Predicted lipid transporter YdfJ, MMPL/SSD domain, RND superfamily	R	0	3	3
COG0634	Hypoxanthine-guanine phosphoribosyltransferase	F	6123	6945	13068
COG1562	Phytoene/squalene synthetase	I	475	1346	1821
COG1433	Predicted Fe-Mo cluster-binding protein, NifX family	O	3209	4236	7445
COG3203	Outer membrane porin OmpC/OmpF/PhoE	M	7	2479	2486
COG3795	5-chloro-2-hydroxyhydroquinone dehydrochlorinase TtfG, YCII superfamily	Q	1	3	4
COG0839	NADH:ubiquinone oxidoreductase subunit 6 (chain J)	C	916	1222	2138
COG5835	Stage V sporulation protein SpoVAB, subunit of dipicolinate uptake complex	D;E	1032	1244	2276
COG1932	Phosphoserine aminotransferase	H;E	6761	7343	14104

COG5498	Endoglucanase Acf2	G	214	199	413
COG4675	Phage tail collar domain	X	1339	2058	3398
COG0347	Nitrogen regulatory protein PII	T;E	2202	2996	5198
COG0427	Propionyl CoA:succinate CoA transferase	C	4802	7977	12779
COG1141	Ferredoxin	C	214	338	552
COG1990	Peptidyl-tRNA hydrolase	J	118	167	285
COG2086	Electron transfer flavoprotein, alpha and beta subunits	C	3423	5406	8829
COG3696	Cu/Ag efflux pump CusA	P	2480	4177	6657
COG0702	Uncharacterized conserved protein YbjT, contains NAD(P)-binding and DUF2867 domains	R	1447	1212	2659
COG1570	Exonuclease VII, large subunit	L	10507	12505	23012
COG0144	16S rRNA C967 or C1407 C5-methylase, RsmB/RsmF family	J	11226	16279	27505
COG3317	Outer membrane beta-barrel protein assembly factor BamC	M	0	184	185
COG2899	Uncharacterized TerC-related membrane protein, DUF475 domain	P	10	131	140
COG1714	Uncharacterized membrane protein YckC, RDD family	S	420	565	985
COG2802	Uncharacterized conserved protein, LON_N-like domain, ASCH/PUA-like superfamily	S	2	4	6
COG1794	Amino acid racemase YgeA	M	1507	2450	3957
COG2431	Lysine export protein LysO/YbjE, DUF340 family	E	215	705	920
COG1217	Predicted membrane GTPase TypA/BipA involved in stress response	T	8772	12325	21097
COG5316	Uncharacterized protein, contains DUF4139 domain	S	96	258	353
COG4272	Uncharacterized membrane protein	S	0	47	47

COG1944	Ribosomal protein S12 methylthiotransferase accessory factor YcaO	J	779	533	1312
COG2008	Threonine aldolase	E	2405	4285	6690
COG3610	Uncharacterized membrane protein YjjB, DUF3815 family	S	707	1799	2506
COG3500	Phage protein D	X	3376	2918	6293
COG5567	Small periplasmic lipoprotein YifL (function unknown)	S	253	352	604
COG4098	Superfamily II DNA/RNA helicase required for DNA uptake (late competence protein)	L	224	250	474
COG4918	Predicted Fe-S cluster biosynthesis protein YqkB	R	0	29	29
COG1586	S-adenosylmethionine decarboxylase	E	800	697	1497
COG3052	Acyl-carrier protein (citrate lyase gamma subunit)	C	196	285	481
COG4570	Holliday junction resolvase RusA (prophage-encoded endonuclease)	L	1314	1561	2875
COG0511	Biotin carboxyl carrier protein	I	4043	5005	9048
COG4100	Cystathionine beta-lyase family protein involved in aluminum resistance	P;R	5997	8324	14321
COG4296	Uncharacterized conserved protein, DUF2262 domain	S	219	281	500
COG4800	Predicted transcriptional regulator with an HTH domain	K	0	0	0
COG3220	Uncharacterized conserved protein, UPF0276 family	S	1	11	12
COG3268	Uncharacterized conserved protein, related to short-chain dehydrogenases	S	1	1	2
COG1640	4-alpha-glucanotransferase	G	12324	14938	27261
COG0805	Twin-arginine protein secretion pathway component TatC	U	2847	3867	6713
COG1605	Chorismate mutase	E	2924	3202	6126

COG0367	Asparagine synthetase B (glutamine-hydrolyzing)	E	12607	13960	26567
COG0554	Glycerol kinase	C	5279	8045	13324
COG3221	ABC-type phosphate/phosphonate transport system, periplasmic component	P	173	299	472
COG0078	Ornithine carbamoyltransferase	E	6375	9387	15762
COG4636	Endonuclease, Uma2 family (restriction endonuclease fold)	R	4420	4850	9270
COG4222	Uncharacterized conserved protein, contains a phytase-like domain	S	0	2	2
COG1543	Predicted glycosyl hydrolase, contains GH57 and DUF1957 domains	G	315	800	1115
COG3237	Uncharacterized conserved protein YjbJ, UPF0337 family	S	0	10	10
COG1316	Anionic cell wall polymer biosynthesis enzyme TagV/TagU, LytR-Cps2A-Psr (LCP) family (peptidoglycan teichoic acid transferase)	M	14275	19382	33657
COG0248	Exopolyphosphatase/pppGpp-phosphohydrolase	F;T	4167	7713	11880
COG0535	Radical SAM superfamily maturase, SkfB/NifB/PqqE family	D;H	22628	26989	49617
COG0349	Ribonuclease D	J	2277	3063	5340
COG2704	Anaerobic C4-dicarboxylate transporter	G	79	936	1015
COG2346	Truncated hemoglobin YjbI	P	11	0	11
COG2019	Archaeal adenylate kinase	F	76	122	199
COG3123	Pyrimidine/purine nucleoside phosphorylase YaiE/PpnP, UPF0345/DUF1255 family	F	0	1	1
COG4964	Flp pilus assembly protein, secretin CpaC	U;W	14	54	68
COG2251	Predicted nuclease, RecB family	R	94	280	375

COG0508	Pyruvate/2-oxoglutarate dehydrogenase complex, dihydrolipoamide acyltransferase (E2) component	C	2038	4778	6816
COG4912	3-methyladenine DNA glycosylase AlkD	L	5769	7381	13149
COG2376	Dihydroxyacetone kinase	G	534	1482	2016
COG4587	ABC-type uncharacterized transport system, permease component	R	3613	3144	6757
COG1243	tRNA U34 5-carboxymethylaminomethylation enzyme Elp3 (RNA elongator complex protein 3), contains radical SAM and acetyltransferase	J	2580	2499	5079
COG0064	Asp-tRNAAsn/Glu-tRNAGln amidotransferase B subunit	J	5599	8021	13620
COG3855	Fructose-1,6-bisphosphatase	G	4826	6369	11195
COG0472	UDP-N-acetylmuramyl pentapeptide phosphotransferase/UDP-N-acetylglucosamine-1-phosphate transferase	M	14519	18792	33311
COG3839	ABC-type sugar transport system, ATPase component MalK	G	14103	16145	30248
COG4281	Acyl-CoA-binding protein	I	1	4	5
COG1993	PII-like signaling protein	T	0	2	2
COG1635	Thiazole synthase/Archaeal ribulose 1,5-bisphosphate synthetase	G;H	309	259	568
COG0443	Molecular chaperone DnaK (HSP70)	O	18133	21434	39568
COG0118	Imidazoleglycerol phosphate synthase glutamine amidotransferase subunit HisH	E	3490	4814	8304
COG5805	Sporulation sensor histidine kinase A (Stage II sporulation protein SpoIIF/SpoIIJ)	D;T	1	15	16
COG4772	Outer membrane receptor for Fe3+-dicitrate	P	3609	3279	6888
COG4552	Predicted acetyltransferase	R	2598	3177	5775
COG5337	Spore coat protein CotH	M	9556	8489	18045
COG0611	Thiamine monophosphate kinase	H	3713	4338	8050

COG3867	Arabinogalactan endo-1,4-beta-galactosidase	G	4833	5421	10254
COG2973	Trp operon repressor	K	0	2	2
COG1164	Oligoendopeptidase F	E	9218	12712	21930
COG3714	Uncharacterized membrane protein YhhN	S	319	159	478
COG5655	Replication initiaton protein REP (rolling circle plasmid replication)	X	1210	14746	15956
COG3190	Flagellar biogenesis protein FliO	N	1266	1172	2438
COG3976	Uncharacterized conserved protein, contains FMN-binding domain	R	2398	3113	5511
COG4149	ABC-type molybdate transport system, permease component ModB	P	676	1332	2008
COG1435	Thymidine kinase	F	3392	3706	7098
COG0372	Citrate synthase	C	7758	8752	16509
COG0395	ABC-type glycerol-3-phosphate transport system, permease component	G	36186	48129	84315
COG1859	RNA:NAD 2'-phosphotransferase, TPT1/KptA family	J	938	1041	1979
COG5005	Mu-like prophage protein gpG	X	793	726	1519
COG2834	Outer membrane lipoprotein-sorting protein	M	1385	2177	3562
COG5441	ATP-binding helicase-inhibiting domain, Tm-1/UPF0261 family	V	51	234	285
COG3741	N-formylglutamate amidohydrolase	E	44	256	300
COG2039	Pyrrolidone-carboxylate peptidase (N-terminal pyroglutamyl peptidase)	O	346	616	963
COG5004	P2-like prophage tail protein X	X	140	178	319
COG5029	Prenyltransferase, beta subunit	O;I	143	750	892
COG1995	4-hydroxy-L-threonine phosphate dehydrogenase PdxA	H	3308	3976	7285
COG2502	Asparagine synthetase A	E	3628	5039	8667
COG2324	Uncharacterized membrane protein	S	15	49	64

COG0700	Spore maturation protein SpmB (function unknown)	S	4052	5289	9341
COG4653	Predicted phage phi-C31 gp36 major capsid-like protein	X	4847	6783	11630
COG2770	HAMP domain	T	730	1638	2368
COG1231	Monoamine oxidase	E	7	12	20
COG4397	Mu-like prophage major head subunit gpT	X	124	327	450
COG1004	UDP-glucose 6-dehydrogenase	M	8497	10097	18594
COG0218	GTP-binding protein EngB required for normal cell division	D	6156	7540	13696
COG0743	1-deoxy-D-xylulose 5-phosphate reductoisomerase	I	8863	10842	19705
COG0494	8-oxo-dGTP pyrophosphatase MutT and related house-cleaning NTP pyrophosphohydrolases, NUDIX family	V	6295	8535	14830
COG3488	Uncharacterized conserved protein with two CxxC motifs, DUF1111 family	R	652	474	1126
COG4455	Protein of avirulence locus involved in temperature-dependent protein secretion	R	2	4	6
COG4688	Uncharacterized conserved protein	S	0	1	1
COG5918	Uncharacterized sporulation protein YmfJ, contains DUF3243 domain	D	4	14	18
COG3188	Outer membrane usher protein FimD/PapC	N;W	107	283	390
COG3765	LPS O-antigen chain length determinant protein, WzzB/FepE family	M	853	953	1805
COG0610	Type I site-specific restriction-modification system, R (restriction) subunit and related helicases ...	V	17418	22913	40331
COG0090	Ribosomal protein L2	J	6542	8469	15012
COG1064	D-arabinose 1-dehydrogenase, Zn-dependent alcohol dehydrogenase family	G	255	475	730
COG5412	Phage-related protein	X	10403	13896	24299

COG3339	Uncharacterized membrane protein YkvA, DUF1232 family	S	1082	1160	2242
COG1930	ABC-type cobalt transport system, periplasmic component	P	20	62	83
COG0722	3-deoxy-D-arabino-heptulosonate 7-phosphate (DAHP) synthase	E	3740	5054	8794
COG1564	Thiamine pyrophosphokinase	H	4367	5031	9397
COG0744	Penicillin-binding protein 1B/1F, peptidoglycan transglycosylase/transpeptidase	M	16256	22962	39218
COG2082	Precorrin-8X methylmutase CbiC/CobH	H	1862	1935	3798
COG2941	Demethoxyubiquinone hydroxylase, CLK1/Coq7/Cat5 family (ubiquinone biosynthesis)	H	1	1	2
COG2359	Stage V sporulation protein SpoVS, predicted DNA-binding, AlbA superfamily	S	335	274	609
COG0247	Fe-S cluster-containing oxidoreductase, includes glycolate oxidase subunit GlcF	C	3082	4734	7815
COG1327	Transcriptional regulator NrdR, contains Zn-ribbon and ATP-cone domains	K	2464	3565	6029
COG0312	Zn-dependent protease PmbA/TldA or its inactivated homolog	R	4694	6732	11426
COG2185	Methylmalonyl-CoA mutase, C-terminal domain/subunit (cobalamin-binding)	I	324	477	802
COG0456	Ribosomal protein S18 acetylase RimI and related acetyltransferases	J	39949	44876	84825
COG1615	Uncharacterized membrane protein, UPF0182 family	S	645	1432	2077
COG4458	Virulence factor SrfC-related protein	Q	519	675	1194
COG0070	Glutamate synthase domain 3	E	1705	1658	3363
COG3978	Acetolactate synthase small subunit, contains ACT domain	E	0	1	1

COG4091	Predicted homoserine dehydrogenase, contains C-terminal SAF domain	E	245	264	509
COG2168	Sulfur transfer complex TusBCD TusB component, DsrH family	J	0	2	2
COG4627	Predicted SAM-depedendent methyltransferase	R	381	275	657
COG4495	Uncharacterized conserved protein, DUF4176 domain	S	282	641	923
COG1119	ABC-type molybdenum transport system, ATPase component ModF/photorepair protein PhrA	P	449	1208	1656
COG2160	L-arabinose isomerase	G	2465	3842	6307
COG1985	Pyrimidine reductase, riboflavin biosynthesis	H	1953	2680	4633
COG4170	ABC-type antimicrobial peptide export system, ATPase component SapD	V	4	4	8
COG3100	Uncharacterized conserved protein YcgL, UPF0745 family	S	0	1	1
COG1563	Uncharacterized MnhB-related membrane protein	R	495	473	968
COG4379	Mu-like prophage tail protein gpP	X	319	584	903
COG1378	Sugar-specific transcriptional regulator	K	659	470	1128
COG0407	Uroporphyrinogen-III decarboxylase HemE	H	2329	4562	6891
COG4385	Bacteriophage P2-related tail formation protein	X	2517	2444	4961
COG0589	Nucleotide-binding universal stress protein, UspA family	T	1095	2470	3565
COG3513	CRISPR-Cas system type-II protein Cas9	V	2965	4788	7753
COG1423	ATP-dependent RNA circularization protein, DNA/RNA ligase (PAB1020)	L	684	996	1680
COG0094	Ribosomal protein L5	J	3681	5206	8888
COG3472	Uncharacterized conserved protein domain, often C-terminal to DUF262	S	373	419	792

COG5821	Stage II sporulation protein SpoIIQ, clostridial version, metallopeptidase M23 family	D	960	1772	2731
COG2989	Murein L,D-transpeptidase YcbB/YkuD	M	0	10	10
COG1238	Uncharacterized membrane protein YqaA, VTT domain	S	781	1113	1894
COG3014	Uncharacterized conserved protein	S	118	111	229
COG1452	LPS assembly outer membrane protein LptD (organic solvent tolerance protein OstA)	M	5233	6789	12022
COG3649	CRISPR-Cas system type I effector complex subunit Cas7, RAMP superfamily	V	2442	2497	4939
COG5479	LGFP repeat-containing protein, may be involved in cell wall binding	M	13	9	22
COG1903	Cobalamin biosynthesis protein CbiD (cobalt-precorrin-5B C-methyltransferase)	H	2823	4186	7008
COG3303	Formate-dependent nitrite reductase, periplasmic cytochrome c552 subunit	P	347	1146	1493
COG2352	Phosphoenolpyruvate carboxylase	C	9	24	33
COG3708	Predicted transcriptional regulator YdeE, contains AraC-type DNA-binding domain	K	631	697	1329
COG5012	Methanogenic corrinoid protein MtbC1	C	795	2109	2904
COG3551	Uncharacterized conserved protein	S	14	11	25
COG4994	Uncharacterized protein CV0675, UCP033623 family	S	45	29	74
COG0618	nanoRNase/pAp phosphatase, hydrolyzes c-di-AMP and oligoRNAs	F;T	15033	17057	32090
COG3257	Ureidoglycine aminohydrolase	F	39	148	187
COG5429	Uncharacterized conserved protein, DUF1223 domain	S	1	4	5
COG0139	Phosphoribosyl-AMP cyclohydrolase	E	1468	2249	3716
COG0574	Phosphoenolpyruvate synthase/pyruvate phosphate dikinase	G	15067	19663	34731

COG2966	Uncharacterized membrane protein YjjP, DUF1212 family	S	1997	3890	5887
COG3403	Uncharacterized conserved protein YcgG, contains conserved FPC and CPF motifs	S	2	0	2
COG0060	Isoleucyl-tRNA synthetase	J	15816	22470	38286
COG5654	Uncharacterized protein, contains RES domain	S	343	280	624
COG0757	3-dehydroquinate dehydratase	E	3894	3999	7893
COG2843	Poly-gamma-glutamate biosynthesis protein CapA/YwtB (capsule formation), metallophosphatase superfamily	M	14583	14853	29436
COG0658	DNA uptake channel protein ComEC, N-terminal domain	U	10636	13127	23763
COG1622	Heme/copper-type cytochrome/quinol oxidase, subunit 2	C	185	141	327
COG1749	Flagellar hook protein FlgE	N	3740	3510	7251
COG1783	Phage terminase large subunit	X	10621	13594	24214
COG0073	tRNA-binding EMAP/Myf domain	J	16141	20247	36388
COG1451	UTP pyrophosphatase, metal-dependent hydrolase family	R	5840	7099	12939
COG0448	Glucose-1-phosphate adenylyltransferase (ADP-glucose pyrophosphorylase)	G	12535	16668	29203
COG0736	Phosphopantetheinyl transferase (holo-ACP synthase)	I	937	1662	2599
COG3252	Methenyltetrahydromethanopterin cyclohydrolase	H	0	0	0
COG0552	Signal recognition particle GTPase FtsY	U	8761	10567	19329
COG3077	Antitoxin component of the RelBE or YafQ-DinJ toxin-antitoxin module	V	4531	5141	9672
COG1138	Cytochrome c biogenesis protein CcmF	C;O	145	672	817
COG0345	Pyrroline-5-carboxylate reductase	E	4791	6688	11479
COG3177	Fic family protein	K	22215	25317	47532

COG1536	Flagellar motor switch protein FliG	N	1904	2161	4066
COG1819	UDP:flavonoid glycosyltransferase YjiC, YdhE family	G	1227	970	2196
COG4639	Predicted kinase	R	1866	2919	4784
COG0420	DNA repair exonuclease SbcCD nuclease subunit	L	8991	12285	21277
COG3790	Predicted membrane protein, encoded in cydAB operon	S	0	55	55
COG0339	Zn-dependent oligopeptidase, M3 family	O	6857	7917	14775
COG0063	NAD(P)H-hydrate repair enzyme Nnr, NAD(P)H-hydrate dehydratase domain	F	1066	1442	2507
COG4227	Antirestriction protein ArdC	L	14680	21947	36627
COG4187	Arginine utilization protein RocB	E	51	160	211
COG4832	Cyclopropanoid cyclopropyl hydrolase, GyrI-like domain	V;T	3525	4389	7914
COG2987	Urocanate hydratase	E	1597	2637	4234
COG3491	Isopenicillin N synthase and related dioxygenases	Q	4	11	15
COG0604	NADPH:quinone reductase or related Zn-dependent oxidoreductase	C;R	327	1277	1604
COG1446	Isoaspartyl peptidase or L-asparaginase, Ntn-hydrolase superfamily	E	43	312	355
COG4650	Sigma54-dependent transcription regulator containing an AAA-type ATPase domain and a DNA-binding domain	K;T	1	11	12
COG0595	mRNA degradation ribonuclease J1/J2	J	10287	12674	22961
COG0185	Ribosomal protein S19	J	957	1286	2243
COG4758	Membrane protein LiaF, inhibitor of the LiaRS two-component envelope stress sensory system	T	146	258	404
COG3980	Spore coat polysaccharide biosynthesis protein SpsG, predicted glycosyltransferase	M	1728	2306	4034

COG0685	5,10-methylenetetrahydrofolate reductase	E	6644	6923	13567
COG4722	Phage-related protein YomH	X	941	964	1905
COG3370	Uncharacterized conserved protein AF1964, DsrE/DsrF-like family	R	0	0	0
COG1489	DNA-binding protein, stimulates sugar fermentation	G;T	2100	2484	4584
COG0245	2C-methyl-D-erythritol 2,4-cyclodiphosphate synthase	I	5432	6510	11942
COG0009	tRNA A37 threonylcarbamoyladenosine synthetase subunit TsaC/SUA5/YrdC	J	9948	12687	22635
COG1414	DNA-binding transcriptional regulator, IclR family	K	834	2468	3303
COG0577	ABC-type antimicrobial peptide transport system, permease component	V	31136	37732	68868
COG4221	NADP-dependent 3-hydroxy acid dehydrogenase YdfG	C	2480	2596	5076
COG5427	Predicted membrane glycosyltransferase AF0583, Arch_YYY/DUF2298 family	S	316	431	746
COG3568	Metal-dependent hydrolase, endonuclease/exonuclease/phosphatase family	R	6321	9495	15816
COG2304	Secreted protein containing bacterial Ig-like domain and vWFA domain	R	3986	3518	7504
COG2367	Beta-lactamase class A	V	2159	3520	5679
COG3563	Capsule polysaccharide export protein KpsC/LpsZ	M	183	136	319
COG4108	Peptide chain release factor RF-3	J	6184	7600	13784
COG1567	CRISPR-Cas system type III CSM-effector complex subunit Csm4, RAMP superfamily Cas5 group	V	117	393	510
COG0232	dGTP triphosphohydrolase	F	9819	12460	22279

COG0303	Molybdopterin Mo-transferase (molybdopterin biosynthesis)	H	1941	4077	6018
COG1025	Secreted/periplasmic Zn-dependent peptidases, insulinase-like	O	0	8	8
COG0468	RecA/RadA recombinase	L	9523	14774	24297
COG2410	Predicted nuclease (RNAse H fold)	R	1	3	4
COG3645	Phage antirepressor protein YoqD, KilAC domain	X	2389	3386	5775
COG4853	YycI protein, regulator of the WalKR two-component signal transduction system	T	130	363	494
COG1101	ABC-type uncharacterized transport system, ATPase component	R	4225	5217	9442
COG4687	Uncharacterized conserved protein	S	21	99	120
COG3137	Putative salt-induced outer membrane protein YdiY	M	1	7	8
COG4279	Uncharacterized protein, contains SWIM-type Zn finger domain	S	938	1330	2269
COG0305	Replicative DNA helicase	L	16202	21462	37664
COG0405	Gamma-glutamyltranspeptidase	E	250	700	950
COG1475	Chromosome segregation protein Spo0J, contains ParB-like nuclease domain	D	37088	42142	79230
COG5703	Photosystem I reaction center iron-sulfur center subunit VII, PsaC	C	1	3	4
COG1353	CRISPR/Cas system-associated protein Cas10, large subunit of type III CRISPR-Cas systems, contains HD superfamily nuclease domain	V	558	1293	1851
COG0440	Acetolactate synthase, small subunit	E	3453	4727	8180
COG4786	Flagellar basal body rod protein FlgG	N	6423	5611	12034
COG1347	Na+-transporting NADH:ubiquinone oxidoreductase, subunit NqrD	C	1490	1482	2972

COG1331	Uncharacterized conserved protein YyaL, SSP411 family, contains thoiredoxin and six-hairpin glycosidase-like domains	R	3765	4696	8461
COG3878	Uncharacterized conserved protein YwqG, DUF1963 family	S	2925	1505	4430
COG2197	DNA-binding response regulator, NarL/FixJ family, contains REC and HTH domains	T;K	9946	13639	23585
COG4678	Muramidase (phage lambda lysozyme)	M;X	5	14	19
COG2406	Ferritin-like DNA-binding protein, DPS (DNA Protection under Starvation) family	L	24	137	161
COG2884	Cell division ATPase FtsE	D	6241	7689	13930
COG2208	Phosphoserine phosphatase RsbU, regulator of sigma subunit	T;K	4563	4742	9305
COG1945	Pyruvoyl-dependent arginine decarboxylase	E	95	111	206
COG4076	Predicted RNA methylase	R	1	15	16
COG3344	Retron-type reverse transcriptase	X	47829	56281	104110
COG0635	Coproporphyrinogen-III oxidase HemN (oxygen-independent) or related Fe-S oxidoreductase	H	17544	22543	40087
COG4833	Predicted alpha-1,6-mannanase, GH76 family	G	396	709	1105
COG1884	Methylmalonyl-CoA mutase, N-terminal domain/subunit	I	5637	6440	12077
COG4915	5-bromo-4-chloroindolyl phosphatase XpaC	Q;R	2364	3427	5791
COG5578	Uncharacterized membrane protein YesL	S	6627	7061	13688
COG4208	ABC-type sulfate transport system, permease component	P	239	126	365
COG1544	Ribosome-associated translation inhibitor RaiA	J	4265	5925	10190
COG3519	Type VI protein secretion system component VasA	U	16	14	30
COG5281	Phage-related minor tail protein	X	960	814	1775

COG1523	Pullulanase/glycogen debranching enzyme	G	9545	10491	20036
COG1300	Stage II sporulation protein SpoIIM, component of the engulfment complex	D	439	993	1431
COG1694	NTP pyrophosphatase, house-cleaning of non-canonical NTPs	V	1932	2159	4091
COG3761	NADH:ubiquinone oxidoreductase NDUFA12 subunit (Leigh syndrome)	C	1	2	3
COG4354	Uncharacterized conserved protein, contains GBA2_N and DUF608 domains	S	1198	1694	2892
COG1982	Arginine/lysine/ornithine decarboxylase	E	8842	10424	19266
COG1506	Dipeptidyl aminopeptidase/acylaminoacyl peptidase	E	17018	19749	36768
COG2000	Uncharacterized Fe-S cluster-containing protein	R	4	6	10
COG3737	Uncharacterized protein, contains Mth938-like domain	S	1	1	2
COG1266	Membrane protease YdiL, CAAX protease family	O	21002	23079	44081
COG2270	MFS-type transporter involved in bile tolerance, Atg22 family	R	2080	3160	5240
COG0656	Aldo/keto reductase, related to diketogulonate reductase	Q	3356	4352	7709
COG5843	Dipicolinate synthase subunit B (sporulation protein SpoVFB)	D;E	2654	2766	5420
COG1228	Imidazolonepropionase or related amidohydrolase	Q	6754	9120	15874
COG4485	Uncharacterized membrane protein YfhO	S	15714	16960	32674
COG3412	Phosphotransferase subunit DhaM of the dihydroxyacetone kinase DhaKLM complex, contains PTS-EIIA, HPr, and PEP-utilizing domains	T	29	107	137

COG4604	ABC-type enterochelin transport system, ATPase component	P	98	176	274
COG3926	Lysozyme family protein	R	520	1536	2056
COG1242	Radical SAM superfamily enzyme	R	4802	5013	9815
COG0594	RNase P protein component	J	4891	5429	10320
COG5454	Predicted secreted protein	S	1	1	2
COG0333	Ribosomal protein L32	J	612	776	1388
COG3238	Uncharacterized membrane protein YdcZ, DUF606 family	S	802	1220	2022
COG4909	Propanediol dehydratase, large subunit	Q	104	172	276
COG2132	Multicopper oxidase with three cupredoxin domains (includes cell division protein FtsP and spore coat protein CotA)	D;P;M	16	186	202
COG4806	L-rhamnose isomerase	G	4173	4695	8868
COG4765	Uncharacterized conserved protein, DUF2155 domain	S	20	88	108
COG0825	Acetyl-CoA carboxylase alpha subunit	I	1974	2731	4705
COG4736	Cbb3-type cytochrome oxidase, subunit 3	C	2	0	2
COG1841	Ribosomal protein L30/L7E	J	577	736	1313
COG3428	Uncharacterized membrane protein YdbT, contains bPH2 (bacterial pleckstrin homology) domain	S	1259	1183	2442
COG4970	Type IV pilus assembly protein FimT	N;W	600	773	1373
COG0036	Pentose-5-phosphate-3-epimerase	G	8318	10560	18879
COG0742	16S rRNA G966 N2-methylase RsmD	J	6126	7361	13487
COG5400	Uncharacterized conserved protein	S	2	2	4
COG0424	7-methyl-GTP pyrophosphatase and related NTP pyrophosphatases, Maf/HAM1 superfamily	Q	6335	8049	14384
COG4541	Uncharacterized membrane protein	S	249	436	685
COG3896	Chloramphenicol 3-O-phosphotransferase	V	394	933	1327
COG3044	Predicted ATPase of the ABC class	R	2845	3113	5958

COG4903	Competence transcription factor ComK	K	124	236	360
COG2224	Isocitrate lyase	C	5	8	13
COG1005	NADH:ubiquinone oxidoreductase subunit 1 (chain H)	C	1480	1983	3463
COG2243	Precorrin-2 methylase	H	1383	2024	3407
COG4088	tRNA uridine 5-carbamoylmethylation protein Kti12 (Killer toxin insensitivity protein)	J;V	47	59	106
COG1131	ABC-type multidrug transport system, ATPase component	V	75529	80190	155719
COG5878	Uncharacterized sporulation protein YyaC,contains DUF1256 domain	D	688	1198	1885
COG3911	Predicted ATPase	R	4	42	46
COG1013	Pyruvate:ferredoxin oxidoreductase or related 2-oxoacid:ferredoxin oxidoreductase, beta subunit	C	7156	8809	15964
COG0834	ABC-type amino acid transport/signal transduction system, periplasmic component/domain	E;T	23360	24616	47975
COG1089	GDP-D-mannose dehydratase	M	4940	5176	10115
COG5437	Phage tail protein, TP901-1 family	X	118	37	156
COG2302	RNA-binding protein YlmH, contains S4-like domain	R	5035	5240	10275
COG3096	Chromosome condensin MukBEF, ATPase and DNA-binding subunit MukB	D	0	9	9
COG1021	EntE, 2,3-dihydroxybenzoate-AMP synthase component of non-ribosomal peptide synthetase	Q	48	27	75
COG1052	Lactate dehydrogenase or related 2-hydroxyacid dehydrogenase	C;H;R	11651	14544	26195
COG3323	PII-like insert in the uncharacterized protein YqfO, YbgI/NIF3 family	S	112	152	264

COG3611	Replication initiation and membrane attachment protein DnaB	L	528	809	1337
COG1987	Flagellar biosynthesis protein FliQ	N	808	679	1487
COG3637	Opacity protein LomR and related surface antigens	M	264	754	1018
COG1576	23S rRNA pseudoU1915 N3-methylase RlmH	J	5096	6284	11381
COG2211	Na+/melibiose symporter or related transporter	G	25179	32844	58023
COG1075	Triacylglycerol esterase/lipase EstA, alpha/beta hydrolase fold	I	3318	2872	6191
COG1293	Ribosome quality control (RQC) protein RqcH, Rqc2/NEMF/Tae2 family, contains fibronectin-(FbpA) and RNA- (NFACT) binding domains	J;O	7575	9602	17177
COG1180	Pyruvate-formate lyase-activating enzyme	O	8787	13209	21996
COG3489	Imelysin-like iron-regulated protein IrpA, duplicated M75 peptidase-like domain	R	611	432	1043
COG2938	Succinate dehydrogenase flavin-adding protein, antitoxin component of the CptAB toxin-antitoxin module	O	0	1	1
COG0059	Ketol-acid reductoisomerase	E;H	2769	3756	6524
COG5583	Uncharacterized conserved protein YezD	S	1	1	2
COG0132	Dethiobiotin synthetase	H	1096	1768	2864
COG0694	Fe-S cluster biogenesis protein NfuA, 4Fe-4S-binding domain	O	170	361	531
COG2957	Agmatine/peptidylarginine deiminase	E	2727	3163	5890
COG2232	Pyrrolysine biosynthesis ligase PylC and related enzymes, ATP-grasp superfamily	E	98	27	126
COG1541	Phenylacetate-coenzyme A ligase PaaK, adenylate-forming domain family	H	10974	13992	24966

COG1262	Formylglycine-generating enzyme, required for sulfatase activity, contains SUMF1/FGE domain	O	5558	6778	12336
COG0250	Transcription termination/antitermination protein NusG	K	9976	13079	23055
COG3474	Cytochrome c2	C	3	434	437
COG1221	Transcriptional regulators containing an AAA-type ATPase domain and a DNA-binding domain	K;T	66	668	734
COG3704	Type IV secretory pathway, VirB6 component	U	23	224	247
COG4530	Uncharacterized conserved protein	S	5	0	5
COG0539	Ribosomal protein S1	J	14956	18657	33613
COG1474	Cdc6-related protein, AAA superfamily ATPase	L	966	776	1743
COG0503	Adenine/guanine phosphoribosyltransferase or related PRPP-binding protein	F	4569	6787	11356
COG0336	tRNA G37 N-methylase TrmD	J	7125	8702	15827
COG5616	TolB amino-terminal domain (function unknown)	T	13	28	41
COG3022	DNA-binding protein YaaA associated with the oxidative stress response	L	367	1005	1372
COG0231	Translation elongation factor P (EF-P)/translation initiation factor 5A (eIF-5A)	J	4133	5655	9787
COG1664	Cytoskeletal protein CcmA, bactofilin family	Z	3769	3838	7607
COG3603	ACT domain, ACT-7 family	T	495	592	1087
COG4416	Mu-like prophage FluMu protein Com	X	0	4	4
COG1150	Heterodisulfide reductase, subunit C	C	2408	2613	5021
COG3200	3-deoxy-D-arabino-heptulosonate 7-phosphate (DAHP) synthase, class II	E	21	5	26
COG3295	Uncharacterized conserved protein	S	469	511	980
COG0669	Phosphopantetheine adenylyltransferase	H	5270	5878	11148

COG3005	Tetraheme cytochrome c subunit NapC of nitrate or TMAO reductase	C	80	220	300
COG0279	Phosphoheptose isomerase	G	2031	2716	4746
COG3136	Dual-topology membrane protein YdgC, GlpM family	S	0	1	1
COG1465	3-dehydroquinate synthase, class II	E	23	34	57
COG3642	tRNA A-37 threonylcarbamoyl transferase component Bud32	J	1584	1866	3450
COG4558	ABC-type hemin transport system, periplasmic component	P	0	2	2
COG5458	Uncharacterized conserved protein, DUF1489 domain	S	0	2	2
COG4756	Predicted cation transporter	R	0	36	37
COG0054	6,7-dimethyl-8-ribityllumazine synthase (Riboflavin synthase beta chain)	H	2150	2623	4773
COG1063	Threonine dehydrogenase or related Zn-dependent dehydrogenase	E;R	7869	15806	23675
COG1352	Methylase of chemotaxis methyl-accepting proteins	T	3227	3671	6898
COG1273	Non-homologous end joining protein Ku, dsDNA break repair	L	614	1020	1634
COG3853	Uncharacterized conserved protein YaaN involved in tellurite resistance	V	3405	4121	7527
COG4233	Thiol-disulfide interchange protein, contains DsbC and DsbD domains	O;C	99	89	189
COG0562	UDP-galactopyranose mutase	M	7345	8189	15534
COG2801	Transposase InsO and inactivated	X	43730	48294	92024
COG3055	N-acetylneuraminic acid mutarotase	M	898	2127	3024
COG1483	Predicted ATPase, AAA+ superfamily	R	293	1177	1470
COG3218	ABC-type uncharacterized transport system, auxiliary component	R	58	114	172

COG0740	ATP-dependent protease ClpP, protease subunit	O	17271	20360	37631
COG1551	sRNA-binding carbon storage regulator CsrA	T	1244	1098	2342
COG0264	Translation elongation factor EF-Ts	J	8199	10854	19053
COG1249	Dihydrolipoamide dehydrogenase (E3) component of pyruvate/2-oxoglutarate dehydrogenase complex or glutathione oxidoreductase	C	5495	9570	15065
COG2936	Predicted acyl esterase	R	398	1583	1981
COG2119	Putative Ca2+/H+ antiporter, TMEM165/GDT1 family	R	85	177	262
COG2172	Anti-sigma regulatory factor (Ser/Thr protein kinase)	T	5413	6289	11702
COG4717	Uncharacterized conserved protein YhaN, contains AAA domain	S	3151	5931	9082
COG1080	Phosphoenolpyruvate-protein kinase (PTS system EI component in bacteria)	G	7430	8251	15682
COG2190	Phosphotransferase system IIA component	G	967	1577	2543
COG5294	Uncharacterized conserved protein YxeA, DUF1093 family	S	425	378	803
COG1462	Curli biogenesis system outer membrane secretion channel CsgG	M	661	865	1526
COG0391	Archaeal 2-phospho-L-lactate transferase/Bacterial gluconeogenesis factor, CofD/UPF0052 family	G;H	1426	2763	4190
COG0756	dUTP pyrophosphatase (dUTPase)	F;V	5808	8026	13834
COG5846	Stage VI sporulation protein SpoVID (LysM domain), required for assembly of the spore coat	D	0	1	1

COG4665	TRAP-type mannitol/chloroaromatic compound transport system, small permease component	Q	25	133	158
COG1014	Pyruvate:ferredoxin oxidoreductase or related 2-oxoacid:ferredoxin oxidoreductase, gamma subunit	C	6274	8118	14392
COG2095	Small neutral amino acid transporter SnatA, MarC family	E	753	1403	2156
COG1598	Antitoxin component HicB of the HicAB toxin-antitoxin system	V	4493	4908	9402
COG1560	Palmitoleoyl-ACP: Kdo2-lipid-IV acyltransferase (lipid A biosynthesis)	I	2988	2747	5735
COG0696	Phosphoglycerate mutase (BPG-independent), AlkP superfamily	G	9385	12944	22329
COG2327	Polysaccharide pyruvyl transferase family protein WcaK (colanic acid biosynthesis)	M	6388	7417	13806
COG1276	Putative copper export protein	P	2	6	8
COG2373	Uncharacterized conserved protein YfaS, alpha-2-macroglobulin family	R	7611	8629	16239
COG2331	Predicted nucleic acid-binding protein, contains Zn-ribbon domain	R	85	190	274
COG4779	ABC-type enterobactin transport system, permease component	P	1	11	12
COG5492	Uncharacterized conserved protein YjdB, contains Ig-like domain	R	51630	64138	115768
COG5631	Predicted transcription regulator, contains HTH domain, MarR family	K	0	5	5
COG2003	DNA repair protein RadC, contains a helix-hairpin-helix DNA-binding motif	L	9438	11403	20841
COG4219	Signal transducer regulating beta-lactamase production, contains metallopeptidase domain	T	34578	38956	73533

COG2923	Sulfur transfer complex TusBCD TusC component, DsrF family	J	0	2	2
COG4997	Predicted house-cleaning noncanonical NTP pyrophosphatase, all-alpha NTP-PPase (MazG) superfamily	R	440	503	944
COG3502	Uncharacterized conserved protein, DUF952 family	S	18	25	44
COG4802	Ferredoxin-thioredoxin reductase, catalytic subunit	C	7	12	19
COG3155	Glyoxalase ElbB, DJ-1 superfamily	V	5	66	71
COG4974	Site-specific recombinase XerD	L	195678	242727	438405
COG3865	Glyoxalase superfamily enzyme, possible 3-demethylubiquinone-9 3-methyltransferase	R	3	2	5
COG1473	Metal-dependent amidase/aminoacylase/carboxypeptidase	R	5474	10251	15725
COG1305	Transglutaminase-like enzyme, putative cysteine protease	O	18116	20847	38963
COG1915	Uncharacterized conserved protein AF1278, contains saccharopine dehydrogenase N-terminal (SDHN) domain	S	109	666	774
COG1970	Large-conductance mechanosensitive	M	4688	5644	10332
COG0848	Biopolymer transport protein ExbD	U	5882	6443	12325
COG5474	NAD(P)H dehydrogenase cyanobacteria/chloroplast-specific assembly factor CRR6	C	32	149	181
COG1392	Phosphate transport regulator YkaA, distantly related to PhoU, UPF0111/DUF47 family	P	181	530	712
COG4466	Uncharacterized conserved protein Veg, DUF1021 family	S	185	387	573
COG1361	S-layer protein MJ0795, predicted component of type IV pili-like system	R	2828	3180	6008

COG1911	Ribosomal protein L30E	J	16	8	24
COG0814	Amino acid permease	E	57	35	92
COG5950	CRISPR-Cas system type II-D effector complex subunit Csx10, RAMP superfamily Cas5 group	V	11	119	130
COG1522	DNA-binding transcriptional regulator, Lrp family	K	4574	5646	10220
COG3294	Metal-dependent phosphatase/phosphodiesterase, HD supefamily	R	1759	2193	3952
COG4249	Uncharacterized conserved protein, contains caspase domain	R	2939	3760	6699
COG0550	DNA topoisomerase IA	L	31037	41210	72247
COG2940	SET domain-containing protein (function unknown)	R	97	70	168
COG4551	Predicted protein tyrosine phosphatase	R	52	28	79
COG0297	Glycogen synthase	G	8725	11255	19980
COG3171	Uncharacterized conserved protein YggL, DUF469 family	S	109	87	196
COG0047	Phosphoribosylformylglycinamidine (FGAM) synthase, glutamine	F	395	701	1096
COG3967	Short-chain dehydrogenase involved in D-alanine esterification of teichoic acids	M;I	6	11	17
COG0254	Ribosomal protein L31	J	956	1115	2071
COG3723	Recombinational DNA repair protein RecT	L	1674	2654	4328
COG0605	Superoxide dismutase	P	2715	3553	6269
COG3524	Capsule polysaccharide export protein KpsE/RkpR	M	24	45	68
COG0275	16S rRNA C1402 N4-methylase RsmH	J	10226	12978	23204
COG0412	Dienelactone hydrolase	Q	571	1470	2040
COG3680	Uncharacterized protein, contains GIY-YIG domain	S	52	64	117

COG0103	Ribosomal protein S9	J	2803	4056	6859
COG2746	Aminoglycoside N3'-acetyltransferase	V	724	1174	1898
COG1057	Nicotinate-nucleotide adenylyltransferase NadD	H	7738	11185	18923
COG0461	Orotate phosphoribosyltransferase	F	7576	9414	16990
COG5837	Stage V sporulation protein SpoVAD, subunit of dipicolinate uptake complex	D;E	6291	7866	14157
COG0504	CTP synthase (UTP-ammonia lyase)	F	9298	12917	22215
COG4319	Ketosteroid isomerase homolog YybH	R	76	140	216
COG0236	Acyl carrier protein	I	5346	6734	12080
COG2140	Oxalate decarboxylase/archaeal phosphoglucose isomerase, cupin	G	58	248	306
COG1399	23S rRNA accumulation protein YceD (essential in plants, uncharacterized in bacteria)	J	4624	5591	10215
COG1569	Predicted nucleic acid-binding protein, contains PIN domain	R	1517	2171	3688
COG0806	Ribosomal 30S subunit maturation factor RimM, required for 16S rRNA processing	J	5214	6421	11635
COG1805	Na+-transporting NADH:ubiquinone oxidoreductase, subunit NqrB	C	2420	2315	4734
COG0803	ABC-type Zn uptake system ZnuABC, Zn-binding component ZnuA	P	5268	6602	11869
COG2835	RNA methyltransferase activator Trm112/YbaR	J	11	6	17
COG4910	Propanediol dehydratase, small subunit	Q	24	47	71
COG0703	Shikimate kinase	E	8391	9388	17778
COG1505	Prolyl endopeptidase PreP, S9A serine peptidase family	E	2250	2468	4717
COG2805	Type IV pilus assembly protein PilT, pilus retraction ATPase	N;W	5006	7158	12164
COG1869	D-ribose pyranose/furanose isomerase RbsD	G	93	219	312

COG3525	N-acetyl-beta-hexosaminidase	G	13882	19110	32992
COG1443	Isopentenyldiphosphate isomerase	I	3836	5691	9527
COG3006	Chromosome condensin MukBEF, kleisin-like MukF subunit	D	0	2	2
COG4987	ABC-type transport system involved in cytochrome bd biosynthesis, fused ATPase and permease components	C;O	157	262	419
COG0055	FoF1-type ATP synthase, beta subunit	C	4629	6257	10887
COG5834	Stage V sporulation protein SpoVAA, subunit of dipicolinate uptake complex	D;E	1333	1679	3013
COG1323	tRNA-Met C34-N4-acetylcytidine synthase TmcAL	J	6904	8552	15456
COG1030	Membrane-bound serine protease NfeD, ClpP class	O	1836	2385	4221
COG4206	Outer membrane cobalamin receptor protein BtuB	H	22230	24604	46834
COG3038	Cytochrome b561	C	2	5	7
COG3400	Uncharacterized protein AQ_414, contains TrkA and USP-like domains	R	17	0	17
COG4294	UV DNA damage repair endonuclease	L	81	180	260
COG1721	Uncharacterized conserved protein, DUF58 family, contains vWF domain	S	11701	11915	23617
COG1734	RNA polymerase-binding transcription factor DksA	K	1036	1572	2609
COG4321	Predicted DNA-binding protein, contains ribbon-helix-helix (RHH) domain	R	7	33	41
COG0111	Phosphoglycerate dehydrogenase or related dehydrogenase	H	12723	17546	30270
COG3028	Ribosomal 50S subunit-associated protein YjgA, DUF615 family	J	6	148	154
COG1441	O-succinylbenzoate synthase	H	250	575	825

COG1636	Epoxyqueuosine reductase QueH (tRNA modification)	J	4956	5842	10799
COG5902	Spore germination receptor GerABC, GerB subunit (amino acid transporter)	D	965	2230	3195
COG1461	Predicted kinase related to dihydroxyacetone kinase	R	8619	10021	18641
COG3299	Uncharacterized phage protein gp47/JayE	X	9650	9559	19209
COG2382	Enterochelin esterase or related enzyme	P	6005	6997	13002
COG5883	Spore coat protein CotJB	D	1770	2691	4460
COG0741	Soluble lytic murein transglycosylase or regulatory protein s (may contain LysM/invasin domain)	M	3099	4845	7944
COG2404	Oligoribonuclease NrnA/c-di-AMP phosphodiesterase DhhP, DHH superfamily	J;T	291	606	897
COG4574	Serine protease inhibitor ecotin	O	0	1	1
COG1837	Predicted RNA-binding protein YlqC, contains KH domain, UPF0109 family	R	1070	1396	2466
COG1490	D-aminoacyl-tRNA deacylase	J	4107	5225	9332
COG2057	Acyl-CoA:acetate/3-ketoacid CoA transferase, beta subunit	I	207	938	1145
COG0714	MoxR-like ATPase	R	18942	21803	40745
COG4935	Regulatory P domain of the subtilisin-like proprotein convertases and other proteases	O	429	256	685
COG0533	tRNA A37 threonylcarbamoyltransferase TsaD	J	11884	14345	26229
COG2264	Ribosomal protein L11 methylase PrmA	J	8866	10395	19261
COG2070	NAD(P)H-dependent flavin oxidoreductase YrpB, nitropropane dioxygenase family	R	3589	6306	9895
COG1070	Sugar (pentulose or hexulose) kinase	G	20036	28066	48103
COG3330	Uncharacterized conserved protein BH1414	S	113	427	540

COG0057	Glyceraldehyde-3-phosphate dehydrogenase/erythrose-4-phosphate dehydrogenase	G	5280	7326	12606
COG2962	Membrane protein RarD, contains two EamA domains, drug/metabolite transporter family	R	601	958	1560
COG5404	Cell division inhibitor SulA, prevents FtsZ ring assembly	D	0	1	1
COG3940	Beta-xylosidase, GH43 family	G	3674	4093	7767
COG0416	Acyl-ACP:phosphate acyltransferase (fatty acid/phospholipid biosynthesis)	I	7874	9981	17855
COG0181	Porphobilinogen deaminase	H	714	1924	2638
COG0386	Thioredoxin/glutathione peroxidase BtuE, reduces lipid peroxides	V;I	241	388	629
COG4580	Maltoporin (phage lambda and maltose receptor)	G	0	6	6
COG3825	Uncharacterized CoxE-like protein, contains von Willebrand factor type A	S	1158	1324	2482
COG0733	Na+-dependent transporter, SNF family	R	6175	9888	16064
COG0637	Beta-phosphoglucomutase, HAD	G	12573	14862	27435
COG3102	Uncharacterized conserved protein YecM, predicted metalloenzyme	R	0	1	1
COG0693	Protein/nucleotide deglycase, PfpI/YajL/DJ-1 family (repair of methylglyoxal-glycated proteins and nucleic acids)	V	7093	7872	14965
COG3087	Cell division protein FtsN	D	4303	6409	10712
COG1142	Fe-S-cluster-containing hydrogenase component 2	C	505	811	1316
COG4472	Uncharacterized conserved protein, UPF0297 family	S	858	1235	2093

COG3266	Cell division protein DamX, binds to the septal ring, contains C-terminal SPOR domain	D	3636	3424	7060
COG3359	Uncharacterized conserved protein YprB, contains RNaseH-like and TPR domains	R	1836	2319	4155
COG0079	Histidinol-phosphate/aromatic aminotransferase or cobyric acid decarboxylase	E	10292	14384	24676
COG4200	Predicted lantabiotic-exporting membrane pepmease, EfiE/EfiG/ABC2 family	V	4901	5540	10440
COG0344	Phospholipid biosynthesis protein PlsY, probable glycerol-3-phosphate acyltransferase	I	7008	8826	15834
COG1626	Neutral trehalase	G	336	504	839
COG0565	tRNA C32,U32 (ribose-2'-O)-methylase TrmJ or a related methyltransferase	J	26	274	300
COG4863	YycH protein, regulator of the WalKR two-component signal transduction system	T	2	136	138
COG3402	Uncharacterized membrane protein YdbS, contains bPH2 (bacterial pleckstrin homology) domain	S	1543	1470	3013
COG3217	N-hydroxylaminopurine reductase subunit YcbX, contains MOSC domain	F;V	1	4	5
COG5701	Photosystem I reaction center subunit A1, PsaA	C	17	19	35
COG0517	CBS domain	T	1882	2336	4218
COG2235	Arginine deiminase	E	700	1628	2327
COG5473	Uncharacterized membrane protein	S	0	27	28
COG1947	4-diphosphocytidyl-2C-methyl-D-erythritol kinase	I	8504	10216	18720
COG3274	Surface polysaccharide O-acyltransferase WecH	M	10151	11957	22108

COG4430	Uncharacterized conserved protein YdeI, YjbR/CyaY-like superfamily, DUF1801 family	S	177	129	305
COG4243	Vitamin K epoxide reductase (VKOR) family protein, predicted involvement in disulfide bond formation	R	202	41	244
COG0067	Glutamate synthase domain 1	E	7711	11396	19107
COG0002	N-acetyl-gamma-glutamylphosphate reductase	E	5924	7133	13057
COG0294	Dihydropteroate synthase	H	4550	5671	10221
COG1757	Na+/H+ antiporter NhaC/MleN	C	6424	12214	18638
COG5747	Light-independent protochlorophyllide reductase subunit N, BchN (=ChlN)	H	1	6	7
COG0701	Uncharacterized membrane protein YraQ, UPF0718 family	S	2293	2271	4564
COG4784	Putative Zn-dependent protease	R	27	211	239
COG3906	Uncharacterized conserved protein YrzB, UPF0473 family	S	1425	1699	3124
COG2369	Uncharacterized protein, contains phage Mu head morphogenesis gpF-like domain	X	5219	3640	8859
COG0127	Inosine/xanthosine triphosphate pyrophosphatase, all-alpha NTP-PPase family	F	8174	10107	18281
COG2881	GTPase-interacting Yip1 domain	R	11	19	30
COG5457	Uncharacterized conserved protein YjiS, DUF1127 family	S	0	1	1
COG3635	2,3-bisphosphoglycerate-independent phosphoglycerate mutase, archeal type	G	2856	3342	6198
COG3977	Alanine-alpha-ketoisovalerate (or valine-pyruvate) aminotransferase	E	0	563	564

COG0022	Pyruvate/2-oxoglutarate/acetoin dehydrogenase complex, dehydrogenase (E1) component, beta subunit	C	1024	2190	3214
COG3350	Heavy metal-bindng TRASH/YHS domain, predicted Cu/Ag metallochaperone	P	7	5	12
COG3595	Uncharacterized conserved protein YvlB, contains DUF4097 and DUF4098 domains	S	134	70	204
COG4412	Bacillopeptidase F, M6 metalloprotease family	O	3853	4291	8145
COG1320	Multisubunit Na+/H+ antiporter, MnhG subunit	P	743	830	1573
COG1332	CRISPR-Cas system type III CSM-effector complex subunit Csm5, RAMP superfamily Cas7 group	V	96	367	463
COG1555	DNA uptake protein ComE or related DNA-binding protein	L	5498	6122	11619
COG1102	Cytidylate kinase	F	8731	10353	19084
COG3479	Phenolic acid decarboxylase	Q	0	69	70
COG5806	Sporulation sensor histidine kinase B	D;T	31	95	126
COG4478	Uncharacterized membrane protein	S	661	1157	1818
COG3520	Predicted component of the type VI protein secretion system	U	119	96	215
COG1975	Molybdoenzyme maturation factor PaoD (Mo cofactor insertion), XdhC/CoxF family	O	818	1908	2726
COG4615	ABC-type siderophore export system, fused ATPase and permease components	P	0	2	2
COG1964	C-terminal domain of the GTP 3',8'-cyclase MoaA, radical SAM superfamily (molybdenum cofactor biosynthesis)	H	218	360	578
COG5055	Recombinational DNA repair protein (RAD52 pathway)	L	4	4	7
COG4752	Uncharacterized conserved protein	S	1	52	53

COG4384	Mu-like prophage protein gp45	X	60	22	82
COG1545	Uncharacterized OB-fold protein, contains Zn-ribbon domain	R	72	109	180
COG3854	Stage III sporulation protein SpoIIIAA	D	5557	6876	12433
COG4537	Competence protein ComGC	X	40	102	142
COG4231	TPP-dependent indolepyruvate ferredoxin oxidoreductase, alpha subunit	C	6069	8750	14819
COG5463	Uncharacterized conserved protein YgiB, UPF0441/DUF1190 family, involved in bioifilm formation	S	0	1	1
COG0014	Gamma-glutamyl phosphate reductase	E	5822	7291	13114
COG2050	Acyl-CoA thioesterase PaaI, contains HGG motif	Q	1512	2638	4149
COG0832	Urease beta subunit	E	913	994	1907
COG3885	Aromatic ring-opening dioxygenase, LigB subunit	Q	4	0	4
COG3109	sRNA-binding protein ProQ	T	2	3	5
COG2390	DNA-binding transcriptional regulator LsrR, DeoR family	K	746	1194	1940
COG5442	Flagellar biosynthesis regulator FlaF	N	0	8	8
COG0221	Inorganic pyrophosphatase	C	2461	2472	4933
COG3021	Uncharacterized conserved protein YafD, endonuclease/exonuclease/phosphatase (EEP) superfamily	R	989	993	1981
COG3659	Carbohydrate-selective porin OprB	M	319	1385	1704
COG0651	Formate hydrogenlyase subunit 3/Multisubunit Na+/H+ antiporter, MnhD subunit	C;P	9522	11538	21060
COG1355	Predicted class III extradiol dioxygenase, MEMO1 family	R	38	95	134
COG0252	L-asparaginase/archaeal Glu-tRNAGln amidotransferase subunit D	J;E	7372	8814	16186

COG0654	2-polyprenyl-6-methoxyphenol hydroxylase and related FAD-dependent oxidoreductases	H;C	187	294	481
COG1060	2-iminoacetate synthase ThiH/Menaquinone biosynthesis enzymes MqnC and MqnE	H	5044	6566	11611
COG2306	Predicted RNA-binding protein, associated with RNAse of E/G family, DUF402 domain	R	1563	1394	2956
COG0112	Glycine/serine hydroxymethyltransferase	E	7837	10493	18330
COG4734	Antirestriction protein ArdA	V	959	1027	1986
COG2944	DNA-binding transcriptional regulator YiaG, XRE-type HTH domain	K	2395	2526	4921
COG5660	Anti-sigma-YlaC factor YlaD, contains Zn-finger domain	T	3698	3948	7646
COG0438	Glycosyltransferase involved in cell wall bisynthesis	M	109081	137776	246857
COG1539	Dihydroneopterin aldolase	H	1298	1300	2598
COG0782	Transcription elongation factor, GreA/GreB family	K	6230	8172	14401
COG5573	Predicted nucleic acid-binding protein, contains PIN domain	R	363	239	603
COG4474	Uncharacterized SPBc2 prophage-derived protein YoqJ	X	7770	8317	16087
COG1176	ABC-type spermidine/putrescine transport system, permease component I	E	4041	5819	9860
COG0194	Guanylate kinase	F	8290	10040	18330
COG2901	DNA-binding protein Fis (factor for inversion stimulation)	K	3	0	3
COG1245	Translation initiation factor RLI1, contains Fe-S and AAA+ ATPase domains	J	7	17	24
COG3150	Predicted esterase YcpF, UPF0227 family	R	461	1021	1482
COG0334	Glutamate dehydrogenase/leucine dehydrogenase	E	7853	10242	18096
COG2326	Polyphosphate kinase 2, PPK2 family	C	1949	2669	4618

COG3953	SLT domain protein	X	21	37	58
COG5905	Spore transcriptional regulator GerE (stand-alone HTH domain)	D;K	54	75	129
COG3944	Capsular polysaccharide biosynthesis protein YveK	M	3177	4136	7312
COG3296	Uncharacterized Tic20-related protein, DUF4870 domain	R	9	198	207
COG5256	Translation elongation factor EF-1alpha (GTPase)	J	148	127	275
COG3341	Ribonuclease HI-related protein, contains viroplasmin and RNaseH domains	R	1853	2320	4173
COG1061	Superfamily II DNA or RNA helicase	K;L	23885	33598	57483
COG4189	Predicted transcriptional regulator, ArsR family	K	1705	1624	3329
COG0507	ATP-dependent exoDNAse (exonuclease V), alpha subunit, helicase superfamily I	L	27300	33651	60951
COG3383	Predicted molibdopterin-dependent oxidoreductase YjgC	R	852	1970	2822
COG1006	Multisubunit Na+/H+ antiporter, MnhC subunit	P	679	737	1415
COG3856	Small basic protein Sbp (function unknown), DUF1290 domain	S	141	420	561
COG0091	Ribosomal protein L22	J	2972	3706	6678
COG1222	ATP-dependent 26S proteasome regulatory subunit	O	1266	1611	2877
COG3031	Type II secretory pathway, component PulC	U	0	2	2
COG0598	Mg2+ and Co2+ transporter CorA	P	4031	5823	9855
COG4659	Na+-translocating ferredoxin:NAD+ oxidoreductase RNF, RnfG subunit	C	5261	5387	10647
COG0462	Phosphoribosylpyrophosphate synthetase	F	9061	13983	23044
COG3455	Type VI protein secretion system component TssL/VasF/DotU	U	10	3	13

COG0168	Trk-type K+ transport system, membrane component	P	18339	22986	41325
COG3962	TPP-dependent trihydroxycyclohexane-1,2-dione (THcHDO) dehydratase, myo-inositol metabolism	G	596	934	1530
COG0555	ABC-type sulfate transport system, permease component	P	319	212	531
COG2826	Transposase and inactivated derivatives, IS30 family	X	39459	35723	75181
COG4968	Type IV pilus assembly protein PilE	N;W	368	1493	1860
COG2388	Predicted acetyltransferase, GNAT superfamily	R	729	753	1482
COG3832	Chalcone/flavanone-binding protein YndB, AHSA1/START/SRPBCC domain	I	100	151	251
COG0331	Malonyl CoA-acyl carrier protein transacylase	I	6197	7954	14151
COG0157	Nicotinate-nucleotide pyrophosphorylase	H	4404	4537	8941
COG1362	Aspartyl aminopeptidase	E	11599	14335	25934
COG0676	D-hexose-6-phosphate mutarotase	G	285	724	1009
COG4071	Gamma-glutamyl ligase family protein MJ1361	R	637	772	1409
COG2041	Molybdopterin-dependent catalytic subunit of periplasmic DMSO/TMAO and protein-methionine-sulfoxide reductases	C	240	535	774
COG4983	Primase-polymerase (Primpol) domain protein	X	1426	1177	2603
COG5426	Uncharacterized protein STM3548, contains class I glutamine amidotransferase domain	R	268	2597	2865
COG2253	Predicted nucleotidyltransferase component of viral defense system	V	9187	11711	20899
COG0025	NhaP-type Na+/H+ or K+/H+ antiporter	P	1552	1389	2941

COG1594	DNA-directed RNA polymerase, subunit M/Transcription elongation factor TFIIS	K	2	0	2
COG3424	Predicted naringenin-chalcone synthase	Q	2	2	4
COG4729	Uncharacterized conserved protein, DUF1850 family	S	79	160	239
COG3223	Phosphate starvation-inducible membrane PsiE (function unknown)	R	154	322	476
COG1699	Flagellar assembly factor FliW	N	1799	1607	3406
COG1752	Predicted acylesterase/phospholipase RssA, containd patatin domain	R	6877	8844	15720
COG0152	Phosphoribosylaminoimidazole-succinocarboxamide synthase	F	5876	7456	13333
COG0040	ATP phosphoribosyltransferase	E	3126	4456	7582
COG5640	Secreted trypsin-like serine protease	O	71	90	161
COG3211	Secreted phosphatase, PhoX family	R	49	87	136
COG0827	Adenine-specific DNA N6-methylase	L	2439	2260	4699
COG1290	Cytochrome b subunit of the bc complex	C	261	234	495
COG3481	3'-5' exoribonuclease YhaM, can participate in 23S rRNA maturation, HD superfamily	J	6044	8241	14285
COG3539	Pilin (type 1 fimbrial protein)	N	0	43	43
COG0617	tRNA nucleotidyltransferase/poly(A) polymerase	J	12291	15308	27599
COG4889	Predicted helicase	R	3434	2865	6298
COG0729	Outer membrane translocation and assembly module TamA	M	41	44	86
COG1045	Serine acetyltransferase	E	9706	12171	21877
COG4106	Trans-aconitate methyltransferase	C	1848	2310	4157
COG1611	Nucleotide monophosphate nucleosidase PpnN/YdgH, Lonely Guy (LOG) family	F	2243	3307	5551
COG0650	Formate hydrogenlyase subunit HyfC	C	959	1145	2104

COG1762	Phosphotransferase system mannitol/fructose-specific IIA domain (Ntr-type)	G;T	2084	4592	6676
COG2706	6-phosphogluconolactonase, cycloisomerase 2 family	G	1778	3341	5119
COG2913	Outer membrane protein assembly factor BamE, lipoprotein component of the BamABCDE complex	M	9	184	193
COG2045	Phosphosulfolactate phosphohydrolase or related enzyme	H	106	322	429
COG2184	Fido, protein-threonine AMPylation domain	T	6053	6436	12488
COG0272	NAD-dependent DNA ligase	L	14956	19615	34571
COG5879	Spore coat protein CotD	D	0	3	3
COG2421	Acetamidase/formamidase	C	95	472	567
COG2204	DNA-binding transcriptional response regulator, NtrC family, contains REC, AAA-type ATPase, and a Fis-type DNA-binding domains	T	10842	15037	25879
COG5277	Actin-related protein	Z	42	31	73
COG0282	Acetate kinase	C	8608	12283	20892
COG0652	Peptidyl-prolyl cis-trans isomerase (rotamase) - cyclophilin family	O	10121	11127	21248
COG0261	Ribosomal protein L21	J	2870	3454	6324
COG3671	Uncharacterized membrane protein	S	0	95	95
COG5868	Sporulation protein YabP	D	2181	2669	4850
COG0769	UDP-N-acetylmuramyl tripeptide synthase	M	15090	20817	35907
COG4448	L-asparaginase II	E	154	328	482
COG1668	ABC-type Na+ efflux pump, permease component NatB	C;P	10616	9683	20299
COG0178	Excinuclease UvrABC ATPase subunit	L	20374	28836	49210
COG2886	Predicted antitoxin, contains HTH domain	R	94	49	142

COG3781	Predicted membrane chloride channel, bestrophin family	P	0	2	2
COG0020	Undecaprenyl pyrophosphate synthase	I	7906	10102	18008
COG4662	ABC-type tungstate transport system, periplasmic component	P	101	247	348
COG0831	Urease gamma subunit	E	525	539	1064
COG0375	Hydrogenase maturation factor HypA/HybF, metallochaperone involved in Ni insertion	O	503	998	1501
COG4823	Abortive infection bacteriophage resistance protein	V	5988	5186	11174
COG3144	Flagellar hook-length control protein FliK	N	3520	3848	7367
COG3654	Prophage maintenance system killer protein	X	1312	1329	2641
COG2739	Predicted DNA-binding protein YlxM, UPF0122 family	K	2952	3345	6297
COG5301	Phage-related tail fiber protein	X	1870	2734	4604
COG0787	Alanine racemase	M	11776	15541	27317
COG1497	Predicted transcriptional regulator MJ0558, contains a CRP-type HTH domain	K	4	3	7
COG1418	HD superfamily phosphodieaserase, includes HD domain of RNase Y	J;R	10196	11145	21341
COG1821	Tyramine-glutamate ligase MfnD (methanofuran biosynthesis), ATP-grasp superfamily	H	6	0	6
COG4282	Cell wall assembly regulator SMI1 (Killer toxin-resistance protein 4)	M	254	274	528
COG4405	Predicted RNA-binding protein YhfF, contains PUA-like ASCH domain	R	107	225	332
COG1312	D-mannonate dehydratase	G	3569	4510	8079
COG1893	Ketopantoate reductase	H	2616	4267	6883
COG3377	Uncharacterized conserved protein YunC, DUF1805 family	S	38	36	74

COG1905	NADH:ubiquinone oxidoreductase 24 kD subunit (chain E)	C	2885	4086	6970
COG4638	Phenylpropionate dioxygenase or related ring-hydroxylating dioxygenase, large terminal subunit	P;R	19	37	56
COG2871	Na+-transporting NADH:ubiquinone oxidoreductase, subunit NqrF	C	1890	2027	3917
COG3683	ABC-type uncharacterized transport system, periplasmic component	R	0	2	2
COG0277	FAD/FMN-containing lactate dehydrogenase/glycolate oxidase	C	1049	2114	3163
COG2524	Predicted transcriptional regulator, contains C-terminal CBS domains	K	68	188	256
COG1393	Arsenate reductase or related protein, glutaredoxin family	P	1621	2436	4057
COG0486	tRNA U34 5-carboxymethylaminomethyl modifying GTPase MnmE/TrmE	J	13060	16221	29281
COG0513	Superfamily II DNA and RNA helicase	L	11758	15926	27684
COG0753	Catalase	P	673	1549	2222
COG1121	ABC-type Mn2+/Zn2+ transport system, ATPase component	P	4723	6033	10755
COG5525	Phage terminase, large subunit GpA	X	3220	3175	6395
COG1873	Sporulation protein YlmC, YlmC/YmxH family, contains PRC-barrel domain	R	1265	1805	3070
COG0849	Cell division ATPase FtsA	D	10113	10909	21023
COG5267	Uncharacterized conserved protein, DUF1800 family	S	7	22	29
COG1925	HPr or related phosphotransfer protein	T;G	3724	4338	8062
COG0793	C-terminal processing protease CtpA/Prc, contains a PDZ domain	O	27128	29845	56973
COG2610	H+/gluconate symporter GntT or related permease, GntP/DsdX family	G	1251	3570	4821

COG0435	Glutathionyl-hydroquinone reductase	C	1	101	102
COG2001	MraZ, DNA-binding transcriptional regulator and inhibitor of RsmH methyltransferase activity	J	3381	4361	7742
COG3673	Uncharacterized conserved protein, PA2063/DUF2235 family	S	31	28	59
COG2917	Intracellular septation protein A	D	0	106	107
COG4577	Carboxysome shell and ethanolamine utilization microcompartment protein CcmL/EutN	Q;C	638	1669	2307
COG3681	L-cysteine desulfidase YhaM	E	4190	6959	11149
COG1105	1-phosphofructokinase or 6-phosphofructokinase II	G	4468	4965	9434
COG0568	DNA-directed RNA polymerase, sigma subunit (sigma70/sigma32)	K	14189	18901	33090
COG4535	Mg2+ and Co2+ transporter CorC, contains CBS pair and CorC-HlyC domains	P	3	198	201
COG0615	Glycerol-3-phosphate cytidylyltransferase, cytidylyltransferase family	M	3159	4255	7413
COG0235	5-methylthioribulose/5-deoxyribulose/Fuculose 1-phosphate aldolase (methionine salvage, sugar degradation)	E;G	8630	10822	19453
COG1096	Exosome complex RNA-binding protein Csl4, contains S1 and Zn-ribbon domains	U	2	2	3
COG2189	Adenine specific DNA methylase Mod	L	11589	14778	26367
COG4664	TRAP-type mannitol/chloroaromatic compound transport system, large permease component	Q	57	128	185
COG1470	Uncharacterized membrane protein	S	339	780	1120
COG0415	Deoxyribodipyrimidine photolyase	L	4	10	15

COG1044	UDP-3-O-[3-hydroxymyristoyl] glucosamine N-acyltransferase	M	6561	8223	14785
COG0307	Riboflavin synthase alpha chain	H	2641	3196	5837
COG3159	Uncharacterized conserved protein YigA, DUF484 family	S	0	4	4
COG2519	tRNA A58 N-methylase Trm61	J	532	854	1386
COG0269	3-keto-L-gulonate-6-phosphate decarboxylase	G	37	245	281
COG3233	Predicted deacetylase	R	1236	923	2159
COG3930	Uncharacterized conserved protein, DUF1704 domain	S	6	0	6
COG5765	Uncharacterized cyanobacterial/chloroplast stress-induced protein Ycf35, DUF1257 domain	C	0	0	0
COG5271	Midasin, AAA ATPase with vWA domain, involved in ribosome maturation	J	390	472	862
COG2405	Predicted nucleic acid-binding protein, contains PIN domain	R	645	811	1456
COG4677	Pectin methylesterase and related acyl-CoA thioesterases	G;I	10130	10198	20328
COG1072	Panthothenate kinase	H	20	137	157
COG3307	O-antigen ligase	M	5702	7981	13683
COG3008	Intermembrane transporter PqiABC subunit PqiB	M	18	81	99
COG0260	Leucyl aminopeptidase	E	245	949	1194
COG1404	Serine protease, subtilisin family	O	33062	44834	77896
COG1728	Uncharacterized conserved protein YaaR, TM1646/DUF327 family	S	810	855	1665
COG3018	Flagellar basal body lipoprotein FlgP, LPP20 family	N	22	283	305
COG2064	Flp pilus assembly protein TadC	W	1899	1947	3846
COG1085	Galactose-1-phosphate uridylyltransferase	G	209	364	573

COG3836	2-keto-3-deoxy-L-rhamnonate aldolase RhmA	G	965	1784	2749
COG2522	Predicted transcriptional regulator, contains XRE-type HTH domain	K	12	36	48
COG1036	Archaeal flavoprotein	C	23	8	31
COG4689	Acetoacetate decarboxylase	Q	14	60	75
COG4229	Enolase-phosphatase E1 involved in merthionine salvage	E	1	3	4
COG3504	Type IV secretory pathway, VirB9 components	U	94	533	627
COG2149	Uncharacterized membrane protein YidH, DUF202 family	S	4	2	6
COG1554	Kojibiose phosphorylase YcjT	G	11589	11742	23331
COG0408	Coproporphyrinogen-III oxidase HemH, oxygen-dependent	H	1	5	6
COG3226	DNA-binding transcriptional regulator YbjK	K	10	21	30
COG2031	Short chain fatty acids transporter	I	174	492	666
COG4953	Membrane carboxypeptidase/penicillin-binding protein PbpC	M	925	744	1669
COG1977	Molybdopterin synthase sulfur carrier subunit MoaD	H	19	88	108
COG3665	Uncharacterized conserved protein YcgI, DUF1989 family	S	36	22	58
COG0802	tRNA A37 threonylcarbamoyladenosine biosynthesis protein TsaE	J	4707	6062	10769
COG3778	Uncharacterized conserved protein YmfQ in lambdoid prophage, DUF2313 family	X	25	100	125
COG1957	Inosine-uridine nucleoside N-ribohydrolase	F	3110	5522	8632
COG3242	Uncharacterized conserved protein YjeT, DUF2065 family	S	1	2	2

COG2378	Predicted DNA-binding transcriptional regulator YobV, contains HTH and WYL domains	K	19073	20920	39993
COG0514	Superfamily II DNA helicase RecQ	L	17615	17931	35546
COG4916	Uncharacterized conserved protein	S	1102	654	1756
COG3909	Cytochrome c556	C	1	2	4
COG0607	Rhodanese-related sulfurtransferase	P	1097	2350	3447
COG3685	Ferritin-like metal-binding protein YciE	P	772	534	1306
COG5832	Stage IV sporulation protein SpoIVB, serine protease, degrades SpoIIQ, SpoIVFA	D;O	6907	8733	15640
COG3801	Uncharacterized conserved protein	S	2	0	2
COG4642	Uncharacterized conserved protein	S	570	383	953
COG1196	Chromosome segregation ATPase Smc	D	35602	48247	83849
COG0255	Ribosomal protein L29	J	986	1151	2137
COG0417	DNA polymerase B elongation subunit	L	454	6748	7201
COG0859	ADP-heptose:LPS heptosyltransferase	M	3239	5264	8503
COG0712	FoF1-type ATP synthase, delta subunit	C	4283	4503	8787
COG4119	Predicted NTP pyrophosphohydrolase, NUDIX family	F;R	18	51	70
COG3943	Uncharacterized protein RhuM, Salmonella virulence factor	S	13850	17662	31512
COG1100	GTPase SAR1 family domain	R	60	72	131
COG0797	Peptidoglycan lytic transglycosylase RlpA, contains C-terminal SPOR domain	M	273	992	1265
COG3485	Protocatechuate 3,4-dioxygenase beta subunit	Q	51	123	174
COG3735	Uncharacterized conserved protein YbaP, TraB family	S	1788	1227	3015
COG2825	Periplasmic chaperone for outer membrane proteins, Skp family	M;O	4566	4688	9253
COG0576	Molecular chaperone GrpE (heat shock protein HSP-70)	O	7331	8893	16224

COG4986	ABC-type anion transport system, duplicated permease component	P	16	20	36
COG0625	Glutathione S-transferase	O	53	313	366
COG0716	Flavodoxin	C	20830	22218	43048
COG4197	DNA-binding transcriptional regulator YdaS, prophage-encoded, Cro superfamily	K	231	211	442
COG3843	Type IV secretory pathway, VirD2 component (relaxase)	U	11706	12793	24499
COG1997	Ribosomal protein L37AE/L43A	J	24	4	28
COG0403	Glycine cleavage system protein P (pyridoxal-binding), N-terminal domain	E	3852	4911	8763
COG2127	ATP-dependent Clp protease adapter protein ClpS	O	51	184	235
COG3422	Uncharacterized conserved protein YegP, UPF0339 family	S	1024	1083	2107
COG2334	Ser/Thr protein kinase RdoA involved in Cpx stress response, MazF antagonist	T	5140	8426	13566
COG4225	Rhamnogalacturonyl hydrolase YesR	G	17902	16757	34659
COG0781	Transcription antitermination protein NusB	K	8812	10122	18934
COG1613	ABC-type sulfate transport system, periplasmic component	P	330	178	508
COG5341	Uncharacterized conserved protein	S	2073	2180	4253
COG4933	Predicted transcriptional regulator, contains an HTH and PUA-like domains	K	586	462	1047
COG4186	Uncharacterized conserved protein MJ1445, calcineurin-like phosphoesterase superfamily	R	1473	3320	4793
COG0824	Acyl-CoA thioesterase FadM	I	3956	4968	8924
COG3960	Glyoxylate carboligase	Q	37	51	88
COG3621	Patatin-like phospholipase/acyl hydrolase, includes sporulation protein CotR	R	446	569	1015
COG4451	Ribulose bisphosphate carboxylase small subunit	G	94	20	115

COG1840	ABC-type Fe3+ transport system, periplasmic component	P	1624	3376	5001
COG0860	N-acetylmuramoyl-L-alanine amidase	M	28577	35280	63857
COG4261	Predicted acyltransferase, LPLAT superfamily	R	130	371	501
COG3718	5-deoxy-D-glucuronate isomerase	G	484	709	1192
COG0213	Thymidine phosphorylase	F	3641	5208	8849
COG3378	DNA primase, phage- or plasmid-associated	X	9218	11268	20486
COG1173	ABC-type dipeptide/oligopeptide/nickel transport system, permease component	E;P	9692	17774	27467
COG3057	Negative regulator of replication initiation SeqA	L	0	3	3
COG2147	Ribosomal protein L19E	J	10	7	17
COG4709	Uncharacterized membrane protein	S	3975	4909	8883
COG0528	Uridylate kinase	F	6081	8433	14514
COG0340	Biotin-(acetyl-CoA carboxylase) ligase	H	9052	10757	19809
COG5309	Exo-beta-1,3-glucanase, GH17 family	G	43	25	68
COG3042	Putative hemolysin	R	377	505	882
COG3788	Uncharacterized membrane protein YecN, MAPEG domain	S	1	5	6
COG4487	Uncharacterized conserved protein, contains DUF2130 domain	S	735	1011	1745
COG5519	Predicted ATPase domain of Cch-like helicases, DUF927 family	R	1593	1125	2718
COG3952	Uncharacterized N-terminal domain of lipid-A-disaccharide synthase	R	202	276	479
COG1054	tRNA U34 5'-hydroxylase TrhO, rhodanese family	J	2	2	5
COG2916	DNA-binding protein H-NS	K	0	146	147
COG2024	O-phosphoseryl-tRNA(Cys) synthetase	J	31	79	110
COG0270	DNA-cytosine methylase	L	33161	39391	72552

COG4190	Predicted transcriptional regulator, contains HTH domain	K	1	1	2
COG1719	Predicted hydrocarbon binding protein, contains 4VR domain	R	123	253	376
COG4372	Uncharacterized protein, contains DUF3084 domain	S	8446	5678	14124
COG2199	GGDEF domain, diguanylate cyclase (c-di-GMP synthetase) or its enzymatically inactive variants	T	52008	45561	97570
COG1678	Putative transcriptional regulator, AlgH/UPF0301 family	K	379	1059	1438
COG0768	Cell division protein FtsI, peptidoglycan transpeptidase (Penicillin-binding protein 2)	D;M	30434	42170	72604
COG1724	Predicted RNA binding protein YcfA, dsRBD-like fold, HicA-like mRNA interferase family	R	776	688	1465
COG1729	Cell division protein CpoB, coordinates peptidoglycan biosynthesis and outer membrane constriction	D	7480	8997	16477
COG1388	LysM repeat	M	13072	16956	30028
COG5293	Uncharacterized conserved protein YydD, contains DUF2326 domain	S	1001	1053	2054
COG1263	Phosphotransferase system IIC components, glucose/maltose/N-acetylglucosamine-specific	G	6044	10158	16202
COG5822	Stage II sporulation protein SpoIIR, required for processing of pro-sigma-E	D	3514	4430	7944
COG1077	Cell shape-determining ATPase MreB, actin-like superfamily	D;Z	10419	12130	22549
COG3385	IS4 transposase InsG	X	6570	9908	16478
COG3001	Fructosamine-3-kinase	G	138	384	521

COG1212	CMP-2-keto-3-deoxyoctulosonic acid synthetase	M	1971	2715	4686
COG2833	Uncharacterized protein, contains ferritin-like DUF455 domain	S	20	1	21
COG0177	Endonuclease III	L	8496	10614	19109
COG3508	Homogentisate 1,2-dioxygenase	Q	6	3	9
COG2180	Nitrate reductase assembly protein NarJ, required for insertion of molybdenum cofactor	C;P;O	0	153	153
COG2051	Ribosomal protein S27E	J	11	12	24
COG3938	Proline racemase/hydroxyproline epimerase	E	49	128	177
COG2996	Predicted RNA-binding protein YitL, contains S1 domains, virulence factor B family	R	3715	4281	7996
COG3866	Pectate lyase	G	11033	10037	21070
COG1486	Alpha-galactosidase/6-phospho-beta-glucosidase, family 4 of glycosyl hydrolase	G	4523	4536	9059
COG5468	Predicted lipoprotein involved in lipopolysaccharide assembly	S	1	58	59
COG5026	Hexokinase	G	245	401	647
COG5818	Stage II sporulation protein SpoIIGA (protease, processing of pro-sigma-E to active sigma-E)	D;K;O	4767	5846	10612
COG1010	Precorrin-3B methylase	H	2617	3284	5901
COG3808	Na+ or H+-translocating membrane pyrophosphatase	C	3239	5547	8786
COG0772	Peptodoglycan polymerase FtsW/RodA/SpoVE	D	24380	31062	55443
COG4691	Plasmid stability protein StbC1, contains ribbon-helix-helix domain	V	1	2	3
COG5013	Nitrate reductase alpha subunit	C;P	12	393	404

COG3936	Membrane-bound acyltransferase YfiQ, involved in biofilm formation	G	813	668	1481
COG3813	Uncharacterized conserved protein, DUF1272 domain	S	0	0	0
COG4956	Uncharacterized conserved protein YacL, contains PIN and TRAM domains	R	203	713	917
COG4134	ABC-type uncharacterized transport system YnjBCD, periplasmic component	R	48	73	121
COG1912	Stereoselective (R,S)-S-adenosylmethionine hydrolase (adenosine-forming)	H;V	250	286	536
COG2162	Arylamine N-acetyltransferase	Q	147	229	376
COG0263	Glutamate 5-kinase	E	6309	7218	13526
COG5533	Ubiquitin C-terminal hydrolase	O	0	4	4
COG2214	Curved DNA-binding protein CbpA, contains a DnaJ-like domain	K	95	45	140
COG2046	ATP sulfurylase (sulfate adenylyltransferase)	P	144	183	327
COG4246	Uncharacterized conserved protein, contains a phytase-like domain	S	1	3	4
COG3033	Tryptophanase	E	881	2061	2942
COG3593	Predicted ATP-dependent endonuclease of the OLD family, contains P-loop ATPase and TOPRIM domains	L	8523	10498	19022
COG2514	Catechol-2,3-dioxygenase	Q	169	267	436
COG4658	Na+-translocating ferredoxin:NAD+ oxidoreductase RNF, RnfD subunit	C	8774	9380	18154
COG2361	HEPN domain protein, predicted toxin of MNT-HEPN system	V	1591	1820	3411
COG3730	Phosphotransferase system sorbitol-specific IIC component	G	214	340	554
COG2015	Alkyl sulfatase BDS1 and related hydrolases, metallo-beta-lactamase	Q	358	181	539
COG2309	Leucyl aminopeptidase (aminopeptidase T)	E	5701	9647	15348

COG5549	Predicted Zn-dependent protease	O	90	712	802
COG2384	tRNA A22 N1-methylase	J	4427	5273	9700
COG2985	Uncharacterized membrane protein YbjL, putative transporter	R	5102	6544	11646
COG5295	Autotransporter adhesin	U;W	710	1979	2690
COG2977	4'-phosphopantetheinyl transferase EntD (siderophore biosynthesis)	Q	26	14	40
COG4161	ABC-type arginine transport system, ATPase component	E	4	1	5
COG1971	Putative Mn2+ efflux pump MntP	P	6213	7420	13634
COG3509	Acetyl xylan esterase AxeA and related esterases, LpqC family	G	812	946	1759
COG3054	Predicted transcriptional regulator YtfJ	R	687	539	1226
COG3830	ACT domain, binds amino acids and other small ligands	T	1154	1359	2513
COG3291	Uncharacterized conserved protein, PKD repeat domain	S	12158	10529	22688
COG0382	4-hydroxybenzoate polyprenyltransferase	H	1433	2285	3718
COG3658	Cytochrome b subunit of Ni2+-dependent hydrogenase	C	1	1	3
COG3522	Predicted component of the type VI protein secretion system	U	122	67	189
COG4724	Endo-beta-N-acetylglucosaminidase D	G	623	804	1426
COG4302	Ethanolamine ammonia-lyase, small subunit	E	853	1399	2252
COG4245	Uncharacterized conserved protein YegL, contains vWA domain of TerY type	S	4319	4964	9283
COG0426	Flavorubredoxin	C	7375	9228	16602
COG3581	Predicted nucleotide-binding protein, sugar kinase/HSP70/actin superfamily	R	3636	4173	7809
COG5632	N-acetylmuramoyl-L-alanine amidase CwlA	M	4703	7126	11829
COG3432	Predicted transcriptional regulator	K	838	934	1772
COG3541	Predicted nucleotidyltransferase YcgL	R	1465	1396	2861

COG3288	NAD/NADP transhydrogenase alpha subunit	C	161	284	444
COG3025	Inorganic triphosphatase YgiF, contains CYTH and CHAD domains	P	612	709	1321
COG5268	Type IV secretory pathway, TrbD component	U	11	31	41
COG1587	Uroporphyrinogen-III synthase	H	2564	2527	5092
COG1205	ATP-dependent helicase YprA, contains C-terminal metal-binding DUF1998 domain	L	3873	5885	9758
COG1087	UDP-glucose 4-epimerase	M	8611	11554	20165
COG1866	Phosphoenolpyruvate carboxykinase, ATP-dependent	C	6120	6879	12999
COG0075	Archaeal aspartate aminotransferase or a related aminotransferase, includes purine catabolism protein PucG	E;F	2447	3454	5902
COG2836	Uncharacterized membrane protein, DsbD domain	S	16	0	16
COG0293	23S rRNA U2552 (ribose-2'-O)-methylase RlmE/FtsJ	J	31	326	357
COG4670	Acyl CoA:acetate/3-ketoacid CoA transferase	I	381	1088	1469
COG1069	Ribulose kinase	G	704	925	1628
COG0421	Spermidine synthase (polyamine aminopropyltransferase)	E	2817	4342	7159
COG1913	Predicted Zn-dependent protease	R	37	122	160
COG4804	Predicted nuclease of restriction endonuclease-like (RecB) superfamily, DUF1016 family	R	11255	16139	27394
COG4213	ABC-type xylose transport system, periplasmic component	G	1204	1643	2847
COG1165	2-succinyl-5-enolpyruvyl-6-hydroxy-3-cyclohexene-1-carboxylate synthase	H	3307	3745	7052

COG1062	Zn-dependent alcohol/formaldehyde dehydrogenase	C	87	213	301
COG3672	Predicted transglutaminase-like protein	O	30	49	79
COG3124	Acyl carrier protein phosphodiesterase	I	22	37	59
COG3837	Uncharacterized conserved protein, cupin superfamily	S	199	229	428
COG2066	Glutaminase	E	587	1372	1958
COG0863	DNA modification methylase	L	19614	25784	45398
COG1488	Nicotinic acid phosphoribosyltransferase	H	6310	10694	17005
COG0220	tRNA G46 N7-methylase TrmB	J	7171	8474	15646
COG4299	Predicted acyltransferase, DUF1624 domain	R	1406	2034	3440
COG0473	Isocitrate/isopropylmalate dehydrogenase	C;E	5315	7745	13060
COG5395	Uncharacterized membrane protein	S	1	2	2
COG0674	Pyruvate:ferredoxin oxidoreductase or related 2-oxoacid:ferredoxin oxidoreductase, alpha subunit	C	21865	29246	51111
COG1929	Glycerate kinase	G	1824	3179	5002
COG2316	Predicted hydrolase, HD superfamily	R	1339	2183	3522
COG2006	Uncharacterized conserved protein, DUF362 family	S	1834	2850	4684
COG1200	RecG-like helicase	L	14303	17802	32105
COG3090	TRAP-type C4-dicarboxylate transport system, small permease component YiaM	G	1383	4063	5446
COG5847	Stage VI sporulation protein SpoVIF, required for heat resistance	D	86	103	189
COG4866	Uncharacterized protein, contains DUF2156 domain	S	3801	4788	8590
COG5484	Uncharacterized conserved protein YjcR, contains N-terminal HTH domain	S	686	1271	1957
COG0477	MFS family permease, includes anhydromuropeptide permease AmpG	G;E;P;R	769	1514	2283

COG4135	ABC-type uncharacterized transport system YnjBCD, permease component	R	0	5	5
COG1989	Prepilin signal peptidase PulO (type II secretory pathway) or related peptidase	N;U	5265	6578	11843
COG0066	3-isopropylmalate dehydratase small subunit	E	2381	3993	6374
COG2141	Flavin-dependent oxidoreductase, luciferase family (includes alkanesulfonate monooxygenase SsuD and methylene tetrahydromethanopterin reductase)	H;R	15	160	175
COG1211	2-C-methyl-D-erythritol 4-phosphate cytidylyltransferase	I	9952	11248	21200
COG2125	Ribosomal protein S6E (S10)	J	8	7	14
COG0529	Adenylylsulfate kinase or related kinase	P	1553	1836	3389
COG5831	Stage IV sporulation protein SpoIVA, required for spore cortex formation and coat assembly	D	6868	8768	15636
COG1939	23S rRNA maturation mini-RNase III	J	3056	3877	6933
COG5331	Uncharacterized conserved protein	S	4	2	6
COG4857	5-Methylthioribose/5-deoxyribose kinase, methionine salvage pathway	E	112	92	204
COG2905	Signal-transduction protein containing cAMP-binding, CBS, and nucleotidyltransferase domains	T	1097	1777	2874
COG1708	Predicted nucleotidyltransferase, MJ0604 family	R	1091	1024	2114
COG2363	Uncharacterized membrane protein YgdD, TMEM256/DUF423 family	S	1	99	100
COG0543	NAD(P)H-flavin reductase	H;C	13216	15716	28932
COG0689	Ribonuclease PH	J	483	1441	1924
COG0671	Membrane-associated phospholipid phosphatase	I	7921	9299	17220

COG1597	Phosphatidylglycerol kinase, diacylglycerol kinase family	I;R	13461	15966	29427
COG5460	Uncharacterized conserved protein, DUF2164 family	S	21	74	95
COG1129	ABC-type sugar transport system, ATPase component	G	7692	16105	23797
COG0290	Translation initiation factor IF-3	J	5744	6938	12682
COG0586	Membrane integrity protein DedA, putative transporter, DedA/Tvp38 family	M	1584	2328	3912
COG5942	CRISPR-Cas system type I-E effector complex large subunit Cas8e	V	191	252	442
COG1272	Predicted membrane channel-forming protein YqfA, hemolysin III family	U	3687	4934	8621
COG4663	TRAP-type mannitol/chloroaromatic compound transport system, periplasmic component	Q	26	101	127
COG0519	GMP synthase, PP-ATPase domain/subunit	F	793	953	1746
COG0737	2',3'-cyclic-nucleotide 2'-phosphodiesterase/5'- or 3'-nucleotidase, 5'-nucleotidase family	F;V	7936	10574	18510
COG4862	Negative regulator MecA of genetic competence, sporulation and motility	K;T;N	2574	3397	5971
COG2193	Bacterioferritin (cytochrome b1)	P	473	393	866
COG0145	N-methylhydantoinase A/oxoprolinase/acetone carboxylase, beta subunit	E;Q	492	970	1462
COG0602	Organic radical activating enzyme NrdG/QueE	H	8308	10035	18344
COG3497	Phage tail sheath protein FI	X	4857	4668	9526
COG4242	Cyanophycinase and related exopeptidases	Q;R	136	219	355

COG2915	Regulator of phage lambda lysogenization HflD, binds to CII and stimulates its degradation	X;T	0	1	1
COG3548	Uncharacterized membrane protein	S	24	159	183
COG3956	Uncharacterized conserved protein YabN, contains tetrapyrrole methylase and MazG-like pyrophosphatase domain	R	5906	7301	13206
COG2247	Putative cell wall-binding domain (amidase enhancer), LytB superfamily	M	8280	8554	16834
COG1279	Arginine exporter protein ArgO	E	122	524	645
COG0419	DNA repair exonuclease SbcCD ATPase subunit	L	16147	18131	34279
COG0299	Folate-dependent phosphoribosylglycinamide	F	5008	6421	11429
COG1688	CRISPR/Cas system-associated protein Cas5, RAMP superfamily	V	268	363	630
COG0624	Acetylornithine deacetylase/Succinyl-diaminopimelate desuccinylase or related deacylase	E	8861	18185	27045
COG0717	dCTP deaminase	F	1046	954	2001
COG4626	Phage terminase-like protein, large subunit, contains N-terminal HTH domain	X	7689	9608	17297
COG5561	Predicted metal-binding protein, contains CGGC domain	S	37	102	139
COG3950	Predicted ATP-binding protein involved in virulence	R	3095	3098	6193
COG0751	Glycyl-tRNA synthetase, beta subunit	J	267	1092	1359
COG0497	DNA repair ATPase RecN	L	10790	14367	25157
COG2183	Transcriptional accessory protein Tex/SPT6	K	9402	11883	21285
COG2105	Predicted gamma-glutamylamine cyclotransferase YtfP, GGCT/AIG2-like family	E	2832	2686	5518

COG3829	RocR-type transcriptional regulator, contains PAS, AAA-type ATPase, and DNA-binding Fis domains	K;T	4396	10335	14731
COG1943	REP element-mobilizing transposase RayT	X	11437	17632	29069
COG0201	Preprotein translocase subunit SecY	U	11951	13727	25677
COG3799	Methylaspartate ammonia-lyase	E	187	435	622
COG0188	DNA gyrase/topoisomerase IV, subunit A	L	27524	35804	63329
COG4303	Ethanolamine ammonia-lyase, large subunit	E	503	1552	2055
COG5611	Predicted nucleic-acid-binding protein, contains PIN domain	R	344	255	599
COG3375	Predicted acetyltransferase, GNAT superfamily	R	7	22	29
COG3613	Nucleoside 2-deoxyribosyltransferase	F	291	583	874
COG0244	Ribosomal protein L10	J	6204	7601	13806
COG2249	Putative NADPH-quinone reductase (modulator of drug activity B)	R	700	1117	1817
COG3633	Na+/serine and Na+/threonine symporter SstT	E	266	690	957
COG3280	Maltooligosyltrehalose synthase	G	0	3	3
COG0429	Predicted hydrolase of the alpha/beta-hydrolase fold	R	34	268	303
COG3850	Signal transduction histidine kinase NarQ, nitrate/nitrite-specific	T	390	603	993
COG5546	Uncharacterized membrane protein	S	223	175	398
COG0357	16S rRNA G527 N7-methylase RsmG (former glucose-inhibited division protein B)	J	8427	9713	18140
COG1534	RNA-binding protein YhbY	J	1822	2163	3985
COG0214	Pyridoxal 5'-phosphate synthase subunit PdxS	H	551	942	1493
COG2091	Phosphopantetheinyl transferase	H	5758	5581	11339
COG4607	ABC-type enterochelin transport system, periplasmic component	P	81	167	248

COG4310	Uncharacterized conserved protein, cotains an aminopeptidase-like domain	R	271	431	701
COG1078	HD superfamily phosphohydrolase	R	3233	3651	6884
COG1187	Pseudouridylate synthase RsuA, specific for 16S rRNA U516 and 23S rRNA U2605	J	11058	14821	25879
COG0730	Sulfite exporter TauE/SafE/YfcA and related permeases, UPF0721 family	P	8708	11696	20404
COG0034	Glutamine phosphoribosylpyrophosphate amidotransferase	F	13116	17126	30243
COG1983	Phage shock protein PspC (stress-responsive transcriptional regulator)	K;T	3608	3104	6712
COG0662	Mannose-6-phosphate isomerase, cupin superfamily	G	4385	5618	10003
COG0572	Uridine kinase	F	11428	14053	25481
COG2401	ABC-type ATPase fused to a predicted acetyltransferase domain	R	86	48	134
COG1456	CO dehydrogenase/acetyl-CoA synthase gamma subunit (corrinoid Fe-S protein)	C	1032	1471	2503
COG1827	Transcriptional regulator of NAD metabolism, contains HTH and 3H domains	K;H	546	969	1515
COG4109	Predicted transcriptional regulator containing CBS domains	K	435	875	1310
COG1109	Phosphomannomutase	G	24314	32550	56864
COG2513	2-Methylisocitrate lyase and related enzymes, PEP mutase family	G	1791	2094	3885
COG2863	Cytochrome c553	C	7	664	670
COG2222	Fructoselysine-6-P-deglycase FrlB or related protein, duplicated sugar isomerase (SIS) domain	M	933	2394	3327
COG3636	Uncharacterized Zn-binding protein, DUF971 domain	X	46	269	315
COG4123	tRNA1(Val) A37 N6-methylase TrmN6	J	9169	11360	20529

COG5443	Flagellar biosynthesis regulator FlbT	N	1	7	7
COG2221	Dissimilatory sulfite reductase (desulfoviridin), alpha and beta subunits	P	3561	4880	8441
COG1281	Redox-regulated molecular chaperone, HSP33 family	O	5545	7463	13007
COG0719	Fe-S cluster assembly scaffold protein SufB	O	10432	12853	23285
COG4669	Type III secretory pathway, lipoprotein EscJ	U	5	7	12
COG1898	dTDP-4-dehydrorhamnose 3,5-epimerase or related enzyme	M	6467	7410	13876
COG0759	Membrane-anchored protein YidD, putatitve component of membrane protein insertase Oxa1/YidC/SpoIIIJ	M	1956	1981	3937
COG3725	Membrane protein AmpE required for beta-lactamase induction	V	0	4	4
COG3600	Uncharacterized phage-associated protein, contains DUF4065 domain	X	3397	3578	6975
COG2978	p-Aminobenzoyl-glutamate transporter	H	678	1470	2148
COG1336	CRISPR-Cas system type III CMR-effector complex subunit Cmr4, RAMP superfamily Cas7 group	V	84	155	239
COG2130	NADPH-dependent curcumin reductase CurA	Q;R	0	1	1
COG1274	Phosphoenolpyruvate carboxykinase, GTP-dependent	C	1832	3284	5116
COG3499	Phage protein U	X	262	330	591
COG3120	Macrodomain Ter protein organizer, MatP/YcbG family	L	0	1	1
COG1224	DNA helicase TIP49, TBP-interacting protein	K	1	0	1
COG1132	ABC-type multidrug transport system, ATPase and permease component	V	133329	147368	280696

COG4235	Cytochrome c-type biogenesis protein CcmH/NrfG	C;O	899	1395	2294
COG1568	Aminopropyltransferase BpsA, N(4)-bis(aminopropyl)spermidine biosynthesis	Q	14	9	23
COG1923	sRNA-binding regulator protein Hfq	T	372	431	804
COG2089	Sialic acid synthase SpsE, contains C-terminal SAF domain	M	5296	6854	12150
COG4211	ABC-type glucose/galactose transport system, permease component	G	1009	1659	2668
COG1203	CRISPR-Cas type I system-associated endonuclease/helicase Cas3	V	4876	5705	10581
COG0789	DNA-binding transcriptional regulator, MerR family	K	15528	22298	37826
COG4237	Hydrogenase-4 membrane subunit HyfE	C	580	516	1095
COG2134	CDP-diacylglycerol pyrophosphatase	I	3	6	9
COG4618	ABC-type protease/lipase transport system, ATPase and permease components	U	8	1	9
COG4542	PduX protein involved in propanediol utilization and related proteins	Q	1	78	79
COG0754	Glutathionylspermidine synthase, CHAP domain	E	42	68	111
COG1008	NADH:ubiquinone oxidoreductase subunit 4 (chain M)	C	1887	2238	4124
COG3747	Phage terminase, small subunit	X	823	1064	1887
COG2114	Adenylate cyclase, class 3	T	2682	2709	5391
COG1726	Na+-transporting NADH:ubiquinone oxidoreductase, subunit NqrA	C	2592	2478	5069
COG1960	Acyl-CoA dehydrogenase related to the alkylation response protein AidB	I	5126	7895	13022
COG3871	General stress protein 26 (function	S	3059	2749	5808
COG0785	Cytochrome c biogenesis protein CcdA	C;O	261	479	739

COG3106	Ras-like GTP-binding stress-induced protein YcjX, DUF463 family	T	0	1	1
COG2407	L-fucose isomerase or related protein	G	7227	10164	17391
COG3276	Selenocysteine-specific translation elongation factor SelB	J	173	444	617
COG1828	Phosphoribosylformylglycinamidine (FGAM) synthase, PurS subunit	F	3	20	24
COG1838	Tartrate dehydratase beta subunit/Fumarate hydratase class I, C-terminal domain	C	5556	6820	12375
COG2932	Phage repressor protein C, contains Cro/C1-type HTH and peptisase s24 domains	X	5523	6672	12195
COG3279	DNA-binding response regulator, LytR/AlgR family	K;T	52709	46706	99415
COG3840	ABC-type thiamine transport system, ATPase component ThiQ	H	304	59	364
COG0026	Phosphoribosylaminoimidazole carboxylase (NCAIR synthetase)	F	1401	1481	2882
COG4292	Low temperature requirement protein LtrA (function unknown)	S	4	101	105
COG4993	Glucose dehydrogenase, PQQ-dependent	G	4	23	28
COG1651	Protein thiol-disulfide isomerase DsbC	O	267	1066	1334
COG0520	Selenocysteine lyase/Cysteine desulfurase	E	9736	12688	22424
COG0547	Anthranilate phosphoribosyltransferase, glycosyltransferase domain	E	3541	3578	7118
COG2808	Predicted FMN-binding regulatory protein PaiB	T	1	1	2
COG3921	Uncharacterized conserved protein, contains Extensin-like_C domain	S	44	300	344
COG3437	Response regulator c-di-GMP phosphodiesterase, RpfG family, contains REC and HD-GYP domains	T	10349	9910	20260
COG2510	Riboflavin transporter RibN, EamA domain	H	5631	6032	11663

COG3181	Tripartite-type tricarboxylate transporter, extracytoplasmic receptor component TctC	C	951	3263	4213
COG2062	Phosphohistidine phosphatase SixA	T	25	11	37
COG0478	RIO-like serine/threonine protein kinase fused to N-terminal HTH domain	T	792	877	1668
COG2250	HEPN domain protein, predicted toxin of MNT-HEPN system	V	1294	1243	2537
COG0755	ABC-type transport system involved in cytochrome c biogenesis, permease component	O	1435	2722	4158
COG5939	CRISPR-Cas system type I-B effector complex large subunit Cas8b2	V	91	293	384
COG0454	N-acetyltransferase, GNAT superfamily (includes histone acetyltransferase HPA2)	K;R	934	810	1744
COG0329	4-hydroxy-tetrahydrodipicolinate synthase/N-acetylneuraminate lyase	E;M	11800	17037	28837
COG4905	Uncharacterized membrane protein	S	11653	14865	26518
COG1848	VapC family ribonuclease, toxin component of the VapBC toxin-antitoxin module, contains PIN domain	V	2316	2402	4719
COG5015	Pyridoxamine 5'-phosphate oxidase (PNPOx-like) family protein	R	521	772	1294
COG2984	ABC-type uncharacterized transport system, periplasmic component	R	5841	7073	12914
COG3700	Acid phosphatase, class B	P;R	0	4	4
COG1580	Flagellar basal body-associated protein FliL	N	46	98	144
COG0377	NADH:ubiquinone oxidoreductase 20 kD subunit (chain B) or related Fe-S oxidoreductase	C	729	918	1647
COG1079	ABC-type guanosine uptake system NupNOPQ, permease subunit NupQ	F	3913	5782	9695

COG4783	Outer membrane protein chaperone/metalloprotease BepA/YfgC, contains M48 and TPR domains	M;O	6797	11643	18439
COG0155	Sulfite reductase, beta subunit	P	677	932	1609
COG0104	Adenylosuccinate synthase	F	9124	12024	21148
COG4147	Na+(or H+)/acetate symporter ActP	C	26	7	33
COG1740	Ni,Fe-hydrogenase I small subunit	C	138	890	1028
COG4445	tRNA isopentenyl-2-thiomethyl-A-37 hydroxylase MiaE (synthesis of 2-methylthio-cis-ribozeatin)	J	0	2	2
COG0195	Transcription antitermination factor NusA, contains S1 and KH domains	K	9238	12499	21738
COG0423	Glycyl-tRNA synthetase, class II	J	7954	10521	18475
COG0216	Protein chain release factor RF1	J	8661	10868	19529
COG4285	Uncharacterized conserved protein, conains N-terminal glutamine amidotransferase (GATase1)-like domain	R	27	70	97
COG0286	Type I restriction-modification system, DNA methylase subunit	V	70673	79971	150643
COG5845	Stage V sporulation protein SpoVT, regulator of stationary/sporulation gene expression	D;K	3366	4711	8077
COG2365	Protein tyrosine/serine phosphatase Oca4	T	1447	2212	3660
COG2891	Cell shape-determining protein MreD	M	996	1341	2338
COG5522	Uncharacterized membrane protein YwaF	S	561	1024	1586
COG2358	TRAP-type uncharacterized transport system, periplasmic component	R	921	2133	3053
COG3887	Cyclic di-AMP phosphodiesterase GdpP, contains GGDEF-like and DHH domains	T	7699	9734	17433
COG4172	ABC-type microcin C transport system, duplicated ATPase component YejF	Q	15	388	403

COG5011	Uncharacterized conserved protein, DUF2344 family	S	3682	3904	7585
COG4789	Type III secretory pathway, component EscV	U	3	19	21
COG1782	Predicted metal-dependent RNase, contains metallo-beta-lactamase and KH domains	R	543	1463	2005
COG0563	Adenylate kinase or related kinase	F	10231	12087	22318
COG3533	Beta-L-arabinofuranosidase, GH127 family	G	6187	7320	13507
COG5323	Phage large terminase packaging protein	X	7	2	9
COG1213	Choline kinase	I	2542	3601	6144
COG3872	Uncharacterized conserved protein YqhQ, DUF1385 family	S	5871	6306	12177
COG5423	Predicted metal-binding protein	S	1816	2350	4166
COG5725	Photosystem II reaction center protein K, PsbK	C	8	1	9
COG4589	Predicted CDP-diglyceride synthetase/phosphatidate cytidylyltransferase	R	284	847	1131
COG3934	Endo-1,4-beta-mannosidase	G	782	956	1738
COG0008	Glutamyl- or glutaminyl-tRNA synthetase	J	20858	28472	49330
COG0038	H+/Cl- antiporter ClcA	P	6230	8014	14244
COG1261	Flagellar basal body P-ring formation protein FlgA	N	155	233	388
COG0463	Glycosyltransferase involved in cell wall bisynthesis	M	126406	151768	278174
COG1836	Cytidylyltransferase family enzyme	R	17	100	117
COG1629	Outer membrane receptor protein, Fe transport	P	53207	66971	120179
COG1579	Predicted nucleic acid-binding protein DR0291, contains C4-type Zn-ribbon domain	R	2717	3192	5909
COG3394	Chitooligosaccharide deacetylase ChbG, YdjC/CelG family	G	816	1116	1932

COG0845	Multidrug efflux pump subunit AcrA (membrane-fusion protein)	M;V	25302	33652	58954
COG3552	Uncharacterized protein CoxE, contains von Willebrand factor type A (vWA) domain	S	237	513	750
COG4110	Tellurium resistance protein TerA/TerD	V	115	105	220
COG0315	Molybdenum cofactor biosynthesis enzyme MoaC	H	548	1144	1692
COG1449	Alpha-amylase/alpha-mannosidase, GH57 family	G	3181	3776	6957
COG5590	Ubiquinone biosynthesis protein COQ9	H	0	2	2
COG3817	Uncharacterized membrane protein	S	36	35	70
COG1889	Fibrillarin-like rRNA 2'-O-methylase NOP1	J	0	0	0
COG5652	VanZ-like family protein (function	S	2588	3186	5774
COG0203	Ribosomal protein L17	J	4997	6133	11131
COG2423	Ornithine cyclodeaminase/archaeal alanine dehydrogenase, mu-crystallin family	E	848	1428	2276
COG1820	N-acetylglucosamine-6-phosphate deacetylase	G	4357	8528	12885
COG3760	Predicted aminoacyl-tRNA deacylase, YbaK-like aminoacyl-tRNA editing domain	R	4016	5137	9153
COG0124	Histidyl-tRNA synthetase	J	10838	13887	24725
COG3468	Autotransporter adhesin AidA	M;U	1389	5723	7112
COG2033	Desulfoferrodoxin, superoxide reductase-like (SORL) domain	C	718	1280	1998
COG2870	ADP-heptose synthase, bifunctional sugar kinase/adenylyltransferase	M	1452	2651	4103
COG3973	DNA helicase IV	L	3133	4848	7981
COG0812	UDP-N-acetylenolpyruvoylglucosamine reductase	M	8996	10828	19824
COG4737	Uncharacterized conserved protein	S	263	250	513
COG1283	Na+/phosphate symporter	P	14046	16472	30518

COG1178	ABC-type Fe3+ transport system, permease component	P	1668	3846	5513
COG1878	Kynurenine formamidase	E	779	1972	2751
COG0750	Membrane-associated protease RseP, regulator of RpoE activity	O;K	9345	12261	21607
COG0498	Threonine synthase	E	8990	11542	20532
COG1156	Archaeal/vacuolar-type H+-ATPase subunit B/Vma2	C	3402	4329	7732
COG1083	CMP-N-acetylneuraminic acid synthetase, NeuA/PseF family	M	5871	6395	12266
COG0310	ABC-type Co2+ transport system, permease component	P	1383	2320	3703
COG0237	Dephospho-CoA kinase	H	6211	7579	13790
COG3184	Uncharacterized conserved protein, contains DUF2059 domain	S	65	64	130
COG3016	Putative heme-binding protein PhuW	R	2	31	33
COG2879	Uncharacterized short protein YbdD, DUF466 family	S	3	0	3
COG4597	ABC-type amino acid transport system, permease component	E	0	205	205
COG0162	Tyrosyl-tRNA synthetase	J	9321	12626	21947
COG4124	Beta-mannanase	G	7841	6025	13866
COG1952	Preprotein translocase subunit SecB	U	144	502	647
COG0619	ECF-type transporter transmembrane protein EcfT	H	10841	13371	24212
COG2022	Thiazole synthase ThiGH, ThiG subunit (thiamin biosynthesis)	H	1766	2394	4160
COG0311	Pyridoxal 5'-phosphate synthase subunit PdxT (glutamine amidotransferase)	H	726	937	1662
COG0317	(p)ppGpp synthase/hydrolase, HD superfamily	T;K	18856	24522	43379
COG2931	Ca2+-binding protein, RTX toxin-related	Q	7060	5974	13034

COG1179	tRNA A37 threonylcarbamoyladenosine dehydratase	J	8297	10406	18703
COG1713	Diadenosine tetraphosphatase YqeK or a related HD superfamily phosphohydrolase	T;R	3315	3630	6945
COG2142	Succinate dehydrogenase, hydrophobic anchor subunit	C	0	2	2
COG5610	Predicted sugar hydrolase, contains GT1 and HAD domains	R	2347	3483	5830
COG0175	3'-phosphoadenosine 5'-phosphosulfate sulfotransferase (PAPS reductase)/FAD synthetase or related enzyme	E;H	11870	14158	26028
COG0343	Queuine/archaeosine tRNA-ribosyltransferase	J	8843	11484	20328
COG2026	mRNA-degrading endonuclease RelE, toxin component of the RelBE toxin-antitoxin system	V	1599	1579	3178
COG1924	Activator of 2-hydroxyglutaryl-CoA dehydratase (HSP70-class ATPase domain)	I	11199	15849	27048
COG3041	mRNA-degrading endonuclease YafQ (mRNA interferase), toxin component of the YafQ-DinJ toxin-antitoxin module	J;V	1639	1988	3627
COG3641	Membrane regulatory protein PfoR, PTS_EIIC_2 domain (does not regulate perfringolysin expression)	T	1646	3423	5069
COG5719	Photosystem II reaction center D2, PsbD	C	22	11	33
COG2342	Endo alpha-1,4 polygalactosaminidase, GH114 family (was erroneously annotated as Cys-tRNA synthetase)	G	23	26	49
COG0180	Tryptophanyl-tRNA synthetase	J	9007	11720	20727
COG0837	Glucokinase	G	34	114	149
COG2603	tRNA 2-selenouridine synthase SelU, contains rhodanese domain	J	18	10	29

COG0778	Nitroreductase	C	14065	19894	33959
COG0710	3-dehydroquinate dehydratase	E	813	1653	2466
COG4499	ESX protein secretion system component YukC	U	448	251	698
COG2192	Predicted carbamoyl transferase, NodU family	R	894	775	1669
COG2353	Polyisoprenoid-binding periplasmic protein YceI	R	37	1	38
COG5534	Plasmid replication initiator protein	X	6	9	15
COG0226	ABC-type phosphate transport system, periplasmic component	P	9019	10626	19645
COG1177	ABC-type spermidine/putrescine transport system, permease component II	E	3811	5319	9130
COG1126	ABC-type polar amino acid transport system, ATPase component	E	7859	11059	18919
COG5914	Spore germination protein YpeB, HAD superfamily, activates cortex-lytic enzyme SleB	D	2100	3339	5439
COG0146	N-methylhydantoinase B/oxoprolinase/acetone carboxylase, alpha subunit	E;Q	32	136	169
COG5459	Ribosomal protein RSM22 (predicted mitochondrial rRNA methylase)	J	1060	423	1483
COG0428	Zinc transporter ZupT	P	7058	10112	17170
COG1172	Ribose/xylose/arabinose/galactoside ABC-type transport system, permease component	G	8319	15493	23812
COG0640	DNA-binding transcriptional regulator, ArsR family	K	4235	6337	10572
COG1666	Cyclic di-GMP-binding protein YajQ, UPF0234 family	T	40	176	215

COG4550	Cell fate regulator YmcA, YheA/YmcA/DUF963 family (controls sporulation, competence, biofilm development)	T	0	2	2
COG1727	Ribosomal protein L18E	J	2	2	4
COG2732	Barstar, RNAse (barnase) inhibitor	K	596	717	1313
COG0791	Cell wall-associated hydrolase, NlpC_P60 family	M	22635	31257	53892
COG1847	Predicted RNA-binding protein Jag (SpoIIIJ-associated), conains KH and R3H domains	R	7341	8446	15788
COG4193	Beta- N-acetylglucosaminidase	G	3684	5191	8875
COG2888	Predicted RNA-binding protein involved in translation, contains Zn-ribbon domain, DUF1610 family	R	608	979	1587
COG4525	ABC-type taurine transport system, ATPase component	P	198	341	539
COG2145	Hydroxyethylthiazole kinase, sugar kinase family	H	1837	3244	5081
COG0387	Cation (Ca2+/Na+/K+)/H+ antiporter ChaA	P	0	3	3
COG0115	Branched-chain amino acid aminotransferase/4-amino-4-deoxychorismate lyase	E;H	9064	11705	20769
COG1907	Beta-ribofuranosylaminobenzene 5'-phosphate synthase (methanopterin biosynthesis)	H	39	80	119
COG0638	20S proteasome, alpha and beta subunits	O	28	108	136
COG2053	Ribosomal protein S28E/S33	J	1	0	2
COG1812	Archaeal S-adenosylmethionine synthetase	H	25	71	97
COG3880	Protein-arginine kinase activator protein McsA	O	1312	1998	3310
COG2860	Uncharacterized membrane protein YeiH	S	1735	2897	4632

COG1687	Branched-chain amino acid transport protein AzlD	E	807	1125	1933
COG1039	Ribonuclease HIII	L	743	1306	2049
COG2085	Predicted dinucleotide-binding enzyme	R	5	22	27
COG1384	Lysyl-tRNA synthetase, class I	J	210	688	898
COG0160	Acetylornithine aminotransferase/4-aminobutyrate aminotransferase	E	491	1331	1822
COG1658	5S rRNA maturation ribonuclease M5, contains TOPRIM domain	J	2884	2456	5340
COG0222	Ribosomal protein L7/L12	J	3214	3852	7066
COG1366	Anti-anti-sigma regulatory factor (antagonist of anti-sigma factor)	T	1325	1564	2889
COG3626	Alpha-D-ribose 1-methylphosphonate 5-triphosphate synthase subunit PhnI	P	114	41	155
COG0609	ABC-type Fe3+-siderophore transport system, permease component	P	7575	10088	17663
COG4335	3-methylpurine DNA glycosylase AlkC	L	434	279	713
COG4297	Uncharacterized conserved protein YjlB, cupin superfamily	S	2	1	3
COG5485	Predicted ester cyclase	R	13	237	250
COG4839	Cell division protein FtsL	D	551	658	1209
COG3263	NhaP-type Na+/H+ and K+/H+ antiporter with C-terminal TrkAC and CorC domains	C;P	3252	3692	6944
COG5280	Phage-related minor tail protein	X	8422	9554	17976
COG2609	Pyruvate dehydrogenase complex, dehydrogenase (E1) component	C	0	5	5
COG0379	Quinolinate synthase	H	5116	5260	10376
COG0687	Spermidine/putrescine-binding periplasmic protein	E	5206	7439	12645
COG1928	Dolichyl-phosphate-mannose--protein O-mannosyl transferase	O	2102	3044	5146

COG0076	Glutamate or tyrosine decarboxylase or a related PLP-dependent protein	E	879	1353	2232
COG4859	Uncharacterized protein, contains DUF2185 domain	S	3084	2564	5648
COG5305	Uncharacterized membrane protein PF0508, contains N-terminal glycosyltransferase domain of PMT family	R	3762	4336	8098
COG0166	Glucose-6-phosphate isomerase	G	9170	12926	22095
COG0603	7-cyano-7-deazaguanine synthase (queuosine biosynthesis)	J	2344	3233	5577
COG0471	Di- and tricarboxylate antiporter	G	2618	7220	9838
COG2171	Tetrahydrodipicolinate N-succinyltransferase	E	2307	2214	4521
COG3445	Autonomous glycyl radical cofactor GrcA	H	0	2	2
COG3349	Uncharacterized protein, contains NAD-binding domain and a Fe-S cluster	R	24	55	79
COG0326	Molecular chaperone, HSP90 family	O	16018	20403	36422
COG2606	Cys-tRNA(Pro) deacylase, prolyl-tRNA editing enzyme YbaK/EbsC	J	5499	6432	11931
COG3580	Predicted nucleotide-binding protein, sugar kinase/HSP70/actin superfamily	R	344	720	1063
COG0621	tRNA A37 methylthiotransferase MiaB	J	31504	39217	70720
COG4947	Esterase/lipase superfamily enzyme	R	916	1565	2481
COG3139	Uncharacterized conserved protein YeaC, DUF1315 family	S	0	0	0
COG0591	Na+/proline symporter	E	9554	13480	23035
COG0752	Glycyl-tRNA synthetase, alpha subunit	J	119	573	692
COG0271	DNA-binding global transcriptional regulator BolA, affects cell shape, cell division and biofilm formation	K;T	0	44	44
COG4951	Uncharacterized conserved protein	S	31	112	143
COG2933	23S rRNA C2498 (ribose-2'-O)-methylase RlmM	J	10	31	41

COG0664	cAMP-binding domain of CRP or a regulatory subunit of cAMP-dependent protein kinases	T	8284	11616	19900
COG2371	Urease accessory protein UreE	O	1174	1297	2471

Supplementary Table 4. Functional annotations of metagenomics sequencing data against the database of Carbohydrate-Active enZYmes (CAZy).

Family	Family_Description	Western Slopes	Estern Slopes	Total
AA1	Laccase / p-diphenol:oxygen oxidoreductase / ferroxidase (EC 1.10.3.2); ; ferroxidase (EC 1.10.3.-); Laccase-like multicopper oxidase (EC 1.10.3.-)	6	19	24
AA10	AA10 (formerly CBM33) proteins are copper-dependent lytic polysaccharide monooxygenases (LPMOs); some proteins have been shown to act on chitin, others on cellulose; lytic cellulose monooxygenase (C1-hydroxylating) (EC 1.14.99.54); lytic cellulose monooxygenase (C4-dehydrogenating)(EC 1.14.99.56); lytic chitin monooxygenase (EC 1.14.99.53)	0	3	3
AA12	The pyrroloquinoline quinone-dependent oxidoreductase activity was demonstrated for the CC1G_09525 protein of Coprinopsis cinerea.	2	2	4
AA1_2	AA1's subfamily	1	2	3
AA1_3	AA1's subfamily	0	5	5
AA2	manganese peroxidase (EC 1.11.1.13); versatile peroxidase (EC 1.11.1.16); lignin peroxidase (EC 1.11.1.14); peroxidase (EC 1.11.1.-)	1	7	7
AA3	cellobiose dehydrogenase (EC 1.1.99.18); glucose 1-oxidase (EC 1.1.3.4); aryl alcohol oxidase (EC 1.1.3.7); alcohol oxidase (EC 1.1.3.13); pyranose oxidase (EC 1.1.3.10)	5117	7477	12594
AA3_1	AA3's subfamily	671	690	1361
AA3_2	AA3's subfamily	152	225	376
AA3_3	AA3's subfamily	11	11	22
AA3_4	AA3's subfamily	87	125	212
AA4	vanillyl-alcohol oxidase (EC 1.1.3.38)	686	1477	2163

AA5	Oxidase with oxygen as acceptor (EC 1.1.3.-); galactose oxidase (EC 1.1.3.9); glyoxal oxidase (EC 1.2.3.15); alcohol oxidase (EC 1.1.3.13)	635	668	1303
AA5_1	AA5's subfamily	0	0	0
AA5_2	AA5's subfamily	10	13	23
AA6	1,4-benzoquinone reductase (EC. 1.6.5.6)	14170	13906	28075
AA7	glucooligosaccharide oxidase (EC 1.1.3.-); chitooligosaccharide oxidase (EC 1.1.3.-)	301	503	803
AA8	Iron reductase domain	106	141	247
CBM11	Modules of approx. 180-200 residues. The CBM11 of Clotridium thermocellum Cel26A-Cel5E has been shown to bind both beta-1,4-glucan and beta-1,3-1,4-mixed linked glucans.	3	1	5
CBM12	Modules of approx. 40-60 residues. The majority of these modules is found among chitinases where the function is chitin-binding. Distantly related to the CBM5 family.	17	14	31

CBM13	Modules of approx. 150 residues which always appear as a threefold internal repeat. The only apparent exception to this, xylanase II of Actinomadura sp. FC7 (GenBank U08894), is in fact not completely sequenced. These modules were first identified in several plant lectins such as ricin or agglutinin of Ricinus communis which bind galactose residues. The three-dimensional structure of a plant lectin has been determined and displays a pseudo-threefold symmetry in accord with the observed sequence threefold repeat. These modules have since been found in a number of other proteins of various functions including glycoside hydrolases and glycosyltransferases. While in the plant lectins this module binds mannose, binding to xylan has been demonstrated in the Streptomyces lividans xylanase A and arabinofuranosidase B. Binding to GalNAc has been shown for the corresponding module of GalNAc transferase 4. For the other proteins, the binding specificity of these modules has not been established. The pseudo three-fold symmetry of the CBM13 module has now been confirmed in the 3-D structure of the intact, two-domain, xylanase of Streptomyces olivaceoviridis.	2439	1726	4166
CBM15	Binding to xylan and xylooligosaccharides has been demonstrated in the case of Xyn10C of Cellvibrio mixtus.	7	2	9

CBM16	Carbohydrate-binding module 16. Binding to cellulose and glucomannan demonstrated [B. Bae et al (2008) J Biol Chem. 283:12415-25 (PMID: 18025086)]	415	449	864
CBM17	Modules of approx. 200 residues. Binding to amorphous cellulose, cellooligosaccharides and derivatized cellulose has been demonstrated.	3	1	4
CBM2	Modules of approx. 100 residues and which are found in a large number of bacterial enzymes. The cellulose-binding function has been demonstrated in many cases. Several of these modules have been shown to also bind chitin or xylan.	437	341	778
CBM20	The granular starch-binding function has been demonstrated in several cases. Interact strongly with cyclodextrins. Often designated as starch-binding domains (SBD).	169	84	253
CBM21	Modules of approx. 100 residues. The granular starch-binding function has been demonstrated in one case. Sometimes designated as starch-binding domains (SBD).	169	52	222
CBM22	A xylan binding function has been demonstrated in several cases and affinity with mixed beta-1,3/beta-1,4-glucans in one. In several cases a thermostabilizing effect has also been seen.	342	476	818
CBM23	Mannan-binding function demonstrated in one case.	48	44	92
CBM25	Starch-binding function demonstrated in one case.	233	360	593
CBM26	Starch-binding function demonstrated in two cases.	445	167	612

CBM27	Mannan-binding function demonstrated in two cases	60	26	86
CBM28	The module from the endo-1,4-glucanase of Bacillus sp. 1139 binds to non-crystalline cellulose, cellooligosaccharides, and beta-(1,3)(1,4)-glucans	22	50	71
CBM3	Modules of approx. 150 residues found in bacterial enzymes. The cellulose-binding function has been demonstrated in many cases. In one instance binding to chitin has been reported.	23	48	71
CBM30	Binding to cellulose has been demonstrated for the N-terminal module of Fibrobacter succinogenes CelF.	21	23	44
CBM32	Binding to galactose and lactose has been demonstrated for the module of Micromonospora viridifaciens sialidase (PMID: 16239725). Binding to polygalacturonic acid has been shown for a Yersinia member (PMID: 17292916). Binding to LacNAc (beta-D-galactosyl-1,4-beta-D-N-acetylglucosamine) has been shown for an N-acetylglucosaminidase from Clostridium perfingens (PMID: 16990278).	3216	3087	6303
CBM34	Modules of approx. 120 residues. Granular starch-binding function has been demonstrated in the case of Thermoactinomyces vulgaris R-47 -amylase 1 (TVAI).	369	381	750

CBM35	Modules of approx. 130 residues. A module that is conserved in three Cellvibrio xylan-degrading enzymes binds to xylan and the interaction is calcium dependent, while a module from a Cellvibrio mannanase binds to decorated soluble mannans and mannooligosaccharides. A module in a	849	1192	2042
CBM36	Modules of approx. 120-130 residues displaying structural similarities to CBM6 modules. The only CBM36 currently characterised, that from Paenbacillus polymyxa xylanase 43A, shows calcium-dependent binding of xylans and xylooligosaccharides. X-ray crystallography shows that there is a direct interaction between calcium and ligand.	9	9	18
CBM37	Modules of approx. 100 residues, conserved in numerous R. albus polysaccharide-degrading enzymes and other proteins from this bacterium. Several members of CBM37 have been shown to exhibit rather broad binding specificity to xylan, chitin, microcrystalline and phosphoric-acid swollen cellulose, as well as more heterogeneous substrates, such as alfalfa cell walls, banana stem and wheat straw.	995	758	1754
CBM38	The inulin-binding function has been demonstrated in the case of the cycloinulo-oligosaccharide fructanotransferase from Paenibacillus macerans (Bacillus macerans) by Lee et al. (2004) FEMS Microbiol Lett 234:105-10. (PMID:15109727).	173	116	289

CBM39	Modules generally found at the N-terminus of a GH16 module (itself frequently lacking a catalytic machinery) and more seldomly in isolation. The beta-1,3-glucan binding function has been demonstrated, along with binding to lipopolysaccharide and lipoteichoic acid.	1	1	1
CBM4	Modules of approx. 150 residues found in bacterial enzymes. Binding of these modules has been demonstrated with xylan, beta-1,3-glucan, beta-1,3-1,4-glucan, beta-1,6-glucan and amorphous cellulose but not with crystalline cellulose.	308	288	596
CBM40	Modules of approx. 200 residues, found at the N-terminus of GH33 sialidases. Can also be found inserted in the beta-propeller of GH33 sialidases. The sialic acid binding function has been demonstrated for the N-terminal CBM40 of Vibrio cholerae sialidase (Moustafa et al. (2004) J Biol Chem 279:40819-26) (PMID: 15226294).	502	1436	1938
CBM41	Modules of approx. 100 residues found in primarily in bacterial pullulanases. The N-terminal module from Thermotoga maritima Pul13 has been shown to bind to the alpha-glucans amylose, amylopectin, pullulan, and oligosaccharide fragments derived from these polysaccharides (Lammerts van Bueren et al. (2004) Biochemistry 43:15633-42) (PMID: 15581376).	105	91	195

CBM42	Modules of approx. 160 residues found mostly at the C-terminus of GH54 catalytic domains. Binding to arabinofuranose (present in arabinoxylan) has been demonstrated.	43	16	59
CBM43	Modules of approx. 90-100 residues found at the C-terminus of GH17 or GH72 enzymatic modules and also sometimes isolated. CBM43 modules sometimes carry a C-terminal membrane anchor. The beta-1,3-glucan binding function has been demonstrated with the olive pollen protein Ole e 10.	1	1	2
CBM44	The C-terminal CBM44 module of the Clostridium thermocellum enzyme has been demonstrated to bind equally well cellulose and xyloglucan	5	10	15
CBM46	Modules of approx. 100 residues, found at the C-terminus of several GH5 cellulases. Cellulose-binding function demonstrated in one case.	24	20	43
CBM47	Modules of approx 150 residues. Fucose-binding activity demonstrated	13	12	26
CBM48	Modules of approx. 100 residues with glycogen-binding function, appended to GH13 modules. Also found in the beta subunit (glycogen-binding) of AMP-activated protein kinases (AMPK)	1208	1369	2576
CBM5	Modules of approx. 60 residues found in bacterial enzymes. Chitin-binding described in several cases. Distantly related to the CBM12 family.	6	5	11

CBM50	Modules of approx. 50 residues found attached to various enzymes from families GH18, GH19, GH23, GH24, GH25 and GH73, i.e. enzymes cleaving either chitin or peptidoglycan. Binding to chitopentaose demonstrated in the case of Pteris ryukyuensis chitinase A [Ohnuma T et al. (2008) J. Biol. Chem. 283:5178-87 (PMID: 18083709)]. CBM50 modules are also found in a multitude of other enzymes targeting the petidoglycan such as peptidases and amidases. These enzymes are not reported in the list below.	1729	1937	3666
CBM51	Modules of approx. 150 residues found attached to various enzymes from families GH2, GH27, GH31, GH95, GH98 and GH101 . Binding to galactose and to blood group A/B-antigens demonstrated in the case of C. perfringens GH95CBM51 and GH98CBM51 respectively [Gregg KJ et al. (2008) J. Biol. Chem. 283:12604-13 PMID: 18292090].	642	638	1280
CBM54	Binding to xylan, yeast cell wall glucan and chitin shown in Dvortsov et al., Microbiology UK (2009) in press.	225	281	506
CBM56	beta-1,3-glucan binding function demonstrated by Yamamoto et al. (1998) FEBS Letters 433:41-43 [PMID: 9738929]	241	354	595
CBM57	Created from reading Schallus et al (2008) Mol Biol Cell. 19:3404-3414 [PMID: 18524852] and finding related domains attached to various glycosidases.	17	34	51
CBM58	The CBM58 module of the Bacteroides thetaiotaomicron SusG protein has been shown to bind maltoheptaose	4	0	4

CBM59	Binding to mannan, xylan, and cellulose demonstrated for the CBM59 of ManF-X10 xylanase from an environmental genomic DNA library (Li et al. (2009) World Journal of Microbiology and Biotechnology 25:2071-2078; doi:10.1007/s11274-009-0111-6)	4	4	7
CBM6	Modules of approx. 120 residues. The cellulose-binding function has been demonstrated in one case on amorphous cellulose and beta-1,4-xylan. Some of these modules also bind beta-1,3-glucan, beta-1,3-1,4-glucan, and beta-1,4-glucan.	723	337	1059
CBM61	Modules of approx. 150 residues found appended to GH16, GH30, GH31, GH43, GH53 and GH66 catalytic domains. A beta-1,4-galactan binding function has been demonstrated for the CBM61 of Thermotoga maritima GH53 galactanase [PMID: 20826814].	372	393	765
CBM62	The CBM62 module of Clostridium thermocellum Cthe_2193 protein binds galactose moieties found on xyloglucan, arabinogalactan and galactomannan.	247	290	536
CBM63	The CBM63 module of Bacillus subtilis expansin EXLX1 has been shown to bind cellulose.	7	3	11
CBM65	CBM65A and CBM65B, derived from Eubacterium cellulosolvens endoglucanase EcCel5A, bind to a range of beta-glucans but, uniquely, display significant preference for xyloglucan	278	105	383
CBM66	The CBM66 module, derived from the Bacillus subtilis exo-acting beta-fructosidase SacC, targets the terminal fructoside residue of fructans.	1086	933	2019

CBM67	Fujimoto et al. [PMID : 23486481] disclosed the L-rhamnose binding activity and 3-D structure of the CBM67 of Streptomyces avermitilis alpha-L-rhamnosidase (SaRha78A);	1545	1755	3300
CBM68	Binding to maltotriose and maltotetraose shown for the pullulanase of Anoxybacillus sp. LM18-11. Binding function derived from crystal structure and deletion of the CBM, which showed reduced specific activity and increased Km value compared to the wild type enzyme.	6	1	7
CBM69	starch-binding function demonstrated in one case; distantly related to families CBM20 and CBM48	75	74	149
CBM70	The hyaluronan-specific binding function of the N-terminal CBM70 module of Streptococcus pneumoniae hyaluronate lyase has been demonstrated.	49	90	139
CBM71	The two CBM71s of S. pneumoniae BgaA bind lactose and LacNAc.	25	28	53
CBM72	Modules of 130-180 residues found at the C-terminus glycoside hydrolases from various families, sometimes as tandem repeats. The CBM72 found on an endoglucanase from an uncultivated microorganism was found to bind a broad spectrum of polysaccharides including soluble and insoluble cellulose, beta-1,3/1,4-mixed linked glucans, xylan, and beta-mannan [PMID=26765840].	173	81	254

CBM74	Modules of approx. 300 residues appended to several alpha-amylases. The starch-binding function has been demonstrated for the CBM74 appended to the GH13 alpha-amylase of Microbacterium aureum B8.A	672	197	869
CBM75	Modules of 290 residues appended to GH43_16 enzymes. So far found exclusively in Ruminococci. The xyloglucan-binding function was demonstrated for the R. flavefaciens protein.	3	11	14
CBM76	Modules of approx. 170 residues appended to GH44 enzymes. So far found exclusively in Ruminococci. Broad specificity binding to beta-glucans demontsrated for the R. flavefaciens module, which binds xyloglucan, glucomannan, and barley beta-glucan.	3	20	23
CBM77	Pectin binding modules of approx. 110 residues. The Ruminococcus flavefaciens CBM77 was shown to bind various pectins of low degree of esterification.	281	179	459
CBM78	Modules of approx. 150 residues appended to the C-terminus of GH5 and GH26 enzymes. So far found exclusively in Ruminococcal enzymes. The R. flavefaciens module has been shown to bind decorated beta-1,4-glucans with a preference for xyloglucan.	7	4	11
CBM79	Modules of approx. 130 residues found so far only in ruminococcal proteins. Binding to various beta-glucans was shown for the R. flavefaciens GH9 enzyme.	56	66	122
CBM8	The cellulose-binding module from a cellulase of the slime mold Dictyostelium discoideum has been experimentally shown to bind cellulose.	5	8	12

CBM80	Modules of approx. 90 residues found so far only in ruminococcal enzymes of families GH5 or GH26. Broad specificity for beta-glycans (xyloglucan, glucomannan, galactomannan, barley beta-glucan).	13	7	19
CBM82	The boundaries, structure and starch-binding function of the CBM82 module of Eubacterium rectale Amy13K have been reported by Cockburn and coworkers in Molec. Microbiol. (2017) (PMID=29139580)	0	2	2
CBM83	The boundaries, structure and starch-binding function of the CBM83 module of Eubacterium rectale Amy13K have been reported by Cockburn and coworkers in Molec. Microbiol. (2017) (PMID=29139580)	1	1	1
CBM84	Modules of approx. 140 aminoacids appended to enzymes of different families of CAZymes. A xanthan-binding function was reported for the GH9 xanthanase of Paenibacillus nanensis by Moroz et al. ACS Catal., 2018, 8 (7), pp 6021-6034.	25	9	34
CBM9	Modules of approx. 170 residues found so far only in xylanases. The cellulose-binding function has been demonstrated in one case.	1832	1830	3662
CE1	acetyl xylan esterase (EC 3.1.1.72); cinnamoyl esterase (EC 3.1.1.-); feruloyl esterase (EC 3.1.1.73); carboxylesterase (EC 3.1.1.1); S-formylglutathione hydrolase (EC 3.1.2.12); diacylglycerol O-acyltransferase (EC 2.3.1.20); trehalose 6-O-mycolyltransferase (EC 2.3.1.122)	29185	31084	60269

CE10	arylesterase (EC 3.1.1.-); carboxyl esterase (EC 3.1.1.3); acetylcholinesterase (EC 3.1.1.7); cholinesterase (EC 3.1.1.8); sterol esterase (EC 3.1.1.13); brefeldin A esterase (EC 3.1.1.-).	28447	32614	61061
CE11	UDP-3-0-acyl N-acetylglucosamine deacetylase (EC 3.5.1.108).	3308	3471	6780
CE12	pectin acetylesterase (EC 3.1.1.-); rhamnogalacturonan acetylesterase (EC 3.1.1.-); acetyl xylan esterase (EC 3.1.1.72)	7985	6758	14743
CE13	pectin acetylesterase (EC 3.1.1.-)	665	1183	1848
CE14	N-acetyl-1-D-myo-inosityl-2-amino-2-deoxy-alpha-D-glucopyranoside deacetylase (EC 3.5.1.89); diacetylchitobiose deacetylase (EC 3.5.1.-); mycothiol S-conjugate amidase (EC 3.5.1.-)	1216	1865	3082
CE15	4-O-methyl-glucuronoyl methylesterase (EC 3.1.1.-)	1248	1564	2812
CE2	acetyl xylan esterase (EC 3.1.1.72).	7441	5362	12803
CE3	acetyl xylan esterase (EC 3.1.1.72).	15736	15749	31485
CE4	acetyl xylan esterase (EC 3.1.1.72); chitin deacetylase (EC 3.5.1.41); chitooligosaccharide deacetylase (EC 3.5.1.-); peptidoglycan GlcNAc deacetylase (EC 3.5.1.-); peptidoglycan N-acetylmuramic acid deacetylase (EC 3.5.1.-).	27386	28582	55968
CE5	acetyl xylan esterase (EC 3.1.1.72); cutinase (EC 3.1.1.74)	5	0	5
CE6	acetyl xylan esterase (EC 3.1.1.72).	427	606	1033
CE7	acetyl xylan esterase (EC 3.1.1.72); cephalosporin-C deacetylase (EC 3.1.1.41).	6849	6504	13353
CE8	pectin methylesterase (EC 3.1.1.11).	7468	5507	12974

CE9	N-acetylglucosamine 6-phosphate deacetylase (EC 3.5.1.25); N-acetylglucosamine 6-phosphate deacetylase (EC 3.5.1.80)	7146	10623	17769
GH1	beta-glucosidase (EC 3.2.1.21); beta-galactosidase (EC 3.2.1.23); beta-mannosidase (EC 3.2.1.25); beta-glucuronidase (EC 3.2.1.31); beta-xylosidase (EC 3.2.1.37); beta-D-fucosidase (EC 3.2.1.38); phlorizin hydrolase (EC 3.2.1.62); exo-beta-1,4-glucanase (EC 3.2.1.74); 6-phospho-beta-galactosidase (EC 3.2.1.85); 6-phospho-beta-glucosidase (EC 3.2.1.86); strictosidine beta-glucosidase (EC 3.2.1.105); lactase (EC 3.2.1.108); amygdalin beta-glucosidase (EC 3.2.1.117); prunasin beta-glucosidase (EC 3.2.1.118); vicianin hydrolase (EC 3.2.1.119); raucaffricine beta-glucosidase (EC 3.2.1.125); thioglucosidase (EC 3.2.1.147); beta-primeverosidase (EC 3.2.1.149); isoflavonoid 7-O-beta-apiosyl-beta-glucosidase (EC 3.2.1.161); ABA-specific beta-glucosidase (EC 3.2.1.175); DIMBOA beta-glucosidase (EC 3.2.1.182); beta-glycosidase (EC 3.2.1.-); hydroxyisourate hydrolase (EC 3.-.-.-)	7890	9147	17037
GH10	endo-1,4-beta-xylanase (EC 3.2.1.8); endo-1,3-beta-xylanase (EC 3.2.1.32); tomatinase (EC 3.2.1.-); xylan endotransglycosylase (EC 2.4.2.-); endo-beta-1,4-glucanase (EC 3.2.1.4)	4151	3406	7557
GH100	alkaline and neutral invertase (EC 3.2.1.26)	2	0	2
GH101	endo-alpha-N-acetylgalactosaminidase (EC 3.2.1.97)	266	859	1124
GH102	peptidoglycan lytic transglycosylase (EC 3.2.1.-)	40	409	449
GH103	peptidoglycan lytic transglycosylase (EC 3.2.1.-)	95	460	555

GH105	unsaturated rhamnogalacturonyl hydrolase (EC 3.2.1.172); d-4,5-unsaturated beta-glucuronyl hydrolase (EC 3.2.1.-); d-4,5-unsaturated alpha-galacturonidase (EC 3.2.1.-)	12639	10489	23128
GH106	alpha-L-rhamnosidase (EC 3.2.1.40); rhamnogalacturonan alpha-L-rhamnohydrolase (EC 3.2.1.174)	6139	7544	13683
GH107	sulfated fucan endo-1,4-fucanase (EC 3.2.1.-)	3	8	10
GH108	N-acetylmuramidase (EC 3.2.1.17)	586	975	1561
GH109	alpha-N-acetylgalactosaminidase (EC 3.2.1.49)	8746	10255	19001
GH11	endo-beta-1,4-xylanase (EC 3.2.1.8); endo-beta-1,3-xylanase (EC 3.2.1.32)	82	99	181
GH110	alpha-galactosidase (EC 3.2.1.22); alpha-1,3-galactosidase (EC 3.2.1.-)	2940	3547	6487
GH111	keratan sulfate hydrolase (endo-beta-N-acetylglucosaminidase) (EC 3.2.1.-)	9	6	15
GH112	lacto-N-biose phosphorylase or galacto-N-biose phosphorylase (EC 2.4.1.211); D-galactosyl-beta-1,4-L-rhamnose phosphorylase (EC 2.4.1.247); galacto-N-biose/lacto-N-biose phosphorylase (EC 2.4.1.-)	1661	2549	4210
GH113	beta-mannanase (EC 3.2.1.78)	1143	806	1950
GH114	endo-alpha-1,4-polygalactosaminidase (EC 3.2.1.109)	5	2	7
GH115	xylan alpha-1,2-glucuronidase (3.2.1.131); alpha-(4-O-methyl)-glucuronidase (3.2.1.-)	3742	2464	6206
GH116	beta-glucosidase (EC 3.2.1.21); beta-xylosidase (EC 3.2.1.37); acid beta-glucosidase/beta-glucosylceramidase (EC 3.2.1.45); beta-N-acetylglucosaminidase (EC 3.2.1.52)	147	258	404

GH117	alpha-1,3-L-neoagarooligosaccharide hydrolase (EC 3.2.1.-); alpha-1,3-L-neoagarobiase / neoagarobiose hydrolase (EC 3.2.1.-)	101	264	365
GH118	beta-agarase (EC 3.2.1.81)	2	4	5
GH119	alpha-amylase (EC 3.2.1.1)	609	560	1169
GH120	beta-xylosidase (EC 3.2.1.37)	46	46	92
GH121	beta-L-arabinobiosidase (EC 3.2.1.-)	179	173	352
GH122	alpha-glucosidase (EC 3.2.1.20)	3	0	3
GH123	beta-N-acetylgalactosaminidase (EC 3.2.1.53); glycosphingolipid beta-N-acetylgalactosaminidase (EC 3.2.1.-)	2188	3024	5212
GH124	endoglucanase (EC 3.2.1.4)	613	278	891
GH125	exo-alpha-1,6-mannosidase (EC 3.2.1.-)	1150	1766	2916
GH126	alpha-amylase (EC 3.2.1.-)	137	307	444
GH127	beta-L-arabinofuranosidase (EC 3.2.1.185); 3-C-carboxy-5-deoxy-L-xylose (aceric acid) hydrolase (EC 3.2.1.-)	4775	5075	9850
GH128	beta-1,3-glucanase (EC 3.2.1.39)	270	344	614
GH129	alpha-N-acetylgalactosaminidase (EC 3.2.1.49);	2583	1881	4464

GH13	alpha-amylase (EC 3.2.1.1); pullulanase (EC 3.2.1.41); cyclomaltodextrin glucanotransferase (EC 2.4.1.19); cyclomaltodextrinase (EC 3.2.1.54); trehalose-6-phosphate hydrolase (EC 3.2.1.93); oligo-alpha-glucosidase (EC 3.2.1.10); maltogenic amylase (EC 3.2.1.133); neopullulanase (EC 3.2.1.135); alpha-glucosidase (EC 3.2.1.20); maltotetraose-forming alpha-amylase (EC 3.2.1.60); isoamylase (EC 3.2.1.68); glucodextranase (EC 3.2.1.70); maltohexaose-forming alpha-amylase (EC 3.2.1.98); maltotriose-forming alpha-amylase (EC 3.2.1.116); branching enzyme (EC 2.4.1.18); trehalose synthase (EC 5.4.99.16); 4-alpha-glucanotransferase (EC 2.4.1.25); maltopentaose-forming alpha-amylase (EC 3.2.1.-) ; amylosucrase (EC 2.4.1.4) ; sucrose phosphorylase (EC 2.4.1.7); malto-oligosyltrehalose trehalohydrolase (EC 3.2.1.141); isomaltulose synthase (EC 5.4.99.11); malto-oligosyltrehalose synthase (EC 5.4.99.15); amylo-alpha-1,6-glucosidase (EC 3.2.1.33); alpha-1,4-glucan: phosphate alpha-maltosyltransferase (EC 2.4.99.16); 6'-P-sucrose phosphorylase (EC 2.4.1.-); amino acid transporter	9772	9450	19221

GH130	beta-1,4-mannosylglucose phosphorylase (EC 2.4.1.281); beta-1,4-mannooligosaccharide phosphorylase (EC 2.4.1.319); beta-1,4-mannosyl-N-acetyl-glucosamine phosphorylase (EC 2.4.1.320); beta-1,2-mannobiose phosphorylase (EC 2.4.1.-); beta-1,2-oligomannan phosphorylase (EC 2.4.1.-); beta-1,2-mannosidase (EC 3.2.1.-)	5524	5031	10554
GH132	Activity on beta-1,3-glucan (curdlan) shown for the Aspergillus fumigatus Sun4 protein; activity on laminarioligosaccharides shown for Aspergillus fumigatus Sun4 protein and Candida albicans Sun41 protein; transglycosylation activity reported in PMID 23508952.	2	0	2
GH133	amylo-alpha-1,6-glucosidase (EC 3.2.1.33);	4603	4108	8711
GH136	lacto-N-biosidase (EC 3.2.1.140)	609	417	1026
GH137	beta-L-arabinofuranosidase (EC 3.2.1.185)	80	154	234
GH138	alpha-galacturonidase (EC 3.2.1.-)	652	592	1244
GH139	alpha-2-O-Me-L-fucosidase (EC 3.2.1.-)	255	243	497
GH13_1	GH13's subfamily	55	61	116
GH13_10	GH13's subfamily	164	248	412
GH13_11	GH13's subfamily	2258	3109	5367
GH13_13	GH13's subfamily	20	34	54
GH13_14	GH13's subfamily	5383	4583	9966
GH13_16	GH13's subfamily	70	188	257
GH13_17	GH13's subfamily	1	0	1
GH13_18	GH13's subfamily	221	373	594
GH13_19	GH13's subfamily	429	407	836
GH13_2	GH13's subfamily	66	142	208
GH13_20	GH13's subfamily	6173	7060	13233
GH13_21	GH13's subfamily	214	180	394
GH13_23	GH13's subfamily	108	132	240

GH13_24	GH13's subfamily	45	32	77
GH13_25	GH13's subfamily	2	3	5
GH13_26	GH13's subfamily	7	5	12
GH13_27	GH13's subfamily	0	2	2
GH13_28	GH13's subfamily	2209	1125	3334
GH13_29	GH13's subfamily	240	478	718
GH13_3	GH13's subfamily	82	238	320
GH13_30	GH13's subfamily	1	4	4
GH13_31	GH13's subfamily	2415	4389	6805
GH13_33	GH13's subfamily	19	37	55
GH13_34	GH13's subfamily	11	9	20
GH13_35	GH13's subfamily	2	0	2
GH13_36	GH13's subfamily	3962	4741	8703
GH13_37	GH13's subfamily	361	496	857
GH13_38	GH13's subfamily	1751	2220	3971
GH13_39	GH13's subfamily	7908	8869	16777
GH13_4	GH13's subfamily	1103	1904	3008
GH13_40	GH13's subfamily	18	18	36
GH13_41	GH13's subfamily	51	38	88
GH13_42	GH13's subfamily	1723	1147	2869
GH13_5	GH13's subfamily	1348	1871	3219
GH13_6	GH13's subfamily	472	504	976
GH13_7	GH13's subfamily	20	63	82
GH13_8	GH13's subfamily	4284	4149	8432
GH13_9	GH13's subfamily	14149	14347	28497
GH14	beta-amylase (EC 3.2.1.2)	2	5	6
GH140	apiosidase (EC 3.2.1.-)	2331	2460	4792
GH141	alpha-L-fucosidase (EC 3.2.1.51); xylanase (EC 3.2.1.8)	693	1258	1951
GH142	beta-L-arabinofuranosidase (EC 3.2.1.185)	130	116	246
GH143	2-keto-3-deoxy-D-lyxo-heptulosaric acid hydrolase (EC 3.2.1.-)	1450	822	2271

GH144	endo-beta-1,2-glucanase (EC 3.2.1.71); beta-1,2-glucooligosaccharide sophorohydrolase (EC 3.2.1.-)	469	781	1249
GH145	L-Rhalpha-alpha-1,4-GlcA alpha-L-rhamnohydrolase (EC 3.2.1.-)	274	369	643
GH146	beta-L-arabinofuranosidase (EC 3.2.1.185)	1449	1883	3332
GH147	beta-galactosidase (EC 3.2.1.23)	275	351	627
GH148	beta-1,3-glucanase (EC 3.2.1.-)	19	46	65
GH149	beta-1,3-glucan phosphorylase (EC 2.4.1.-)	3	0	3
GH15	glucoamylase (EC 3.2.1.3); glucodextranase (EC 3.2.1.70); alpha,alpha-trehalase (EC 3.2.1.28); dextran dextrinase (EC 2.4.1.2)	487	1079	1565
GH150	l-carrageenase (EC 3.2.1.-)	45	10	55
GH151	alpha-L-fucosidase (EC 3.2.1.51)	281	175	457
GH153	poly-beta-1,6-D-glucosamine hydrolase (EC 3.2.1.-)	9	263	271
GH154	beta-glucuronidase (3.2.1.31)	1952	3466	5418
GH156	exo-alpha-sialidase (EC 3.2.1.18);	850	663	1513
GH157	endo-beta-1,3-glucanase (EC 3.2.1.39); endo-beta-1,3-glucanase / laminarinase (EC 3.2.1.39)	41	30	71
GH158	endo-beta-1,3-glucanase (EC 3.2.1.39)	119	261	380
GH159	beta-D-galactofuranosidase (EC 3.2.1.146)	102	141	242

GH16	xyloglucan:xyloglucosyltransferase (EC 2.4.1.207); keratan-sulfate endo-1,4-beta-galactosidase (EC 3.2.1.103); endo-1,3-beta-glucanase / laminarinase (EC 3.2.1.39); endo-1,3(4)-beta-glucanase (EC 3.2.1.6); licheninase (EC 3.2.1.73); beta-agarase (EC 3.2.1.81); kappa;-carrageenase (EC 3.2.1.83); xyloglucanase (EC 3.2.1.151); endo-beta-1,3-galactanase (EC 3.2.1.181); [retaining] beta-porphyranase (EC 3.2.1.178); hyaluronidase (EC 3.2.1.35); endo-beta-1,4-galactosidase (EC 3.2.1.-); chitin beta-1,6-glucanosyltransferase (EC 2.4.1.-); beta-transglycosidase (EC 2.4.1.-); beta-glycosidase (EC 3.2.1.-); endo-beta-1,3-galactanase (EC 3.2.1.181)	5034	5167	10201
GH160	endo-beta-1,4-galactosidase (EC 3.2.1.-)	5	0	5
GH161	beta-1,3-glucan phosphorylase (EC 2.4.1.-)	866	724	1590
GH163	endo-beta-N-acetylglucosaminidase cleaving GlcNAc-beta-1,2-Man (EC 3.2.1.-)	1125	1488	2612
GH164	beta-mannosidase (EC 3.2.1.25); beta-mannosidase (EC 3.2.1.25)	577	422	998
GH165	beta-galactosidase (EC 3.2.1.23)	111	154	265
GH17	glucan endo-1,3-beta-glucosidase (EC 3.2.1.39); glucan 1,3-beta-glucosidase (EC 3.2.1.58); licheninase (EC 3.2.1.73); ABA-specific beta-glucosidase (EC 3.2.1.175); beta-1,3-glucanosyltransglycosylase (EC 2.4.1.-)	44	33	76

GH18	chitinase (EC 3.2.1.14); lysozyme (EC 3.2.1.17); endo-beta-N-acetylglucosaminidase (EC 3.2.1.96); peptidoglycan hydrolase with endo-beta-N-acetylglucosaminidase specificity (EC 3.2.1.-); Nod factor hydrolase (EC 3.2.1.-); xylanase inhibitor; concanavalin B; narbonin	10365	12282	22647
GH19	chitinase (EC 3.2.1.14); lysozyme (EC 3.2.1.17)	135	231	366
GH2	beta-galactosidase (EC 3.2.1.23) ; beta-mannosidase (EC 3.2.1.25); beta-glucuronidase (EC 3.2.1.31); alpha-L-arabinofuranosidase (EC 3.2.1.55); mannosylglycoprotein endo-beta-mannosidase (EC 3.2.1.152); exo-beta-glucosaminidase (EC 3.2.1.165); alpha-L-arabinopyranosidase (EC 3.2.1.-); beta-galacturonidase (EC 3.2.1.-); beta-xylosidase (EC 3.2.1.37); beta-D-galactofuranosidase (EC 3.2.1.146);	60821	63101	123923
GH20	beta-hexosaminidase (EC 3.2.1.52); lacto-N-biosidase (EC 3.2.1.140); beta-1,6-N-acetylglucosaminidase (EC 3.2.1.-); beta-6-SO3-N-acetylglucosaminidase (EC 3.2.1.-)	10820	13734	24554
GH22	lysozyme type C (EC 3.2.1.17); lysozyme type i (EC 3.2.1.17); alpha-lactalbumin	3	1	5
GH23	lysozyme type G (EC 3.2.1.17); peptidoglycan lyase (EC 4.2.2.n1) also known in the literature as peptidoglycan lytic transglycosylase; chitinase (EC 3.2.1.14)	8392	7762	16154
GH24	lysozyme (EC 3.2.1.17)	2704	3710	6414
GH25	lysozyme (EC 3.2.1.17)	19876	16387	36263

GH26	beta-mannanase (EC 3.2.1.78); exo-beta-1,4-mannobiohydrolase (EC 3.2.1.100); beta-1,3-xylanase (EC 3.2.1.32); lichenase / endo-beta-1,3-1,4-glucanase (EC 3.2.1.73); mannobiose-producing exo-beta-mannanase (EC 3.2.1.-)	7897	5273	13170
GH27	alpha-galactosidase (EC 3.2.1.22); alpha-N-acetylgalactosaminidase (EC 3.2.1.49); isomalto-dextranase (EC 3.2.1.94); beta-L-arabinopyranosidase (EC 3.2.1.88); galactan:galactan galactosyltransferase (EC 2.4.1.-)	4117	4298	8415
GH28	polygalacturonase (EC 3.2.1.15); alpha-L-rhamnosidase (EC 3.2.1.40); exo-polygalacturonase (EC 3.2.1.67); exo-polygalacturonosidase (EC 3.2.1.82); rhamnogalacturonase (EC 3.2.1.171); rhamnogalacturonan alpha-1,2-galacturonohydrolase (EC 3.2.1.173); endo-xylogalacturonan hydrolase (EC 3.2.1.-)	14209	13363	27572
GH29	alpha-L-fucosidase (EC 3.2.1.51); alpha-1,3/1,4-L-fucosidase (EC 3.2.1.111)	8232	11409	19641

GH3	beta-glucosidase (EC 3.2.1.21); xylan 1,4-beta-xylosidase (EC 3.2.1.37); beta-glucosylceramidase (EC 3.2.1.45); beta-N-acetylhexosaminidase (EC 3.2.1.52); alpha-L-arabinofuranosidase (EC 3.2.1.55); glucan 1,3-beta-glucosidase (EC 3.2.1.58); glucan 1,4-beta-glucosidase (EC 3.2.1.74); isoprimeverose-producing oligoxyloglucan hydrolase (EC 3.2.1.120); coniferin beta-glucosidase (EC 3.2.1.126); exo-1,3-1,4-glucanase (EC 3.2.1.-); beta-N-acetylglucosaminide phosphorylases (EC 2.4.1.-)	20961	24922	45883
GH30	endo-beta-1,4-xylanase (EC 3.2.1.8); beta-glucosidase (3.2.1.21); beta-glucuronidase (EC 3.2.1.31); beta-xylosidase (EC 3.2.1.37); beta-fucosidase (EC 3.2.1.38); glucosylceramidase (EC 3.2.1.45); beta-1,6-glucanase (EC 3.2.1.75); glucuronoarabinoxylan endo-beta-1,4-xylanase (EC 3.2.1.136); endo-beta-1,6-galactanase (EC:3.2.1.164); [reducing end] beta-xylosidase (EC 3.2.1.-)	487	596	1083
GH30_1	GH30's subfamily	585	628	1212
GH30_2	GH30's subfamily	129	231	360
GH30_3	GH30's subfamily	531	544	1075
GH30_4	GH30's subfamily	1059	1448	2507
GH30_5	GH30's subfamily	447	795	1242
GH30_6	GH30's subfamily	21	2	23
GH30_7	GH30's subfamily	2	3	5
GH30_8	GH30's subfamily	228	98	326

GH31	alpha-glucosidase (EC 3.2.1.20); alpha-galactosidase (EC 3.2.1.22); alpha-mannosidase (EC 3.2.1.24); alpha-1,3-glucosidase (EC 3.2.1.84); sucrase-isomaltase (EC 3.2.1.48) (EC 3.2.1.10); alpha-xylosidase (EC 3.2.1.177); alpha-glucan lyase (EC 4.2.2.13); isomaltosyltransferase (EC 2.4.1.-); oligosaccharide alpha-1,4-glucosyltransferase (EC 2.4.1.161); sulfoquinovosidase (EC 3.2.1.-)	15499	17854	33353
GH32	invertase (EC 3.2.1.26); endo-inulinase (EC 3.2.1.7); beta-2,6-fructan 6-levanbiohydrolase (EC 3.2.1.64); endo-levanase (EC 3.2.1.65); exo-inulinase (EC 3.2.1.80); fructan beta-(2,1)-fructosidase/1-exohydrolase (EC 3.2.1.153); fructan beta-(2,6)-fructosidase/6-exohydrolase (EC 3.2.1.154); sucrose:sucrose 1-fructosyltransferase (EC 2.4.1.99); fructan:fructan 1-fructosyltransferase (EC 2.4.1.100); sucrose:fructan 6-fructosyltransferase (EC 2.4.1.10); fructan:fructan 6G-fructosyltransferase (EC 2.4.1.243); levan fructosyltransferase (EC 2.4.1.-); [retaining] sucrose:sucrose 6-fructosyltransferase (6-SST) (EC 2.4.1.-); cycloinulo-oligosaccharide fructanotransferase (EC 2.4.1.-)	6527	10078	16604
GH33	sialidase or neuraminidase (EC 3.2.1.18); trans-sialidase (EC 2.4.1.-); anhydrosialidase (EC 4.2.2.15); Kdo hydrolase (EC 3.2.1.-); 2-keto-3-deoxynononic acid hydrolase / KDNase (EC 3.2.1.-)	5786	6828	12614

GH35	beta-galactosidase (EC 3.2.1.23); exo-beta-glucosaminidase (EC 3.2.1.165); exo-beta-1,4-galactanase (EC 3.2.1.-); beta-1,3-galactosidase (EC 3.2.1.-)	3735	4454	8189
GH36	alpha-galactosidase (EC 3.2.1.22); alpha-N-acetylgalactosaminidase (EC 3.2.1.49); stachyose synthase (EC 2.4.1.67); raffinose synthase (EC 2.4.1.82)	12620	15756	28376
GH37	alpha,alpha-trehalase (EC 3.2.1.28).	393	342	735
GH38	alpha-mannosidase (EC 3.2.1.24); mannosyl-oligosaccharide alpha-1,2-mannosidase (EC 3.2.1.113); mannosyl-oligosaccharide alpha-1,3-1,6-mannosidase (EC 3.2.1.114); alpha-2-O-mannosylglycerate hydrolase (EC 3.2.1.170); mannosyl-oligosaccharide alpha-1,3-mannosidase (EC 3.2.1.-)	1035	2072	3107
GH39	alpha-L-iduronidase (EC 3.2.1.76); beta-xylosidase (EC 3.2.1.37).	3917	5318	9235
GH4	maltose-6-phosphate glucosidase (EC 3.2.1.122); alpha-glucosidase (EC 3.2.1.20); alpha-galactosidase (EC 3.2.1.22); 6-phospho-beta-glucosidase (EC 3.2.1.86); alpha-glucuronidase (EC 3.2.1.139); alpha-galacturonase (EC 3.2.1.67); palatinase (EC 3.2.1.-)	5513	4703	10216
GH42	beta-galactosidase (EC 3.2.1.23); alpha-L-arabinopyranosidase (EC 3.2.1.-)	4992	5541	10534

GH43	beta-xylosidase (EC 3.2.1.37); alpha-L-arabinofuranosidase (EC 3.2.1.55); xylanase (EC 3.2.1.8); alpha-1,2-L-arabinofuranosidase (EC 3.2.1.-); exo-alpha-1,5-L-arabinofuranosidase (EC 3.2.1.-); [inverting] exo-alpha-1,5-L-arabinanase (EC 3.2.1.-); beta-1,3-xylosidase (EC 3.2.1.-); [inverting] exo-alpha-1,5-L-arabinanase (EC 3.2.1.-); [inverting] endo-alpha-1,5-L-arabinanase (EC 3.2.1.99); exo-beta-1,3-galactanase (EC 3.2.1.145); beta-D-galactofuranosidase (EC 3.2.1.146)	967	1412	2379
GH43_1	GH43's subfamily	286	362	648
GH43_10	GH43's subfamily	5045	3765	8810
GH43_11	GH43's subfamily	744	673	1417
GH43_12	GH43's subfamily	3839	2854	6693
GH43_13	GH43's subfamily	1	1	2
GH43_14	GH43's subfamily	4	11	14
GH43_16	GH43's subfamily	968	752	1720
GH43_17	GH43's subfamily	799	949	1748
GH43_18	GH43's subfamily	253	268	521
GH43_19	GH43's subfamily	763	936	1698
GH43_2	GH43's subfamily	429	585	1014
GH43_20	GH43's subfamily	165	127	292
GH43_21	GH43's subfamily	12	17	29
GH43_22	GH43's subfamily	544	716	1261
GH43_23	GH43's subfamily	3	2	6
GH43_24	GH43's subfamily	1633	2207	3840
GH43_26	GH43's subfamily	1889	1756	3645
GH43_27	GH43's subfamily	595	565	1160
GH43_28	GH43's subfamily	1120	1686	2806
GH43_29	GH43's subfamily	1042	1145	2187

GH43_3	GH43's subfamily	293	471	764
GH43_30	GH43's subfamily	15	13	28
GH43_31	GH43's subfamily	216	510	725
GH43_32	GH43's subfamily	502	430	932
GH43_33	GH43's subfamily	271	536	807
GH43_34	GH43's subfamily	685	812	1498
GH43_35	GH43's subfamily	5558	5336	10894
GH43_36	GH43's subfamily	4	1	6
GH43_37	GH43's subfamily	159	157	315
GH43_4	GH43's subfamily	2899	3454	6353
GH43_5	GH43's subfamily	203	299	502
GH43_7	GH43's subfamily	144	308	451
GH43_8	GH43's subfamily	32	38	69
GH43_9	GH43's subfamily	337	192	529
GH44	endoglucanase (EC 3.2.1.4); xyloglucanase (EC 3.2.1.151)	1241	747	1988
GH46	chitosanase (EC 3.2.1.132)	26	73	99
GH47	alpha-mannosidase (EC 3.2.1.113)	8	10	18
GH48	reducing end-acting cellobiohydrolase (EC 3.2.1.176); endo-beta-1,4-glucanase (EC 3.2.1.4); chitinase (EC 3.2.1.14)	239	118	357
GH49	dextranase (EC 3.2.1.11); isopullulanase (EC 3.2.1.57); dextran 1,6-alpha-isomaltotriosidase (EC 3.2.1.95); sulfated arabinan endo-1,4-beta-L-arabinanase (EC 3.2.1.-)	4	51	55

GH5	endo-beta-1,4-glucanase / cellulase (EC 3.2.1.4); endo-beta-1,4-xylanase (EC 3.2.1.8); beta-glucosidase (EC 3.2.1.21); beta-mannosidase (EC 3.2.1.25); beta-glucosylceramidase (EC 3.2.1.45); glucan beta-1,3-glucosidase (EC 3.2.1.58); licheninase (EC 3.2.1.73); exo-beta-1,4-glucanase / cellodextrinase (EC 3.2.1.74); glucan endo-1,6-beta-glucosidase (EC 3.2.1.75); mannan endo-beta-1,4-mannosidase (EC 3.2.1.78); cellulose beta-1,4-cellobiosidase (EC 3.2.1.91); steryl beta-glucosidase (EC 3.2.1.104); endoglycoceramidase (EC 3.2.1.123); chitosanase (EC 3.2.1.132); beta-primeverosidase (EC 3.2.1.149); xyloglucan-specific endo-beta-1,4-glucanase (EC 3.2.1.151); endo-beta-1,6-galactanase (EC 3.2.1.164); hesperidin 6-O-alpha-L-rhamnosyl-beta-glucosidase (EC 3.2.1.168); beta-1,3-mannanase (EC 3.2.1.-); arabinoxylan-specific endo-beta-1,4-xylanase (EC 3.2.1.-); mannan transglycosylase (EC 2.4.1.-); lichenase / endo-beta-1,3-1,4-glucanase (EC 3.2.1.73); beta-glycosidase (EC 3.2.1.-); endo-beta-1,3-glucanase / laminarinase (EC 3.2.1.39); beta-N-acetylhexosaminidase (EC 3.2.1.52); chitosanase (EC 3.2.1.132); beta-D-galactofuranosidase (EC 3.2.1.146);	1255	1581	2836
GH50	beta-agarase (EC 3.2.1.81).	401	507	907
GH51	endoglucanase (EC 3.2.1.4); endo-beta-1,4-xylanase (EC 3.2.1.8); beta-xylosidase (EC 3.2.1.37); alpha-L-arabinofuranosidase (EC 3.2.1.55); lichenase / endo-beta-1,3-1,4-glucanase (EC 3.2.1.73)	9004	10911	19915

GH52	beta-xylosidase (EC 3.2.1.37).	0	7	7
GH53	endo-beta-1,4-galactanase (EC 3.2.1.89).	4941	5204	10146
GH55	exo-beta-1,3-glucanase (EC 3.2.1.58); endo-beta-1,3-glucanase (EC 3.2.1.39)	709	1453	2162
GH57	alpha-amylase (EC 3.2.1.1); alpha-galactosidase (EC 3.2.1.22); amylopullulanase (EC 3.2.1.41); cyclomaltodextrinase (EC 3.2.1.54); branching enzyme (EC 2.4.1.18); 4-alpha-glucanotransferase (EC 2.4.1.25)	3959	4090	8049
GH59	beta-galactosidase (EC 3.2.1.23); galactocerebrosidase (EC 3.2.1.46)	965	540	1505
GH5_1	GH5's subfamily	1007	538	1544
GH5_10	GH5's subfamily	560	330	890
GH5_11	GH5's subfamily	1	0	1
GH5_12	GH5's subfamily	1	0	1
GH5_13	GH5's subfamily	211	307	518
GH5_15	GH5's subfamily	2	2	3
GH5_18	GH5's subfamily	2	0	2
GH5_19	GH5's subfamily	127	104	231
GH5_2	GH5's subfamily	3905	3677	7582
GH5_22	GH5's subfamily	63	26	89
GH5_24	GH5's subfamily	0	1	1
GH5_25	GH5's subfamily	22	23	45
GH5_28	GH5's subfamily	162	126	288
GH5_29	GH5's subfamily	2	3	5
GH5_31	GH5's subfamily	2	0	3
GH5_35	GH5's subfamily	84	45	129
GH5_36	GH5's subfamily	185	273	458
GH5_37	GH5's subfamily	1551	999	2551
GH5_38	GH5's subfamily	7	0	7
GH5_39	GH5's subfamily	5	18	23
GH5_4	GH5's subfamily	7907	4343	12250

GH5_40	GH5's subfamily	19	13	33
GH5_41	GH5's subfamily	5	0	5
GH5_42	GH5's subfamily	11	9	21
GH5_43	GH5's subfamily	11	6	17
GH5_44	GH5's subfamily	431	432	863
GH5_45	GH5's subfamily	5	13	19
GH5_46	GH5's subfamily	945	884	1829
GH5_47	GH5's subfamily	7	2	10
GH5_5	GH5's subfamily	5	3	8
GH5_52	GH5's subfamily	4	8	12
GH5_54	GH5's subfamily	203	210	413
GH5_7	GH5's subfamily	567	556	1123
GH5_8	GH5's subfamily	601	394	995
GH62	alpha-L-arabinofuranosidase (EC 3.2.1.55)	2	3	5
GH63	processing alpha-glucosidase (EC 3.2.1.106); alpha-1,3-glucosidase (EC 3.2.1.84); alpha-glucosidase (EC 3.2.1.20); mannosylglycerate alpha-mannosidase / mannosylglycerate hydrolase (EC 3.2.1.170); glucosylglycerate hydrolase (EC 3.2.1.208)	931	1260	2191
GH65	alpha,alpha-trehalase (EC 3.2.1.28); maltose phosphorylase (EC 2.4.1.8); trehalose phosphorylase (EC 2.4.1.64); kojibiose phosphorylase (EC 2.4.1.230); trehalose-6-phosphate phosphorylase (EC 2.4.1.216); nigerose phosphorylase (EC 2.4.1.279); 3-O-alpha-glucopyranosyl-L-rhamnose phosphorylase (EC 2.4.1.282); 2-O-alpha-glucopyranosylglycerol: phosphate beta-glucosyltransferase (EC 2.4.1.-); alpha-glucosyl-1,2-beta-galactosyl-L-hydroxylysine alpha-glucosidase (EC 3.2.1.107)	2107	2464	4571

GH66	cycloisomaltooligosaccharide glucanotransferase (EC 2.4.1.248); dextranase (EC 3.2.1.11).	161	863	1024
GH67	alpha-glucuronidase (EC 3.2.1.139); xylan alpha-1,2-glucuronidase (EC 3.2.1.131)	1373	1128	2501
GH68	levansucrase (EC 2.4.1.10); beta-fructofuranosidase (EC 3.2.1.26); inulosucrase (EC 2.4.1.9).	1	0	1
GH70	dextransucrase (EC 2.4.1.5); alternansucrase (EC 2.4.1.140); reuteransucrase (EC 2.4.1.-); alpha-4,6-glucanotransferase (EC 2.4.1.-); alpha-1,2-branched dextransucrase (EC 2.4.1.-); alpha-4,3-glucanotransferase (EC 2.4.1.-)	178	280	458
GH72	beta-1,3-glucanosyltransglycosylase (EC 2.4.1.-)	22	19	42
GH73	lysozyme (EC 3.2.1.17); mannosyl-glycoprotein endo-beta-N-acetylglucosaminidase (EC 3.2.1.96); peptidoglycan hydrolase with endo-beta-N-acetylglucosaminidase specificity (EC 3.2.1.-)	5390	5716	11107
GH74	endoglucanase (EC 3.2.1.4); oligoxyloglucan reducing end-specific cellobiohydrolase (EC 3.2.1.150); xyloglucanase (EC 3.2.1.151)	1400	1566	2966
GH75	chitosanase (EC 3.2.1.132)	277	308	584
GH76	alpha-1,6-mannanase (EC 3.2.1.101); alpha-glucosidase (EC 3.2.1.20)	2791	3064	5855
GH77	amylomaltase or 4-alpha-glucanotransferase (EC 2.4.1.25)	13605	14913	28518
GH78	alpha-L-rhamnosidase (EC 3.2.1.40); rhamnogalacturonan alpha-L-rhamnohydrolase (EC 3.2.1.174); L-Rhap-alpha-1,3-D-Apif -specific alpha-1,3-L-rhamnosidase (EC 3.2.1.-)	16350	23136	39486

GH79	beta-glucuronidase (EC 3.2.1.31); hyaluronoglucuronidase (EC 3.2.1.36); heparanase (EC 3.2.1.166); baicalin beta-glucuronidase (EC 3.2.1.167); beta-4-O-methyl-glucuronidase (EC 3.2.1.-)	1643	2222	3865
GH8	chitosanase (EC 3.2.1.132); cellulase (EC 3.2.1.4); licheninase (EC 3.2.1.73); endo-1,4-beta-xylanase (EC 3.2.1.8); reducing-end-xylose releasing exo-oligoxylanase (EC 3.2.1.156)	1025	898	1923
GH81	endo-beta-1,3-glucanase (EC 3.2.1.39)	147	64	211
GH82	Iota;-carrageenase (EC 3.2.1.157)	45	23	68
GH83	neuraminidase (EC 3.2.1.18)	0	7	7
GH84	N-acetyl beta-glucosaminidase (EC 3.2.1.52); hyaluronidase (EC 3.2.1.35); [protein]-3-O-(GlcNAc)-L-Ser/Thr beta-N-acetylglucosaminidase (EC 3.2.1.169)	2566	2826	5392
GH85	endo-beta-N-acetylglucosaminidase (EC 3.2.1.96)	132	129	261
GH86	beta-agarase (EC 3.2.1.81); beta-porphyranase (EC 3.2.1.178)	3	4	7
GH87	mycodextranase (EC 3.2.1.61); alpha-1,3-glucanase (EC 3.2.1.59)	144	419	563
GH88	d-4,5-unsaturated beta-glucuronyl hydrolase (EC 3.2.1.-)	3663	5068	8731
GH89	alpha-N-acetylglucosaminidase (EC 3.2.1.50)	3039	3089	6127

GH9	endoglucanase (EC 3.2.1.4); endo-beta-1,3(4)-glucanase / lichenase-laminarinase (EC 3.2.1.6); beta-glucosidase (EC 3.2.1.21); lichenase / endo-beta-1,3-1,4-glucanase (EC 3.2.1.73); exo-beta-1,4-glucanase / cellodextrinase (EC 3.2.1.74); cellobiohydrolase (EC 3.2.1.91); xyloglucan-specific endo-beta-1,4-glucanase / endo-xyloglucanase (EC 3.2.1.151); exo-beta-glucosaminidase (EC 3.2.1.165)	7736	5360	13096
GH91	inulin lyase [DFA-I-forming] (EC 4.2.2.17); inulin lyase [DFA-III-forming] (EC 4.2.2.18); difructofuranose 1,2':2,3' dianhydride hydrolase [DFA-IIIase] (EC 3.2.1.-)	62	31	93
GH92	mannosyl-oligosaccharide alpha-1,2-mannosidase (EC 3.2.1.113); mannosyl-oligosaccharide alpha-1,3-mannosidase (EC 3.2.1.-); mannosyl-oligosaccharide alpha-1,6-mannosidase (EC 3.2.1.-); alpha-mannosidase (EC 3.2.1.24); alpha-1,2-mannosidase (EC 3.2.1.-); alpha-1,3-mannosidase (EC 3.2.1.-); alpha-1,4-mannosidase (EC 3.2.1.-); mannosyl-1-phosphodiester alpha-1,P-mannosidase (EC 3.2.1.-)	5842	7618	13460
GH93	exo-alpha-L-1,5-arabinanase (EC 3.2.1.-)	258	271	529
GH94	cellobiose phosphorylase (EC 2.4.1.20); laminaribiose phosphorylase (EC 2.4.1.31); cellodextrin phosphorylase (EC 2.4.1.49); chitobiose phosphorylase (EC 2.4.1.-); cyclic beta-1,2-glucan synthase (EC 2.4.1.-); cellobionic acid phosphorylase (EC 2.4.1.321); beta-1,2-oligoglucan phosphorylase (EC 2.4.1.-)	10924	13093	24017

GH95	alpha-L-fucosidase (EC 3.2.1.51); alpha-1,2-L-fucosidase (EC 3.2.1.63); alpha-L-galactosidase (EC 3.2.1.-)	11119	10614	21733
GH97	glucoamylase (EC 3.2.1.3); alpha-glucosidase (EC 3.2.1.20); alpha-galactosidase (EC 3.2.1.22)	5225	5710	10935
GH98	blood-group endo-beta-1,4-galactosidase (EC 3.2.1.102); blood group A- and B-cleaving endo-beta-1,4-galactosidase (EC 3.2.1.-); endo-beta-1,4-xylanase (EC 3.2.1.8); endo-beta-1,4-xylanase (EC 3.2.1.8)	201	152	353
GH99	glycoprotein endo-alpha-1,2-mannosidase (EC 3.2.1.130); mannan endo-1,2-alpha-mannanase (3.2.1.-)	2866	2090	4956

GT1	UDP-glucuronosyltransferase (EC 2.4.1.17); zeatin O-beta-xylosyltransferase (EC 2.4.2.40); 2-hydroxyacylsphingosine 1-beta-galactosyltransferase (EC 2.4.1.45); N-acylsphingosine galactosyltransferase (EC 2.4.1.47); flavonol 3-O-glucosyltransferase (EC 2.4.1.91); anthocyanidin 3-O-glucosyltransferase (EC 2.4.1.115); sinapate 1-glucosyltransferase (EC 2.4.1.120); indole-3-acetate beta-glucosyltransferase (EC 2.4.1.121); flavonol L-rhamnosyltransferase (EC 2.4.1.159); sterol glucosyltransferase (EC 2.4.1.173); UDP-Glc: 4-hydroxybenzoate 4-O-beta-glucosyltransferase (EC 2.4.1.194); zeatin O-beta-glucosyltransferase (EC 2.4.1.203); limonoid glucosyltransferase (EC 2.4.1.210); UDP-GlcA: baicalein 7-O-beta-glucuronosyltransferase (EC 2.4.1.253); UDP-Glc: chalcone 4a2-O-beta-glucosyltransferase (EC 2.4.1.286); ecdysteroid UDP-glucosyltransferase (EC 2.4.1.-); salicylic acid beta-glucosyltransferase (EC 2.4.1.-); anthocyanin 3-O-galactosyltransferase (EC 2.4.1.-); anthocyanin 5-O-glucosyltransferase (EC 2.4.1.-); dTDP-beta-2-deoxy-L-fucose: alpha-L-2-deoxyfucosyltransferase (EC 2.4.1.-); UDP-beta-L-rhamnose: alpha-L-rhamnosyltransferase (EC 2.4.1.-); zeaxanthin glucosyltransferase (EC 2.4.1.-); flavone 8-C-glycosyltransferase	1216	827	2043
GT10	galactoside alpha-1,3/1,4-L-fucosyltransferase (EC 2.4.1.65); galactoside alpha-1,3-L-fucosyltransferase (EC 2.4.1.152); glycoprotein alpha-1,3-L-fucosyltransferase (EC 2.4.1.214)	1368	2662	4029
GT101	glucosyltransferase (EC 2.4.1.-)	1351	1387	2738

GT102	dTDP-beta-L-Rhap : O-antigen-polysaccharide alpha-1,3-L-rhamnosyltransferase (EC 2.4.1.289)	158	54	212
GT103	UDP-GlcpNAc: O-antigen-polysaccharide beta-1,4-N-acetylglucosaminyltransferase (EC 2.4.1.56)	92	0	92
GT104	dTDP-beta-L-Rhap : arginine alpha-L-rhamnosyltransferase (EC 2.4.1.-)	1	209	210
GT107	CMP-beta-KDO: beta-2,4-KDO transferase (EC 2.4.99.-); CMP-beta-KDO: beta-2,7-KDO transferase (EC 2.4.99.-)	216	118	334
GT11	GDP-L-Fuc: galactoside alpha-1,2-L-fucosyltransferase (EC 2.4.1.69); GDP-L-Fuc: beta-LacNac alpha-1,3-L-fucosyltransferase (EC 2.4.1.-)	6518	5708	12226
GT12	[N-acetylneuraminyl]-galactosylglucosylceramide N-acetylgalactosaminyltransferase (EC 2.4.1.92).	73	61	134
GT13	alpha-1,3-mannosyl-glycoprotein beta-1,2-N-acetylglucosaminyltransferase (EC 2.4.1.101)	1249	1555	2804
GT14	beta-1,3-galactosyl-O-glycosyl-glycoprotein beta-1,6-N-acetylglucosaminyltransferase (EC 2.4.1.102); N-acetyllactosaminide beta-1,6-N-acetylglucosaminyltransferase (EC 2.4.1.150); protein O-beta-xylosyltransferase (EC 2.4.2.26); UDP-GlcA:arabinogalactan beta-glucuronosyltransferase (EC 2.4.1.-)	1151	818	1969
GT15	glycolipid 2-alpha-mannosyltransferase (EC 2.4.1.131); GDP-Man: alpha-1,2-mannosyltransferase (EC 2.4.1.-).	1	0	1
GT17	beta-1,4-mannosyl-glycoprotein beta-1,4-N-acetylglucosaminyltransferase (EC 2.4.1.144).	750	596	1346

GT19	lipid-A-disaccharide synthase (EC 2.4.1.182).	5680	7335	13015
GT20	alpha,alpha-trehalose-phosphate synthase [UDP-forming] (EC 2.4.1.15); Glucosylglycerol-phosphate synthase (EC 2.4.1.213); trehalose-6-P phosphatase (EC 3.1.3.12); [retaining] GDP-valeniol: validamine 7-phosphate valeniolyltransferase (EC 2.-.-.-)	40	230	270
GT21	UDP-Glc: ceramide beta-glucosyltransferase (EC 2.4.1.80).	405	387	792
GT22	Dol-P-Man: Man6GlcNAc2-PP-Dol alpha-1,2-mannosyltransferase (EC 2.4.1.259); Dol-P-Man: Man8GlcNAc2-PP-Dol alpha-1,2-mannosyltransferase (EC 2.4.1.261); Dol-P-Man: Man2-GlcNAc-phosphatidylinositol alpha-1,2-mannosyltransferase (EC 2.4.1.-); Dol-P-Man: Man3-GlcNAc-phosphatidylinositol alpha-1,2-mannosyltransferase (EC 2.4.1.-)	75	126	201
GT23	N-acetyl-beta-D-glucosaminide alpha-1,6-L-fucosyltransferase (EC 2.4.1.68); chitin-oligosaccharide alpha-1,6-L-fucosyltransferase (EC 2.4.1.-)	2761	2093	4854
GT25	lipopolysaccharide beta-1,4-galactosyltransferase (EC 2.4.1.-); beta-1,3-glucosyltransferase (EC 2.4.1.-); beta-1,2-glucosyltransferase (EC 2.4.1.-); beta-1,2-galactosyltransferase (EC 2.4.1.-); LPS beta-1,4-galactosyltransferase (EC 2.4.1.-); occidiofungin beta-xylosyltransferase (EC 2.4.2.-); UDP-Gal:procollagen beta-galactosyltransferase (EC 2.4.1.50)	813	722	1536

GT26	UDP-ManNAcA: beta-N-acetyl mannosaminuronyltransferase (EC 2.4.1.-); UDP-ManNAc: beta-N-acetyl-mannosaminyltransferase (EC 2.4.1.-); UDP-Glc: beta-1,4-glucosyltransferase (EC 2.4.1.-); beta-1,4-galactosyltransferase (EC 2.4.1.-)	3932	5390	9322
GT27	polypeptide alpha-N-acetylgalactosaminyltransferase (EC 2.4.1.41)	2974	3186	6160
GT28	1,2-diacylglycerol 3-beta-galactosyltransferase (EC 2.4.1.46); 1,2-diacylglycerol 3-beta-glucosyltransferase (EC 2.4.1.157); UDP-GlcNAc: Und-PP-MurAc-pentapeptide beta-N-acetylglucosaminyltransferase (EC 2.4.1.227); digalactosyldiacylglycerol synthase (EC 2.4.1.241)	16179	18609	34787
GT29	sialyltransferase (EC 2.4.99.-); beta-galactoside alpha-2,6-sialyltransferase (EC 2.4.99.1); alpha-N-acetylgalactosaminide alpha-2,6-sialyltransferase (EC 2.4.99.3); beta-galactoside alpha-2,3-sialyltransferase (EC 2.4.99.4); N-acetyllactosaminide alpha-2,3-sialyltransferase (EC 2.4.99.6); (alpha-N-acetyl-neuraminyl-2,3-beta-galactosyl-1,3)-N-acetylgalactosaminide alpha-2,6-sialyltransferase (EC 2.4.99.7); alpha-N-acetyl-neuraminide alpha-2,8-sialyltransferase (EC 2.4.99.8); lactosylceramide alpha-2,3-sialyltransferase (EC 2.4.99.9)	8	187	195
2_Cellulose_s	GT2's subfamily	0	3	3
2_Chitin_synt	GT2's subfamily	16	16	32
_Glyco_tranf_	GT2's subfamily	1254	1336	2590
_Glyco_tranf_	GT2's subfamily	10211	13701	23912
_Glyco_tranf_	GT2's subfamily	192	242	435

2_Glyco_tranf_	GT2's subfamily	792	709	1500
_Glyco_trans_	GT2's subfamily	1305	1352	2656
2_Glycos_tran	GT2's subfamily	137728	142382	280111
GT3	glycogen synthase (EC 2.4.1.11).	1903	1637	3541
GT30	CMP-beta-KDO: alpha-3-deoxy-D-manno-octulosonic-acid (KDO) transferase (EC 2.4.99.-).	2639	3150	5789
GT31	N-acetyllactosaminide beta-1,3-N-acetylglucosaminyltransferase (EC 2.4.1.149); Glycoprotein-N-acetylgalactosamine 3-beta-galactosyltransferase (EC 2.4.1.122); fucose-specific beta-1,3-N-acetylglucosaminyltransferase (EC 2.4.1.-); globotriosylceramide beta-1,3-GalNAc transferase (EC 2.4.1.79); chondroitin synthase (beta-1,3-GlcUA and beta-1,4-GalNAc transferase (EC 2.4.1.175); chondroitin beta-1,3-glucuronyltransferase (EC 2.4.1.226); chondroitin beta-1,4-N-acetylgalactosaminyltransferase (EC 2.4.1.-); UDP-Gal: beta-galactosylxylosylprotein beta-1,3-galactosyltransferase (EC 2.4.1.134); UDP-GlcNAc: O-fucosylpeptide beta-1,3-N-acetylglucosaminyltransferase (EC 2.4.1.222)	39	0	40

GT32	alpha-1,6-mannosyltransferase (EC 2.4.1.-); alpha-1,4-N-acetylglucosaminyltransferase (EC 2.4.1.-); alpha-1,4-N-acetylgalactosaminyltransferase (EC 2.4.1.-); GDP-Man: inositol-phosphorylceramide transferase (EC 2.4.1.-); UDP-Gal: beta-galactoside alpha-1,4-galactosyltransferase (EC 2.4.1.-); UDP-Gal: lactose/N-acetyl-lactosamine alpha-1,4-galactosyltransferase (EC 2.4.1.-); UDP-Glc: protein alpha-glucosyltransferase (EC 2.4.1.-)	3203	2510	5713
GT33	GDP-Man: chitobiosyldiphosphodolichol beta-mannosyltransferase (EC 2.4.1.142).	178	63	241
GT35	glycogen or starch phosphorylase (EC 2.4.1.1).	18449	20390	38839
GT38	polysialyltransferase (EC 2.4.-.-)	5	20	25
GT39	Dol-P-Man: protein alpha-mannosyltransferase (EC 2.4.1.109)	1922	3324	5245

GT4	sucrose synthase (EC 2.4.1.13); sucrose-phosphate synthase (EC 2.4.1.14); alpha-glucosyltransferase (EC 2.4.1.52); lipopolysaccharide N-acetylglucosaminyltransferase (EC 2.4.1.56); phosphatidylinositol alpha-mannosyltransferase (EC 2.4.1.57); GDP-Man: Man1GlcNAc2-PP-dolichol alpha-1,3-mannosyltransferase (EC 2.4.1.132); GDP-Man: Man3GlcNAc2-PP-dolichol/Man4GlcNAc2-PP-dolichol alpha-1,2-mannosyltransferase (EC 2.4.1.131); digalactosyldiacylglycerol synthase (EC 2.4.1.141); 1,2-diacylglycerol 3-glucosyltransferase (EC 2.4.1.157); diglucosyl diacylglycerol synthase (EC 2.4.1.208); trehalose phosphorylase (EC 2.4.1.231); NDP-Glc: alpha-glucose alpha-glucosyltransferase / alpha,alpha-trehalose synthase (EC 2.4.1.245); GDP-Man: Man2GlcNAc2-PP-dolichol alpha-1,6-mannosyltransferase (EC 2.4.1.257); UDP-GlcNAc: 2-deoxystreptamine alpha-N-acetylglucosaminyltransferase (EC 2.4.1.283); UDP-GlcNAc: ribostamycin alpha-N-acetylglucosaminyltransferase (EC 2.4.1.285); UDP-Gal alpha-galactosyltransferase (EC 2.4.1.-); UDP-Xyl alpha-xylosyltransferase (EC 2.4.2.-); UDP-GlcA alpha-glucuronyltransferase (EC 2.4.1.-); UDP-Glc alpha-glucosyltransferase (EC 2.4.1.-); UDP-GalNAc: GalNAc-PP-Und alpha-1,3-N-acetylgalactosaminyltransferase (EC 2.4.1.306); UDP-GalNAc: N,N'-diacetylbacillosaminyl-PP-Und alpha-1,3-N-acetylgalactosaminyltransferase (EC 2.4.1.290); ADP-dependent	83400	90294	173694
GT40	beta-1,3-galactofuranosyltransferases (EC 2.4.1.-)	17	102	119
GT41	UDP-GlcNAc: peptide beta-N-acetylglucosaminyltransferase (EC 2.4.1.255); UDP-Glc: peptide N-beta-glucosyltransferase (EC 2.4.1.-)	25854	29734	55588

GT42	CMP-NeuAc alpha-2,3-sialyltransferase (EC 2.4.99.-)	72	14	86
GT45	alpha-N-acteylglucosaminyltransferase (EC 2.4.1.-)	28	2	30
GT46	Deleted	369	441	810
GT47	heparan beta-glucuronyltransferase (EC 2.4.1.225); xyloglucan beta-galactosyltransferase (EC 2.4.1.-); heparan synthase (EC 2.4.1.-); arabinan alpha-L-arabinosyltransferase (EC 2.4.2.-).	12	16	28
GT48	1,3-beta-glucan synthase (EC 2.4.1.34)	1	0	1
GT49	beta-1,3-N-acetylglucosaminyltransferase (EC 2.4.1.-).	1	0	1
GT5	UDP-Glc: glycogen glucosyltransferase (EC 2.4.1.11); ADP-Glc: starch glucosyltransferase (EC 2.4.1.21); NDP-Glc: starch glucosyltransferase (EC 2.4.1.242); UDP-Glc: alpha-1,3-glucan synthase (EC 2.4.1.183) UDP-Glc: alpha-1,4-glucan synthase (EC 2.4.1.-)	17632	19362	36995
GT50	Dol-P-Man alpha-1,4-mannosyltransferase (EC 2.4.1.-)	3	5	8
GT51	murein polymerase (EC 2.4.1.129).	3558	4467	8025
GT52	alpha-2,3-sialyltransferase (EC 2.4.99.4); alpha-glucosyltransferase (EC 2.4.1.-)	0	5	5
GT54	UDP-GlcNAc: alpha-1,3-D-mannoside beta-1,4-N-acetylglucosaminyltransferase (EC 2.4.1.145)	2	1	3
GT55	GDP-Man: mannosyl-3-phosphoglycerate synthase (EC 2.4.1.217)	15	24	39
GT56	TDP-Fuc4NAc: lipid II Fuc4NAc transferase (EC 2.4.1.-)	715	525	1239

GT57	Dol-P-Glc: alpha-1,3-glucosyltransferase (EC 2.4.1.-)	35	69	104
GT58	Dol-P-Man: Man5GlcNAc2-PP-Dol alpha-1,3-mannosyltransferase (EC 2.4.1.258)	17	14	30
GT59	Dol-P-Glc: Glc2Man9GlcNAc2-PP-Dol alpha-1,2-glucosyltransferase (EC 2.4.1.256)	47	10	57
GT6	alpha-1,3-galactosyltransferase (EC 2.4.1.87); alpha-1,3 N-acetylgalactosaminyltransferase (EC 2.4.1.40); alpha-galactosyltransferase (EC 2.4.1.37); globoside alpha-N-acetylgalactosaminyltransferase (EC 2.4.1.88).	139	282	421
GT60	UDP-GlcNAc: polypeptide alpha-N-acetylglucosaminyltransferase (EC 2.4.1.-); UDP-GlcNAc: hydroxyproline polypeptide alpha-N-acetylglucosaminyltransferase (EC 2.4.1.-)	35	6	41
GT61	beta-1,2-xylosyltransferase (EC 2.4.2.38) ; protein O-beta-N-acetylglucosaminyltransferase (EC 2.4.1.94) ; xylan alpha-1,3-arabinofuranosyltransferase (EC 2.4.2.-) ;	0	1	1
GT64	UDP-GlcNAc: heparan alpha-N-acetylhexosaminyltransferase (EC 2.4.1.224)	5	0	5
GT65	GDP-Fuc: protein O-alpha-fucosyltransferase (EC 2.4.1.-)	0	2	2
GT66	dolichyl-diphosphooligosaccharidea protein glycotransferase (EC 2.4.99.18); undecaprenyl-diphosphooligosaccharidea protein glycotransferase (EC 2.4.99.19)	1105	928	2034

GT7	lactose synthase (EC 2.4.1.22); beta-N-acetylglucosaminyl-glycopeptide beta-1,4-galactosyltransferase (EC 2.4.1.38); N-acetyllactosamine synthase (EC 2.4.1.90); xylosylprotein beta-4-galactosyltransferase (EC 2.4.1.133); UDP-Gal: neolactotriaosylceramide beta-1,4-galactosyltransferase (EC 2.4.1.275); beta-1,4-N-acetylglucosaminyltransferase (EC 2.4.1.-)	9	8	17
GT70	UDP-GlcA: beta-glucuronosyltransferase (EC 2.4.1.17)	165	81	246
GT73	CMP-beta-KDO: alpha-3-deoxy-D-manno-octulosonic-acid (KDO) transferase (EC 2.4.99.-).	639	388	1026
GT74	alpha-1,2-L-fucosyltransferase (EC 2.4.1.69)	10	9	19
GT75	UDP-Glc: self-glucosylating beta-glucosyltransferase (EC 2.4.1.-); UDP-L-arabinopyranose mutase (EC 5.4.99.-)	25	12	37
GT76	Dol-P-Man: alpha-1,6-mannosyltransferase (EC 2.4.1.-)	620	910	1530
GT8	lipopolysaccharide alpha-1,3-galactosyltransferase (EC 2.4.1.44); UDP-Glc: (glucosyl)lipopolysaccharide alpha-1,2-glucosyltransferase (EC 2.4.1.-); lipopolysaccharide glucosyltransferase 1 (EC 2.4.1.58); glycogenin glucosyltransferase (EC 2.4.1.186); inositol 1-alpha-galactosyltransferase (galactinol synthase) (EC 2.4.1.123); homogalacturonan alpha-1,4-galacturonosyltransferase (EC 2.4.1.43); UDP-GlcA: xylan alpha-glucuronyltransferase (EC 2.4.1.-)	10993	11954	22947

GT80	beta-galactoside alpha-2,6-sialyltransferase (EC 2.4.99.1); beta-galactoside alpha-2,3-sialyltransferase (EC 2.4.99.4)	65	92	157
GT81	NDP-Glc: glucosyl-3-phosphoglycerate synthase (EC 2.4.1.-); NDP-Man: mannosyl-3-phosphoglycerate synthase (EC 2.4.1.-);	675	315	990
GT82	UDP-GalNAc: beta-1,4-N-acetylgalactosaminyltransferase (EC 2.4.1.-)	60	89	150
GT83	undecaprenyl phosphate-alpha-L-Ara4N: 4-amino-4-deoxy-beta-L-arabinosyltransferase (EC 2.4.2.43); dodecaprenyl phosphate-beta-galacturonic acid: lipopolysaccharide core alpha-galacturonosyl transferase (EC 2.4.1.-)	7857	9328	17184
GT84	cyclic beta-1,2-glucan synthase (EC 2.4.1.-);	407	507	915
GT85	beta-D-arabinofuranosyl monophosphoryldecaprenol: galactan alpha-D-arabinofuranosyltransferase (EC 2.4.2.-)	3	4	7
GT87	polyprenol-P-Man: alpha-1,2-mannosyltransferase (EC 2.4.1.-)	168	218	385
GT89	beta-D-arabinofuranosyl-1-monophosphoryldecaprenol : arabinan beta-1,2-arabinofuranosyltransferase (EC 2.4.2.-)	26	11	37
GT9	lipopolysaccharide N-acetylglucosaminyltransferase (EC 2.4.1.56); heptosyltransferase (EC 2.4.-.-).	3378	4135	7514
GT90	UDP-Xyl: (mannosyl) glucuronoxylomannan/galactoxylomannan beta-1,2-xylosyltransferase (EC 2.4.2.-); UDP-Glc: protein O-beta-glucosyltransferase (EC 2.4.1.-); UDP-Xyl: protein O-beta-xylosyltransferase (EC 2.4.2.-)	896	512	1408

GT92	UDP-Gal: N-glycan core alpha-1,6-fucoside beta-1,4-galactosyltransferase (EC 2.4.1.-); UDP-Gal: beta-galactoside beta-1,4-galactosyltransferase (EC 2.4.1.-)	1605	896	2501
GT94	GDP-Man: GlcA-beta-1,2-Man-alpha-1,3-Glc-beta-1,4-Glc-alpha-1-PP-undecaprenol beta-1,4-mannosyltransferase (2.4.1.251)	715	696	1411
GT95	UDP-beta-L-Araf:hydroxyproline beta-L-arabinofuranosyltransferase (EC 2.4.2.-);	5	8	13
GT96	UDP-Gal: peptidyl serine alpha-galactosyltransferase (EC 2.4.1.-)	5	10	15
GT97	CMP-Neu5Ac:alpha-galactoside alpha-2,6-sialyltransferase (EC 2.4.99.-); CMP-Neu5Ac:alpha-glucoside alpha-2,6-sialyltransferase (EC 2.4.99.-);	3	3	6
GT99	CMP-beta-KDO 3-deoxy-beta-D-manno-oct-2-ulosonic acid transferase (EC 2.4.99.-)	74	158	232
PL1	pectate lyase (EC 4.2.2.2); exo-pectate lyase (EC 4.2.2.9); pectin lyase (EC 4.2.2.10).	1316	1560	2876
PL10	pectate lyase (EC 4.2.2.2)	18	123	141
PL10_1	PL10's subfamily	1439	1328	2767
PL10_2	PL10's subfamily	251	164	415
PL11	rhamnogalacturonan endolyase (EC 4.2.2.23); rhamnogalacturonan exolyase (EC 4.2.2.24)	2046	1672	3718
PL11_1	PL11's subfamily	2155	2447	4602
PL11_2	PL11's subfamily	61	44	105
PL12	heparin-sulfate lyase (EC 4.2.2.8)	83	83	165
PL12_1	PL12's subfamily	13	36	49
PL12_2	PL12's subfamily	13	20	34
PL12_3	PL12's subfamily	25	23	49
PL13	heparin lyase (EC 4.2.2.7)	349	225	574
PL14_2	PL14's subfamily	0	2	2

PL14_4	PL14's subfamily	1	2	2
PL15	oligo-alginate lyase (EC 4.2.2.-); alginate lyase (EC 4.2.2.3)	3	8	10
PL15_1	PL15's subfamily	0	0	0
PL16	hyaluronan lyase (EC 4.2.2.1).	3	2	5
PL17	alginate lyase (EC 4.2.2.3); oligoalginate lyase (EC 4.2.2.26)	23	11	34
PL17_1	PL17's subfamily	1	2	3
PL18	alginate lyase (EC 4.2.2.3); poly(alpha-L-guluronate) lyase / G-specific alginate lyase (EC 4.2.2.11); MG-specific alginate lyase (EC 4.2.2.-)	0	2	2
PL1_11	PL1's subfamily	0	1	1
PL1_2	PL1's subfamily	2738	2848	5586
PL1_3	PL1's subfamily	5	11	16
PL1_5	PL1's subfamily	14	48	62
PL1_7	PL1's subfamily	7	1	8
PL1_8	PL1's subfamily	20	47	67
PL2	pectate lyase (EC 4.2.2.2); exo-polygalacturonate lyase (EC 4.2.2.9).	5	6	11
PL21	heparin lyase (EC 4.2.2.7); heparin-sulfate lyase (EC 4.2.2.8); acharan-sulfate lyase (EC 4.2.2.-)	1	0	1
PL22	oligogalacturonate lyase / oligogalacturonide lyase (EC 4.2.2.6)	922	727	1649
PL22_1	PL22's subfamily	5	4	10
PL22_2	PL22's subfamily	39	34	73
PL24	ulvan lyase (EC 4.2.2.-).	1	1	1
PL25	ulvan lyase (EC 4.2.2.-).	29	38	66
PL26	rhamnogalacturonan exolyase (EC 4.2.2.24).	710	470	1179
PL27	L-rhamnose-alpha-1,4-D-glucuronate lyase (EC 4.2.2.-)	171	111	283

PL29	hyaluronate lyase (EC 4.2.2.1); chondroitin-sulfate ABC endolyase (EC 4.2.2.20); dermatan sulfate lyase (4.2.2.-); dermatan sulfate lyase (EC 4.2.2.-)	756	457	1213
PL3	pectate lyase (EC 4.2.2.2).	16	22	37
PL30	hyaluronate lyase (EC 4.2.2.1)	65	197	262
PL31	endo-beta-1,4-glucuronan lyase (EC 4.2.2.14)	102	49	151
PL32	poly(beta-mannuronate) lyase / M-specific alginate lyase (EC 4.2.2.3)	23	94	117
PL33	hyaluronate lyase (EC 4.2.2.1); gellan lyase (EC 4.2.2.25); chondroitin sulfate lyase (EC 4.2.2.20)	30	23	53
PL33_1	PL33's subfamily	99	78	177
PL33_2	PL33's subfamily	54	44	98
PL34	alginate lyase (EC 4.2.2.-)	1	0	1
PL35	chondroitin lyase / chondroitinase (EC 4.2.2.-); chondroitin AC lyase (EC 4.2.2.5)	38	34	72
PL37	chondroitin-sulfate ABC endolyase (EC 4.2.2.20); heparin-sulfate lyase / heparin lyase III (EC 4.2.2.8); ulvan lyase (EC 4.2.2.-)	258	664	921
PL3_1	PL3's subfamily	0	2	2
PL3_2	PL3's subfamily	0	1	1
PL3_3	PL3's subfamily	1	1	2
PL4	rhamnogalacturonan endolyase (EC 4.2.2.23).	77	33	110
PL4_1	PL4's subfamily	4	10	13
PL4_2	PL4's subfamily	1	0	1
PL4_3	PL4's subfamily	3	0	3
PL4_4	PL4's subfamily	83	91	174
PL4_5	PL4's subfamily	11	6	17
PL5	alginate lyase (EC 4.2.2.3).	175	100	276

PL6	alginate lyase (EC 4.2.2.3); chondroitinase B (EC 4.2.2.19); MG-specific alginate lyase (EC 4.2.2.-); poly(alpha-L-guluronate) lyase / G-specific alginate lyase (EC 4.2.2.11);	263	187	450
PL6_1	PL6's subfamily	344	342	686
PL6_2	PL6's subfamily	26	10	36
PL6_3	PL6's subfamily	3	4	7
PL7	poly(beta-mannuronate) lyase / M-specific alginate lyase (EC 4.2.2.3); alpha-L-guluronate lyase / G-specific alginate lyase (EC 4.2.2.11); poly-(MG)-lyase / MG-specific alginate lyase (EC 4.2.2.-); endo-beta-1,4-glucuronan lyase (EC 4.2.2.14)	12	0	12
PL7_2	PL7's subfamily	1	0	1
PL7_3	PL7's subfamily	17	11	29
PL7_4	PL7's subfamily	2	4	7
PL8	hyaluronate lyase (EC 4.2.2.1); chondroitin AC lyase (EC 4.2.2.5); xanthan lyase (EC 4.2.2.12); chondroitin ABC lyase (EC 4.2.2.20)	237	161	399
PL8_2	PL8's subfamily	4	12	16
PL8_3	PL8's subfamily	252	255	508
PL9	pectate lyase (EC 4.2.2.2); exopolygalacturonate lyase (EC 4.2.2.9); thiopeptidoglycan lyase (EC 4.2.2.-).	663	934	1597
PL9_1	PL9's subfamily	1496	1105	2601
PL9_2	PL9's subfamily	259	327	586
PL9_3	PL9's subfamily	8	1	10
PL9_4	PL9's subfamily	12	4	16
